# Computational study of productive and non-productive cycles in fluoroalkene metathesis

**DOI:** 10.3762/bjoc.11.232

**Published:** 2015-11-10

**Authors:** Markéta Rybáčková, Jan Hošek, Ondřej Šimůnek, Viola Kolaříková, Jaroslav Kvíčala

**Affiliations:** 1 Department of Organic Chemistry, University of Chemistry and Technology, Technická 5, 166 28 Prague 6, Czech Republic

**Keywords:** alkene metathesis, computation, DFT, fluoroalkene, mechanism, non-productive cycle, productive cycle

## Abstract

A detailed DFT study of the mechanism of metathesis of fluoroethene, 1-fluoroethene, 1,1-difluoroethene, *cis*- and *trans*-1,2-difluoroethene, tetrafluoroethene and chlorotrifluoroethene catalysed with the Hoveyda–Grubbs 2^nd^ generation catalyst was performed. It revealed that a successful metathesis of hydrofluoroethenes is hampered by a high preference for a non-productive catalytic cycle proceeding through a ruthenacyclobutane intermediate bearing fluorines in positions 2 and 4. Moreover, the calculations showed that the cross-metathesis of perfluoro- or perhaloalkenes should be a feasible process and that the metathesis is not very sensitive to stereochemical issues.

## Introduction

Over the course of the last 20 years, alkene metathesis catalysed with homogeneous transitition metal-based precatalysts evolved into a valuable tool for organic synthetic chemists mainly due to its variability and high compatibility with functional groups. It hence became the subject of multiple books [1–3] and reviews [4–8] discussing its synthetic applications, catalysts, mechanism, regio- and stereoselectivity.

Computational chemistry proved to be extremely valuable in the study of reaction mechanisms. In particular, the use of time-efficient DFT methods for the theoretical study of alkene metathesis has been extensively reviewed [9–11] and computational results have been found to agree well with recent experimental mechanistic studies based on easily initiating ruthenium precatalysts [12–13]. A theoretical approach has been also employed in attempts to gain a better insight into the complex structure of intertwined productive and non-productive catalytic cycles of alkene metathesis [14]. In contrast to the older computations, new publications also include the initiation steps starting from metathesis precatalysts as Grubbs or Hoveyda–Grubbs 2^nd^ generation precatalysts [15–22].

In contrast to common alkenes, the metathesis of fluoroalkenes has attracted far less attention [23]. Fluorinated modifications have mostly concentrated on the side chain of the vinyl group [24–27] or applications of 2-fluoroalkenes [28–30]. As an exception, the reaction of the Grubbs 2^nd^ generation catalyst with 1,1-difluoroethene gave an isolable difluoromethylene-containing ruthenium complex with very poor catalytic activity [31] and the analogous reaction of 1-fluoroalkene formed a fluoromethylene-containing complex with low catalytic activity [32–33]. Up to now, the only metathesis which included tetrafluoroethene and its analogues has been reported in a patent [34], describing the disproportionation of perfluoroalkenes and alkenes to hydrofluoroalkenes. Moreover, just recently a successful cross metathesis of perfluoroalkenes with vinyl ethers has been published [35].

The reported computations dealing with the metathesis of fluoroalkenes are also extremely scarce. Thus, the mechanism of the cross metathesis of norbornene with selected fluoro- and chloroalkenes has been studied and the higher stability of a ruthenium intermediate containing a difluoromethylene ligand has been emphasized [36–37].

A complete mechanism of alkene metathesis including initiation, productive and non-productive cycles represents a highly complex system [38], the understanding of which for fluoroalkenes is negligible. We hence report herein the results of a computational study dealing with the metathesis of most fluoroethenes with the emphasis on subsequent catalytic cycles, catalysed with the Hoveyda–Grubbs 2^nd^ generation precatalyst (**HG2**).

## Results and Discussion

**Scheme 1:**
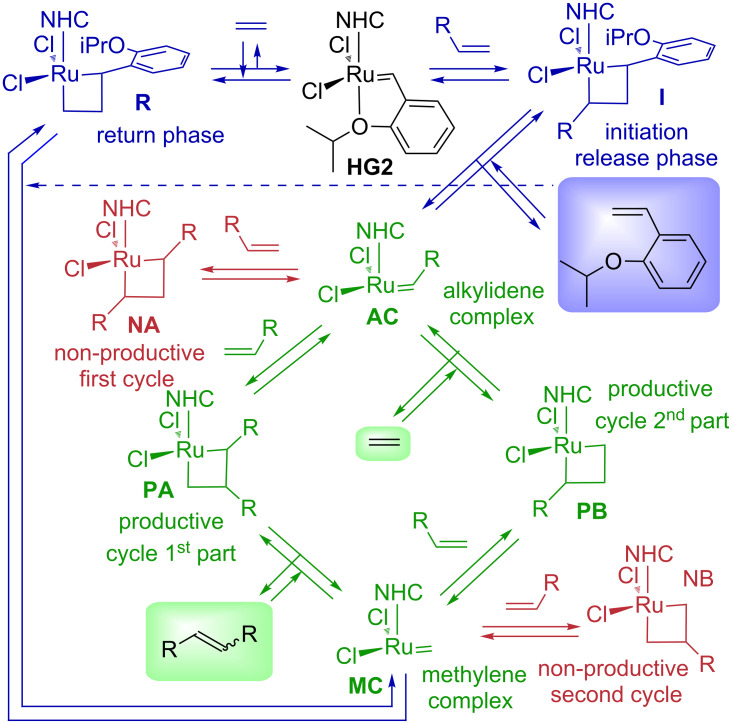
Initiation, productive and non-productive cycles in alkene homometathesis.

In contrast to textbook pictures describing alkene metathesis as a single catalytic cycle, even a simplified system of homometathesis of 1-alkene represents a complex system, in any step of which problems can arise due to a high energetical barrier or unfavourable equilibrium. Moreover, the preference for non-productive cycles can hamper the formation of the desired product even in the absence of kinetic and thermodynamic issues (Scheme 1).

At the beginning, the starting precomplex **HG2** reacts in the initiation phase with alkene forming ruthenacyclobutane **I**, which releases 2-isopropoxystyrene and the first active catalytic species, alkylideneruthenium complex **AC**. Depending on the regioselectivity of the coordination of the second alkene molecule, the **AC** complex can form ruthenacyclobutane **PA** in the first part of productive catalytic cycle, or symmetrical ruthenacyclobutane **NA** in the first part of the non-productive catalytic cycle. While the first species **PA** reacts further to the product and methyleneruthenium complex **MC**, the non-productive ruthenacyclobutane **NA** can only return back to complex **AC**. Intermediary complex **MC** can again enter either the second part of the productive catalytic cycle closing the ring into the ruthenacyclobutane **PB**, or it can react in the non-productive cycle forming symmetrical ruthenacyclobutane **NB**. Finishing the productive cycle, the ruthenacyclobutane **PB** releases ethene and starts the next catalytic cycle, while the non-productive complex **NB** can again only return back to complex **MC**. It should be noted that the main aim of the development of the family of Hoveyda–Grubbs catalysts was the recycling of the catalyst. This implies a successful release-return mechanism, in which after a successful metathesis the active complexes **AC** or **MC** react with 2-isopropoxystyrene restoring the starting precatalyst **HG2**, a controversial issue, which some authors support [8,39] and the others contest [40]. Recent experiments have shown that the complex **MC** can be successfully transformed into the parent precatalyst **HG2** [41].

**Figure 1:**
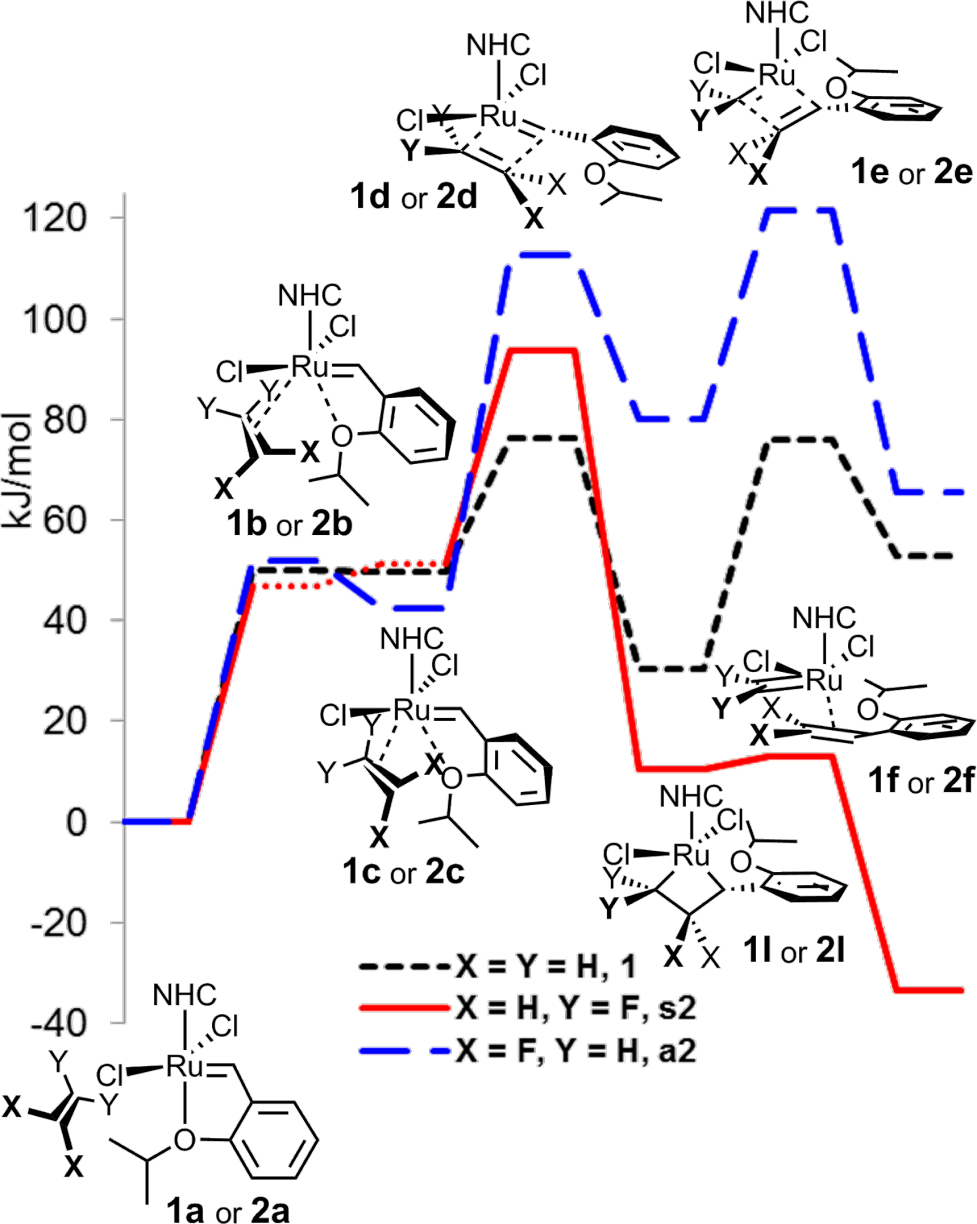
Initiation phase of the reaction of **HG2** with ethene (**1**) and 1,1-difluoroethene (**2**).

In our computational study, we first addressed the initiation phase of possible metathesis of a highly unsymmetrical alkene, 1,1-difluoroethene, and compared its behaviour with the already reported initiation of ethene [18–19]. Among the three possible mechanisms, interchange, dissociative and associative, the first emerged as the most energetically favourable. In contrast to the symmetrical molecule of ethene, two orientations of 1,1-difluoroethene are possible, which we arbitrarily assigned as *syn* for the coordination of difluoromethylene to ruthenium forming 2,2-difluororuthenacyclobutane intermediate **s2I** and *anti* for the coordinaton of methylene to ruthenium forming 3,3-difluororuthenacyclobutane intermediate **a2I**. The computations started from an alkene weakly coordinated to the NHC ligand without any coordination to ruthenium (structures **1a** and **2a**), and continued with the first mechanistic step, the coordination of alkene to ruthenium with partial decoordination of alkoxybenzylidene oxygen (structures **1c** and **2c**). For ethene (**1c**) and the *anti-*coordinated 1,1-difluoroethene (**a2c**), shallow minima were observed in the Gibbs free energy profile, while for the *syn* structure (**s2c**), the minimum obtained by the calculation of electronic energy changed just to inflexion when converted to free Gibbs energy at 25 °C (see Figure 1, dotted red line). At this stage only minimal relative energy differences were observed for the structures **1c** and **2c**. However, in the next step forming metallacyclobutane the transition state energy was by ca. 20 kJ/mol higher for the *syn*-coordinated 1,1-difluoroethene (**s2d**) and by another ca. 20 kJ/mol higher for the *anti*-coordinationed 1,1-difluoroethene (**a2d**) compared to ethene (**1d**), already energetically preferring the *syn*-coordination. The picture changed dramatically on the formation of metallacyclobutane (**1I** or **2I**), where 2,2-difluororuthenacyclobutane intermediate **s2I** was by 20 kJ/mol more stable than ruthenacyclobutane **s1** and by another 40 kJ/mol more stable than 3,3-difluororuthenacyclobutane **a2I**. These differences further rose for the last step of initiation, the formation of methyleneruthenium **1f** or **a2f** or difluoromethyleneruthenium **s2f**, where the relative stability of the latter was by 100 kJ/mol higher compared to the *anti* coordination. These results are in agreement with the previous computations of a norbornene derivative with fluoroalkenes [36–37], but are suprisingly contradictory to the experimental observations of the reaction of the Grubbs 2^nd^ generation catalyst with 1,1-difluoroethene, where at room temperature the formation of both methylene- and difluoromethyleneruthenium complexes was observed [31].

**Figure 2:**
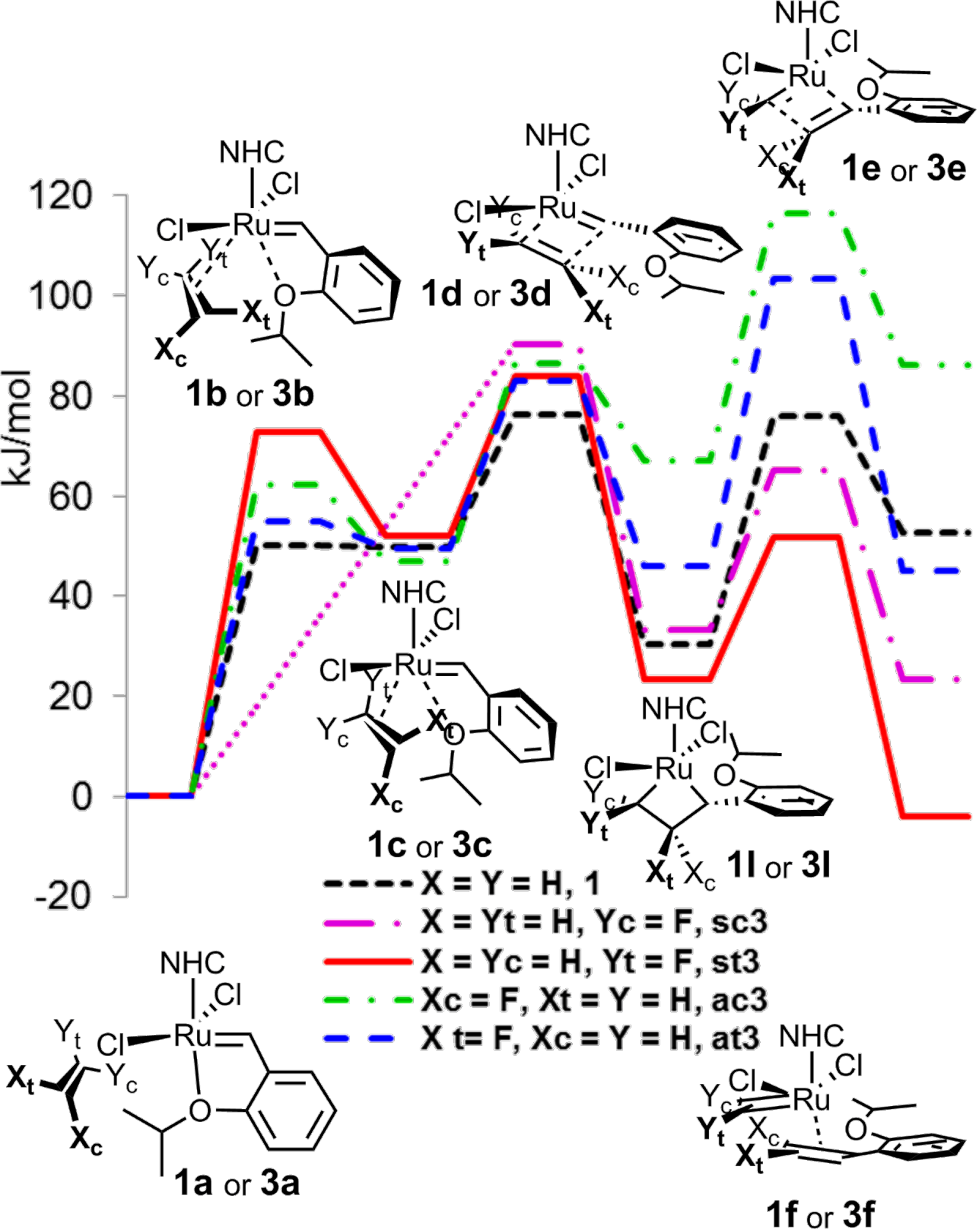
Initiation phase of the reaction of **HG2** with ethene (**1**) and 1-fluoroethene (**3**).

With the additional aim to obtain the information about stereoselectivity, we next analogously computed the initation phase of the reaction of precatalyst **HG2** with 1-fluoroethene, where apart of the *syn*- and *anti-*approach, also *cis*- or *trans*- orientations of the alkene relatively to the alkoxyphenyl ring in the intermediary metallacyclobutane are possible. In the first step of the reaction, intermediary complex **3c** with partial bonding of alkene and the alkoxy group of the alkoxybenzylidene ligand to ruthenium was detected with the exception of a *syn–cis* arranged alkene, for which just a weak inflexion was observed in analogy to [42]. No large differences in the energies of transition states **3d** preceeding the formation of metallacyclobutane were found, but in analogy to the initiation of 1,1-difluoroethene, 2-fluorometallacyclobutanes **sc3I** and **st3I** with coordination of fluoromethylene to ruthenium were again more stable then the corresponding 3-fluorometallacyclobutanes **ac3I** and **at3I**. The difference is further augmented in the transition state **3e** and in the final stage of the initiation, the formation of fluoromethyleneruthenium complex **s3f** or methyleneruthenium complex **a3f**. On the other hand, this difference in the energies reaches ca. 50 kJ/mol, about one half of the *syn*–*anti* difference for difluoromethylated complex **2AC**. We also observed significant differences in the energies for *cis*- and *trans*-ruthenacyclobutanes **c3I** and **t3I** and complexes **c3f** and **t3f**, where the *trans*-structures were more stable by 25 to 60 kJ/mol, probably due to the repulsion of fluorine with the alkoxybenzylidene oxygen (Figure 2).

**Figure 3:**
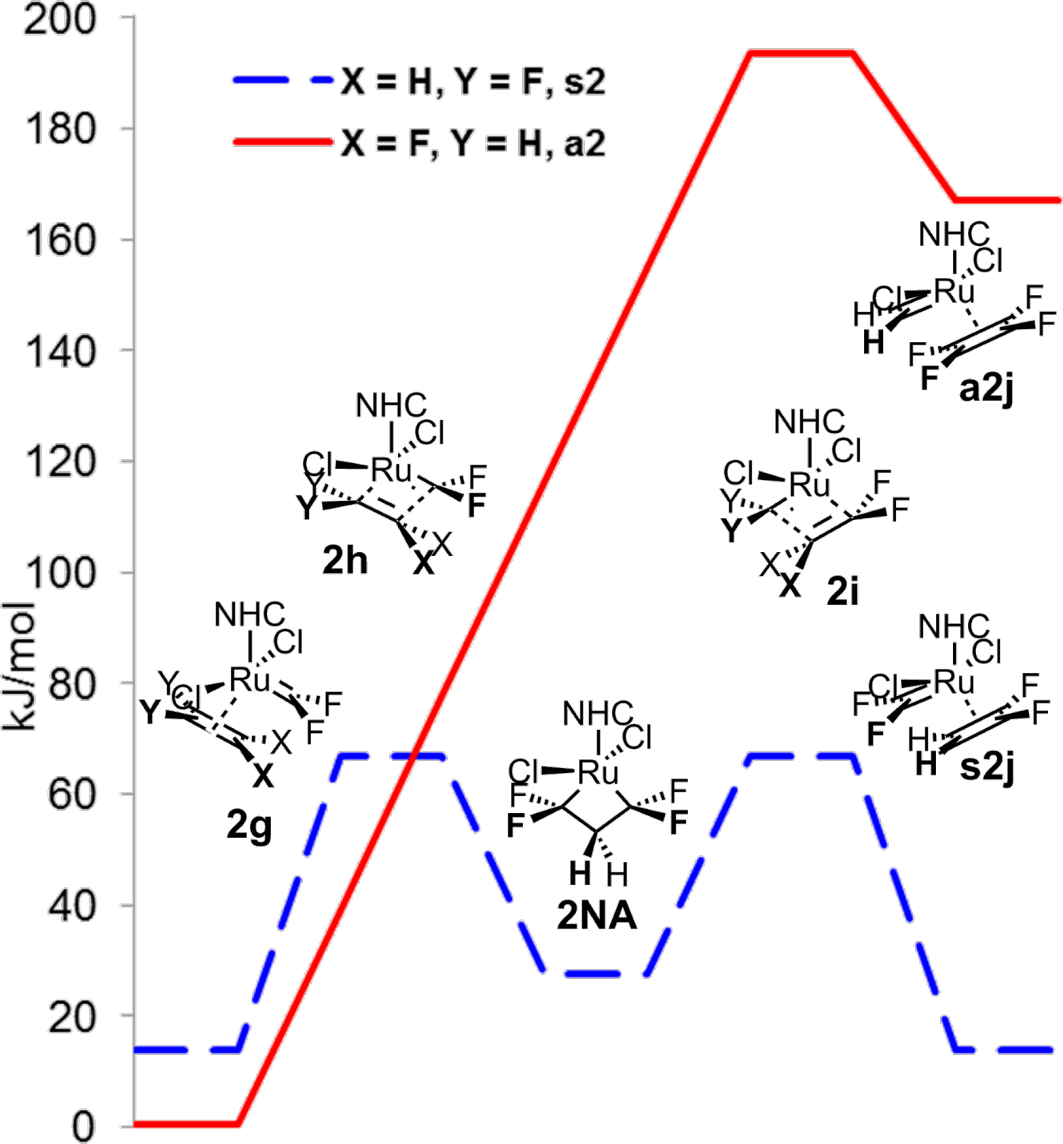
First part **A** of the catalytic cycle of homometathesis of 1,1-difluoroethene (**2**).

We continued our study by the computation of the first step **A** of the catalytic cycle for 1,1-difluoroethene (**2**), which started with the coordination of the starting active catalytic form **2AC** with 1,1-difluoroethene (**2**). The non-productive cycle started with the *syn*-coordination of 1,1-difluoroethene (**2**) to 1,1-difluoromethyleneruthenium complex **2AC**, leading to symmetrical metallacyclobutane **2NA** with activation energy around 65 kJ/mol and through the same transition state back to complex **2AC** and 1,1-difluoroethene (**2**). However, for the productive *anti*-coordination of 1,1-difluoroethene (**2**), no stable metallacyclobutane structure **2PC** was found (a detailed study detected only inflection on the potential energy surface), probably due to a steep rise in the energy leading through the transition state **s2i** to a highly unstable complex of methyleneruthenium with tetrafluoroethene. The comparison of the productive and non-productive cycle shows that the transition state energies differ by more than 120 kJ/mol, making thus the first part **A** of the productive cycle highly improbable and probably resulting in stopping the productive metathesis of vinylidene fluoride at all, because all active catalytic species **AC** move forth and back in the non-productive cycle (Figure 3).

**Figure 4:**
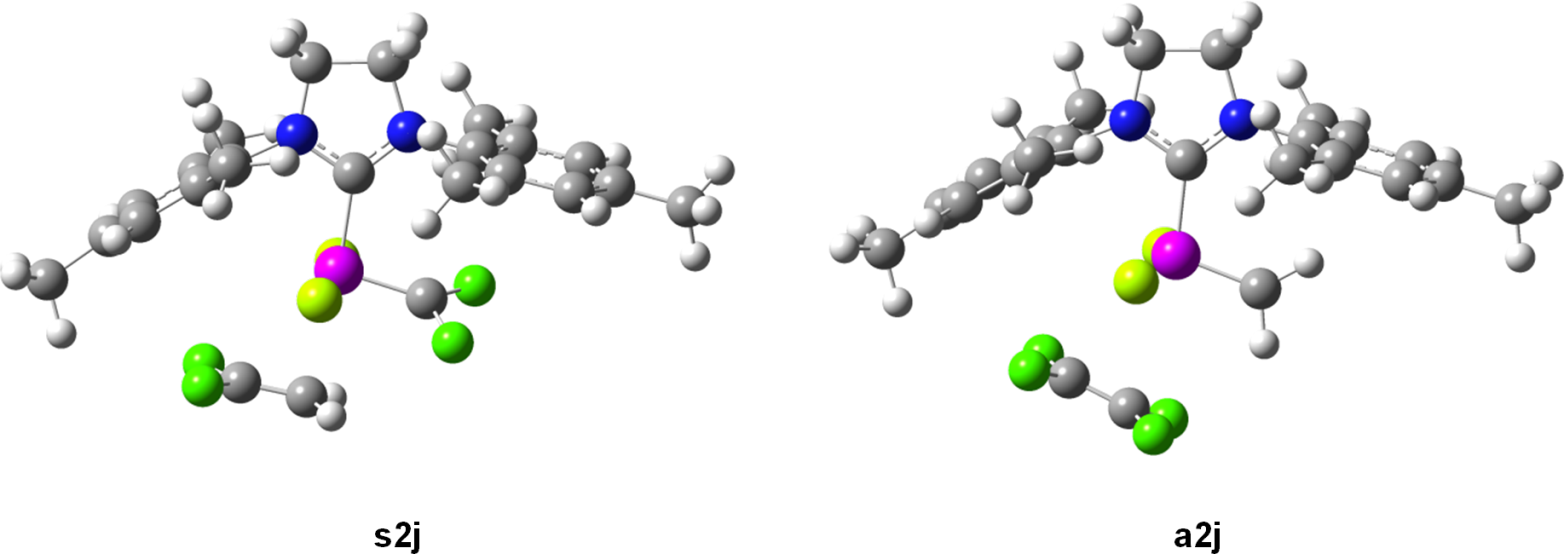
Computed structures of complexes **s2j** and **a2j**.

The difference in the stability of the corresponding complexes **a2j** and **s2j** can be explained partially by the π-donation of difluoromethylene carbene in analogy to [37], but also by the electron donation of the π-bond of the 1,1-difluoroethene (**2**) molecule with a high negative charge on the CH_2_ group. This results in lowering of the positive charge on ruthenium and shortening of the CH_2_–Ru distance (262 pm) in the **s2j** structure compared to repulsive CF_2_–Ru interaction of tetrafluoroethene in the **a2j** structure with longer CF_2_–Ru distance (323 pm, Figure 4).

**Figure 5:**
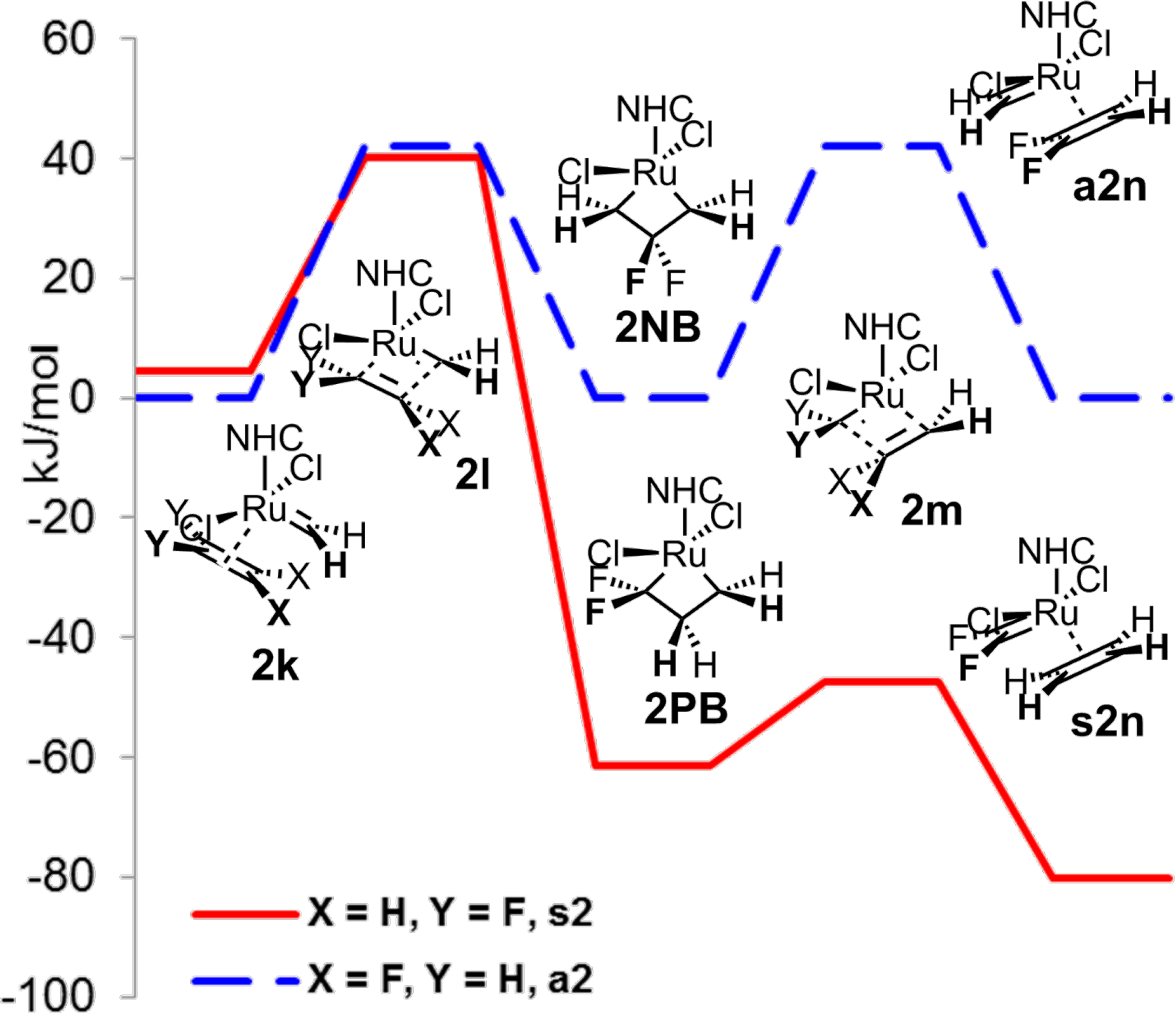
Second part **B** of the catalytic cycle of homometathesis of 1,1-difluoroethene (**2**).

We continued the calculations by the study of the second part **B** of the catalytic cycle of the metathesis of 1,1-difluoroethene (**2**), which starts by the decoordination of tetrafluoroethene from complex **a2j** and coordination of another molecule of 1,1-difluoroethene (**2**) to methyleneruthenium complex **MC** forming complex **2k**. While both *syn*- and *anti*-coordinations of starting complex **2k** and subsequent transition states **2l** have nearly equal energies, the subsequent non-symmetrical productive metallacyclobutane **2PB** is significantly more stable by ca. 60 kJ/mol than symmetrical non-productive metallacyclobutane **2NB**. The difference in the energies was again more augmented for the subsequent transition states **2m** and finally with the alkene coordinated to the alkylidene ruthenium, where the productive complex **s2n**, a complex of ethylene with difluororuthenium, is by ca. 80 kJ/mol more stable than the non-productive complex **a2n** (Figure 5). Thus, in the second part **B** of the catalytic cycle the non-productive cycle has no negative influence on the 1,1-difluoroethene (**2**) metathesis.

**Figure 6:**
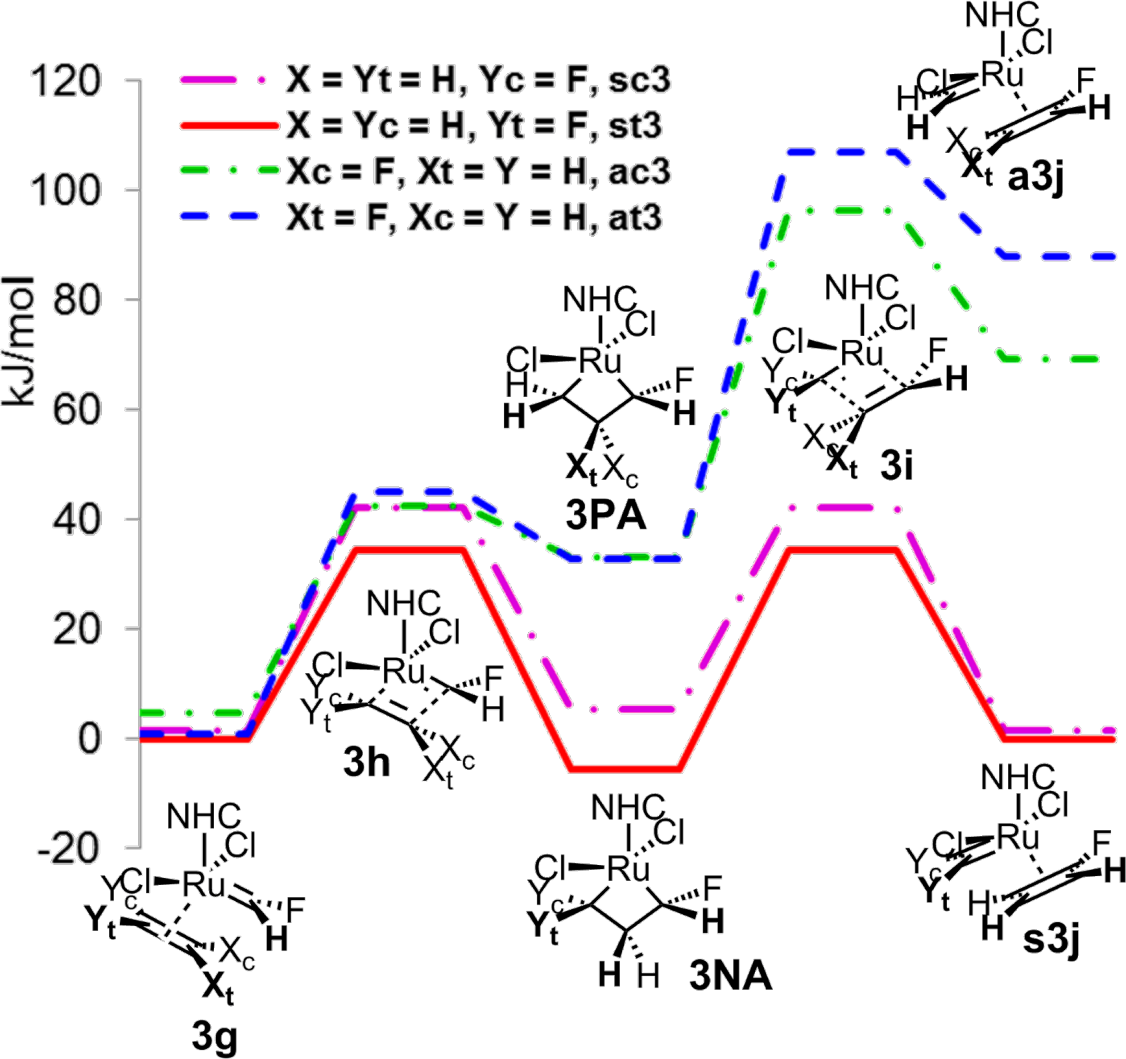
First part **A** of the catalytic cycle of homometathesis of 1-fluoroethene (**3**).

To obtain the information about the stereoselectivity in the active catalytic cycle, we again decided to study the productive and non-productive cycles in the homometathesis of 1-fluoroethene (**3**). In the first step **A** of the catalytic cycle, a similar pattern, although less emphasized, could be observed for *syn*- and *anti*-coordination of the starting 1-fluoroethene (**3**) as in the case of 1,1-difluoroethene (**2**). Thus, the relative energies of the complexes of 1-fluoroethene (**3**) with fluoromethyleneruthenium **3g**, as well as the subsequent transition states **3h**, differ minimally regardless of the regio- and stereoselectivity, while the intermediary symmetrical metallacyclobutane **3NA** of the non-productive cycle with the *syn*-coordination of 1-fluoroethene (**3**) shows a significantly higher stability compared to the productive *anti*-intermediate **3PA**. These differences again increased for the transition states **3i** and final complexes **3j**, preferring strongly the non-productive cycle and probably significantly slowing the possible productive metathesis. The calculations also show only low stereoselectivity with the transition states of the non-productive cycle preferring *trans*-configuration of both fluorine atoms by ca. 10 kJ/mol, while for the productive cycle the *cis*-configuration is preferred (Figure 6).

**Figure 7:**
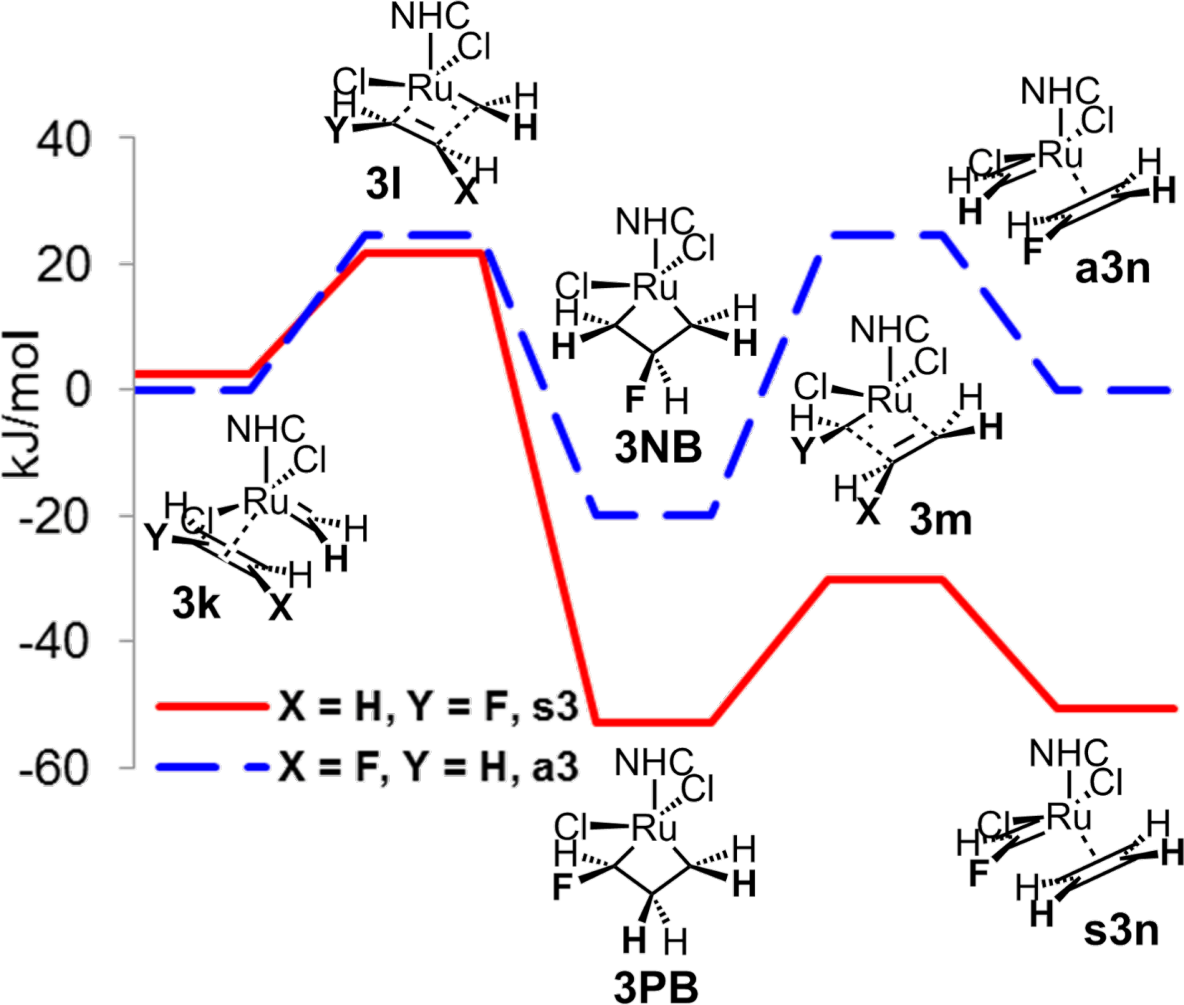
Second part **B** of the catalytic cycle of homometathesis of 1-fluoroethene (**3**).

For the homometathesis of 1-fluoroethene (**3**), we finally studied the second part **B** of the catalytic cycle. In analogy to the homometathesis of 1,1-difluoroethene (**2**), the productive cycle is energetically more favourable, indicating that in the part **B** the non-productive cycle does not block the catalytic cycle (Figure 7).

The obtained results imply that the key problem in the metathesis of non-symmetrically substituted fluoroalkenes is probably not the high stability of the fluorinated methyleneruthenium complex, but the consumption of most of the active catalytic form by the non-productive cycle proceeding through symmetrical metallacyclobutane substituted with fluorines in positions 2 and 4 of the ring.

**Figure 8:**
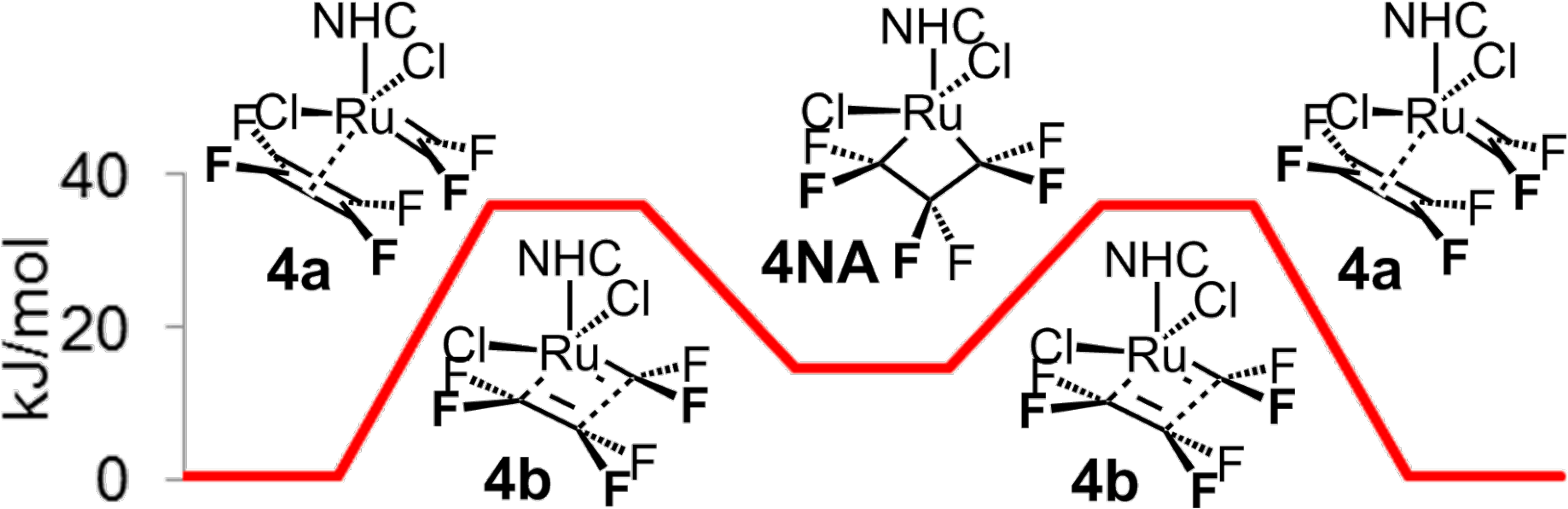
Non-productive catalytic cycle of homometathesis of tetrafluoroethene (**4**).

To further confirm this hypothesis, we decided to study the active catalytic cycle of the metathesis of two perhaloethenes, tetrafluoroethene (**4**) and chlorotrifluoroethene (**5**), starting from the active catalytic form **2AC**. Due to symmetry, both parts **A** and **B** of the catalytic cycle for tetrafluoroethene (**4**) are identical and non-productive with surprisingly low transition state energy (Figure 8).

**Figure 9:**
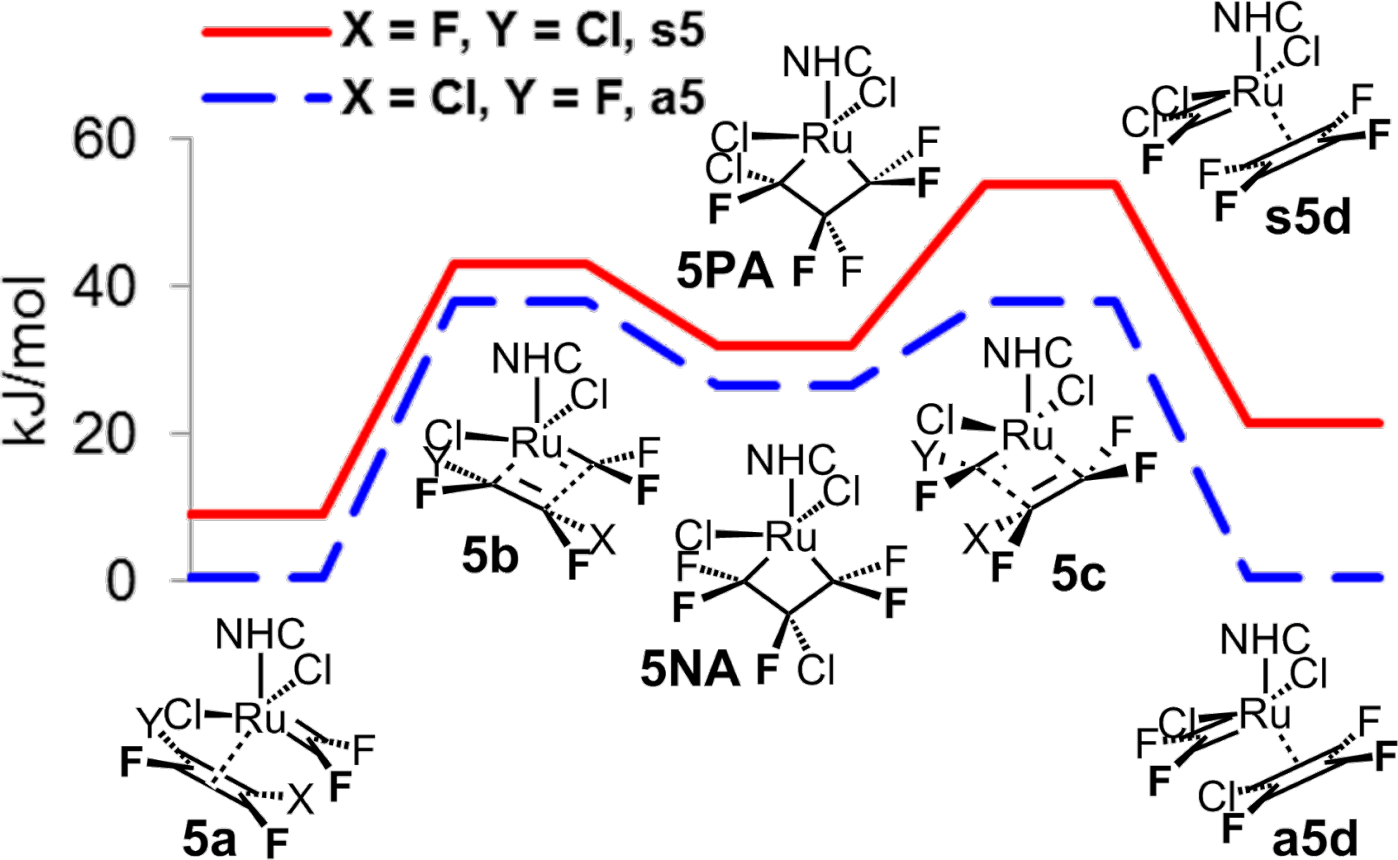
First part **A** of the catalytic cycle of homometathesis of chlorotrifluoroethene (**5**).

For the homometathesis of chlortrifluoroethene (**5**), the situation again becomes more complex with two parts **A** and **B** of the catalytic cycle and both productive and non-productive cycles participating. The first part **A** is in analogy to the homometathesis of tetrafluoroethene (**4**) characteristic by the low energy of the transition state, with the preference for the non-productive cycle of ca. 20 kJ/mol, i.e., much less pronounced than in the case of 1-fluoroethene (**3**) complexes (Figure 6 and Figure 9).

**Figure 10:**
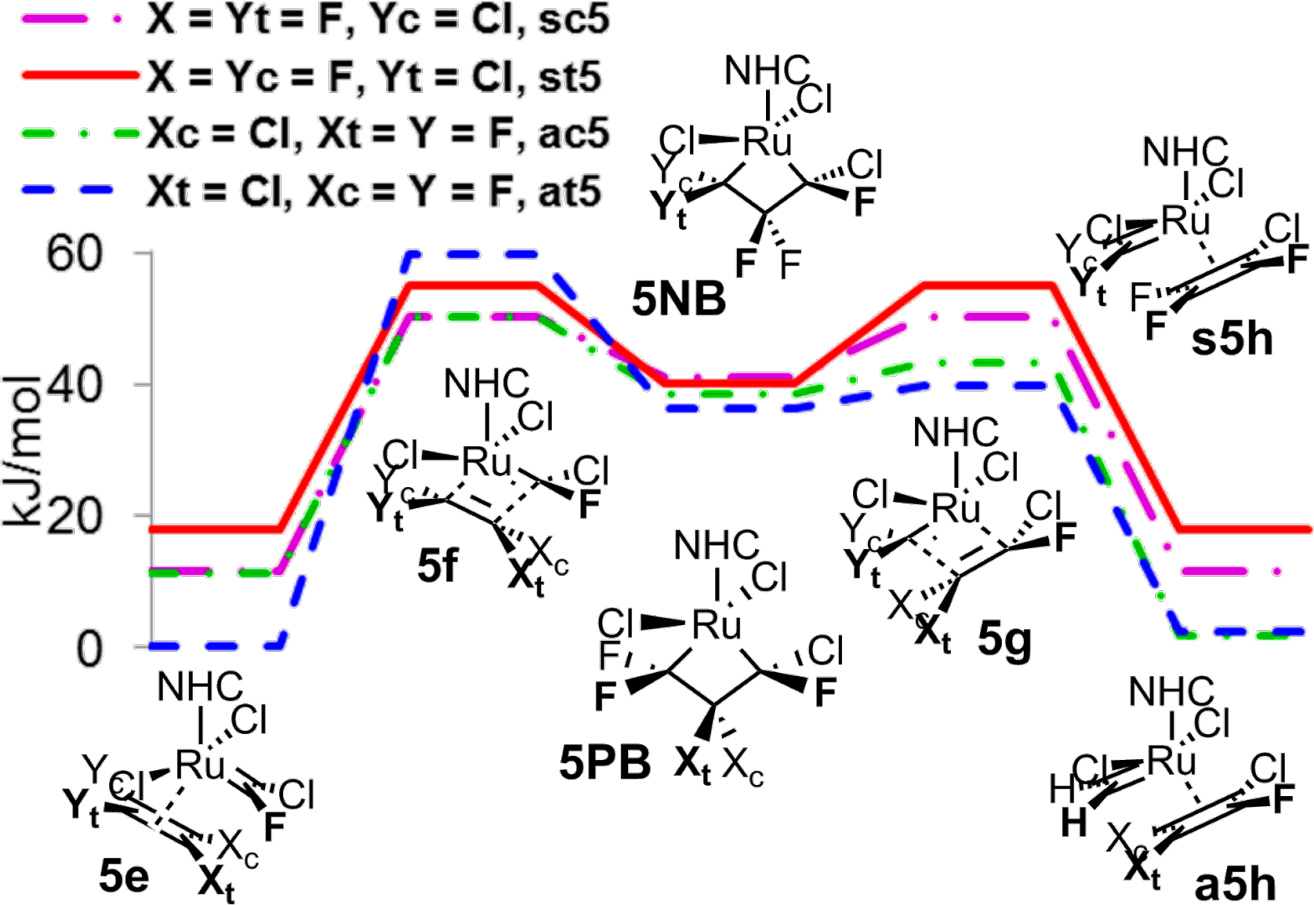
Second part **B** of the catalytic cycle of homometathesis of chlorotrifluoroethene (**5**).

With the second part **B** of the catalytic cycle starting from a non-symmetrical chlorofluoromethyleneruthenium complex, the stereochemistry of coordination in complex **5e** became an issue with *cis*- and *trans*-ruthenacyclobutanes possible. In analogy to the homometathesis of tetrafluoroethene (**4**), the relative transition state energies were quite low with small preference for the productive cycle without any stereochemical priority (Figure 10).

## Conclusion

Our computational study, which included the metathesis of both partially and fully fluorinated alkenes, showed that the formation of stable intermediary fluoro- or difluoromethyleneruthenium does not block the subsequent metathetic cycles. For partially fluorinated alkenes as 1,1-difluoroethene (**2**) or 1-fluoroethene (**3**), poor preparative results of metathesis can be caused by the overwhelming participation of the non-productive metathetic cycle proceeding through ruthenacyclobutanes substituted with fluorine atoms in positions 2 and 4. On the other hand, the results of computations of the catalytic cycles of both tetrafluoroethene (**4**) and chlorotrifluoroethene (**5**) indicate that their metathesis should proceed without any significant problems providing no reactive alkylideneruthenium complexes, e.g., methyleneruthenium, participates in the active catalytic cycles. Our results are in full agreement with the recently described surprisingly successful metathesis of perhaloalkenes with vinyl ethers [35], but contradicts the patent [34] which describes the successful synthesis of partially fluorinated alkenes from perfluorinated and non-fluorinated alkenes.

## Computational Details

DFT calculations were performed using the Gaussian 09W program suite [43] using the resolution-of-identity approach [44], M06L pure functional [45] in analogy to [18], def2-SV(P) basis set [46] and universal def2 auxilliary basis set [47]. Vibrational frequencies were calculated for all structures to characterize them as minima or transition states. These computations gave their free Gibbs energies at 25 °C, which were used for the PES description in Figures 1–3 and Figures 5–10. Starting geometries were obtained by a careful series of preoptimization of structures **1a**–**3a** (Hoveyda–Grubbs 2^nd^ generation precatalyst **HG2** with weakly coordinated alkene) and **1l**–**3l** (metallacyclobutane) with the most stable conformation of the isopropoxy group differing from the crystal structure of the parent precatalyst **HG2**. For all computed structures, the corresponding pdb files are listed in Supporting Information File 1 together with a table containing the total electronic and free Gibbs energies in hartrees, and the total and relative electronic and free Gibbs energies in kJ/mol.

## Supporting Information

File 1 Table containing total electronic and free Gibbs energies in hartrees, total and relative electronic and free Gibbs energies in kJ/mol for all computed structures, as well as their coordinates in the pdb format.
