# Supplementary material for: Computational study of productive and non-productive cycles in fluoroalkene metathesis
Source: Beilstein J Org Chem. 2015 Nov 10;11:2150–7. doi: 10.3762/bjoc.11.232 (PMC4660995; doi:10.3762/bjoc.11.232)
Supplement: File 1 — Table containing total electronic and free Gibbs energies in hartrees, total and relative electronic and free Gibbs energies in kJ/mol for all computed structures, as well as their coordinates in the pdb format. [file Beilstein_J_Org_Chem-11-2150-s001.pdf]

Supporting Information

for

Computational study of productive and non-productive cycles in fluoroalkene metathesis

Markéta Rybáčková, Jan Hošek, Ondřej Šimůnek, Viola Kolaříková and Jaroslav Kvíčala*

Address: Department of Organic Chemistry, University of Chemistry and Technology, Technická 5, 166 28 Prague 6, Czech Republic

Email: Jaroslav Kvíčala - kvicalaj@vscht.cz

*Corresponding author

Table containing total electronic and free Gibbs energies in hartrees, total and relative electronic and free Gibbs energies in kJ/mol for all computed structures, as well as their coordinates in the pdb format.

Table S1. Total electronic and free Gibbs energies in hartrees, total and relative electronic and free Gibbs energies in kJ/mol for all computed structures.

Structure	Total electronic energy (hartree)	Total free Gibbs energy (hartree)	Total electronic energy (kJ/mol)	Relative electronic energy (kJ/mol)	Total free Gibbs energy (kJ/mol)	Relative free Gibbs energy (kJ/mol)
**1a**	-2482.573127	-2481.977548	-6517995.75	1.50	-6516432.05	**0.00**
**1b**	-2482.557065	-2481.958447	-6517953.57	43.67	-6516381.90	**50.15**
**1c**	-2482.559513	-2481.958603	-6517960.00	37.25	-6516382.31	**49.74**
**1d**	-2482.552073	-2481.948530	-6517940.47	56.78	-6516355.87	**76.19**
**1I**	-2482.571286	-2481.965937	-6517990.91	6.34	-6516401.57	**30.48**
**1e**	-2482.553076	-2481.948593	-6517943.10	54.15	-6516356.03	**76.02**
**1f**	-2482.559890	-2481.957404	-6517960.99	36.26	-6516379.16	**52.89**
**s2a**	-2680.988342	-2680.403412	-7038934.89	35.93	-7037399.16	**33.32**
**s2b**	-2680.968034	-2680.385592	-7038881.57	89.25	-7037352.37	**80.11**
**s2c**	-2680.968461	-2680.383870	-7038882.69	88.13	-7037347.85	**84.63**
**s2d**	-2680.955706	-2680.367753	-7038849.21	121.61	-7037305.54	**126.95**
**s2I**	-2680.986839	-2680.399458	-7038930.95	39.87	-7037388.78	**43.70**
**s2e**	-2680.985199	-2680.398466	-7038926.64	44.18	-7037386.17	**46.31**
**s2f**	-2681.002026	-2680.416104	-7038970.82	0.00	-7037432.48	**0.00**
**a2a**	-2680.986070	-2680.401973	-7038928.93	41.89	-7037395.38	**37.10**
**a2b**	-2680.965169	-2680.382171	-7038874.05	96.77	-7037343.39	**89.09**
**a2c**	-2680.968993	-2680.385796	-7038884.09	86.73	-7037352.91	**79.57**

**a2d**	-2680.948949	-2680.359077	-7038831.47	139.35	-7037282.76	**149.72**
**a2I**	-2680.963854	-2680.371511	-7038870.60	100.22	-7037315.40	**117.08**
**a2e**	-2680.942048	-2680.355736	-7038813.35	157.47	-7037273.98	**158.50**
**a2f**	-2680.960824	-2680.377057	-7038862.64	108.18	-7037329.96	**102.52**
**sc3a**	-2581.780048	-2581.190987	-6778463.51	0.00	-6776916.94	**0.00**
**sc3b**	n.a.	n.a.	n.a.	n.a.	n.a.	n.a.
**sc3c**	n.a.	n.a.	n.a.	n.a.	n.a.	n.a.
**sc3d**	-2581.750821	-2581.156613	-6778386.78	76.73	-6776826.69	**90.25**
**sc3I**	-2581.775253	-2581.178330	-6778450.93	12.59	-6776883.71	**33.23**
**sc3e**	-2581.761722	-2581.166154	-6778415.40	48.12	-6776851.74	**65.20**
**sc3f**	-2581.776091	-2581.182029	-6778453.13	10.39	-6776893.42	**23.52**
**st3a**	-2581.777829	-2581.187167	-6778457.69	5.83	-6776906.91	**10.03**
**st3b**	-2581.756024	-2581.159477	-6778400.44	63.07	-6776834.21	**82.73**
**st3c**	-2581.761868	-2581.167358	-6778415.78	47.73	-6776854.90	**62.04**
**st3d**	-2581.747896	-2581.155150	-6778379.10	84.41	-6776822.85	**94.09**
**st3I**	-2581.775970	-2581.178282	-6778452.81	10.71	-6776883.58	**33.36**
**st3e**	-2581.763141	-2581.167459	-6778419.13	44.39	-6776855.16	**61.77**
**st3f**	-2581.779831	-2581.188713	-6778462.95	0.57	-6776910.97	**5.97**
**ac3a**	-2581.771516	-2581.185848	-6778441.11	22.40	-6776903.44	**13.49**
**ac3b**	-2581.754684	-2581.162093	-6778396.92	66.59	-6776841.08	**75.86**
**ac3c**	-2581.761055	-2581.167999	-6778413.65	49.86	-6776856.58	**60.35**
**ac3d**	-2581.748141	-2581.152903	-6778379.75	83.77	-6776816.95	**99.99**
**ac3I**	-2581.760538	-2581.160331	-6778412.29	51.22	-6776836.45	**80.49**
**ac3e**	-2581.737046	-2581.141452	-6778350.61	112.90	-6776786.88	**130.05**
**ac3f**	-2581.747783	-2581.153042	-6778378.80	84.71	-6776817.31	**99.62**
**at3a**	-2581.773076	-2581.183512	-6778445.21	18.30	-6776897.31	**19.63**
**at3b**	-2581.756790	-2581.162633	-6778402.45	61.06	-6776842.49	**74.44**
**at3c**	-2581.758188	-2581.164675	-6778406.12	57.39	-6776847.85	**69.08**
**at3d**	-2581.746289	-2581.151835	-6778374.88	88.63	-6776814.14	**102.79**
**at3I**	-2581.761737	-2581.166037	-6778415.44	48.07	-6776851.43	**65.51**
**at3e**	-2581.738830	-2581.144079	-6778355.30	108.22	-6776793.78	**123.16**
**at3f**	-2581.760321	-2581.166387	-6778411.72	51.79	-6776852.35	**64.59**
**s2g**	-2455.278100	-2454.869732	-6446332.65	3.92	-6445260.48	**13.48**
**s2h**	-2455.260649	-2454.849518	-6446286.83	49.74	-6445207.41	**66.55**
**s2NA**	-2455.272139	-2454.864468	-6446317.00	19.57	-6445246.66	**27.30**
**s2i**	-2455.260649	-2454.849518	-6446286.83	49.74	-6445207.41	**66.55**
**s2j**	-2455.278100	-2454.869732	-6446332.65	3.92	-6445260.48	**13.48**
**a2g**	-2455.279592	-2454.874867	-6446336.57	0.00	-6445273.96	**0.00**
**a2h**	n.a.	n.a.	n.a.	n.a.	n.a.	n.a.
**a2PA**	n.a.	n.a.	n.a.	n.a.	n.a.	n.a.
**a2i**	-2455.207427	-2454.801310	-6446147.10	189.47	-6445080.84	**193.12**
**a2j**	-2455.215796	-2454.811428	-6446169.07	167.50	-6445107.40	**166.56**
**s2k**	-2256.835696	-2256.413633	-5925322.12	91.31	-5924213.99	84.68
**s2l**	-2256.822386	-2256.399978	-5925287.17	126.25	-5924178.14	**120.53**
**s2PB**	-2256.861741	-2256.438763	-5925390.50	22.93	-5924279.97	**18.70**
**s2m**	-2256.858350	-2256.433379	-5925381.60	31.83	-5924265.84	**32.84**
**s2n**	-2256.870473	-2256.445887	-5925413.43	0.00	-5924298.68	**0.00**
**a2k**	-2256.836828	-2256.415327	-5925325.09	88.34	-5924218.44	**80.24**
**a2l**	-2256.823518	-2256.399301	-5925290.15	123.28	-5924176.36	**122.31**
**a2NB**	-2256.841262	-2256.415358	-5925336.73	76.69	-5924218.52	**80.15**
**a2m**	-2256.823518	-2256.399301	-5925290.15	123.28	-5924176.36	**122.31**

**a2n**	-2256.836828	-2256.415327	-5925325.09	88.34	-5924218.44	**80.24**
**sc3g**	-2256.849388	-2256.427990	-5925358.07	19.30	-5924251.69	**7.10**
**sc3h**	-2256.838573	-2256.412564	-5925329.67	47.70	-5924211.19	**47.60**
**sc3NA**	-2256.853256	-2256.426577	-5925368.22	9.15	-5924247.98	**10.81**
**sc3i**	-2256.838573	-2256.412564	-5925329.67	47.70	-5924211.19	**47.60**
**sc3j**	-2256.849388	-2256.427990	-5925358.07	19.30	-5924251.69	**7.10**
**st3g**	-2256.850232	-2256.428577	-5925360.28	17.09	-5924253.23	**5.56**
**st3h**	-2256.841508	-2256.415519	-5925337.38	39.99	-5924218.95	**39.84**
**st3NA**	-2256.856740	-2256.430693	-5925377.37	0.00	-5924258.78	**0.00**
**st3i**	-2256.841508	-2256.415519	-5925337.38	39.99	-5924218.95	**39.84**
**st3j**	-2256.850232	-2256.428577	-5925360.28	17.09	-5924253.23	**5.56**
**ac3g**	-2256.850198	-2256.426778	-5925360.19	17.18	-5924248.51	**10.28**
**ac3h**	-2256.837399	-2256.412466	-5925326.59	50.78	-5924210.93	**47.85**
**ac3PA**	-2256.841934	-2256.415971	-5925338.50	38.87	-5924220.13	**38.65**
**ac3i**	-2256.814521	-2256.391862	-5925266.53	110.85	-5924156.83	**101.95**
**ac3j**	-2256.821547	-2256.402205	-5925284.97	92.40	-5924183.99	**74.80**
**at3g**	-2256.851405	-2256.428217	-5925363.36	14.01	-5924252.28	**6.50**
**at3h**	-2256.836260	-2256.411376	-5925323.60	53.77	-5924208.07	**50.72**
**at3PA**	-2256.842348	-2256.416065	-5925339.58	37.79	-5924220.38	**38.41**
**at3i**	-2256.810557	-2256.387829	-5925256.12	121.25	-5924146.25	**112.54**
**at3j**	-2256.817500	-2256.395121	-5925274.35	103.03	-5924165.39	**93.39**
**s3k**	-2157.627650	-2157.197385	-5664851.39	62.43	-5663721.73	**55.44**
**s3l**	-2157.622366	-2157.190129	-5664837.52	76.30	-5663702.68	**74.49**
**s3PB**	-2157.651429	-2157.218502	-5664913.83	0.00	-5663777.18	**0.00**
**s3m**	-2157.642241	-2157.209862	-5664889.70	24.12	-5663754.49	**22.68**
**s3n**	-2157.650156	-2157.217637	-5664910.48	3.34	-5663774.91	**2.27**
**a3k**	-2157.629594	-2157.198377	-5664856.50	57.33	-5663724.34	**52.84**
**a3l**	-2157.621859	-2157.189034	-5664836.19	77.64	-5663699.81	**77.37**
**a3NB**	-2157.640468	-2157.206005	-5664885.05	28.78	-5663744.37	**32.81**
**a3m**	-2157.621859	-2157.189034	-5664836.19	77.64	-5663699.81	**77.37**
**a3n**	-2157.629594	-2157.198377	-5664856.50	57.33	-5663724.34	**52.84**
**4a**	-2653.653700	-2653.263990	-6967167.79	0.00	-6966144.61	**0.00**
**4b**	-2653.644966	-2653.250541	-6967144.86	22.93	-6966109.30	**35.31**
**4NA**	-2653.651385	-2653.258662	-6967161.71	6.08	-6966130.62	**13.99**
**s5a**	-3014.014501	-3013.625279	-7913295.07	0.54	-7912273.17	**8.57**
**s5b**	-3014.001344	-3013.612325	-7913260.53	35.08	-7912239.16	**42.58**
**s5PA**	-3014.005400	-3013.616550	-7913271.18	24.44	-7912250.25	**31.49**
**s5c**	-3013.996461	-3013.608234	-7913247.71	47.90	-7912228.42	**53.32**
**s5d**	-3014.007883	-3013.620550	-7913277.70	17.92	-7912260.75	**20.99**
**a5a**	-3014.014707	-3013.628544	-7913295.61	0.00	-7912281.74	**0.00**
**a5b**	-3014.002632	-3013.614315	-7913263.91	31.70	-7912244.38	**37.36**
**a5NA**	-3014.007052	-3013.618624	-7913275.52	20.10	-7912255.70	**26.04**
**a5c**	-3014.002632	-3013.614315	-7913263.91	31.70	-7912244.38	**37.36**
**a5d**	-3014.014707	-3013.628544	-7913295.61	0.00	-7912281.74	**0.00**
**sc5e**	-3374.368689	-3373.983471	-8859404.99	13.22	-8858393.60	**11.21**
**sc5f**	-3374.355026	-3373.968736	-8859369.12	46.93	-8858354.92	**49.89**
**sc5NB**	-3374.359399	-3373.972199	-8859380.60	37.61	-8858364.01	**40.80**
**sc5g**	-3374.355026	-3373.968736	-8859369.12	46.93	-8858354.92	**49.89**
**sc5h**	-3374.368689	-3373.983471	-8859404.99	13.22	-8858393.60	**11.21**
**st5e**	-3374.369031	-3373.981034	-8859405.89	12.32	-8858387.20	**17.60**
**st5f**	-3374.354438	-3373.966917	-8859367.58	48.48	-8858350.14	**54.67**

**st5NB**	-3374.358728	-3373.972549	-8859378.84	39.37	-8858364.93	**39.88**
**st5g**	-3374.354438	-3373.966917	-8859367.58	48.48	-8858350.14	**54.67**
**st5h**	-3374.369031	-3373.981034	-8859405.89	12.32	-8858387.20	**17.60**
**ac5e**	-3374.368393	-3373.983597	-8859404.22	13.99	-8858393.93	**10.87**
**ac5f**	-3374.355166	-3373.968679	-8859369.49	46.57	-8858354.77	**50.04**
**ac5NB**	-3374.359740	-3373.973217	-8859381.50	36.71	-8858366.68	**38.13**
**ac5g**	-3374.358353	-3373.971373	-8859377.86	38.20	-8858361.84	**42.97**
**ac5h**	-3374.372902	-3373.987204	-8859416.05	2.15	-8858403.40	**1.40**
**at5e**	-3374.369568	-3373.987739	-8859407.30	10.91	-8858404.81	**0.00**
**at5f**	-3374.354242	-3373.965129	-8859367.06	48.99	-8858345.45	**59.36**
**at5PB**	-3374.360599	-3373.973985	-8859383.75	34.46	-8858368.70	**36.11**
**at5g**	-3374.358353	-3373.972653	-8859377.86	38.20	-8858365.20	**39.61**
**at5h**	-3374.373723	-3373.986934	-8859418.21	0.00	-8858402.70	**2.11**

List of all computed structures in the pdb format. For visualization, copy the corresponding part of the text into a separate file named „title“.pdb.

1a.pdb

```
TITLE       1a
HETATM    1 Ru              0       -0.018   0.350   0.115                      Ru
HETATM    2  C              0       -0.585  -3.913   0.231                       C
HETATM    3  C              0        0.923  -3.775   0.385                       C
HETATM    4  H              0       -1.067  -4.416   1.091                       H
HETATM    5  H              0        1.298  -4.153   1.356                       H
HETATM    6  C              0       -0.021  -1.613   0.146                       C
HETATM    7  N              0        1.135  -2.335   0.288                       N
HETATM    8  N              0       -1.037  -2.527   0.134                       N
HETATM    9 Cl              0        0.909   0.625  -2.058                      Cl
HETATM   10 Cl              0        0.215   0.528   2.471                      Cl
HETATM   11  C              0       -1.804   0.744  -0.147                       C
HETATM   12  H              0       -2.594  -0.004  -0.313                       H
HETATM   13  C              0       -2.267   2.110  -0.150                       C
HETATM   14  C              0       -1.326   3.157   0.023                       C
HETATM   15  C              0       -3.628   2.436  -0.312                       C
HETATM   16  C              0       -1.745   4.488   0.032                       C
HETATM   17  C              0       -4.046   3.762  -0.300                       C
HETATM   18  H              0       -4.349   1.619  -0.443                       H
HETATM   19  C              0       -3.103   4.779  -0.129                       C
HETATM   20  H              0       -1.036   5.307   0.163                       H
HETATM   21  H              0       -5.106   4.010  -0.424                       H
HETATM   22  H              0       -3.423   5.827  -0.119                       H
HETATM   23  O              0       -0.062   2.700   0.167                       O
HETATM   24  C              0        1.114   3.533   0.363                       C
HETATM   25  H              0        1.894   2.756   0.436                       H
HETATM   26  C              0        2.477  -1.864   0.167                       C
HETATM   27  C              0        3.236  -1.582   1.320                       C
HETATM   28  C              0        3.082  -1.870  -1.112                       C
HETATM   29  C              0        4.583  -1.227   1.162                       C
HETATM   30  C              0        4.426  -1.510  -1.218                       C
HETATM   31  C              0        5.190  -1.175  -0.093                       C
```

```
HETATM   32  H           0       5.175  -0.997   2.059                     H
HETATM   33  H           0       4.893  -1.493  -2.212                     H
HETATM   34  C           0      -2.428  -2.283  -0.024                     C
HETATM   35  C           0      -3.235  -2.123   1.115                     C
HETATM   36  C           0      -2.968  -2.206  -1.321                     C
HETATM   37  C           0      -4.603  -1.889   0.935                     C
HETATM   38  C           0      -4.340  -1.975  -1.460                     C
HETATM   39  C           0      -5.170  -1.812  -0.343                     C
HETATM   40  H           0      -5.240  -1.755   1.819                     H
HETATM   41  H           0      -4.768  -1.905  -2.468                     H
HETATM   42  C           0       2.683  -1.740   2.704                     C
HETATM   43  H           0       2.810  -0.817   3.296                     H
HETATM   44  H           0       1.608  -1.969   2.711                     H
HETATM   45  H           0       3.219  -2.545   3.243                     H
HETATM   46  C           0       2.340  -2.336  -2.328                     C
HETATM   47  H           0       1.253  -2.181  -2.252                     H
HETATM   48  H           0       2.678  -1.796  -3.229                     H
HETATM   49  H           0       2.523  -3.414  -2.510                     H
HETATM   50  C           0       6.619  -0.751  -0.245                     C
HETATM   51  H           0       6.681   0.291  -0.615                     H
HETATM   52  H           0       7.168  -0.792   0.710                     H
HETATM   53  H           0       7.160  -1.375  -0.978                     H
HETATM   54  C           0      -2.622  -2.133   2.481                     C
HETATM   55  H           0      -3.386  -2.009   3.265                     H
HETATM   56  H           0      -2.077  -3.072   2.691                     H
HETATM   57  H           0      -1.882  -1.316   2.594                     H
HETATM   58  C           0      -2.064  -2.271  -2.514                     C
HETATM   59  H           0      -1.428  -3.176  -2.517                     H
HETATM   60  H           0      -2.637  -2.262  -3.456                     H
HETATM   61  H           0      -1.372  -1.406  -2.531                     H
HETATM   62  C           0      -6.632  -1.524  -0.517                     C
HETATM   63  H           0      -6.812  -0.445  -0.687                     H
HETATM   64  H           0      -7.056  -2.053  -1.389                     H
HETATM   65  H           0      -7.218  -1.808   0.372                     H
HETATM   66  C           0       1.073   4.266   1.687                     C
HETATM   67  H           0       2.079   4.665   1.905                     H
HETATM   68  H           0       0.373   5.118   1.707                     H
HETATM   69  H           0       0.802   3.569   2.497                     H
HETATM   70  C           0       1.415   4.369  -0.860                     C
HETATM   71  H           0       0.718   5.212  -1.003                     H
HETATM   72  H           0       2.431   4.789  -0.769                     H
HETATM   73  H           0       1.393   3.736  -1.764                     H
HETATM   74  H           0       1.487  -4.299  -0.409                     H
HETATM   75  H           0      -0.880  -4.477  -0.676                     H
HETATM   76  H           0       3.307   1.023  -0.310                     H
HETATM   77  C           0       4.089   1.792  -0.406                     C
HETATM   78  H           0       4.723   1.960   0.477                     H
HETATM   79  C           0       4.258   2.466  -1.543                     C
HETATM   80  H           0       5.040   3.230  -1.661                     H
HETATM   81  H           0       3.612   2.280  -2.413                     H
END
```

1b.pdb

```
TITLE       1b.pdb
HETATM    1 Ru           0      -0.525   0.346  -0.407                    Ru
HETATM    2  C           0      -0.272  -3.599   1.278                     C
HETATM    3  C           0      -1.758  -3.261   1.342                     C
HETATM    4  H           0       0.186  -3.712   2.280                     H
HETATM    5  H           0      -2.166  -3.294   2.370                     H
HETATM    6  C           0      -0.592  -1.454   0.358                     C
```

HETATM	7	N	0	-1.800	-1.905	0.816	N
HETATM	8	N	0	0.313	-2.442	0.593	N
HETATM	9	Cl	0	-0.526	1.361	1.787	Cl
HETATM	10	Cl	0	-0.944	-0.534	-2.658	Cl
HETATM	11	C	0	1.282	0.307	-0.816	C
HETATM	12	H	0	1.620	-0.606	-1.342	H
HETATM	13	C	0	2.377	1.237	-0.643	C
HETATM	14	C	0	2.245	2.617	-0.303	C
HETATM	15	C	0	3.680	0.713	-0.815	C
HETATM	16	C	0	3.402	3.387	-0.106	C
HETATM	17	C	0	4.817	1.475	-0.587	C
HETATM	18	H	0	3.769	-0.338	-1.116	H
HETATM	19	C	0	4.668	2.817	-0.228	C
HETATM	20	H	0	3.322	4.452	0.121	H
HETATM	21	H	0	5.813	1.035	-0.704	H
HETATM	22	H	0	5.552	3.445	-0.062	H
HETATM	23	O	0	0.999	3.098	-0.286	O
HETATM	24	C	0	0.595	4.308	0.392	C
HETATM	25	H	0	-0.491	4.125	0.495	H
HETATM	26	C	0	-3.033	-1.240	0.545	C
HETATM	27	C	0	-3.696	-1.487	-0.679	C
HETATM	28	C	0	-3.603	-0.391	1.517	C
HETATM	29	C	0	-4.849	-0.752	-0.970	C
HETATM	30	C	0	-4.758	0.325	1.179	C
HETATM	31	C	0	-5.373	0.183	-0.069	C
HETATM	32	H	0	-5.352	-0.918	-1.932	H
HETATM	33	H	0	-5.190	1.009	1.921	H
HETATM	34	C	0	1.726	-2.435	0.408	C
HETATM	35	C	0	2.256	-3.069	-0.733	C
HETATM	36	C	0	2.564	-1.895	1.399	C
HETATM	37	C	0	3.645	-3.162	-0.860	C
HETATM	38	C	0	3.950	-2.013	1.234	C
HETATM	39	C	0	4.506	-2.642	0.116	C
HETATM	40	H	0	4.066	-3.648	-1.750	H
HETATM	41	H	0	4.612	-1.591	2.001	H
HETATM	42	C	0	-3.251	-2.571	-1.612	C
HETATM	43	H	0	-3.414	-2.289	-2.664	H
HETATM	44	H	0	-2.182	-2.816	-1.513	H
HETATM	45	H	0	-3.835	-3.495	-1.424	H
HETATM	46	C	0	-3.071	-0.323	2.915	C
HETATM	47	H	0	-3.122	0.701	3.320	H
HETATM	48	H	0	-3.682	-0.967	3.580	H
HETATM	49	H	0	-2.023	-0.647	2.989	H
HETATM	50	C	0	-6.562	1.017	-0.437	C
HETATM	51	H	0	-6.251	1.937	-0.968	H
HETATM	52	H	0	-7.249	0.481	-1.114	H
HETATM	53	H	0	-7.133	1.339	0.449	H
HETATM	54	C	0	1.341	-3.570	-1.807	C
HETATM	55	H	0	1.904	-4.051	-2.624	H
HETATM	56	H	0	0.607	-4.308	-1.430	H
HETATM	57	H	0	0.744	-2.744	-2.243	H
HETATM	58	C	0	1.993	-1.168	2.575	C
HETATM	59	H	0	1.137	-1.698	3.031	H
HETATM	60	H	0	2.753	-1.011	3.358	H
HETATM	61	H	0	1.597	-0.176	2.280	H
HETATM	62	C	0	5.992	-2.705	-0.067	C
HETATM	63	H	0	6.530	-2.692	0.897	H
HETATM	64	H	0	6.306	-3.605	-0.623	H
HETATM	65	H	0	6.360	-1.835	-0.645	H
HETATM	66	C	0	0.798	5.529	-0.486	C
HETATM	67	H	0	0.297	6.402	-0.031	H
HETATM	68	H	0	1.861	5.791	-0.618	H

```
HETATM   69  H             0       0.363   5.379  -1.489                      H
HETATM   70  C             0       1.157   4.452   1.794                      C
HETATM   71  H             0       2.180   4.861   1.826                      H
HETATM   72  H             0       0.514   5.146   2.363                      H
HETATM   73  H             0       1.139   3.482   2.317                      H
HETATM   74  H             0      -2.379  -3.934   0.719                      H
HETATM   75  H             0      -0.058  -4.528   0.717                      H
HETATM   76  C             0      -1.559   2.624  -1.897                      C
HETATM   77  H             0      -1.075   3.602  -1.786                      H
HETATM   78  H             0      -1.385   2.081  -2.834                      H
HETATM   79  C             0      -2.335   2.114  -0.926                      C
HETATM   80  H             0      -2.879   1.163  -1.073                      H
HETATM   81  H             0      -2.528   2.647   0.015                      H
END
```

1c.pdb

```
TITLE      1c.pdb
HETATM    1 Ru             0      -0.571   0.369  -0.649                     Ru
HETATM    2  C             0      -0.670  -3.296   1.732                      C
HETATM    3  C             0      -2.126  -2.850   1.691                      C
HETATM    4  H             0      -0.232  -3.249   2.748                      H
HETATM    5  H             0      -2.565  -2.700   2.695                      H
HETATM    6  C             0      -0.816  -1.322   0.464                      C
HETATM    7  N             0      -2.051  -1.583   0.968                      N
HETATM    8  N             0       0.002  -2.324   0.859                      N
HETATM    9 Cl             0      -0.568   1.553   1.501                     Cl
HETATM   10 Cl             0      -1.033  -0.958  -2.658                     Cl
HETATM   11  C             0       1.259   0.205  -0.935                      C
HETATM   12  H             0       1.507  -0.791  -1.357                      H
HETATM   13  C             0       2.465   0.995  -0.791                      C
HETATM   14  C             0       2.571   2.359  -0.364                      C
HETATM   15  C             0       3.670   0.302  -1.072                      C
HETATM   16  C             0       3.846   2.931  -0.210                      C
HETATM   17  C             0       4.921   0.876  -0.909                      C
HETATM   18  H             0       3.586  -0.736  -1.419                      H
HETATM   19  C             0       5.001   2.200  -0.470                      C
HETATM   20  H             0       3.936   3.977   0.089                      H
HETATM   21  H             0       5.829   0.304  -1.131                      H
HETATM   22  H             0       5.978   2.683  -0.345                      H
HETATM   23  O             0       1.436   3.041  -0.205                      O
HETATM   24  C             0       1.282   4.194   0.655                      C
HETATM   25  H             0       0.191   4.174   0.822                      H
HETATM   26  C             0      -3.221  -0.839   0.621                      C
HETATM   27  C             0      -3.919  -1.158  -0.566                      C
HETATM   28  C             0      -3.712   0.148   1.500                      C
HETATM   29  C             0      -5.006  -0.359  -0.935                      C
HETATM   30  C             0      -4.801   0.924   1.087                      C
HETATM   31  C             0      -5.438   0.708  -0.139                      C
HETATM   32  H             0      -5.537  -0.589  -1.868                      H
HETATM   33  H             0      -5.169   1.713   1.757                      H
HETATM   34  C             0       1.402  -2.469   0.632                      C
HETATM   35  C             0       1.832  -3.320  -0.406                      C
HETATM   36  C             0       2.324  -1.843   1.489                      C
HETATM   37  C             0       3.203  -3.535  -0.569                      C
HETATM   38  C             0       3.689  -2.087   1.292                      C
HETATM   39  C             0       4.145  -2.928   0.273                      C
HETATM   40  H             0       3.545  -4.192  -1.379                      H
HETATM   41  H             0       4.415  -1.595   1.953                      H
HETATM   42  C             0      -3.613  -2.395  -1.357                      C
HETATM   43  H             0      -3.743  -2.226  -2.437                      H
```

```
HETATM   44  H             0       -2.584  -2.759  -1.217                      H
HETATM   45  H             0       -4.310  -3.206  -1.064                      H
HETATM   46  C             0       -3.178   0.316   2.890                      C
HETATM   47  H             0       -3.065   1.380   3.152                      H
HETATM   48  H             0       -3.886  -0.129   3.618                      H
HETATM   49  H             0       -2.194  -0.154   3.030                      H
HETATM   50  C             0       -6.562   1.591  -0.589                      C
HETATM   51  H             0       -6.195   2.399  -1.250                      H
HETATM   52  H             0       -7.320   1.034  -1.166                      H
HETATM   53  H             0       -7.069   2.081   0.260                      H
HETATM   54  C             0        0.836  -3.929  -1.344                      C
HETATM   55  H             0        1.323  -4.614  -2.057                      H
HETATM   56  H             0        0.050  -4.500  -0.815                      H
HETATM   57  H             0        0.307  -3.146  -1.924                      H
HETATM   58  C             0        1.866  -0.898   2.553                      C
HETATM   59  H             0        1.006  -1.288   3.128                      H
HETATM   60  H             0        2.676  -0.667   3.264                      H
HETATM   61  H             0        1.510   0.055   2.116                      H
HETATM   62  C             0        5.612  -3.137   0.048                      C
HETATM   63  H             0        6.211  -2.852   0.929                      H
HETATM   64  H             0        5.848  -4.187  -0.201                      H
HETATM   65  H             0        5.978  -2.527  -0.801                      H
HETATM   66  C             0        1.628   5.479  -0.072                      C
HETATM   67  H             0        1.279   6.344   0.518                      H
HETATM   68  H             0        2.711   5.612  -0.239                      H
HETATM   69  H             0        1.126   5.521  -1.054                      H
HETATM   70  C             0        1.933   4.025   2.013                      C
HETATM   71  H             0        3.027   4.168   2.011                      H
HETATM   72  H             0        1.508   4.771   2.706                      H
HETATM   73  H             0        1.703   3.025   2.418                      H
HETATM   74  H             0       -2.779  -3.560   1.146                      H
HETATM   75  H             0       -0.513  -4.323   1.357                      H
HETATM   76  C             0       -0.488   2.113  -2.355                      C
HETATM   77  H             0        0.490   2.605  -2.330                      H
HETATM   78  H             0       -0.723   1.516  -3.243                      H
HETATM   79  C             0       -1.427   2.367  -1.395                      C
HETATM   80  H             0       -2.464   2.011  -1.519                      H
HETATM   81  H             0       -1.258   3.087  -0.587                      H
END
```

1d.pdb

```
TITLE      1d.pdb
HETATM    1 Ru             0        0.537   0.190   1.003                     Ru
HETATM    2  C             0        0.815  -3.199  -1.687                      C
HETATM    3  C             0        2.276  -2.910  -1.377                      C
HETATM    4  H             0        0.559  -3.030  -2.753                      H
HETATM    5  H             0        2.922  -2.885  -2.272                      H
HETATM    6  C             0        0.945  -1.263  -0.381                      C
HETATM    7  N             0        2.207  -1.587  -0.751                      N
HETATM    8  N             0        0.111  -2.219  -0.851                      N
HETATM    9 Cl             0        1.129   2.039  -0.463                     Cl
HETATM   10 Cl             0        0.573  -1.709   2.585                     Cl
HETATM   11  C             0       -1.297   0.289   1.497                      C
HETATM   12  H             0       -1.521  -0.476   2.266                      H
HETATM   13  C             0       -2.534   0.902   1.039                      C
HETATM   14  C             0       -2.692   2.121   0.309                      C
HETATM   15  C             0       -3.696   0.149   1.325                      C
HETATM   16  C             0       -3.959   2.452  -0.203                      C
HETATM   17  C             0       -4.949   0.502   0.844                      C
HETATM   18  H             0       -3.579  -0.768   1.915                      H
```

HETATM	19	C	0	-5.068	1.649	0.058	C
HETATM	20	H	0	-4.096	3.366	-0.783	H
HETATM	21	H	0	-5.825	-0.114	1.072	H
HETATM	22	H	0	-6.045	1.947	-0.342	H
HETATM	23	O	0	-1.614	2.899	0.244	O
HETATM	24	C	0	-1.474	4.073	-0.585	C
HETATM	25	H	0	-0.413	4.312	-0.397	H
HETATM	26	C	0	3.351	-0.736	-0.643	C
HETATM	27	C	0	4.214	-0.816	0.466	C
HETATM	28	C	0	3.619	0.146	-1.709	C
HETATM	29	C	0	5.305	0.062	0.521	C
HETATM	30	C	0	4.726	0.990	-1.621	C
HETATM	31	C	0	5.568	0.976	-0.502	C
HETATM	32	H	0	5.974	0.015	1.390	H
HETATM	33	H	0	4.929	1.689	-2.443	H
HETATM	34	C	0	-1.314	-2.208	-0.825	C
HETATM	35	C	0	-1.998	-3.215	-0.116	C
HETATM	36	C	0	-2.017	-1.257	-1.592	C
HETATM	37	C	0	-3.395	-3.252	-0.191	C
HETATM	38	C	0	-3.411	-1.344	-1.651	C
HETATM	39	C	0	-4.117	-2.335	-0.963	C
HETATM	40	H	0	-3.933	-4.026	0.372	H
HETATM	41	H	0	-3.963	-0.602	-2.244	H
HETATM	42	C	0	4.014	-1.840	1.541	C
HETATM	43	H	0	4.635	-1.613	2.424	H
HETATM	44	H	0	2.964	-1.917	1.875	H
HETATM	45	H	0	4.313	-2.848	1.192	H
HETATM	46	C	0	2.713	0.198	-2.900	C
HETATM	47	H	0	2.494	-0.803	-3.315	H
HETATM	48	H	0	1.748	0.659	-2.620	H
HETATM	49	H	0	3.151	0.807	-3.708	H
HETATM	50	C	0	6.718	1.932	-0.402	C
HETATM	51	H	0	7.202	2.099	-1.380	H
HETATM	52	H	0	6.383	2.924	-0.044	H
HETATM	53	H	0	7.489	1.579	0.304	H
HETATM	54	C	0	-1.257	-4.237	0.692	C
HETATM	55	H	0	-1.893	-4.636	1.499	H
HETATM	56	H	0	-0.953	-5.106	0.076	H
HETATM	57	H	0	-0.351	-3.811	1.156	H
HETATM	58	C	0	-1.297	-0.166	-2.321	C
HETATM	59	H	0	-0.458	-0.551	-2.930	H
HETATM	60	H	0	-1.980	0.384	-2.991	H
HETATM	61	H	0	-0.850	0.570	-1.621	H
HETATM	62	C	0	-5.611	-2.417	-1.051	C
HETATM	63	H	0	-6.050	-1.483	-1.441	H
HETATM	64	H	0	-5.937	-3.235	-1.723	H
HETATM	65	H	0	-6.070	-2.623	-0.068	H
HETATM	66	C	0	-2.306	5.244	-0.094	C
HETATM	67	H	0	-1.920	6.175	-0.546	H
HETATM	68	H	0	-3.377	5.181	-0.347	H
HETATM	69	H	0	-2.220	5.351	1.001	H
HETATM	70	C	0	-1.602	3.749	-2.059	C
HETATM	71	H	0	-2.615	3.425	-2.355	H
HETATM	72	H	0	-1.354	4.645	-2.655	H
HETATM	73	H	0	-0.884	2.956	-2.326	H
HETATM	74	H	0	2.712	-3.641	-0.667	H
HETATM	75	H	0	0.509	-4.226	-1.427	H
HETATM	76	C	0	-0.022	1.642	2.723	C
HETATM	77	H	0	-0.407	2.571	2.287	H
HETATM	78	H	0	-0.596	1.211	3.554	H
HETATM	79	C	0	1.351	1.336	2.625	C
HETATM	80	H	0	1.801	0.640	3.346	H

```
HETATM   81  H            0        2.021   2.059   2.140                        H
END
```

1I.pdb

```
TITLE       1I.pdb
HETATM    1 Ru            0        0.524  -0.589  -0.370                       Ru
HETATM    2  C            0        1.367   3.390   0.979                        C
HETATM    3  C            0        2.770   2.791   1.078                        C
HETATM    4  H            0        0.995   3.789   1.940                        H
HETATM    5  H            0        3.158   2.762   2.115                        H
HETATM    6  C            0        1.295   1.151   0.310                        C
HETATM    7  N            0        2.587   1.426   0.573                        N
HETATM    8  N            0        0.552   2.246   0.559                        N
HETATM    9 Cl            0       -0.419  -1.121   1.787                       Cl
HETATM   10 Cl            0        1.739  -0.055  -2.451                       Cl
HETATM   11  C            0       -0.880  -1.270  -1.673                        C
HETATM   12  H            0       -0.518  -0.990  -2.674                        H
HETATM   13  C            0       -2.333  -1.049  -1.595                        C
HETATM   14  C            0       -3.250  -1.595  -0.651                        C
HETATM   15  C            0       -2.858  -0.215  -2.606                        C
HETATM   16  C            0       -4.615  -1.280  -0.757                        C
HETATM   17  C            0       -4.207   0.102  -2.695                        C
HETATM   18  H            0       -2.154   0.198  -3.341                        H
HETATM   19  C            0       -5.089  -0.437  -1.758                        C
HETATM   20  H            0       -5.327  -1.730  -0.062                        H
HETATM   21  H            0       -4.570   0.760  -3.493                        H
HETATM   22  H            0       -6.162  -0.217  -1.811                        H
HETATM   23  O            0       -2.760  -2.454   0.255                        O
HETATM   24  C            0       -3.359  -2.697   1.547                        C
HETATM   25  H            0       -2.481  -3.058   2.110                        H
HETATM   26  C            0        3.673   0.509   0.429                        C
HETATM   27  C            0        4.545   0.627  -0.669                        C
HETATM   28  C            0        3.871  -0.479   1.412                        C
HETATM   29  C            0        5.569  -0.315  -0.809                        C
HETATM   30  C            0        4.906  -1.403   1.231                        C
HETATM   31  C            0        5.751  -1.348   0.118                        C
HETATM   32  H            0        6.238  -0.243  -1.677                        H
HETATM   33  H            0        5.060  -2.180   1.991                        H
HETATM   34  C            0       -0.875   2.292   0.509                        C
HETATM   35  C            0       -1.516   2.501  -0.725                        C
HETATM   36  C            0       -1.611   2.166   1.704                        C
HETATM   37  C            0       -2.914   2.565  -0.746                        C
HETATM   38  C            0       -3.005   2.217   1.631                        C
HETATM   39  C            0       -3.673   2.409   0.416                        C
HETATM   40  H            0       -3.421   2.730  -1.705                        H
HETATM   41  H            0       -3.587   2.092   2.555                        H
HETATM   42  C            0        4.419   1.746  -1.658                        C
HETATM   43  H            0        4.714   1.416  -2.667                        H
HETATM   44  H            0        3.385   2.119  -1.735                        H
HETATM   45  H            0        5.079   2.596  -1.389                        H
HETATM   46  C            0        3.014  -0.536   2.638                        C
HETATM   47  H            0        2.880   0.462   3.096                        H
HETATM   48  H            0        1.994  -0.912   2.428                        H
HETATM   49  H            0        3.458  -1.197   3.400                        H
HETATM   50  C            0        6.822  -2.376  -0.089                        C
HETATM   51  H            0        6.491  -3.159  -0.798                        H
HETATM   52  H            0        7.741  -1.937  -0.515                        H
HETATM   53  H            0        7.091  -2.888   0.851                        H
HETATM   54  C            0       -0.725   2.655  -1.988                        C
HETATM   55  H            0       -1.375   2.944  -2.831                        H
```

```
HETATM   56  H            0        0.065   3.424  -1.894                      H
HETATM   57  H            0       -0.196   1.723  -2.273                      H
HETATM   58  C            0       -0.931   2.004   3.029                      C
HETATM   59  H            0       -0.761   2.982   3.522                      H
HETATM   60  H            0       -1.548   1.401   3.717                      H
HETATM   61  H            0        0.040   1.491   2.940                      H
HETATM   62  C            0       -5.171   2.447   0.377                      C
HETATM   63  H            0       -5.605   1.500   0.748                      H
HETATM   64  H            0       -5.579   3.250   1.018                      H
HETATM   65  H            0       -5.553   2.606  -0.646                      H
HETATM   66  C            0       -4.376  -3.821   1.483                      C
HETATM   67  H            0       -4.656  -4.129   2.506                      H
HETATM   68  H            0       -5.306  -3.537   0.959                      H
HETATM   69  H            0       -3.955  -4.703   0.971                      H
HETATM   70  C            0       -3.847  -1.441   2.243                      C
HETATM   71  H            0       -4.835  -1.090   1.900                      H
HETATM   72  H            0       -3.931  -1.644   3.326                      H
HETATM   73  H            0       -3.114  -0.627   2.113                      H
HETATM   74  H            0        3.514   3.327   0.462                      H
HETATM   75  H            0        1.294   4.203   0.232                      H
HETATM   76  C            0       -0.146  -2.577  -1.200                      C
HETATM   77  H            0       -0.802  -3.167  -0.549                      H
HETATM   78  H            0        0.067  -3.084  -2.158                      H
HETATM   79  C            0        1.267  -2.398  -0.547                      C
HETATM   80  H            0        2.111  -2.518  -1.245                      H
HETATM   81  H            0        1.373  -2.950   0.402                      H
END
```

1e.pdb

```
TITLE      1e.pdb
HETATM    1 Ru            0       -0.526  -0.716   0.318                     Ru
HETATM    2  C            0       -1.222   3.427  -0.759                      C
HETATM    3  C            0       -2.644   2.886  -0.875                      C
HETATM    4  H            0       -0.848   3.874  -1.698                      H
HETATM    5  H            0       -3.048   2.946  -1.904                      H
HETATM    6  C            0       -1.214   1.140  -0.237                      C
HETATM    7  N            0       -2.499   1.484  -0.470                      N
HETATM    8  N            0       -0.444   2.230  -0.426                      N
HETATM    9 Cl            0        0.443  -0.983  -1.905                     Cl
HETATM   10 Cl            0       -1.596  -0.297   2.496                     Cl
HETATM   11  C            0        0.756  -1.997   1.562                      C
HETATM   12  H            0        0.287  -1.947   2.554                      H
HETATM   13  C            0        2.107  -1.419   1.572                      C
HETATM   14  C            0        3.138  -1.677   0.625                      C
HETATM   15  C            0        2.409  -0.611   2.688                      C
HETATM   16  C            0        4.416  -1.137   0.847                      C
HETATM   17  C            0        3.674  -0.075   2.891                      C
HETATM   18  H            0        1.603  -0.417   3.407                      H
HETATM   19  C            0        4.679  -0.347   1.963                      C
HETATM   20  H            0        5.229  -1.368   0.155                      H
HETATM   21  H            0        3.877   0.548   3.770                      H
HETATM   22  H            0        5.691   0.053   2.107                      H
HETATM   23  O            0        2.831  -2.506  -0.382                      O
HETATM   24  C            0        3.539  -2.570  -1.637                      C
HETATM   25  H            0        2.754  -2.990  -2.290                      H
HETATM   26  C            0       -3.628   0.615  -0.385                      C
HETATM   27  C            0       -4.469   0.672   0.740                      C
HETATM   28  C            0       -3.901  -0.260  -1.456                      C
HETATM   29  C            0       -5.549  -0.217   0.808                      C
HETATM   30  C            0       -4.987  -1.134  -1.344                      C
```

```
HETATM   31  C             0       -5.811  -1.137  -0.212                      C
HETATM   32  H             0       -6.197  -0.192   1.694                      H
HETATM   33  H             0       -5.199  -1.824  -2.172                      H
HETATM   34  C             0        0.983   2.265  -0.381                      C
HETATM   35  C             0        1.632   2.392   0.862                      C
HETATM   36  C             0        1.711   2.256  -1.586                      C
HETATM   37  C             0        3.028   2.490   0.877                      C
HETATM   38  C             0        3.106   2.339  -1.521                      C
HETATM   39  C             0        3.780   2.448  -0.301                      C
HETATM   40  H             0        3.538   2.602   1.841                      H
HETATM   41  H             0        3.681   2.311  -2.457                      H
HETATM   42  C             0       -4.261   1.679   1.830                      C
HETATM   43  H             0       -4.543   1.264   2.811                      H
HETATM   44  H             0       -3.211   2.000   1.908                      H
HETATM   45  H             0       -4.889   2.577   1.665                      H
HETATM   46  C             0       -3.060  -0.239  -2.695                      C
HETATM   47  H             0       -3.057   0.760  -3.170                      H
HETATM   48  H             0       -1.999  -0.489  -2.497                      H
HETATM   49  H             0       -3.437  -0.956  -3.443                      H
HETATM   50  C             0       -6.944  -2.111  -0.091                      C
HETATM   51  H             0       -6.633  -3.023   0.455                      H
HETATM   52  H             0       -7.795  -1.688   0.468                      H
HETATM   53  H             0       -7.312  -2.441  -1.077                      H
HETATM   54  C             0        0.850   2.455   2.138                      C
HETATM   55  H             0        1.520   2.566   3.007                      H
HETATM   56  H             0        0.145   3.309   2.149                      H
HETATM   57  H             0        0.231   1.552   2.306                      H
HETATM   58  C             0        1.030   2.195  -2.920                      C
HETATM   59  H             0        0.930   3.203  -3.370                      H
HETATM   60  H             0        1.612   1.582  -3.630                      H
HETATM   61  H             0        0.027   1.742  -2.859                      H
HETATM   62  C             0        5.277   2.492  -0.258                      C
HETATM   63  H             0        5.707   1.478  -0.372                      H
HETATM   64  H             0        5.699   3.106  -1.074                      H
HETATM   65  H             0        5.652   2.892   0.700                      H
HETATM   66  C             0        4.685  -3.561  -1.565                      C
HETATM   67  H             0        5.098  -3.733  -2.575                      H
HETATM   68  H             0        5.513  -3.209  -0.925                      H
HETATM   69  H             0        4.343  -4.533  -1.171                      H
HETATM   70  C             0        3.918  -1.217  -2.213                      C
HETATM   71  H             0        4.874  -0.819  -1.831                      H
HETATM   72  H             0        4.025  -1.315  -3.308                      H
HETATM   73  H             0        3.118  -0.482  -2.024                      H
HETATM   74  H             0       -3.360   3.401  -0.210                      H
HETATM   75  H             0       -1.108   4.187   0.037                      H
HETATM   76  C             0        0.127  -2.901   0.666                      C
HETATM   77  H             0        0.614  -3.222  -0.260                      H
HETATM   78  H             0       -0.569  -3.618   1.124                      H
HETATM   79  C             0       -1.749  -2.053  -0.013                      C
HETATM   80  H             0       -2.518  -2.347   0.731                      H
HETATM   81  H             0       -1.796  -2.578  -0.990                      H
END
```

1f.pdb

```
TITLE       1f.pdb
HETATM    1 Ru             0       -0.520  -0.791   0.393                     Ru
HETATM    2  C             0       -1.219   3.293  -0.766                      C
HETATM    3  C             0       -2.665   2.836  -0.622                      C
HETATM    4  H             0       -0.959   3.582  -1.803                      H
HETATM    5  H             0       -3.269   3.004  -1.532                      H
```

HETATM	6	C	0	-1.241	1.013	-0.191	C
HETATM	7	N	0	-2.531	1.401	-0.361	N
HETATM	8	N	0	-0.460	2.103	-0.378	N
HETATM	9	Cl	0	0.228	-1.322	-1.869	Cl
HETATM	10	Cl	0	-1.218	-0.123	2.657	Cl
HETATM	11	C	0	0.978	-2.156	1.706	C
HETATM	12	C	0	2.227	-1.387	1.656	C
HETATM	13	C	0	3.246	-1.555	0.672	C
HETATM	14	C	0	2.469	-0.508	2.731	C
HETATM	15	C	0	4.463	-0.871	0.831	C
HETATM	16	C	0	3.670	0.176	2.867	C
HETATM	17	H	0	1.668	-0.377	3.471	H
HETATM	18	C	0	4.669	-0.019	1.913	C
HETATM	19	H	0	5.272	-1.023	0.114	H
HETATM	20	H	0	3.828	0.856	3.712	H
HETATM	21	H	0	5.632	0.500	2.006	H
HETATM	22	O	0	2.988	-2.435	-0.304	O
HETATM	23	C	0	3.694	-2.517	-1.557	C
HETATM	24	H	0	2.982	-3.121	-2.148	H
HETATM	25	C	0	-3.693	0.578	-0.330	C
HETATM	26	C	0	-4.555	0.613	0.784	C
HETATM	27	C	0	-4.004	-0.197	-1.467	C
HETATM	28	C	0	-5.695	-0.199	0.765	C
HETATM	29	C	0	-5.152	-0.994	-1.440	C
HETATM	30	C	0	-6.001	-1.018	-0.327	C
HETATM	31	H	0	-6.361	-0.191	1.638	H
HETATM	32	H	0	-5.394	-1.605	-2.320	H
HETATM	33	C	0	0.966	2.129	-0.461	C
HETATM	34	C	0	1.716	2.445	0.691	C
HETATM	35	C	0	1.585	1.995	-1.719	C
HETATM	36	C	0	3.101	2.585	0.564	C
HETATM	37	C	0	2.977	2.121	-1.791	C
HETATM	38	C	0	3.749	2.407	-0.663	C
HETATM	39	H	0	3.690	2.837	1.455	H
HETATM	40	H	0	3.468	1.995	-2.766	H
HETATM	41	C	0	-4.284	1.505	1.955	C
HETATM	42	H	0	-4.711	1.086	2.881	H
HETATM	43	H	0	-3.203	1.642	2.126	H
HETATM	44	H	0	-4.745	2.504	1.816	H
HETATM	45	C	0	-3.126	-0.150	-2.678	C
HETATM	46	H	0	-2.977	0.888	-3.034	H
HETATM	47	H	0	-2.115	-0.556	-2.479	H
HETATM	48	H	0	-3.562	-0.728	-3.509	H
HETATM	49	C	0	-7.205	-1.911	-0.300	C
HETATM	50	H	0	-6.985	-2.866	0.214	H
HETATM	51	H	0	-8.049	-1.450	0.241	H
HETATM	52	H	0	-7.550	-2.169	-1.316	H
HETATM	53	C	0	1.046	2.654	2.013	C
HETATM	54	H	0	1.776	2.954	2.784	H
HETATM	55	H	0	0.268	3.440	1.964	H
HETATM	56	H	0	0.528	1.743	2.369	H
HETATM	57	C	0	0.796	1.792	-2.977	C
HETATM	58	H	0	0.663	2.750	-3.520	H
HETATM	59	H	0	1.319	1.105	-3.663	H
HETATM	60	H	0	-0.198	1.357	-2.793	H
HETATM	61	C	0	5.242	2.488	-0.756	C
HETATM	62	H	0	5.703	1.496	-0.581	H
HETATM	63	H	0	5.581	2.824	-1.752	H
HETATM	64	H	0	5.669	3.171	-0.000	H
HETATM	65	C	0	4.984	-3.306	-1.429	C
HETATM	66	H	0	5.372	-3.548	-2.434	H
HETATM	67	H	0	5.779	-2.759	-0.894	H

```
HETATM   68  H             0       4.811  -4.258  -0.899                     H
HETATM   69  C             0       3.828  -1.181  -2.262                     C
HETATM   70  H             0       4.607  -0.526  -1.838                     H
HETATM   71  H             0       4.087  -1.357  -3.321                     H
HETATM   72  H             0       2.862  -0.649  -2.231                     H
HETATM   73  H             0      -3.188   3.328   0.220                     H
HETATM   74  H             0      -0.962   4.145  -0.112                     H
HETATM   75  C             0       0.429  -3.035   0.805                     C
HETATM   76  H             0       0.870  -3.268  -0.165                     H
HETATM   77  C             0      -2.069  -1.725   0.247                     C
HETATM   78  H             0      -2.844  -1.736   1.043                     H
HETATM   79  H             0      -2.259  -2.391  -0.625                     H
HETATM   80  H             0       0.479  -2.084   2.681                     H
HETATM   81  H             0      -0.418  -3.650   1.130                     H
END
```

s2a.pdb

```
TITLE      s2a.pdb
HETATM    1 Ru             0       0.125   0.317  -0.098                    Ru
HETATM    2  C             0       0.794  -3.797   0.911                     C
HETATM    3  C             0      -0.671  -3.814   0.528                     C
HETATM    4  H             0       0.952  -3.917   2.001                     H
HETATM    5  H             0      -1.327  -4.234   1.310                     H
HETATM    6  C             0       0.170  -1.628   0.192                     C
HETATM    7  N             0      -0.962  -2.400   0.312                     N
HETATM    8  N             0       1.218  -2.465   0.482                     N
HETATM    9 Cl             0      -0.358   0.715   2.199                    Cl
HETATM   10 Cl             0      -0.617   0.403  -2.344                    Cl
HETATM   11  C             0       1.921   0.678  -0.332                     C
HETATM   12  H             0       2.704  -0.089  -0.409                     H
HETATM   13  C             0       2.406   2.024  -0.517                     C
HETATM   14  C             0       1.498   3.109  -0.455                     C
HETATM   15  C             0       3.769   2.287  -0.763                     C
HETATM   16  C             0       1.950   4.417  -0.638                     C
HETATM   17  C             0       4.220   3.590  -0.938                     C
HETATM   18  H             0       4.464   1.439  -0.809                     H
HETATM   19  C             0       3.308   4.648  -0.875                     C
HETATM   20  H             0       1.257   5.262  -0.614                     H
HETATM   21  H             0       5.280   3.789  -1.128                     H
HETATM   22  H             0       3.652   5.678  -1.021                     H
HETATM   23  O             0       0.219   2.720  -0.246                     O
HETATM   24  C             0      -0.831   3.600   0.246                     C
HETATM   25  H             0      -1.581   2.849   0.531                     H
HETATM   26  C             0      -2.294  -2.084  -0.097                     C
HETATM   27  C             0      -2.658  -2.219  -1.452                     C
HETATM   28  C             0      -3.271  -1.846   0.893                     C
HETATM   29  C             0      -3.995  -2.005  -1.809                     C
HETATM   30  C             0      -4.599  -1.669   0.493                     C
HETATM   31  C             0      -4.976  -1.720  -0.854                     C
HETATM   32  H             0      -4.277  -2.081  -2.867                     H
HETATM   33  H             0      -5.358  -1.464   1.259                     H
HETATM   34  C             0       2.615  -2.212   0.397                     C
HETATM   35  C             0       3.251  -2.392  -0.846                     C
HETATM   36  C             0       3.340  -1.826   1.537                     C
HETATM   37  C             0       4.629  -2.174  -0.932                     C
HETATM   38  C             0       4.717  -1.614   1.409                     C
HETATM   39  C             0       5.377  -1.780   0.185                     C
HETATM   40  H             0       5.130  -2.306  -1.900                     H
HETATM   41  H             0       5.290  -1.303   2.293                     H
HETATM   42  C             0      -1.683  -2.678  -2.493                     C
```

```
HETATM   43  H             0       -1.873  -2.187  -3.462                      H
HETATM   44  H             0       -0.638  -2.462  -2.225                      H
HETATM   45  H             0       -1.778  -3.770  -2.658                      H
HETATM   46  C             0       -2.906  -1.828   2.345                      C
HETATM   47  H             0       -3.758  -1.502   2.964                      H
HETATM   48  H             0       -2.611  -2.834   2.701                      H
HETATM   49  H             0       -2.054  -1.156   2.549                      H
HETATM   50  C             0       -6.390  -1.443  -1.263                      C
HETATM   51  H             0       -6.556  -0.357  -1.403                      H
HETATM   52  H             0       -6.648  -1.930  -2.219                      H
HETATM   53  H             0       -7.115  -1.776  -0.500                      H
HETATM   54  C             0        2.442  -2.729  -2.060                      C
HETATM   55  H             0        3.086  -2.940  -2.930                      H
HETATM   56  H             0        1.787  -3.606  -1.903                      H
HETATM   57  H             0        1.772  -1.890  -2.335                      H
HETATM   58  C             0        2.643  -1.597   2.842                      C
HETATM   59  H             0        2.207  -2.527   3.253                      H
HETATM   60  H             0        3.338  -1.201   3.601                      H
HETATM   61  H             0        1.808  -0.879   2.738                      H
HETATM   62  C             0        6.846  -1.507   0.067                      C
HETATM   63  H             0        7.392  -1.777   0.986                      H
HETATM   64  H             0        7.303  -2.055  -0.775                      H
HETATM   65  H             0        7.042  -0.432  -0.110                      H
HETATM   66  C             0       -1.405   4.451  -0.868                      C
HETATM   67  H             0       -2.389   4.842  -0.554                      H
HETATM   68  H             0       -0.776   5.318  -1.133                      H
HETATM   69  H             0       -1.554   3.845  -1.778                      H
HETATM   70  C             0       -0.434   4.339   1.504                      C
HETATM   71  H             0        0.265   5.175   1.334                      H
HETATM   72  H             0       -1.347   4.762   1.961                      H
HETATM   73  H             0        0.012   3.640   2.230                      H
HETATM   74  H             0       -0.861  -4.384  -0.406                      H
HETATM   75  H             0        1.391  -4.575   0.403                      H
HETATM   76  C             0       -3.491   1.467  -0.440                      C
HETATM   77  H             0       -3.285   2.208  -1.216                      H
HETATM   78  H             0       -3.332   0.408  -0.658                      H
HETATM   79  C             0       -3.889   1.871   0.758                      C
HETATM   80  F             0       -4.154   1.105   1.793                      F
HETATM   81  F             0       -4.053   3.141   1.100                      F
END
```

s2b.pdb

```
TITLE       s2b.pdb
HETATM    1 Ru             0       -0.286   0.179  -0.354                     Ru
HETATM    2  C             0        0.022  -3.747   1.298                      C
HETATM    3  C             0       -1.478  -3.584   1.115                      C
HETATM    4  H             0        0.327  -3.740   2.364                      H
HETATM    5  H             0       -2.050  -3.726   2.049                      H
HETATM    6  C             0       -0.392  -1.647   0.309                      C
HETATM    7  N             0       -1.593  -2.208   0.647                      N
HETATM    8  N             0        0.568  -2.570   0.620                      N
HETATM    9 Cl             0       -0.730   1.048   1.823                     Cl
HETATM   10 Cl             0       -0.785  -0.422  -2.631                     Cl
HETATM   11  C             0        1.527   0.211  -0.723                      C
HETATM   12  H             0        1.956  -0.730  -1.109                      H
HETATM   13  C             0        2.522   1.260  -0.736                      C
HETATM   14  C             0        2.286   2.603  -0.324                      C
HETATM   15  C             0        3.835   0.901  -1.126                      C
HETATM   16  C             0        3.355   3.511  -0.288                      C
HETATM   17  C             0        4.885   1.806  -1.089                      C
```

HETATM	18	H	0	4.007	-0.134	-1.448	H
HETATM	19	C	0	4.637	3.115	-0.663	C
HETATM	20	H	0	3.183	4.546	0.014	H
HETATM	21	H	0	5.893	1.502	-1.393	H
HETATM	22	H	0	5.452	3.848	-0.633	H
HETATM	23	O	0	1.016	2.918	-0.037	O
HETATM	24	C	0	0.649	3.927	0.944	C
HETATM	25	H	0	-0.374	3.604	1.190	H
HETATM	26	C	0	-2.878	-1.637	0.403	C
HETATM	27	C	0	-3.498	-1.827	-0.851	C
HETATM	28	C	0	-3.561	-0.990	1.454	C
HETATM	29	C	0	-4.739	-1.224	-1.078	C
HETATM	30	C	0	-4.804	-0.410	1.182	C
HETATM	31	C	0	-5.389	-0.485	-0.085	C
HETATM	32	H	0	-5.212	-1.343	-2.062	H
HETATM	33	H	0	-5.327	0.120	1.990	H
HETATM	34	C	0	1.981	-2.488	0.473	C
HETATM	35	C	0	2.564	-3.105	-0.651	C
HETATM	36	C	0	2.769	-1.843	1.441	C
HETATM	37	C	0	3.954	-3.069	-0.790	C
HETATM	38	C	0	4.158	-1.828	1.263	C
HETATM	39	C	0	4.766	-2.430	0.157	C
HETATM	40	H	0	4.414	-3.537	-1.671	H
HETATM	41	H	0	4.780	-1.316	2.009	H
HETATM	42	C	0	-2.922	-2.741	-1.890	C
HETATM	43	H	0	-3.092	-2.353	-2.908	H
HETATM	44	H	0	-1.837	-2.888	-1.783	H
HETATM	45	H	0	-3.411	-3.735	-1.833	H
HETATM	46	C	0	-3.041	-0.987	2.859	C
HETATM	47	H	0	-3.161	0.003	3.330	H
HETATM	48	H	0	-3.608	-1.712	3.477	H
HETATM	49	H	0	-1.973	-1.242	2.922	H
HETATM	50	C	0	-6.667	0.237	-0.381	C
HETATM	51	H	0	-6.460	1.269	-0.726	H
HETATM	52	H	0	-7.247	-0.254	-1.181	H
HETATM	53	H	0	-7.313	0.325	0.510	H
HETATM	54	C	0	1.691	-3.690	-1.719	C
HETATM	55	H	0	2.290	-4.159	-2.517	H
HETATM	56	H	0	0.993	-4.455	-1.331	H
HETATM	57	H	0	1.059	-2.908	-2.185	H
HETATM	58	C	0	2.144	-1.118	2.590	C
HETATM	59	H	0	1.219	-1.601	2.951	H
HETATM	60	H	0	2.844	-1.023	3.437	H
HETATM	61	H	0	1.839	-0.097	2.290	H
HETATM	62	C	0	6.249	-2.341	-0.042	C
HETATM	63	H	0	6.793	-2.290	0.916	H
HETATM	64	H	0	6.644	-3.196	-0.616	H
HETATM	65	H	0	6.518	-1.427	-0.607	H
HETATM	66	C	0	0.579	5.318	0.344	C
HETATM	67	H	0	0.085	5.996	1.062	H
HETATM	68	H	0	1.566	5.752	0.111	H
HETATM	69	H	0	-0.025	5.324	-0.578	H
HETATM	70	C	0	1.468	3.824	2.214	C
HETATM	71	H	0	2.497	4.209	2.116	H
HETATM	72	H	0	0.972	4.410	3.008	H
HETATM	73	H	0	1.509	2.776	2.554	H
HETATM	74	H	0	-1.898	-4.279	0.359	H
HETATM	75	H	0	0.422	-4.674	0.850	H
HETATM	76	C	0	-1.458	2.840	-1.885	C
HETATM	77	H	0	-0.748	3.663	-1.976	H
HETATM	78	H	0	-1.421	2.001	-2.585	H
HETATM	79	C	0	-2.361	2.858	-0.913	C

```
HETATM   80  F            0       -3.257   1.921  -0.692                          F
HETATM   81  F            0       -2.503   3.827  -0.028                          F
END
```

s2c.pdb

```
TITLE       s2c.pdb
HETATM    1 Ru            0       -0.445   0.163  -0.477                         Ru
HETATM    2  C            0       -0.248  -3.614   1.568                          C
HETATM    3  C            0       -1.743  -3.336   1.483                          C
HETATM    4  H            0        0.139  -3.565   2.605                          H
HETATM    5  H            0       -2.244  -3.339   2.468                          H
HETATM    6  C            0       -0.571  -1.579   0.428                          C
HETATM    7  N            0       -1.785  -2.006   0.888                          N
HETATM    8  N            0        0.338  -2.534   0.769                          N
HETATM    9 Cl            0       -0.768   1.276   1.631                         Cl
HETATM   10 Cl            0       -0.849  -0.857  -2.656                         Cl
HETATM   11  C            0        1.382   0.145  -0.807                          C
HETATM   12  H            0        1.730  -0.823  -1.212                          H
HETATM   13  C            0        2.486   1.086  -0.792                          C
HETATM   14  C            0        2.467   2.426  -0.289                          C
HETATM   15  C            0        3.725   0.587  -1.271                          C
HETATM   16  C            0        3.658   3.171  -0.266                          C
HETATM   17  C            0        4.892   1.335  -1.250                          C
HETATM   18  H            0        3.742  -0.441  -1.657                          H
HETATM   19  C            0        4.852   2.634  -0.737                          C
HETATM   20  H            0        3.646   4.199   0.101                          H
HETATM   21  H            0        5.828   0.916  -1.634                          H
HETATM   22  H            0        5.760   3.248  -0.714                          H
HETATM   23  O            0        1.284   2.917   0.089                          O
HETATM   24  C            0        1.111   3.958   1.081                          C
HETATM   25  H            0        0.063   3.784   1.370                          H
HETATM   26  C            0       -3.022  -1.357   0.589                          C
HETATM   27  C            0       -3.664  -1.615  -0.641                          C
HETATM   28  C            0       -3.619  -0.514   1.551                          C
HETATM   29  C            0       -4.811  -0.881  -0.961                          C
HETATM   30  C            0       -4.767   0.196   1.188                          C
HETATM   31  C            0       -5.351   0.057  -0.075                          C
HETATM   32  H            0       -5.295  -1.053  -1.931                          H
HETATM   33  H            0       -5.216   0.881   1.920                          H
HETATM   34  C            0        1.747  -2.526   0.563                          C
HETATM   35  C            0        2.269  -3.250  -0.525                          C
HETATM   36  C            0        2.590  -1.846   1.461                          C
HETATM   37  C            0        3.656  -3.292  -0.695                          C
HETATM   38  C            0        3.972  -1.910   1.252                          C
HETATM   39  C            0        4.521  -2.628   0.183                          C
HETATM   40  H            0        4.070  -3.850  -1.545                          H
HETATM   41  H            0        4.637  -1.374   1.941                          H
HETATM   42  C            0       -3.221  -2.725  -1.546                          C
HETATM   43  H            0       -3.344  -2.456  -2.607                          H
HETATM   44  H            0       -2.163  -2.999  -1.411                          H
HETATM   45  H            0       -3.837  -3.628  -1.360                          H
HETATM   46  C            0       -3.118  -0.441   2.960                          C
HETATM   47  H            0       -3.188   0.584   3.361                          H
HETATM   48  H            0       -3.734  -1.090   3.614                          H
HETATM   49  H            0       -2.068  -0.754   3.055                          H
HETATM   50  C            0       -6.518   0.906  -0.477                          C
HETATM   51  H            0       -6.173   1.844  -0.953                          H
HETATM   52  H            0       -7.168   0.400  -1.210                          H
HETATM   53  H            0       -7.137   1.200   0.388                          H
HETATM   54  C            0        1.345  -3.883  -1.519                          C
```

```
HETATM   55  H             0        1.904  -4.429  -2.297                          H
HETATM   56  H             0        0.639  -4.597  -1.053                          H
HETATM   57  H             0        0.720  -3.117  -2.022                          H
HETATM   58  C             0        2.015  -1.028   2.574                          C
HETATM   59  H             0        1.282  -1.592   3.180                          H
HETATM   60  H             0        2.803  -0.662   3.253                          H
HETATM   61  H             0        1.462  -0.151   2.184                          H
HETATM   62  C             0        6.002  -2.640  -0.046                          C
HETATM   63  H             0        6.568  -2.587   0.900                          H
HETATM   64  H             0        6.330  -3.541  -0.591                          H
HETATM   65  H             0        6.319  -1.769  -0.651                          H
HETATM   66  C             0        1.205   5.342   0.468                          C
HETATM   67  H             0        0.822   6.089   1.185                          H
HETATM   68  H             0        2.231   5.644   0.196                          H
HETATM   69  H             0        0.577   5.403  -0.438                          H
HETATM   70  C             0        1.950   3.732   2.321                          C
HETATM   71  H             0        3.027   3.928   2.184                          H
HETATM   72  H             0        1.593   4.401   3.123                          H
HETATM   73  H             0        1.823   2.694   2.675                          H
HETATM   74  H             0       -2.278  -4.060   0.835                          H
HETATM   75  H             0        0.040  -4.599   1.159                          H
HETATM   76  C             0       -0.778   2.357  -2.080                          C
HETATM   77  H             0        0.155   2.919  -2.038                          H
HETATM   78  H             0       -0.946   1.610  -2.860                          H
HETATM   79  C             0       -1.767   2.674  -1.239                          C
HETATM   80  F             0       -2.950   2.102  -1.224                          F
HETATM   81  F             0       -1.724   3.634  -0.349                          F
END
```

s2d.pdb

```
TITLE       s2d.pdb
HETATM    1 Ru             0       -0.302   0.021  -0.772                         Ru
HETATM    2  C             0       -0.348  -3.423   1.921                          C
HETATM    3  C             0       -1.834  -3.078   1.874                          C
HETATM    4  H             0        0.092  -3.315   2.931                          H
HETATM    5  H             0       -2.268  -2.884   2.871                          H
HETATM    6  C             0       -0.650  -1.545   0.555                          C
HETATM    7  N             0       -1.858  -1.860   1.061                          N
HETATM    8  N             0        0.249  -2.440   1.009                          N
HETATM    9 Cl             0       -0.722   1.354   1.251                         Cl
HETATM   10 Cl             0       -0.058  -1.631  -2.548                         Cl
HETATM   11  C             0        1.507   0.520  -1.052                          C
HETATM   12  H             0        1.984  -0.265  -1.677                          H
HETATM   13  C             0        2.490   1.467  -0.571                          C
HETATM   14  C             0        2.254   2.778  -0.047                          C
HETATM   15  C             0        3.823   0.990  -0.589                          C
HETATM   16  C             0        3.329   3.489   0.515                          C
HETATM   17  C             0        4.871   1.693  -0.016                          C
HETATM   18  H             0        4.006   0.011  -1.049                          H
HETATM   19  C             0        4.610   2.945   0.548                          C
HETATM   20  H             0        3.174   4.498   0.900                          H
HETATM   21  H             0        5.886   1.280  -0.020                          H
HETATM   22  H             0        5.424   3.529   0.993                          H
HETATM   23  O             0        1.035   3.278  -0.221                          O
HETATM   24  C             0        0.476   4.393   0.520                          C
HETATM   25  H             0       -0.599   4.190   0.397                          H
HETATM   26  C             0       -3.072  -1.161   0.782                          C
HETATM   27  C             0       -3.732  -1.364  -0.444                          C
HETATM   28  C             0       -3.617  -0.311   1.768                          C
HETATM   29  C             0       -4.896  -0.628  -0.705                          C
```

```
HETATM   30  C              0        -4.779   0.399   1.464                       C
HETATM   31  C              0        -5.420   0.270   0.225                       C
HETATM   32  H              0        -5.407  -0.774  -1.666                       H
HETATM   33  H              0        -5.193   1.082   2.218                       H
HETATM   34  C              0         1.640  -2.491   0.695                       C
HETATM   35  C              0         2.100  -3.434  -0.242                       C
HETATM   36  C              0         2.540  -1.655   1.390                       C
HETATM   37  C              0         3.475  -3.493  -0.509                       C
HETATM   38  C              0         3.904  -1.771   1.114                       C
HETATM   39  C              0         4.388  -2.671   0.155                       C
HETATM   40  H              0         3.837  -4.210  -1.257                       H
HETATM   41  H              0         4.608  -1.125   1.655                       H
HETATM   42  C              0        -3.255  -2.357  -1.460                       C
HETATM   43  H              0        -2.705  -1.878  -2.289                       H
HETATM   44  H              0        -2.561  -3.104  -1.040                       H
HETATM   45  H              0        -4.111  -2.894  -1.906                       H
HETATM   46  C              0        -3.001  -0.190   3.128                       C
HETATM   47  H              0        -3.154   0.821   3.542                       H
HETATM   48  H              0        -3.460  -0.904   3.841                       H
HETATM   49  H              0        -1.914  -0.367   3.111                       H
HETATM   50  C              0        -6.639   1.084  -0.088                       C
HETATM   51  H              0        -6.372   2.132  -0.320                       H
HETATM   52  H              0        -7.185   0.689  -0.961                       H
HETATM   53  H              0        -7.340   1.121   0.764                       H
HETATM   54  C              0         1.169  -4.394  -0.916                       C
HETATM   55  H              0         1.494  -4.603  -1.949                       H
HETATM   56  H              0         1.143  -5.366  -0.384                       H
HETATM   57  H              0         0.141  -4.004  -0.976                       H
HETATM   58  C              0         2.050  -0.670   2.404                       C
HETATM   59  H              0         1.469  -1.157   3.212                       H
HETATM   60  H              0         2.891  -0.135   2.877                       H
HETATM   61  H              0         1.369   0.084   1.963                       H
HETATM   62  C              0         5.851  -2.716  -0.166                       C
HETATM   63  H              0         6.475  -2.688   0.745                       H
HETATM   64  H              0         6.124  -3.620  -0.736                       H
HETATM   65  H              0         6.156  -1.845  -0.779                       H
HETATM   66  C              0         0.807   5.704  -0.164                       C
HETATM   67  H              0         0.231   6.522   0.304                       H
HETATM   68  H              0         1.877   5.969  -0.089                       H
HETATM   69  H              0         0.536   5.673  -1.233                       H
HETATM   70  C              0         0.775   4.368   2.009                       C
HETATM   71  H              0         1.716   4.870   2.290                       H
HETATM   72  H              0        -0.040   4.892   2.539                       H
HETATM   73  H              0         0.790   3.331   2.381                       H
HETATM   74  H              0        -2.447  -3.864   1.393                       H
HETATM   75  H              0        -0.128  -4.449   1.574                       H
HETATM   76  C              0         0.047   1.485  -2.461                       C
HETATM   77  H              0         0.673   2.373  -2.347                       H
HETATM   78  H              0         0.169   0.873  -3.363                       H
HETATM   79  C              0        -1.237   1.542  -1.858                       C
HETATM   80  F              0        -2.283   0.947  -2.467                       F
HETATM   81  F              0        -1.674   2.666  -1.293                       F
END
```

s2I.pdb

```
TITLE       S2I.pdb
HETATM    1 Ru              0         0.490  -0.455  -0.334                      Ru
HETATM    2  C              0         1.158   3.626   0.863                       C
HETATM    3  C              0         2.573   3.069   1.021                       C
HETATM    4  H              0         0.756   4.063   1.794                       H
```

HETATM	5	H	0	2.932	3.085	2.068	H
HETATM	6	C	0	1.158	1.365	0.290	C
HETATM	7	N	0	2.436	1.682	0.560	N
HETATM	8	N	0	0.379	2.443	0.485	N
HETATM	9	Cl	0	-0.577	-0.846	1.794	Cl
HETATM	10	Cl	0	1.602	0.193	-2.431	Cl
HETATM	11	C	0	-0.863	-1.326	-1.637	C
HETATM	12	H	0	-0.479	-1.090	-2.640	H
HETATM	13	C	0	-2.312	-1.101	-1.587	C
HETATM	14	C	0	-3.240	-1.639	-0.648	C
HETATM	15	C	0	-2.821	-0.288	-2.624	C
HETATM	16	C	0	-4.605	-1.336	-0.785	C
HETATM	17	C	0	-4.170	0.016	-2.741	C
HETATM	18	H	0	-2.104	0.114	-3.351	H
HETATM	19	C	0	-5.064	-0.515	-1.810	C
HETATM	20	H	0	-5.329	-1.779	-0.098	H
HETATM	21	H	0	-4.524	0.656	-3.557	H
HETATM	22	H	0	-6.138	-0.306	-1.889	H
HETATM	23	O	0	-2.758	-2.480	0.276	O
HETATM	24	C	0	-3.384	-2.736	1.554	C
HETATM	25	H	0	-2.511	-3.069	2.142	H
HETATM	26	C	0	3.542	0.782	0.453	C
HETATM	27	C	0	4.425	0.888	-0.636	C
HETATM	28	C	0	3.736	-0.187	1.457	C
HETATM	29	C	0	5.457	-0.049	-0.747	C
HETATM	30	C	0	4.778	-1.106	1.304	C
HETATM	31	C	0	5.635	-1.064	0.198	C
HETATM	32	H	0	6.134	0.012	-1.610	H
HETATM	33	H	0	4.925	-1.872	2.076	H
HETATM	34	C	0	-1.051	2.433	0.452	C
HETATM	35	C	0	-1.719	2.575	-0.778	C
HETATM	36	C	0	-1.764	2.308	1.661	C
HETATM	37	C	0	-3.119	2.552	-0.780	C
HETATM	38	C	0	-3.159	2.277	1.609	C
HETATM	39	C	0	-3.853	2.387	0.397	C
HETATM	40	H	0	-3.647	2.664	-1.735	H
HETATM	41	H	0	-3.721	2.150	2.544	H
HETATM	42	C	0	4.302	1.986	-1.649	C
HETATM	43	H	0	4.602	1.635	-2.649	H
HETATM	44	H	0	3.269	2.357	-1.740	H
HETATM	45	H	0	4.960	2.842	-1.395	H
HETATM	46	C	0	2.863	-0.227	2.674	C
HETATM	47	H	0	2.754	0.772	3.135	H
HETATM	48	H	0	1.837	-0.579	2.456	H
HETATM	49	H	0	3.284	-0.902	3.438	H
HETATM	50	C	0	6.707	-2.095	0.021	C
HETATM	51	H	0	6.336	-2.955	-0.570	H
HETATM	52	H	0	7.582	-1.695	-0.520	H
HETATM	53	H	0	7.055	-2.500	0.986	H
HETATM	54	C	0	-0.957	2.770	-2.052	C
HETATM	55	H	0	-1.643	2.893	-2.907	H
HETATM	56	H	0	-0.313	3.669	-2.012	H
HETATM	57	H	0	-0.277	1.926	-2.281	H
HETATM	58	C	0	-1.062	2.237	2.983	C
HETATM	59	H	0	-0.939	3.244	3.432	H
HETATM	60	H	0	-1.638	1.632	3.702	H
HETATM	61	H	0	-0.065	1.773	2.903	H
HETATM	62	C	0	-5.351	2.344	0.375	C
HETATM	63	H	0	-5.735	1.414	0.832	H
HETATM	64	H	0	-5.794	3.179	0.950	H
HETATM	65	H	0	-5.748	2.398	-0.652	H
HETATM	66	C	0	-4.365	-3.889	1.462	C

```
HETATM   67  H           0       -4.674  -4.197   2.477                    H
HETATM   68  H           0       -5.281  -3.634   0.902                    H
HETATM   69  H           0       -3.901  -4.763   0.974                    H
HETATM   70  C           0       -3.933  -1.495   2.234                    C
HETATM   71  H           0       -4.938  -1.200   1.888                    H
HETATM   72  H           0       -4.007  -1.689   3.319                    H
HETATM   73  H           0       -3.239  -0.648   2.095                    H
HETATM   74  H           0        3.320   3.601   0.407                    H
HETATM   75  H           0        1.080   4.397   0.073                    H
HETATM   76  C           0       -0.099  -2.508  -1.035                    C
HETATM   77  H           0       -0.688  -3.082  -0.308                    H
HETATM   78  H           0        0.238  -3.118  -1.890                    H
HETATM   79  C           0        1.339  -2.179  -0.361                    C
HETATM   80  F           0        2.364  -2.496  -1.163                    F
HETATM   81  F           0        1.520  -2.900   0.758                    F
END
```

s2e.pdb

```
TITLE      s2e.pdb
HETATM    1 Ru           0       -0.474  -0.554   0.291                   Ru
HETATM    2 Cl           0       -1.523  -0.005   2.450                   Cl
HETATM    3 Cl           0        0.604  -0.763  -1.868                   Cl
HETATM    4  C           0       -1.119   1.346  -0.215                    C
HETATM    5  N           0       -2.397   1.701  -0.438                    N
HETATM    6  C           0       -2.526   3.110  -0.829                    C
HETATM    7  C           0       -1.103   3.640  -0.669                    C
HETATM    8  N           0       -0.334   2.428  -0.372                    N
HETATM    9  C           0       -3.526   0.828  -0.366                    C
HETATM   10  C           0       -4.394   0.904   0.737                    C
HETATM   11  C           0       -5.471   0.013   0.797                    C
HETATM   12  C           0       -5.705  -0.927  -0.211                    C
HETATM   13  C           0       -4.857  -0.941  -1.324                    C
HETATM   14  C           0       -3.771  -0.066  -1.427                    C
HETATM   15  C           0       -4.213   1.928   1.816                    C
HETATM   16  C           0       -6.831  -1.909  -0.094                    C
HETATM   17  C           0       -2.906  -0.067  -2.650                    C
HETATM   18  C           0        1.095   2.416  -0.380                    C
HETATM   19  C           0        1.771   2.383  -1.616                    C
HETATM   20  C           0        3.168   2.367  -1.609                    C
HETATM   21  C           0        3.897   2.397  -0.414                    C
HETATM   22  C           0        3.200   2.463   0.794                    C
HETATM   23  C           0        1.800   2.473   0.835                    C
HETATM   24  C           0        1.030   2.387  -2.919                    C
HETATM   25  C           0        5.396   2.363  -0.439                    C
HETATM   26  C           0        1.075   2.572   2.142                    C
HETATM   27  H           0       -3.256   3.621  -0.178                    H
HETATM   28  H           0       -2.902   3.181  -1.868                    H
HETATM   29  H           0       -0.999   4.366   0.160                    H
HETATM   30  H           0       -0.713   4.124  -1.582                    H
HETATM   31  H           0       -6.139   0.050   1.668                    H
HETATM   32  H           0       -5.046  -1.649  -2.142                    H
HETATM   33  H           0       -4.850   2.817   1.633                    H
HETATM   34  H           0       -3.168   2.263   1.906                    H
HETATM   35  H           0       -4.504   1.522   2.799                    H
HETATM   36  H           0       -7.194  -2.241  -1.082                    H
HETATM   37  H           0       -6.512  -2.819   0.450                    H
HETATM   38  H           0       -7.687  -1.493   0.464                    H
HETATM   39  H           0       -3.311  -0.746  -3.419                    H
HETATM   40  H           0       -2.833   0.940  -3.102                    H
HETATM   41  H           0       -1.868  -0.389  -2.445                    H
```

```
HETATM   42  H            0        3.702   2.314  -2.568                      H
HETATM   43  H            0        3.756   2.509   1.738                      H
HETATM   44  H            0        0.915   3.415  -3.318                      H
HETATM   45  H            0        1.574   1.806  -3.682                      H
HETATM   46  H            0        0.027   1.939  -2.831                      H
HETATM   47  H            0        5.816   3.170  -1.066                      H
HETATM   48  H            0        5.772   1.413  -0.863                      H
HETATM   49  H            0        5.826   2.465   0.572                      H
HETATM   50  H            0        0.429   3.470   2.184                      H
HETATM   51  H            0        1.784   2.633   2.985                      H
HETATM   52  H            0        0.403   1.712   2.327                      H
HETATM   53  C            0        0.791  -1.711   1.551                      C
HETATM   54  C            0        2.195  -1.296   1.597                      C
HETATM   55  C            0        3.218  -1.648   0.672                      C
HETATM   56  C            0        4.532  -1.211   0.911                      C
HETATM   57  C            0        4.841  -0.437   2.026                      C
HETATM   58  C            0        3.847  -0.082   2.940                      C
HETATM   59  C            0        2.548  -0.517   2.720                      C
HETATM   60  O            0        2.860  -2.458  -0.333                      O
HETATM   61  C            0        3.595  -2.623  -1.564                      C
HETATM   62  C            0        4.068  -1.321  -2.184                      C
HETATM   63  C            0        4.674  -3.681  -1.424                      C
HETATM   64  H            0        0.328  -1.643   2.545                      H
HETATM   65  H            0        5.336  -1.510   0.234                      H
HETATM   66  H            0        5.879  -0.120   2.185                      H
HETATM   67  H            0        4.088   0.525   3.820                      H
HETATM   68  H            0        1.749  -0.255   3.426                      H
HETATM   69  H            0        2.802  -3.024  -2.219                      H
HETATM   70  H            0        3.287  -0.548  -2.090                      H
HETATM   71  H            0        4.247  -1.487  -3.261                      H
HETATM   72  H            0        5.011  -0.938  -1.757                      H
HETATM   73  H            0        4.264  -4.606  -0.983                      H
HETATM   74  H            0        5.079  -3.935  -2.419                      H
HETATM   75  H            0        5.521  -3.353  -0.797                      H
HETATM   76  C            0        0.111  -2.676   0.678                      C
HETATM   77  C            0       -1.513  -2.088   0.042                      C
HETATM   78  F            0       -2.484  -2.516   0.846                      F
HETATM   79  F            0       -1.699  -2.722  -1.115                      F
HETATM   80  H            0       -0.407  -3.449   1.267                      H
HETATM   81  H            0        0.657  -3.061  -0.191                      H
END
```

s2f.pdb

```
TITLE      s2f.pdb
HETATM    1 Ru            0       -0.418  -0.699   0.418                     Ru
HETATM    2  C            0       -1.010   3.444  -0.601                      C
HETATM    3  C            0       -2.461   2.991  -0.680                      C
HETATM    4  H            0       -0.637   3.879  -1.547                      H
HETATM    5  H            0       -2.892   3.086  -1.696                      H
HETATM    6  C            0       -1.119   1.140  -0.133                      C
HETATM    7  N            0       -2.389   1.576  -0.302                      N
HETATM    8  N            0       -0.293   2.201  -0.308                      N
HETATM    9 Cl            0        0.341  -1.114  -1.858                     Cl
HETATM   10 Cl            0       -0.940  -0.040   2.728                     Cl
HETATM   11  C            0        1.074  -2.149   1.641                      C
HETATM   12  C            0        2.341  -1.414   1.591                      C
HETATM   13  C            0        3.334  -1.571   0.578                      C
HETATM   14  C            0        2.634  -0.591   2.698                      C
HETATM   15  C            0        4.571  -0.922   0.734                      C
HETATM   16  C            0        3.856   0.055   2.833                      C
```

HETATM	17	H	0	1.857	-0.474	3.464	H
HETATM	18	C	0	4.825	-0.123	1.844	C
HETATM	19	H	0	5.359	-1.066	-0.008	H
HETATM	20	H	0	4.055	0.689	3.704	H
HETATM	21	H	0	5.803	0.367	1.934	H
HETATM	22	O	0	3.033	-2.407	-0.422	O
HETATM	23	C	0	3.729	-2.486	-1.682	C
HETATM	24	H	0	2.994	-3.053	-2.280	H
HETATM	25	C	0	-3.586	0.798	-0.332	C
HETATM	26	C	0	-4.459	0.823	0.772	C
HETATM	27	C	0	-3.918	0.101	-1.510	C
HETATM	28	C	0	-5.654	0.102	0.688	C
HETATM	29	C	0	-5.119	-0.616	-1.545	C
HETATM	30	C	0	-5.994	-0.631	-0.454	C
HETATM	31	H	0	-6.335	0.108	1.549	H
HETATM	32	H	0	-5.377	-1.171	-2.457	H
HETATM	33	C	0	1.135	2.190	-0.397	C
HETATM	34	C	0	1.909	2.469	0.748	C
HETATM	35	C	0	1.734	2.079	-1.668	C
HETATM	36	C	0	3.297	2.565	0.605	C
HETATM	37	C	0	3.127	2.159	-1.757	C
HETATM	38	C	0	3.924	2.388	-0.632	C
HETATM	39	H	0	3.903	2.785	1.493	H
HETATM	40	H	0	3.600	2.049	-2.742	H
HETATM	41	C	0	-4.098	1.562	2.021	C
HETATM	42	H	0	-4.891	1.469	2.781	H
HETATM	43	H	0	-3.158	1.168	2.454	H
HETATM	44	H	0	-3.937	2.642	1.844	H
HETATM	45	C	0	-2.999	0.117	-2.691	C
HETATM	46	H	0	-2.768	1.148	-3.021	H
HETATM	47	H	0	-2.024	-0.360	-2.469	H
HETATM	48	H	0	-3.443	-0.413	-3.549	H
HETATM	49	C	0	-7.259	-1.433	-0.503	C
HETATM	50	H	0	-7.080	-2.484	-0.208	H
HETATM	51	H	0	-8.026	-1.037	0.184	H
HETATM	52	H	0	-7.691	-1.461	-1.518	H
HETATM	53	C	0	1.270	2.699	2.082	C
HETATM	54	H	0	2.026	2.958	2.842	H
HETATM	55	H	0	0.533	3.523	2.051	H
HETATM	56	H	0	0.717	1.811	2.441	H
HETATM	57	C	0	0.922	1.962	-2.923	C
HETATM	58	H	0	0.821	2.948	-3.419	H
HETATM	59	H	0	1.407	1.287	-3.647	H
HETATM	60	H	0	-0.088	1.560	-2.744	H
HETATM	61	C	0	5.417	2.419	-0.744	C
HETATM	62	H	0	5.842	1.402	-0.622	H
HETATM	63	H	0	5.755	2.787	-1.729	H
HETATM	64	H	0	5.880	3.049	0.035	H
HETATM	65	C	0	4.992	-3.322	-1.577	C
HETATM	66	H	0	5.366	-3.555	-2.590	H
HETATM	67	H	0	5.807	-2.814	-1.035	H
HETATM	68	H	0	4.789	-4.279	-1.068	H
HETATM	69	C	0	3.910	-1.142	-2.360	C
HETATM	70	H	0	4.722	-0.530	-1.931	H
HETATM	71	H	0	4.153	-1.308	-3.425	H
HETATM	72	H	0	2.971	-0.567	-2.310	H
HETATM	73	H	0	-3.127	3.539	0.009	H
HETATM	74	H	0	-0.827	4.186	0.198	H
HETATM	75	C	0	0.461	-2.958	0.714	C
HETATM	76	H	0	0.871	-3.169	-0.275	H
HETATM	77	C	0	-1.972	-1.643	0.281	C
HETATM	78	H	0	0.605	-2.110	2.632	H

```
HETATM   79  H             0       -0.383  -3.577   1.045                       H
HETATM   80  F             0       -2.927  -1.778   1.175                       F
HETATM   81  F             0       -2.290  -2.465  -0.703                       F
END
```

a2a.pdb

```
TITLE       a2a.pdb
HETATM    1 Ru             0       -0.001   0.264   0.226                      Ru
HETATM    2  C             0        0.578  -3.925  -0.564                       C
HETATM    3  C             0       -0.937  -3.796  -0.580                       C
HETATM    4  H             0        0.947  -4.598   0.236                       H
HETATM    5  H             0       -1.440  -4.463   0.144                       H
HETATM    6  C             0        0.003  -1.678  -0.087                       C
HETATM    7  N             0       -1.151  -2.400  -0.213                       N
HETATM    8  N             0        1.025  -2.555  -0.317                       N
HETATM    9 Cl             0       -0.675   0.098   2.514                      Cl
HETATM   10 Cl             0       -0.283   0.772  -2.083                      Cl
HETATM   11  C             0        1.810   0.597   0.319                       C
HETATM   12  H             0        2.603  -0.120   0.586                       H
HETATM   13  C             0        2.262   1.910  -0.088                       C
HETATM   14  C             0        1.328   2.967  -0.111                       C
HETATM   15  C             0        3.563   2.143  -0.571                       C
HETATM   16  C             0        1.654   4.201  -0.663                       C
HETATM   17  C             0        3.900   3.384  -1.106                       C
HETATM   18  H             0        4.294   1.324  -0.544                       H
HETATM   19  C             0        2.944   4.402  -1.165                       C
HETATM   20  H             0        0.900   4.990  -0.725                       H
HETATM   21  H             0        4.909   3.557  -1.497                       H
HETATM   22  H             0        3.202   5.370  -1.610                       H
HETATM   23  O             0        0.101   2.689   0.451                       O
HETATM   24  C             0       -0.162   3.313   1.761                       C
HETATM   25  H             0       -0.986   2.688   2.140                       H
HETATM   26  C             0       -2.494  -1.933  -0.093                       C
HETATM   27  C             0       -3.205  -1.551  -1.251                       C
HETATM   28  C             0       -3.147  -2.035   1.154                       C
HETATM   29  C             0       -4.548  -1.181  -1.117                       C
HETATM   30  C             0       -4.485  -1.638   1.241                       C
HETATM   31  C             0       -5.197  -1.194   0.120                       C
HETATM   32  H             0       -5.097  -0.861  -2.013                       H
HETATM   33  H             0       -4.990  -1.692   2.214                       H
HETATM   34  C             0        2.422  -2.328  -0.188                       C
HETATM   35  C             0        3.177  -1.990  -1.325                       C
HETATM   36  C             0        3.024  -2.464   1.076                       C
HETATM   37  C             0        4.554  -1.797  -1.176                       C
HETATM   38  C             0        4.404  -2.261   1.184                       C
HETATM   39  C             0        5.183  -1.926   0.070                       C
HETATM   40  H             0        5.150  -1.527  -2.058                       H
HETATM   41  H             0        4.880  -2.356   2.169                       H
HETATM   42  C             0       -2.598  -1.613  -2.620                       C
HETATM   43  H             0       -2.859  -0.719  -3.211                       H
HETATM   44  H             0       -1.500  -1.671  -2.605                       H
HETATM   45  H             0       -2.986  -2.491  -3.174                       H
HETATM   46  C             0       -2.483  -2.647   2.350                       C
HETATM   47  H             0       -2.746  -2.107   3.274                       H
HETATM   48  H             0       -2.821  -3.695   2.479                       H
HETATM   49  H             0       -1.386  -2.648   2.278                       H
HETATM   50  C             0       -6.609  -0.711   0.249                       C
HETATM   51  H             0       -6.636   0.376   0.463                       H
HETATM   52  H             0       -7.185  -0.863  -0.680                       H
HETATM   53  H             0       -7.146  -1.208   1.074                       H
```

```
HETATM   54  C              0         2.502  -1.775  -2.644                          C
HETATM   55  H              0         3.236  -1.600  -3.447                          H
HETATM   56  H              0         1.876  -2.636  -2.942                          H
HETATM   57  H              0         1.820  -0.903  -2.606                          H
HETATM   58  C              0         2.180  -2.716   2.287                          C
HETATM   59  H              0         1.509  -3.587   2.167                          H
HETATM   60  H              0         2.798  -2.890   3.183                          H
HETATM   61  H              0         1.519  -1.850   2.495                          H
HETATM   62  C              0         6.655  -1.678   0.206                          C
HETATM   63  H              0         7.050  -2.049   1.166                          H
HETATM   64  H              0         7.230  -2.159  -0.605                          H
HETATM   65  H              0         6.889  -0.598   0.155                          H
HETATM   66  C              0        -0.630   4.741   1.596                          C
HETATM   67  H              0        -1.065   5.097   2.547                          H
HETATM   68  H              0         0.195   5.428   1.341                          H
HETATM   69  H              0        -1.408   4.817   0.818                          H
HETATM   70  C              0         1.017   3.156   2.693                          C
HETATM   71  H              0         1.884   3.762   2.373                          H
HETATM   72  H              0         0.729   3.487   3.706                          H
HETATM   73  H              0         1.320   2.097   2.763                          H
HETATM   74  H              0        -1.378  -3.999  -1.576                          H
HETATM   75  H              0         0.996  -4.300  -1.517                          H
HETATM   76  C              0        -3.057   2.269  -0.942                          C
HETATM   77  C              0        -3.109   1.750   0.278                          C
HETATM   78  H              0        -3.594   0.786   0.452                          H
HETATM   79  H              0        -2.672   2.296   1.115                          H
HETATM   80  F              0        -2.499   3.422  -1.247                          F
HETATM   81  F              0        -3.569   1.725  -2.025                          F
END
```

a2b.pdb

```
TITLE       a2b.pdb
HETATM    1 Ru              0         0.594  -0.498  -0.271                         Ru
HETATM    2  C              0         1.081   3.499   1.197                          C
HETATM    3  C              0         2.513   2.987   1.190                          C
HETATM    4  H              0         0.687   3.652   2.222                          H
HETATM    5  H              0         3.003   3.044   2.180                          H
HETATM    6  C              0         1.093   1.296   0.356                          C
HETATM    7  N              0         2.359   1.597   0.774                          N
HETATM    8  N              0         0.339   2.423   0.538                          N
HETATM    9 Cl              0         0.755  -1.350   1.998                         Cl
HETATM   10 Cl              0         1.494  -0.214  -2.494                         Cl
HETATM   11  C              0        -1.060   0.036  -0.937                          C
HETATM   12  H              0        -0.983   0.897  -1.627                          H
HETATM   13  C              0        -2.417  -0.466  -0.932                          C
HETATM   14  C              0        -2.950  -1.397   0.013                          C
HETATM   15  C              0        -3.295   0.065  -1.907                          C
HETATM   16  C              0        -4.306  -1.752  -0.060                          C
HETATM   17  C              0        -4.630  -0.305  -1.982                          C
HETATM   18  H              0        -2.884   0.790  -2.621                          H
HETATM   19  C              0        -5.130  -1.215  -1.047                          C
HETATM   20  H              0        -4.724  -2.475   0.641                          H
HETATM   21  H              0        -5.281   0.114  -2.757                          H
HETATM   22  H              0        -6.182  -1.522  -1.085                          H
HETATM   23  O              0        -2.077  -1.865   0.907                          O
HETATM   24  C              0        -2.425  -2.608   2.093                          C
HETATM   25  H              0        -1.458  -2.575   2.625                          H
HETATM   26  C              0         3.512   0.800   0.490                          C
HETATM   27  C              0         4.140   0.925  -0.769                          C
HETATM   28  C              0         4.054  -0.036   1.485                          C
```

```
HETATM   29  C             0        5.229   0.101  -1.057                          C
HETATM   30  C             0        5.144  -0.850   1.149                          C
HETATM   31  C             0        5.725  -0.815  -0.121                          C
HETATM   32  H             0        5.704   0.179  -2.044                          H
HETATM   33  H             0        5.557  -1.520   1.915                          H
HETATM   34  C             0       -1.072   2.568   0.391                          C
HETATM   35  C             0       -1.567   3.226  -0.752                          C
HETATM   36  C             0       -1.941   2.102   1.393                          C
HETATM   37  C             0       -2.947   3.388  -0.886                          C
HETATM   38  C             0       -3.320   2.279   1.216                          C
HETATM   39  C             0       -3.840   2.914   0.087                          C
HETATM   40  H             0       -3.340   3.877  -1.788                          H
HETATM   41  H             0       -4.007   1.898   1.985                          H
HETATM   42  C             0        3.729   1.980  -1.752                          C
HETATM   43  H             0        3.913   1.653  -2.787                          H
HETATM   44  H             0        2.660   2.236  -1.685                          H
HETATM   45  H             0        4.317   2.907  -1.593                          H
HETATM   46  C             0        3.576  -0.003   2.905                          C
HETATM   47  H             0        3.419  -1.018   3.305                          H
HETATM   48  H             0        4.336   0.486   3.547                          H
HETATM   49  H             0        2.625   0.538   3.020                          H
HETATM   50  C             0        6.858  -1.729  -0.477                          C
HETATM   51  H             0        6.510  -2.574  -1.101                          H
HETATM   52  H             0        7.639  -1.212  -1.061                          H
HETATM   53  H             0        7.335  -2.165   0.418                          H
HETATM   54  C             0       -0.627   3.656  -1.838                          C
HETATM   55  H             0       -1.165   4.150  -2.663                          H
HETATM   56  H             0        0.146   4.360  -1.477                          H
HETATM   57  H             0       -0.077   2.792  -2.261                          H
HETATM   58  C             0       -1.414   1.378   2.589                          C
HETATM   59  H             0       -0.460   1.795   2.958                          H
HETATM   60  H             0       -2.139   1.391   3.420                          H
HETATM   61  H             0       -1.187   0.324   2.343                          H
HETATM   62  C             0       -5.317   3.095  -0.090                          C
HETATM   63  H             0       -5.643   2.799  -1.103                          H
HETATM   64  H             0       -5.893   2.498   0.636                          H
HETATM   65  H             0       -5.618   4.151   0.040                          H
HETATM   66  C             0       -2.735  -4.059   1.783                          C
HETATM   67  H             0       -2.816  -4.629   2.725                          H
HETATM   68  H             0       -3.678  -4.201   1.228                          H
HETATM   69  H             0       -1.923  -4.513   1.190                          H
HETATM   70  C             0       -3.449  -1.907   2.969                          C
HETATM   71  H             0       -4.492  -2.016   2.630                          H
HETATM   72  H             0       -3.394  -2.329   3.988                          H
HETATM   73  H             0       -3.224  -0.830   3.047                          H
HETATM   74  H             0        3.157   3.531   0.471                          H
HETATM   75  H             0        0.952   4.454   0.655                          H
HETATM   76  C             0       -0.311  -3.125  -1.711                          C
HETATM   77  C             0        0.693  -3.053  -0.826                          C
HETATM   78  H             0        1.704  -2.860  -1.195                          H
HETATM   79  H             0        0.523  -3.356   0.209                          H
HETATM   80  F             0       -1.533  -3.501  -1.418                          F
HETATM   81  F             0       -0.224  -2.865  -2.983                          F
END
```

a2c.pdb

```
TITLE       a2c.pdb
HETATM    1 Ru             0        0.699  -0.405  -0.483                         Ru
HETATM    2  C             0        1.067   3.628   0.951                          C
HETATM    3  C             0        2.454   3.048   1.211                          C
```

HETATM	4	H	0	0.610	4.081	1.849	H
HETATM	5	H	0	2.702	2.996	2.291	H
HETATM	6	C	0	1.072	1.366	0.308	C
HETATM	7	N	0	2.346	1.706	0.650	N
HETATM	8	N	0	0.293	2.462	0.520	N
HETATM	9	Cl	0	0.659	-1.691	1.569	Cl
HETATM	10	Cl	0	1.419	0.483	-2.651	Cl
HETATM	11	C	0	-1.040	0.034	-1.004	C
HETATM	12	H	0	-1.023	0.893	-1.702	H
HETATM	13	C	0	-2.361	-0.567	-1.008	C
HETATM	14	C	0	-2.957	-1.330	0.043	C
HETATM	15	C	0	-3.112	-0.360	-2.189	C
HETATM	16	C	0	-4.211	-1.927	-0.174	C
HETATM	17	C	0	-4.350	-0.953	-2.393	C
HETATM	18	H	0	-2.665	0.265	-2.974	H
HETATM	19	C	0	-4.889	-1.748	-1.377	C
HETATM	20	H	0	-4.679	-2.525	0.609	H
HETATM	21	H	0	-4.894	-0.796	-3.330	H
HETATM	22	H	0	-5.867	-2.224	-1.512	H
HETATM	23	O	0	-2.290	-1.343	1.194	O
HETATM	24	C	0	-2.561	-2.213	2.312	C
HETATM	25	H	0	-1.707	-1.958	2.963	H
HETATM	26	C	0	3.447	0.802	0.561	C
HETATM	27	C	0	4.275	0.823	-0.581	C
HETATM	28	C	0	3.710	-0.082	1.630	C
HETATM	29	C	0	5.275	-0.150	-0.695	C
HETATM	30	C	0	4.718	-1.038	1.470	C
HETATM	31	C	0	5.488	-1.105	0.304	C
HETATM	32	H	0	5.907	-0.153	-1.593	H
HETATM	33	H	0	4.908	-1.746	2.288	H
HETATM	34	C	0	-1.128	2.516	0.454	C
HETATM	35	C	0	-1.761	3.120	-0.649	C
HETATM	36	C	0	-1.876	1.986	1.524	C
HETATM	37	C	0	-3.159	3.139	-0.686	C
HETATM	38	C	0	-3.271	2.028	1.447	C
HETATM	39	C	0	-3.929	2.582	0.342	C
HETATM	40	H	0	-3.661	3.580	-1.558	H
HETATM	41	H	0	-3.861	1.604	2.270	H
HETATM	42	C	0	4.172	1.907	-1.609	C
HETATM	43	H	0	4.383	1.525	-2.620	H
HETATM	44	H	0	3.174	2.369	-1.643	H
HETATM	45	H	0	4.916	2.702	-1.398	H
HETATM	46	C	0	3.007	0.051	2.946	C
HETATM	47	H	0	2.908	-0.924	3.450	H
HETATM	48	H	0	3.583	0.716	3.620	H
HETATM	49	H	0	1.991	0.464	2.849	H
HETATM	50	C	0	6.522	-2.177	0.133	C
HETATM	51	H	0	6.073	-3.108	-0.263	H
HETATM	52	H	0	7.313	-1.879	-0.577	H
HETATM	53	H	0	7.003	-2.444	1.090	H
HETATM	54	C	0	-0.955	3.666	-1.788	C
HETATM	55	H	0	-1.591	3.862	-2.667	H
HETATM	56	H	0	-0.462	4.623	-1.532	H
HETATM	57	H	0	-0.154	2.967	-2.098	H
HETATM	58	C	0	-1.179	1.366	2.693	C
HETATM	59	H	0	-0.390	2.027	3.101	H
HETATM	60	H	0	-1.885	1.144	3.512	H
HETATM	61	H	0	-0.681	0.418	2.407	H
HETATM	62	C	0	-5.423	2.532	0.245	C
HETATM	63	H	0	-5.761	1.543	-0.122	H
HETATM	64	H	0	-5.907	2.686	1.225	H
HETATM	65	H	0	-5.820	3.285	-0.456	H

```
HETATM   66  C              0       -2.427  -3.676   1.942                          C
HETATM   67  H              0       -2.496  -4.295   2.854                          H
HETATM   68  H              0       -3.209  -4.024   1.245                          H
HETATM   69  H              0       -1.442  -3.856   1.481                          H
HETATM   70  C              0       -3.835  -1.852   3.056                          C
HETATM   71  H              0       -4.761  -2.204   2.572                          H
HETATM   72  H              0       -3.805  -2.305   4.063                          H
HETATM   73  H              0       -3.915  -0.760   3.189                          H
HETATM   74  H              0        3.261   3.615   0.712                          H
HETATM   75  H              0        1.072   4.401   0.158                          H
HETATM   76  C              0        0.036  -2.745  -1.796                          C
HETATM   77  C              0        1.327  -2.563  -1.451                          C
HETATM   78  H              0        2.017  -2.162  -2.199                          H
HETATM   79  H              0        1.691  -3.021  -0.527                          H
HETATM   80  F              0       -0.851  -3.377  -1.067                          F
HETATM   81  F              0       -0.484  -2.411  -2.947                          F
END
```

a2d.pdb

```
TITLE       a2d.pdb
HETATM    1 Ru              0        0.532  -0.668  -0.093                         Ru
HETATM    2  C              0        1.169   3.616   0.211                          C
HETATM    3  C              0        2.585   3.135   0.514                          C
HETATM    4  H              0        0.748   4.259   1.002                          H
HETATM    5  H              0        2.891   3.323   1.562                          H
HETATM    6  C              0        1.224   1.279   0.116                          C
HETATM    7  N              0        2.495   1.690   0.281                          N
HETATM    8  N              0        0.419   2.358   0.120                          N
HETATM    9 Cl              0       -0.133  -0.475   2.233                         Cl
HETATM   10 Cl              0        1.883  -0.726  -2.151                         Cl
HETATM   11  C              0       -0.844  -1.272  -1.281                          C
HETATM   12  H              0       -0.433  -1.579  -2.259                          H
HETATM   13  C              0       -2.284  -1.138  -1.350                          C
HETATM   14  C              0       -3.189  -1.085  -0.246                          C
HETATM   15  C              0       -2.820  -1.029  -2.656                          C
HETATM   16  C              0       -4.560  -0.898  -0.491                          C
HETATM   17  C              0       -4.174  -0.833  -2.884                          C
HETATM   18  H              0       -2.124  -1.091  -3.502                          H
HETATM   19  C              0       -5.042  -0.766  -1.789                          C
HETATM   20  H              0       -5.259  -0.832   0.347                          H
HETATM   21  H              0       -4.557  -0.741  -3.906                          H
HETATM   22  H              0       -6.116  -0.612  -1.946                          H
HETATM   23  O              0       -2.636  -1.217   0.949                          O
HETATM   24  C              0       -3.303  -1.652   2.147                          C
HETATM   25  H              0       -2.433  -1.946   2.760                          H
HETATM   26  C              0        3.660   0.862   0.268                          C
HETATM   27  C              0        4.478   0.869  -0.882                          C
HETATM   28  C              0        4.004   0.094   1.394                          C
HETATM   29  C              0        5.604   0.046  -0.906                          C
HETATM   30  C              0        5.140  -0.725   1.322                          C
HETATM   31  C              0        5.941  -0.772   0.180                          C
HETATM   32  H              0        6.229   0.031  -1.809                          H
HETATM   33  H              0        5.409  -1.330   2.198                          H
HETATM   34  C              0       -1.004   2.337   0.005                          C
HETATM   35  C              0       -1.597   2.166  -1.261                          C
HETATM   36  C              0       -1.789   2.568   1.153                          C
HETATM   37  C              0       -2.993   2.197  -1.352                          C
HETATM   38  C              0       -3.179   2.587   1.015                          C
HETATM   39  C              0       -3.798   2.404  -0.228                          C
HETATM   40  H              0       -3.462   2.051  -2.333                          H
```

```
HETATM   41  H              0       -3.797   2.752   1.908                      H
HETATM   42  C              0        4.165   1.744  -2.058                      C
HETATM   43  H              0        4.552   1.301  -2.991                      H
HETATM   44  H              0        3.080   1.884  -2.194                      H
HETATM   45  H              0        4.634   2.744  -1.961                      H
HETATM   46  C              0        3.223   0.143   2.671                      C
HETATM   47  H              0        2.532   1.001   2.723                      H
HETATM   48  H              0        2.590  -0.751   2.807                      H
HETATM   49  H              0        3.904   0.191   3.540                      H
HETATM   50  C              0        7.127  -1.685   0.105                      C
HETATM   51  H              0        6.893  -2.597  -0.477                      H
HETATM   52  H              0        7.986  -1.207  -0.397                      H
HETATM   53  H              0        7.457  -2.017   1.104                      H
HETATM   54  C              0       -0.760   1.998  -2.494                      C
HETATM   55  H              0       -1.395   1.923  -3.393                      H
HETATM   56  H              0       -0.076   2.856  -2.644                      H
HETATM   57  H              0       -0.111   1.101  -2.465                      H
HETATM   58  C              0       -1.161   2.830   2.488                      C
HETATM   59  H              0       -1.033   3.917   2.666                      H
HETATM   60  H              0       -1.794   2.449   3.307                      H
HETATM   61  H              0       -0.176   2.346   2.588                      H
HETATM   62  C              0       -5.291   2.461  -0.349                      C
HETATM   63  H              0       -5.788   1.809   0.393                      H
HETATM   64  H              0       -5.674   3.483  -0.170                      H
HETATM   65  H              0       -5.633   2.150  -1.350                      H
HETATM   66  C              0       -4.161  -2.885   1.938                      C
HETATM   67  H              0       -4.395  -3.330   2.921                      H
HETATM   68  H              0       -5.121  -2.689   1.432                      H
HETATM   69  H              0       -3.611  -3.645   1.356                      H
HETATM   70  C              0       -3.981  -0.496   2.853                      C
HETATM   71  H              0       -4.836  -0.080   2.290                      H
HETATM   72  H              0       -4.356  -0.825   3.838                      H
HETATM   73  H              0       -3.249   0.311   3.019                      H
HETATM   74  H              0        3.349   3.586  -0.142                      H
HETATM   75  H              0        1.095   4.170  -0.745                      H
HETATM   76  C              0        0.088  -2.935  -0.237                      C
HETATM   77  C              0        1.349  -2.544   0.306                      C
HETATM   78  H              0        2.237  -2.739  -0.312                      H
HETATM   79  H              0        1.455  -2.628   1.396                      H
HETATM   80  F              0        0.084  -3.649  -1.369                      F
HETATM   81  F              0       -0.865  -3.406   0.569                      F
END
```

a2I.pdb

```
TITLE      a2I.pdb
HETATM    1 Ru              0        0.381  -0.503   0.132                     Ru
HETATM    2  C              0        1.477   3.542  -0.045                      C
HETATM    3  C              0        2.831   2.993   0.391                      C
HETATM    4  H              0        1.078   4.310   0.639                      H
HETATM    5  H              0        3.071   3.227   1.447                      H
HETATM    6  C              0        1.370   1.219   0.086                      C
HETATM    7  N              0        2.666   1.540   0.241                      N
HETATM    8  N              0        0.635   2.344  -0.016                      N
HETATM    9 Cl              0       -0.290  -0.256   2.383                     Cl
HETATM   10 Cl              0        1.758  -0.649  -1.968                     Cl
HETATM   11  C              0       -0.891  -1.604  -1.139                      C
HETATM   12  H              0       -0.375  -1.712  -2.103                      H
HETATM   13  C              0       -2.308  -1.292  -1.360                      C
HETATM   14  C              0       -3.259  -1.078  -0.325                      C
HETATM   15  C              0       -2.747  -1.172  -2.695                      C
```

HETATM	16	C	0	-4.599	-0.815	-0.652	C
HETATM	17	C	0	-4.075	-0.911	-3.014	C
HETATM	18	H	0	-2.012	-1.321	-3.496	H
HETATM	19	C	0	-5.003	-0.739	-1.983	C
HETATM	20	H	0	-5.324	-0.632	0.145	H
HETATM	21	H	0	-4.388	-0.847	-4.062	H
HETATM	22	H	0	-6.054	-0.527	-2.214	H
HETATM	23	O	0	-2.765	-1.058	0.914	O
HETATM	24	C	0	-3.464	-1.484	2.097	C
HETATM	25	H	0	-2.619	-1.805	2.732	H
HETATM	26	C	0	3.793	0.661	0.216	C
HETATM	27	C	0	4.623	0.674	-0.923	C
HETATM	28	C	0	4.088	-0.157	1.318	C
HETATM	29	C	0	5.735	-0.169	-0.947	C
HETATM	30	C	0	5.211	-0.993	1.248	C
HETATM	31	C	0	6.039	-1.018	0.124	C
HETATM	32	H	0	6.375	-0.176	-1.840	H
HETATM	33	H	0	5.446	-1.634	2.108	H
HETATM	34	C	0	-0.794	2.367	-0.098	C
HETATM	35	C	0	-1.433	2.090	-1.324	C
HETATM	36	C	0	-1.538	2.726	1.047	C
HETATM	37	C	0	-2.832	2.138	-1.369	C
HETATM	38	C	0	-2.929	2.767	0.951	C
HETATM	39	C	0	-3.593	2.472	-0.247	C
HETATM	40	H	0	-3.338	1.912	-2.316	H
HETATM	41	H	0	-3.513	3.034	1.842	H
HETATM	42	C	0	4.342	1.564	-2.096	C
HETATM	43	H	0	4.657	1.082	-3.037	H
HETATM	44	H	0	3.267	1.791	-2.198	H
HETATM	45	H	0	4.895	2.522	-2.031	H
HETATM	46	C	0	3.240	-0.161	2.552	C
HETATM	47	H	0	2.723	0.799	2.720	H
HETATM	48	H	0	2.442	-0.923	2.499	H
HETATM	49	H	0	3.845	-0.390	3.446	H
HETATM	50	C	0	7.213	-1.947	0.052	C
HETATM	51	H	0	6.964	-2.860	-0.523	H
HETATM	52	H	0	8.077	-1.484	-0.455	H
HETATM	53	H	0	7.540	-2.278	1.052	H
HETATM	54	C	0	-0.662	1.790	-2.573	C
HETATM	55	H	0	-1.238	2.083	-3.467	H
HETATM	56	H	0	0.314	2.306	-2.601	H
HETATM	57	H	0	-0.422	0.716	-2.672	H
HETATM	58	C	0	-0.865	3.098	2.333	C
HETATM	59	H	0	-0.719	4.194	2.410	H
HETATM	60	H	0	-1.476	2.794	3.200	H
HETATM	61	H	0	0.119	2.615	2.449	H
HETATM	62	C	0	-5.087	2.550	-0.324	C
HETATM	63	H	0	-5.569	2.019	0.517	H
HETATM	64	H	0	-5.440	3.598	-0.272	H
HETATM	65	H	0	-5.472	2.116	-1.262	H
HETATM	66	C	0	-4.364	-2.684	1.876	C
HETATM	67	H	0	-4.617	-3.127	2.855	H
HETATM	68	H	0	-5.316	-2.444	1.373	H
HETATM	69	H	0	-3.845	-3.459	1.286	H
HETATM	70	C	0	-4.133	-0.307	2.778	C
HETATM	71	H	0	-4.965	0.110	2.182	H
HETATM	72	H	0	-4.544	-0.617	3.755	H
HETATM	73	H	0	-3.394	0.492	2.955	H
HETATM	74	H	0	3.669	3.351	-0.230	H
HETATM	75	H	0	1.491	3.973	-1.066	H
HETATM	76	C	0	-0.359	-2.800	-0.294	C
HETATM	77	C	0	0.973	-2.428	0.360	C

```
HETATM   78  H            0       1.826  -2.730  -0.265                   H
HETATM   79  H            0       1.046  -2.749   1.412                   H
HETATM   80  F            0      -0.179  -3.851  -1.162                   F
HETATM   81  F            0      -1.256  -3.245   0.632                   F
END
```

a2e.pdb

```
TITLE      a2e.pdb
HETATM    1 Ru            0       0.592  -0.608  -0.225                  Ru
HETATM    2 Cl            0       1.667  -0.349  -2.414                  Cl
HETATM    3 Cl            0      -0.398  -0.715   1.982                  Cl
HETATM    4  C            0       1.189   1.311   0.199                   C
HETATM    5  N            0       2.461   1.688   0.436                   N
HETATM    6  C            0       2.572   3.111   0.774                   C
HETATM    7  C            0       1.142   3.617   0.604                   C
HETATM    8  N            0       0.392   2.389   0.318                   N
HETATM    9  C            0       3.616   0.848   0.390                   C
HETATM   10  C            0       4.446   0.875  -0.744                   C
HETATM   11  C            0       5.562   0.029  -0.770                   C
HETATM   12  C            0       5.870  -0.815   0.300                   C
HETATM   13  C            0       5.053  -0.783   1.437                   C
HETATM   14  C            0       3.930   0.047   1.507                   C
HETATM   15  C            0       4.186   1.805  -1.889                   C
HETATM   16  C            0       7.041  -1.749   0.229                   C
HETATM   17  C            0       3.090   0.093   2.746                   C
HETATM   18  C            0      -1.033   2.409   0.221                   C
HETATM   19  C            0      -1.804   2.478   1.398                   C
HETATM   20  C            0      -3.194   2.556   1.276                   C
HETATM   21  C            0      -3.823   2.598   0.027                   C
HETATM   22  C            0      -3.028   2.563  -1.122                   C
HETATM   23  C            0      -1.635   2.459  -1.050                   C
HETATM   24  C            0      -1.171   2.507   2.756                   C
HETATM   25  C            0      -5.316   2.676  -0.070                   C
HETATM   26  C            0      -0.804   2.432  -2.296                   C
HETATM   27  H            0       3.291   3.604   0.095                   H
HETATM   28  H            0       2.956   3.228   1.805                   H
HETATM   29  H            0       1.029   4.333  -0.232                   H
HETATM   30  H            0       0.742   4.102   1.512                   H
HETATM   31  H            0       6.203   0.029  -1.661                   H
HETATM   32  H            0       5.300  -1.414   2.301                   H
HETATM   33  H            0       4.743   2.756  -1.768                   H
HETATM   34  H            0       3.115   2.041  -2.001                   H
HETATM   35  H            0       4.514   1.360  -2.843                   H
HETATM   36  H            0       7.830  -1.372  -0.444                   H
HETATM   37  H            0       6.739  -2.740  -0.160                   H
HETATM   38  H            0       7.491  -1.924   1.221                   H
HETATM   39  H            0       2.054  -0.255   2.573                   H
HETATM   40  H            0       3.004   1.121   3.148                   H
HETATM   41  H            0       3.524  -0.535   3.542                   H
HETATM   42  H            0      -3.804   2.583   2.190                   H
HETATM   43  H            0      -3.503   2.610  -2.109                   H
HETATM   44  H            0      -1.045   3.546   3.122                   H
HETATM   45  H            0      -0.188   2.011   2.771                   H
HETATM   46  H            0      -1.802   1.985   3.496                   H
HETATM   47  H            0      -5.722   3.538   0.490                   H
HETATM   48  H            0      -5.657   2.763  -1.115                   H
HETATM   49  H            0      -5.794   1.774   0.359                   H
HETATM   50  H            0      -1.439   2.487  -3.197                   H
HETATM   51  H            0      -0.092   3.279  -2.338                   H
HETATM   52  H            0      -0.185   1.517  -2.380                   H
```

```
HETATM   53  C             0       -0.770  -1.850  -1.369                      C
HETATM   54  C             0       -2.143  -1.329  -1.412                      C
HETATM   55  C             0       -3.173  -1.511  -0.444                      C
HETATM   56  C             0       -4.456  -1.002  -0.712                      C
HETATM   57  C             0       -4.731  -0.328  -1.897                      C
HETATM   58  C             0       -3.729  -0.132  -2.849                      C
HETATM   59  C             0       -2.457  -0.625  -2.595                      C
HETATM   60  O             0       -2.857  -2.243   0.623                      O
HETATM   61  C             0       -3.554  -2.224   1.884                      C
HETATM   62  C             0       -3.894  -0.829   2.373                      C
HETATM   63  C             0       -4.717  -3.197   1.885                      C
HETATM   64  H             0       -0.309  -1.821  -2.366                      H
HETATM   65  H             0       -5.264  -1.174   0.002                      H
HETATM   66  H             0       -5.748   0.041  -2.078                      H
HETATM   67  H             0       -3.939   0.400  -3.784                      H
HETATM   68  H             0       -1.655  -0.483  -3.331                      H
HETATM   69  H             0       -2.769  -2.621   2.552                      H
HETATM   70  H             0       -4.797  -0.397   1.910                      H
HETATM   71  H             0       -3.041  -0.153   2.193                      H
HETATM   72  H             0       -4.072  -0.865   3.463                      H
HETATM   73  H             0       -5.561  -2.861   1.257                      H
HETATM   74  H             0       -5.101  -3.320   2.913                      H
HETATM   75  H             0       -4.400  -4.190   1.524                      H
HETATM   76  C             0       -0.053  -2.836  -0.603                      C
HETATM   77  C             0        1.715  -2.036   0.150                      C
HETATM   78  F             0        0.631  -3.725  -1.355                      F
HETATM   79  F             0       -0.538  -3.479   0.442                      F
HETATM   80  H             0        2.472  -2.389  -0.576                      H
HETATM   81  H             0        1.728  -2.510   1.152                      H
END
```

a2f.pdb

```
TITLE      a2f.pdb
HETATM    1 Ru             0       -0.496  -0.487   0.395                     Ru
HETATM    2  C             0       -1.428   3.514  -0.637                      C
HETATM    3  C             0       -2.847   2.996  -0.447                      C
HETATM    4  H             0       -1.209   3.787  -1.689                      H
HETATM    5  H             0       -3.506   3.198  -1.310                      H
HETATM    6  C             0       -1.341   1.228  -0.113                      C
HETATM    7  N             0       -2.650   1.553  -0.284                      N
HETATM    8  N             0       -0.611   2.367  -0.241                      N
HETATM    9 Cl             0        0.284  -1.097  -1.802                     Cl
HETATM   10 Cl             0       -1.083   0.004   2.721                     Cl
HETATM   11  C             0        1.115  -2.112   1.551                      C
HETATM   12  C             0        2.340  -1.304   1.495                      C
HETATM   13  C             0        3.373  -1.434   0.527                      C
HETATM   14  C             0        2.523  -0.391   2.553                      C
HETATM   15  C             0        4.546  -0.676   0.670                      C
HETATM   16  C             0        3.684   0.362   2.677                      C
HETATM   17  H             0        1.716  -0.287   3.289                      H
HETATM   18  C             0        4.698   0.212   1.732                      C
HETATM   19  H             0        5.363  -0.806  -0.044                      H
HETATM   20  H             0        3.795   1.066   3.510                      H
HETATM   21  H             0        5.627   0.789   1.818                      H
HETATM   22  O             0        3.178  -2.360  -0.420                      O
HETATM   23  C             0        3.770  -2.336  -1.733                      C
HETATM   24  H             0        3.028  -2.925  -2.301                      H
HETATM   25  C             0       -3.757   0.661  -0.344                      C
HETATM   26  C             0       -4.639   0.544   0.748                      C
HETATM   27  C             0       -3.994  -0.037  -1.548                      C
```

```
HETATM   28  C            0       -5.732  -0.321   0.629                    C
HETATM   29  C            0       -5.099  -0.890  -1.622                    C
HETATM   30  C            0       -5.972  -1.052  -0.540                    C
HETATM   31  H            0       -6.415  -0.428   1.482                    H
HETATM   32  H            0       -5.285  -1.436  -2.556                    H
HETATM   33  C            0        0.813   2.425  -0.352                    C
HETATM   34  C            0        1.578   2.756   0.784                    C
HETATM   35  C            0        1.418   2.270  -1.617                    C
HETATM   36  C            0        2.962   2.892   0.638                    C
HETATM   37  C            0        2.807   2.396  -1.709                    C
HETATM   38  C            0        3.594   2.701  -0.594                    C
HETATM   39  H            0        3.564   3.151   1.518                    H
HETATM   40  H            0        3.285   2.261  -2.689                    H
HETATM   41  C            0       -4.413   1.317   2.010                    C
HETATM   42  H            0       -4.956   0.863   2.855                    H
HETATM   43  H            0       -3.343   1.352   2.279                    H
HETATM   44  H            0       -4.775   2.360   1.923                    H
HETATM   45  C            0       -3.080   0.143  -2.719                    C
HETATM   46  H            0       -2.936   1.212  -2.965                    H
HETATM   47  H            0       -2.071  -0.271  -2.521                    H
HETATM   48  H            0       -3.477  -0.358  -3.617                    H
HETATM   49  C            0       -7.130  -2.000  -0.629                    C
HETATM   50  H            0       -6.829  -3.028  -0.350                    H
HETATM   51  H            0       -7.952  -1.714   0.049                    H
HETATM   52  H            0       -7.536  -2.059  -1.654                    H
HETATM   53  C            0        0.929   2.970   2.116                    C
HETATM   54  H            0        1.660   3.325   2.861                    H
HETATM   55  H            0        0.112   3.714   2.066                    H
HETATM   56  H            0        0.467   2.042   2.505                    H
HETATM   57  C            0        0.613   2.047  -2.862                    C
HETATM   58  H            0        0.432   3.002  -3.394                    H
HETATM   59  H            0        1.148   1.383  -3.562                    H
HETATM   60  H            0       -0.361   1.575  -2.662                    H
HETATM   61  C            0        5.082   2.813  -0.722                    C
HETATM   62  H            0        5.550   1.816  -0.833                    H
HETATM   63  H            0        5.378   3.398  -1.612                    H
HETATM   64  H            0        5.539   3.288   0.163                    H
HETATM   65  C            0        5.090  -3.082  -1.747                    C
HETATM   66  H            0        5.436  -3.218  -2.787                    H
HETATM   67  H            0        5.886  -2.545  -1.202                    H
HETATM   68  H            0        4.984  -4.082  -1.294                    H
HETATM   69  C            0        3.823  -0.954  -2.357                    C
HETATM   70  H            0        4.689  -0.349  -2.036                    H
HETATM   71  H            0        3.889  -1.058  -3.455                    H
HETATM   72  H            0        2.897  -0.402  -2.128                    H
HETATM   73  H            0       -3.334   3.418   0.453                    H
HETATM   74  H            0       -1.195   4.392  -0.010                    H
HETATM   75  C            0        0.588  -3.144   0.842                    C
HETATM   76  C            0       -2.008  -1.477   0.240                    C
HETATM   77  H            0       -2.184  -2.133  -0.642                    H
HETATM   78  H            0       -2.769  -1.544   1.046                    H
HETATM   79  H            0        0.552  -1.980   2.482                    H
HETATM   80  F            0       -0.462  -3.792   1.318                    F
HETATM   81  F            0        0.963  -3.710  -0.262                    F
END
```

sc3a.pdb

```
TITLE     sc3a.pdb
REMARK   1 File created by GaussView 5.0.8
HETATM    1 Ru            0        0.073   0.277   0.091                   Ru
```

HETATM	2	C	0	1.104	-3.893	0.274		C
HETATM	3	C	0	-0.413	-3.914	0.406		C
HETATM	4	H	0	1.622	-4.331	1.148		H
HETATM	5	H	0	-0.759	-4.312	1.380		H
HETATM	6	C	0	0.303	-1.669	0.151		C
HETATM	7	N	0	-0.773	-2.506	0.281		N
HETATM	8	N	0	1.410	-2.467	0.162		N
HETATM	9	Cl	0	-0.263	0.461	2.435		Cl
HETATM	10	Cl	0	-0.723	0.391	-2.150		Cl
HETATM	11	C	0	1.804	0.881	-0.108		C
HETATM	12	H	0	2.689	0.232	-0.199		H
HETATM	13	C	0	2.092	2.293	-0.167		C
HETATM	14	C	0	1.034	3.228	-0.027		C
HETATM	15	C	0	3.405	2.770	-0.351		C
HETATM	16	C	0	1.297	4.598	-0.068		C
HETATM	17	C	0	3.666	4.135	-0.391		C
HETATM	18	H	0	4.215	2.037	-0.460		H
HETATM	19	C	0	2.610	5.040	-0.248		C
HETATM	20	H	0	0.493	5.329	0.029		H
HETATM	21	H	0	4.689	4.500	-0.532		H
HETATM	22	H	0	2.805	6.118	-0.279		H
HETATM	23	O	0	-0.177	2.640	0.115		O
HETATM	24	C	0	-1.380	3.320	0.584		C
HETATM	25	H	0	-2.047	2.454	0.734		H
HETATM	26	C	0	-2.150	-2.143	0.183		C
HETATM	27	C	0	-2.777	-2.182	-1.081		C
HETATM	28	C	0	-2.901	-1.903	1.353		C
HETATM	29	C	0	-4.139	-1.879	-1.161		C
HETATM	30	C	0	-4.259	-1.588	1.221		C
HETATM	31	C	0	-4.892	-1.559	-0.025		C
HETATM	32	H	0	-4.626	-1.884	-2.145		H
HETATM	33	H	0	-4.842	-1.378	2.128		H
HETATM	34	C	0	2.766	-2.073	-0.000		C
HETATM	35	C	0	3.295	-1.950	-1.297		C
HETATM	36	C	0	3.551	-1.814	1.137		C
HETATM	37	C	0	4.635	-1.574	-1.439		C
HETATM	38	C	0	4.887	-1.438	0.954		C
HETATM	39	C	0	5.443	-1.313	-0.325		C
HETATM	40	H	0	5.055	-1.472	-2.448		H
HETATM	41	H	0	5.505	-1.227	1.836		H
HETATM	42	C	0	-2.044	-2.620	-2.313		C
HETATM	43	H	0	-2.401	-2.077	-3.203		H
HETATM	44	H	0	-0.958	-2.451	-2.248		H
HETATM	45	H	0	-2.213	-3.700	-2.503		H
HETATM	46	C	0	-2.321	-2.094	2.722		C
HETATM	47	H	0	-2.650	-1.301	3.414		H
HETATM	48	H	0	-2.665	-3.057	3.148		H
HETATM	49	H	0	-1.222	-2.087	2.725		H
HETATM	50	C	0	-6.335	-1.176	-0.142		C
HETATM	51	H	0	-6.452	-0.076	-0.133		H
HETATM	52	H	0	-6.786	-1.541	-1.080		H
HETATM	53	H	0	-6.934	-1.562	0.701		H
HETATM	54	C	0	2.412	-2.138	-2.492		C
HETATM	55	H	0	2.984	-2.063	-3.432		H
HETATM	56	H	0	1.899	-3.118	-2.491		H
HETATM	57	H	0	1.613	-1.371	-2.522		H
HETATM	58	C	0	2.942	-1.871	2.503		C
HETATM	59	H	0	2.472	-2.849	2.716		H
HETATM	60	H	0	3.696	-1.688	3.287		H
HETATM	61	H	0	2.142	-1.115	2.617		H
HETATM	62	C	0	6.866	-0.872	-0.500		C
HETATM	63	H	0	7.503	-1.187	0.344		H

```
HETATM   64  H              0        7.310  -1.266  -1.430                    H
HETATM   65  H              0        6.941   0.231  -0.556                    H
HETATM   66  C              0       -1.976   4.203  -0.492                    C
HETATM   67  H              0       -3.022   4.436  -0.229                    H
HETATM   68  H              0       -1.447   5.164  -0.614                    H
HETATM   69  H              0       -1.985   3.681  -1.463                    H
HETATM   70  C              0       -1.175   3.980   1.930                    C
HETATM   71  H              0       -0.542   4.882   1.886                    H
HETATM   72  H              0       -2.158   4.288   2.326                    H
HETATM   73  H              0       -0.730   3.266   2.643                    H
HETATM   74  H              0       -0.908  -4.509  -0.385                    H
HETATM   75  H              0        1.469  -4.434  -0.620                    H
HETATM   76  C              0       -4.342   1.725  -2.102                    C
HETATM   77  H              0       -5.226   2.346  -2.290                    H
HETATM   78  H              0       -3.749   1.362  -2.946                    H
HETATM   79  C              0       -3.954   1.408  -0.872                    C
HETATM   80  H              0       -3.080   0.791  -0.624                    H
HETATM   81  F              0       -4.610   1.827   0.222                    F
END
```

sc3d.pdb

```
TITLE       sc3d.pdb
HETATM    1 Ru              0       -0.591   0.344  -0.578                   Ru
HETATM    2  C              0       -1.081  -3.577   1.322                    C
HETATM    3  C              0       -2.465  -2.944   1.467                    C
HETATM    4  H              0       -0.637  -3.882   2.286                    H
HETATM    5  H              0       -2.733  -2.727   2.520                    H
HETATM    6  C              0       -1.046  -1.454   0.348                    C
HETATM    7  N              0       -2.322  -1.696   0.717                    N
HETATM    8  N              0       -0.289  -2.501   0.713                    N
HETATM    9 Cl              0        0.517   0.726   1.583                   Cl
HETATM   10 Cl              0       -1.747  -0.380  -2.614                   Cl
HETATM   11  C              0        1.039   0.478  -1.507                    C
HETATM   12  H              0        0.923  -0.023  -2.495                    H
HETATM   13  C              0        2.415   0.861  -1.264                    C
HETATM   14  C              0        2.892   1.920  -0.428                    C
HETATM   15  C              0        3.369   0.066  -1.939                    C
HETATM   16  C              0        4.275   2.041  -0.214                    C
HETATM   17  C              0        4.733   0.196  -1.719                    C
HETATM   18  H              0        2.997  -0.699  -2.632                    H
HETATM   19  C              0        5.178   1.179  -0.834                    C
HETATM   20  H              0        4.663   2.838   0.422                    H
HETATM   21  H              0        5.443  -0.462  -2.233                    H
HETATM   22  H              0        6.250   1.307  -0.644                    H
HETATM   23  O              0        1.977   2.788   0.001                    O
HETATM   24  C              0        2.200   3.835   0.969                    C
HETATM   25  H              0        1.158   4.126   1.185                    H
HETATM   26  C              0       -3.374  -0.745   0.539                    C
HETATM   27  C              0       -4.307  -0.940  -0.506                    C
HETATM   28  C              0       -3.478   0.363   1.403                    C
HETATM   29  C              0       -5.281   0.034  -0.720                    C
HETATM   30  C              0       -4.469   1.322   1.141                    C
HETATM   31  C              0       -5.363   1.184   0.079                    C
HETATM   32  H              0       -5.992  -0.102  -1.545                    H
HETATM   33  H              0       -4.544   2.194   1.805                    H
HETATM   34  C              0        1.125  -2.597   0.554                    C
HETATM   35  C              0        1.657  -2.919  -0.707                    C
HETATM   36  C              0        1.961  -2.396   1.669                    C
HETATM   37  C              0        3.047  -3.014  -0.840                    C
HETATM   38  C              0        3.344  -2.478   1.485                    C
```

```
HETATM   39  C              0        3.904  -2.783   0.240                        C
HETATM   40  H              0        3.471  -3.266  -1.822                        H
HETATM   41  H              0        4.003  -2.299   2.345                        H
HETATM   42  C              0       -4.277  -2.179  -1.347                        C
HETATM   43  H              0       -4.747  -2.002  -2.329                        H
HETATM   44  H              0       -3.248  -2.525  -1.535                        H
HETATM   45  H              0       -4.836  -3.005  -0.863                        H
HETATM   46  C              0       -2.626   0.534   2.623                        C
HETATM   47  H              0       -1.981  -0.333   2.830                        H
HETATM   48  H              0       -1.946   1.398   2.531                        H
HETATM   49  H              0       -3.266   0.712   3.507                        H
HETATM   50  C              0       -6.395   2.233  -0.207                        C
HETATM   51  H              0       -6.149   2.798  -1.126                        H
HETATM   52  H              0       -7.394   1.792  -0.374                        H
HETATM   53  H              0       -6.479   2.965   0.613                        H
HETATM   54  C              0        0.754  -3.173  -1.876                        C
HETATM   55  H              0        1.332  -3.452  -2.773                        H
HETATM   56  H              0        0.043  -3.997  -1.674                        H
HETATM   57  H              0        0.129  -2.297  -2.138                        H
HETATM   58  C              0        1.407  -2.144   3.038                        C
HETATM   59  H              0        1.383  -3.076   3.637                        H
HETATM   60  H              0        2.033  -1.423   3.590                        H
HETATM   61  H              0        0.390  -1.721   3.008                        H
HETATM   62  C              0        5.392  -2.844   0.075                        C
HETATM   63  H              0        5.846  -1.840   0.174                        H
HETATM   64  H              0        5.868  -3.480   0.844                        H
HETATM   65  H              0        5.683  -3.237  -0.914                        H
HETATM   66  C              0        2.899   5.027   0.341                        C
HETATM   67  H              0        2.865   5.885   1.037                        H
HETATM   68  H              0        3.960   4.836   0.106                        H
HETATM   69  H              0        2.394   5.333  -0.591                        H
HETATM   70  C              0        2.806   3.358   2.276                        C
HETATM   71  H              0        3.901   3.236   2.249                        H
HETATM   72  H              0        2.583   4.103   3.060                        H
HETATM   73  H              0        2.346   2.402   2.577                        H
HETATM   74  H              0       -3.273  -3.564   1.040                        H
HETATM   75  H              0       -1.079  -4.464   0.660                        H
HETATM   76  C              0       -0.437   2.318  -1.747                        C
HETATM   77  H              0        0.458   2.943  -1.687                        H
HETATM   78  H              0       -0.821   2.043  -2.736                        H
HETATM   79  C              0       -1.322   2.322  -0.657                        C
HETATM   80  F              0       -1.123   3.154   0.386                        F
HETATM   81  H              0       -2.396   2.104  -0.795                        H
END
```

sc3I.pdb

```
TITLE      sc3I.pdb
HETATM    1 Ru              0        0.511  -0.487  -0.388                       Ru
HETATM    2  C              0        1.300   3.477   1.074                        C
HETATM    3  C              0        2.702   2.874   1.168                        C
HETATM    4  H              0        0.926   3.864   2.039                        H
HETATM    5  H              0        3.065   2.780   2.210                        H
HETATM    6  C              0        1.235   1.266   0.328                        C
HETATM    7  N              0        2.528   1.542   0.579                        N
HETATM    8  N              0        0.485   2.340   0.636                        N
HETATM    9 Cl              0       -0.551  -0.920   1.734                       Cl
HETATM   10 Cl              0        1.739   0.067  -2.462                       Cl
HETATM   11  C              0       -0.891  -1.195  -1.705                        C
HETATM   12  H              0       -0.544  -0.876  -2.700                        H
HETATM   13  C              0       -2.345  -1.010  -1.607                        C
```

HETATM	14	C	0	-3.237	-1.622	-0.680	C
HETATM	15	C	0	-2.901	-0.154	-2.583	C
HETATM	16	C	0	-4.613	-1.349	-0.769	C
HETATM	17	C	0	-4.259	0.125	-2.650	C
HETATM	18	H	0	-2.215	0.307	-3.305	H
HETATM	19	C	0	-5.118	-0.480	-1.732	C
HETATM	20	H	0	-5.307	-1.851	-0.092	H
HETATM	21	H	0	-4.648	0.804	-3.418	H
HETATM	22	H	0	-6.197	-0.292	-1.772	H
HETATM	23	O	0	-2.713	-2.496	0.188	O
HETATM	24	C	0	-3.291	-2.815	1.475	C
HETATM	25	H	0	-2.389	-3.130	2.028	H
HETATM	26	C	0	3.611	0.623	0.425	C
HETATM	27	C	0	4.514	0.792	-0.643	C
HETATM	28	C	0	3.772	-0.419	1.357	C
HETATM	29	C	0	5.540	-0.144	-0.798	C
HETATM	30	C	0	4.811	-1.336	1.159	C
HETATM	31	C	0	5.692	-1.223	0.081	C
HETATM	32	H	0	6.233	-0.032	-1.642	H
HETATM	33	H	0	4.934	-2.156	1.878	H
HETATM	34	C	0	-0.940	2.395	0.551	C
HETATM	35	C	0	-1.551	2.596	-0.699	C
HETATM	36	C	0	-1.706	2.271	1.727	C
HETATM	37	C	0	-2.948	2.647	-0.758	C
HETATM	38	C	0	-3.098	2.308	1.618	C
HETATM	39	C	0	-3.736	2.488	0.385	C
HETATM	40	H	0	-3.431	2.804	-1.730	H
HETATM	41	H	0	-3.702	2.181	2.527	H
HETATM	42	C	0	4.400	1.950	-1.587	C
HETATM	43	H	0	4.765	1.678	-2.591	H
HETATM	44	H	0	3.356	2.283	-1.704	H
HETATM	45	H	0	5.006	2.814	-1.246	H
HETATM	46	C	0	2.867	-0.564	2.541	C
HETATM	47	H	0	2.531	0.410	2.939	H
HETATM	48	H	0	1.945	-1.122	2.298	H
HETATM	49	H	0	3.373	-1.108	3.356	H
HETATM	50	C	0	6.769	-2.241	-0.146	C
HETATM	51	H	0	6.464	-2.981	-0.910	H
HETATM	52	H	0	7.703	-1.781	-0.513	H
HETATM	53	H	0	7.003	-2.807	0.772	H
HETATM	54	C	0	-0.731	2.751	-1.944	C
HETATM	55	H	0	-1.358	3.070	-2.794	H
HETATM	56	H	0	0.076	3.498	-1.825	H
HETATM	57	H	0	-0.219	1.813	-2.238	H
HETATM	58	C	0	-1.062	2.124	3.072	C
HETATM	59	H	0	-0.924	3.107	3.567	H
HETATM	60	H	0	-1.687	1.512	3.743	H
HETATM	61	H	0	-0.080	1.628	3.009	H
HETATM	62	C	0	-5.233	2.507	0.305	C
HETATM	63	H	0	-5.665	1.560	0.680	H
HETATM	64	H	0	-5.668	3.314	0.923	H
HETATM	65	H	0	-5.590	2.644	-0.729	H
HETATM	66	C	0	-4.228	-4.003	1.367	C
HETATM	67	H	0	-4.504	-4.355	2.377	H
HETATM	68	H	0	-5.165	-3.765	0.833	H
HETATM	69	H	0	-3.742	-4.842	0.841	H
HETATM	70	C	0	-3.870	-1.621	2.212	C
HETATM	71	H	0	-4.899	-1.361	1.911	H
HETATM	72	H	0	-3.897	-1.854	3.292	H
HETATM	73	H	0	-3.220	-0.739	2.081	H
HETATM	74	H	0	3.459	3.445	0.603	H
HETATM	75	H	0	1.230	4.299	0.336	H

```
HETATM   76  C              0       -0.116  -2.463  -1.262                           C
HETATM   77  H              0       -0.711  -3.109  -0.604                           H
HETATM   78  H              0        0.148  -2.958  -2.213                           H
HETATM   79  C              0        1.307  -2.245  -0.591                           C
HETATM   80  F              0        1.496  -3.055   0.480                           F
HETATM   81  H              0        2.161  -2.357  -1.281                           H
END
```

sc3e.pdb

```
TITLE       sc3e.pdb
HETATM    1 Ru              0       -0.497  -0.625   0.355                          Ru
HETATM    2 Cl              0       -1.604  -0.155   2.505                          Cl
HETATM    3 Cl              0        0.549  -0.858  -1.828                          Cl
HETATM    4  C              0       -1.190   1.242  -0.222                           C
HETATM    5  N              0       -2.473   1.583  -0.442                           N
HETATM    6  C              0       -2.619   2.978  -0.875                           C
HETATM    7  C              0       -1.202   3.528  -0.733                           C
HETATM    8  N              0       -0.421   2.330  -0.411                           N
HETATM    9  C              0       -3.598   0.706  -0.349                           C
HETATM   10  C              0       -4.459   0.795   0.759                           C
HETATM   11  C              0       -5.531  -0.101   0.838                           C
HETATM   12  C              0       -5.765  -1.059  -0.154                           C
HETATM   13  C              0       -4.917  -1.090  -1.267                           C
HETATM   14  C              0       -3.839  -0.208  -1.394                           C
HETATM   15  C              0       -4.268   1.832   1.823                           C
HETATM   16  C              0       -6.891  -2.039  -0.018                           C
HETATM   17  C              0       -2.972  -0.229  -2.614                           C
HETATM   18  C              0        1.009   2.347  -0.418                           C
HETATM   19  C              0        1.687   2.321  -1.653                           C
HETATM   20  C              0        3.083   2.348  -1.641                           C
HETATM   21  C              0        3.809   2.414  -0.446                           C
HETATM   22  C              0        3.106   2.465   0.760                           C
HETATM   23  C              0        1.708   2.431   0.799                           C
HETATM   24  C              0        0.950   2.278  -2.957                           C
HETATM   25  C              0        5.307   2.423  -0.468                           C
HETATM   26  C              0        0.975   2.507   2.104                           C
HETATM   27  H              0       -3.355   3.501  -0.239                           H
HETATM   28  H              0       -2.998   3.015  -1.915                           H
HETATM   29  H              0       -1.103   4.272   0.081                           H
HETATM   30  H              0       -0.821   3.996  -1.657                           H
HETATM   31  H              0       -6.197  -0.052   1.710                           H
HETATM   32  H              0       -5.103  -1.816  -2.070                           H
HETATM   33  H              0       -3.217   2.152   1.908                           H
HETATM   34  H              0       -4.567   1.447   2.811                           H
HETATM   35  H              0       -4.890   2.728   1.626                           H
HETATM   36  H              0       -7.754  -1.605   0.516                           H
HETATM   37  H              0       -7.243  -2.401  -0.999                           H
HETATM   38  H              0       -6.581  -2.931   0.559                           H
HETATM   39  H              0       -1.925  -0.510  -2.391                           H
HETATM   40  H              0       -2.927   0.763  -3.102                           H
HETATM   41  H              0       -3.353  -0.948  -3.358                           H
HETATM   42  H              0        3.622   2.302  -2.598                           H
HETATM   43  H              0        3.659   2.538   1.704                           H
HETATM   44  H              0        1.495   1.665  -3.695                           H
HETATM   45  H              0       -0.054   1.836  -2.858                           H
HETATM   46  H              0        0.842   3.290  -3.397                           H
HETATM   47  H              0        5.710   1.473  -0.867                           H
HETATM   48  H              0        5.706   3.225  -1.116                           H
HETATM   49  H              0        5.733   2.561   0.541                           H
HETATM   50  H              0        1.678   2.603   2.948                           H
```

```
HETATM   51  H              0        0.342   1.619   2.294                        H
HETATM   52  H              0        0.288   3.374   2.139                        H
HETATM   53  C              0        0.809  -1.798   1.633                        C
HETATM   54  C              0        2.191  -1.304   1.634                        C
HETATM   55  C              0        3.205  -1.627   0.688                        C
HETATM   56  C              0        4.511  -1.153   0.899                        C
HETATM   57  C              0        4.819  -0.364   2.006                        C
HETATM   58  C              0        3.833  -0.031   2.934                        C
HETATM   59  C              0        2.542  -0.504   2.742                        C
HETATM   60  O              0        2.851  -2.450  -0.306                        O
HETATM   61  C              0        3.551  -2.578  -1.562                        C
HETATM   62  C              0        4.642  -3.628  -1.471                        C
HETATM   63  C              0        4.000  -1.260  -2.167                        C
HETATM   64  H              0        0.358  -1.713   2.631                        H
HETATM   65  H              0        5.309  -1.436   0.210                        H
HETATM   66  H              0        5.852  -0.019   2.144                        H
HETATM   67  H              0        4.072   0.587   3.807                        H
HETATM   68  H              0        1.752  -0.264   3.465                        H
HETATM   69  H              0        5.037  -3.847  -2.479                        H
HETATM   70  H              0        4.251  -4.570  -1.051                        H
HETATM   71  H              0        5.493  -3.306  -0.846                        H
HETATM   72  H              0        2.742  -2.970  -2.204                        H
HETATM   73  H              0        4.119  -1.395  -3.257                        H
HETATM   74  H              0        4.967  -0.896  -1.781                        H
HETATM   75  H              0        3.232  -0.484  -2.010                        H
HETATM   76  C              0        0.143  -2.748   0.783                        C
HETATM   77  C              0       -1.636  -2.063   0.139                        C
HETATM   78  H              0       -0.462  -3.489   1.328                        H
HETATM   79  H              0        0.632  -3.123  -0.123                        H
HETATM   80  F              0       -1.852  -2.760  -0.977                        F
HETATM   81  H              0       -2.388  -2.364   0.899                        H
END
```

sc3f.pdb

```
TITLE       sc3f.pdb
HETATM    1 Ru              0       -0.486  -0.740   0.419                       Ru
HETATM    2 Cl              0       -1.114   0.001   2.687                       Cl
HETATM    3 Cl              0        0.317  -1.194  -1.831                       Cl
HETATM    4  C              0       -1.187   1.090  -0.183                        C
HETATM    5  N              0       -2.463   1.519  -0.331                        N
HETATM    6  C              0       -2.560   2.953  -0.612                        C
HETATM    7  C              0       -1.104   3.365  -0.773                        C
HETATM    8  N              0       -0.377   2.155  -0.382                        N
HETATM    9  C              0       -3.648   0.726  -0.312                        C
HETATM   10  C              0       -4.507   0.770   0.803                        C
HETATM   11  C              0       -5.655  -0.031   0.783                        C
HETATM   12  C              0       -5.970  -0.844  -0.311                        C
HETATM   13  C              0       -5.119  -0.829  -1.423                        C
HETATM   14  C              0       -3.961  -0.047  -1.449                        C
HETATM   15  C              0       -4.214   1.646   1.981                        C
HETATM   16  C              0       -7.183  -1.724  -0.291                        C
HETATM   17  C              0       -3.064  -0.039  -2.647                        C
HETATM   18  C              0        1.053   2.159  -0.478                        C
HETATM   19  C              0        1.656   2.026  -1.743                        C
HETATM   20  C              0        3.050   2.124  -1.825                        C
HETATM   21  C              0        3.838   2.389  -0.703                        C
HETATM   22  C              0        3.204   2.576   0.530                        C
HETATM   23  C              0        1.817   2.465   0.668                        C
HETATM   24  C              0        0.857   1.848  -2.998                        C
HETATM   25  C              0        5.332   2.441  -0.806                        C
```

```
HETATM   26  C            0       1.163   2.708   1.992                   C
HETATM   27  H            0      -3.063   3.469   0.228                   H
HETATM   28  H            0      -3.169   3.125  -1.517                   H
HETATM   29  H            0      -0.813   4.214  -0.130                   H
HETATM   30  H            0      -0.844   3.638  -1.815                   H
HETATM   31  H            0      -6.321  -0.020   1.656                   H
HETATM   32  H            0      -5.363  -1.443  -2.300                   H
HETATM   33  H            0      -3.130   1.756   2.151                   H
HETATM   34  H            0      -4.651   1.230   2.904                   H
HETATM   35  H            0      -4.649   2.657   1.852                   H
HETATM   36  H            0      -6.928  -2.753   0.027                   H
HETATM   37  H            0      -7.951  -1.355   0.410                   H
HETATM   38  H            0      -7.645  -1.812  -1.290                   H
HETATM   39  H            0      -2.083  -0.512  -2.439                   H
HETATM   40  H            0      -2.840   0.991  -2.985                   H
HETATM   41  H            0      -3.521  -0.578  -3.492                   H
HETATM   42  H            0       3.530   1.996  -2.805                   H
HETATM   43  H            0       3.804   2.820   1.415                   H
HETATM   44  H            0       1.359   1.152  -3.690                   H
HETATM   45  H            0      -0.147   1.436  -2.811                   H
HETATM   46  H            0       0.745   2.811  -3.536                   H
HETATM   47  H            0       5.777   1.449  -0.591                   H
HETATM   48  H            0       5.670   2.730  -1.817                   H
HETATM   49  H            0       5.773   3.147  -0.081                   H
HETATM   50  H            0       1.908   2.984   2.757                   H
HETATM   51  H            0       0.607   1.824   2.358                   H
HETATM   52  H            0       0.419   3.526   1.940                   H
HETATM   53  C            0       0.992  -2.144   1.708                   C
HETATM   54  C            0       2.252  -1.393   1.674                   C
HETATM   55  C            0       3.273  -1.563   0.691                   C
HETATM   56  C            0       4.494  -0.889   0.855                   C
HETATM   57  C            0       4.705  -0.044   1.940                   C
HETATM   58  C            0       3.707   0.152   2.896                   C
HETATM   59  C            0       2.501  -0.523   2.756                   C
HETATM   60  O            0       3.014  -2.439  -0.288                   O
HETATM   61  C            0       3.722  -2.518  -1.541                   C
HETATM   62  C            0       4.996  -3.331  -1.418                   C
HETATM   63  C            0       3.885  -1.176  -2.229                   C
HETATM   64  H            0       0.491  -2.085   2.683                   H
HETATM   65  H            0       5.303  -1.045   0.138                   H
HETATM   66  H            0       5.671   0.468   2.037                   H
HETATM   67  H            0       3.870   0.824   3.746                   H
HETATM   68  H            0       1.702  -0.391   3.496                   H
HETATM   69  H            0       5.390  -3.559  -2.424                   H
HETATM   70  H            0       4.803  -4.291  -0.909                   H
HETATM   71  H            0       5.794  -2.808  -0.864                   H
HETATM   72  H            0       3.000  -3.101  -2.141                   H
HETATM   73  H            0       4.709  -0.562  -1.825                   H
HETATM   74  H            0       2.946  -0.603  -2.151                   H
HETATM   75  H            0       4.096  -1.343  -3.300                   H
HETATM   76  C            0       0.422  -2.977   0.777                   C
HETATM   77  C            0      -2.063  -1.616   0.339                   C
HETATM   78  H            0      -2.929  -1.467   1.020                   H
HETATM   79  H            0      -0.439  -3.584   1.079                   H
HETATM   80  F            0      -2.349  -2.620  -0.489                   F
HETATM   81  H            0       0.871  -3.203  -0.191                   H
END
```

st3a.pdb

```
TITLE      st3a.pdb
```

HETATM	1	Ru	0	0.085	0.330	0.017	Ru
HETATM	2	C	0	0.845	-3.814	0.780	C
HETATM	3	C	0	-0.661	-3.801	0.581	C
HETATM	4	H	0	1.132	-3.993	1.834	H
HETATM	5	H	0	-1.221	-4.204	1.444	H
HETATM	6	C	0	0.172	-1.622	0.204	C
HETATM	7	N	0	-0.954	-2.383	0.392	N
HETATM	8	N	0	1.235	-2.466	0.370	N
HETATM	9	Cl	0	0.000	0.599	2.377	Cl
HETATM	10	Cl	0	-0.955	0.493	-2.122	Cl
HETATM	11	C	0	1.834	0.772	-0.377	C
HETATM	12	H	0	2.634	0.047	-0.593	H
HETATM	13	C	0	2.245	2.155	-0.425	C
HETATM	14	C	0	1.270	3.167	-0.225	C
HETATM	15	C	0	3.584	2.532	-0.643	C
HETATM	16	C	0	1.636	4.513	-0.235	C
HETATM	17	C	0	3.949	3.874	-0.655	C
HETATM	18	H	0	4.331	1.743	-0.797	H
HETATM	19	C	0	2.974	4.855	-0.450	C
HETATM	20	H	0	0.899	5.302	-0.074	H
HETATM	21	H	0	4.993	4.162	-0.822	H
HETATM	22	H	0	3.254	5.915	-0.454	H
HETATM	23	O	0	0.033	2.659	-0.033	O
HETATM	24	C	0	-1.190	3.428	0.129	C
HETATM	25	H	0	-1.919	2.606	0.204	H
HETATM	26	C	0	-2.294	-2.026	0.037	C
HETATM	27	C	0	-2.711	-2.177	-1.300	C
HETATM	28	C	0	-3.216	-1.681	1.047	C
HETATM	29	C	0	-4.048	-1.915	-1.619	C
HETATM	30	C	0	-4.543	-1.434	0.682	C
HETATM	31	C	0	-4.975	-1.538	-0.644	C
HETATM	32	H	0	-4.372	-2.018	-2.663	H
HETATM	33	H	0	-5.260	-1.151	1.464	H
HETATM	34	C	0	2.616	-2.170	0.203	C
HETATM	35	C	0	3.160	-2.215	-1.095	C
HETATM	36	C	0	3.417	-1.856	1.315	C
HETATM	37	C	0	4.518	-1.934	-1.266	C
HETATM	38	C	0	4.772	-1.575	1.101	C
HETATM	39	C	0	5.338	-1.607	-0.179	C
HETATM	40	H	0	4.945	-1.962	-2.278	H
HETATM	41	H	0	5.400	-1.316	1.963	H
HETATM	42	C	0	-1.785	-2.677	-2.367	C
HETATM	43	H	0	-2.009	-2.203	-3.337	H
HETATM	44	H	0	-0.727	-2.468	-2.145	H
HETATM	45	H	0	-1.894	-3.771	-2.508	H
HETATM	46	C	0	-2.811	-1.648	2.488	C
HETATM	47	H	0	-3.581	-1.157	3.106	H
HETATM	48	H	0	-2.679	-2.672	2.890	H
HETATM	49	H	0	-1.857	-1.118	2.645	H
HETATM	50	C	0	-6.397	-1.235	-1.005	C
HETATM	51	H	0	-6.574	-0.143	-1.041	H
HETATM	52	H	0	-6.670	-1.641	-1.994	H
HETATM	53	H	0	-7.107	-1.640	-0.262	H
HETATM	54	C	0	2.271	-2.481	-2.271	C
HETATM	55	H	0	2.852	-2.581	-3.203	H
HETATM	56	H	0	1.671	-3.402	-2.144	H
HETATM	57	H	0	1.544	-1.657	-2.415	H
HETATM	58	C	0	2.835	-1.799	2.693	C
HETATM	59	H	0	2.662	-2.810	3.111	H
HETATM	60	H	0	3.516	-1.281	3.389	H
HETATM	61	H	0	1.867	-1.267	2.711	H
HETATM	62	C	0	6.783	-1.265	-0.386	C

```
HETATM   63  H              0       7.401  -1.531   0.489                         H
HETATM   64  H              0       7.206  -1.773  -1.270                         H
HETATM   65  H              0       6.922  -0.179  -0.550                         H
HETATM   66  C              0      -1.516   4.231  -1.110                         C
HETATM   67  H              0      -2.545   4.620  -1.024                         H
HETATM   68  H              0      -0.848   5.095  -1.265                         H
HETATM   69  H              0      -1.469   3.585  -2.003                         H
HETATM   70  C              0      -1.215   4.180   1.442                         C
HETATM   71  H              0      -0.588   5.088   1.448                         H
HETATM   72  H              0      -2.252   4.495   1.652                         H
HETATM   73  H              0      -0.890   3.520   2.264                         H
HETATM   74  H              0      -0.978  -4.376  -0.314                         H
HETATM   75  H              0       1.363  -4.570   0.163                         H
HETATM   76  C              0      -4.338   1.796  -0.627                         C
HETATM   77  H              0      -5.086   2.171  -1.333                         H
HETATM   78  H              0      -3.462   1.262  -1.021                         H
HETATM   79  C              0      -4.527   1.994   0.674                         C
HETATM   80  F              0      -3.657   1.578   1.603                         F
HETATM   81  H              0      -5.383   2.509   1.129                         H
END
```

st3b.pdb

```
TITLE       st3b.pdb
HETATM    1 Ru              0      -0.332   0.323  -0.338                        Ru
HETATM    2 Cl              0      -0.851  -0.244  -2.620                        Cl
HETATM    3 Cl              0      -0.640   1.202   1.873                        Cl
HETATM    4  C              0      -0.618  -1.484   0.334                         C
HETATM    5  N              0      -1.867  -1.900   0.703                         N
HETATM    6  C              0      -1.904  -3.282   1.164                         C
HETATM    7  C              0      -0.430  -3.634   1.284                         C
HETATM    8  N              0       0.234  -2.515   0.613                         N
HETATM    9  C              0      -3.089  -1.214   0.421                         C
HETATM   10  C              0      -3.698  -1.377  -0.842                         C
HETATM   11  C              0      -4.877  -0.675  -1.108                         C
HETATM   12  C              0      -5.480   0.140  -0.145                         C
HETATM   13  C              0      -4.915   0.193   1.132                         C
HETATM   14  C              0      -3.734  -0.488   1.443                         C
HETATM   15  C              0      -3.187  -2.365  -1.847                         C
HETATM   16  C              0      -6.694   0.951  -0.479                         C
HETATM   17  C              0      -3.247  -0.508   2.861                         C
HETATM   18  C              0       1.649  -2.577   0.471                         C
HETATM   19  C              0       2.492  -2.038   1.459                         C
HETATM   20  C              0       3.877  -2.164   1.292                         C
HETATM   21  C              0       4.428  -2.805   0.179                         C
HETATM   22  C              0       3.563  -3.334  -0.789                         C
HETATM   23  C              0       2.175  -3.225  -0.663                         C
HETATM   24  C              0       1.932  -1.280   2.621                         C
HETATM   25  C              0       5.914  -2.879  -0.004                         C
HETATM   26  C              0       1.258  -3.688  -1.753                         C
HETATM   27  H              0      -2.438  -3.915   0.426                         H
HETATM   28  H              0      -2.451  -3.357   2.121                         H
HETATM   29  H              0      -0.165  -4.591   0.798                         H
HETATM   30  H              0      -0.088  -3.696   2.336                         H
HETATM   31  H              0      -5.340  -0.776  -2.099                         H
HETATM   32  H              0      -5.407   0.782   1.918                         H
HETATM   33  H              0      -2.110  -2.568  -1.746                         H
HETATM   34  H              0      -3.349  -2.011  -2.877                         H
HETATM   35  H              0      -3.729  -3.327  -1.743                         H
HETATM   36  H              0      -6.406   1.946  -0.871                         H
HETATM   37  H              0      -7.313   0.472  -1.258                         H
```

```
HETATM   38  H            0       -7.331   1.131   0.404                  H
HETATM   39  H            0       -2.200  -0.836   2.948                  H
HETATM   40  H            0       -3.313   0.490   3.324                  H
HETATM   41  H            0       -3.876  -1.188   3.470                  H
HETATM   42  H            0        4.542  -1.741   2.056                  H
HETATM   43  H            0        3.980  -3.829  -1.676                  H
HETATM   44  H            0        1.686  -0.240   2.332                  H
HETATM   45  H            0        2.649  -1.237   3.457                  H
HETATM   46  H            0        0.985  -1.706   2.995                  H
HETATM   47  H            0        6.291  -2.001  -0.566                  H
HETATM   48  H            0        6.451  -2.889   0.960                  H
HETATM   49  H            0        6.219  -3.772  -0.576                  H
HETATM   50  H            0        1.813  -4.191  -2.561                  H
HETATM   51  H            0        0.713  -2.831  -2.197                  H
HETATM   52  H            0        0.482  -4.390  -1.393                  H
HETATM   53  C            0        1.477   0.177  -0.695                  C
HETATM   54  C            0        2.559   1.136  -0.742                  C
HETATM   55  C            0        2.465   2.497  -0.330                  C
HETATM   56  C            0        3.608   3.309  -0.363                  C
HETATM   57  C            0        4.831   2.803  -0.799                  C
HETATM   58  C            0        4.942   1.473  -1.216                  C
HETATM   59  C            0        3.818   0.662  -1.188                  C
HETATM   60  O            0        1.246   2.930   0.029                  O
HETATM   61  C            0        1.036   3.946   1.043                  C
HETATM   62  C            0        1.030   5.344   0.457                  C
HETATM   63  C            0        1.929   3.761   2.251                  C
HETATM   64  H            0        1.819  -0.803  -1.071                  H
HETATM   65  H            0        3.540   4.359  -0.069                  H
HETATM   66  H            0        5.705   3.464  -0.822                  H
HETATM   67  H            0        5.903   1.079  -1.565                  H
HETATM   68  H            0        3.885  -0.388  -1.503                  H
HETATM   69  H            0        0.643   6.055   1.209                  H
HETATM   70  H            0        2.028   5.702   0.154                  H
HETATM   71  H            0        0.366   5.403  -0.423                  H
HETATM   72  H            0        0.011   3.698   1.368                  H
HETATM   73  H            0        2.977   4.062   2.083                  H
HETATM   74  H            0        1.911   2.707   2.577                  H
HETATM   75  H            0        1.540   4.373   3.083                  H
HETATM   76  C            0       -1.164   3.022  -2.010                  C
HETATM   77  C            0       -1.871   2.873  -0.891                  C
HETATM   78  H            0       -1.394   2.449  -2.913                  H
HETATM   79  H            0       -0.311   3.705  -2.019                  H
HETATM   80  F            0       -2.917   2.040  -0.813                  F
HETATM   81  H            0       -1.695   3.388   0.061                  H
END
```

st3c.pdb

```
TITLE      st3c.pdb
HETATM    1 Ru            0       -0.517   0.338  -0.628                 Ru
HETATM    2  C            0       -0.514  -3.390   1.716                  C
HETATM    3  C            0       -1.982  -2.982   1.700                  C
HETATM    4  H            0       -0.065  -3.356   2.727                  H
HETATM    5  H            0       -2.408  -2.844   2.711                  H
HETATM    6  C            0       -0.733  -1.403   0.475                  C
HETATM    7  N            0       -1.953  -1.712   0.978                  N
HETATM    8  N            0        0.121  -2.379   0.859                  N
HETATM    9 Cl            0       -0.505   1.433   1.580                 Cl
HETATM   10 Cl            0       -0.984  -0.961  -2.643                 Cl
HETATM   11  C            0        1.315   0.215  -0.901                  C
HETATM   12  H            0        1.579  -0.782  -1.310                  H
```

HETATM	13	C	0	2.504	1.030	-0.774	C
HETATM	14	C	0	2.587	2.390	-0.332	C
HETATM	15	C	0	3.720	0.367	-1.084	C
HETATM	16	C	0	3.850	2.990	-0.198	C
HETATM	17	C	0	4.960	0.969	-0.941	C
HETATM	18	H	0	3.654	-0.670	-1.436	H
HETATM	19	C	0	5.017	2.290	-0.489	C
HETATM	20	H	0	3.922	4.034	0.113	H
HETATM	21	H	0	5.877	0.421	-1.185	H
HETATM	22	H	0	5.984	2.795	-0.378	H
HETATM	23	O	0	1.439	3.042	-0.130	O
HETATM	24	C	0	1.283	4.160	0.776	C
HETATM	25	H	0	0.198	4.109	0.975	H
HETATM	26	C	0	-3.151	-0.999	0.664	C
HETATM	27	C	0	-3.852	-1.315	-0.521	C
HETATM	28	C	0	-3.660	-0.048	1.571	C
HETATM	29	C	0	-4.965	-0.541	-0.861	C
HETATM	30	C	0	-4.778	0.701	1.188	C
HETATM	31	C	0	-5.417	0.496	-0.038	C
HETATM	32	H	0	-5.495	-0.762	-1.797	H
HETATM	33	H	0	-5.161	1.466	1.877	H
HETATM	34	C	0	1.521	-2.491	0.607	C
HETATM	35	C	0	1.951	-3.301	-0.463	C
HETATM	36	C	0	2.444	-1.873	1.469	C
HETATM	37	C	0	3.324	-3.479	-0.655	C
HETATM	38	C	0	3.810	-2.078	1.241	C
HETATM	39	C	0	4.267	-2.875	0.188	C
HETATM	40	H	0	3.666	-4.104	-1.491	H
HETATM	41	H	0	4.536	-1.591	1.905	H
HETATM	42	C	0	-3.512	-2.520	-1.345	C
HETATM	43	H	0	-3.679	-2.335	-2.418	H
HETATM	44	H	0	-2.465	-2.841	-1.240	H
HETATM	45	H	0	-4.164	-3.370	-1.055	H
HETATM	46	C	0	-3.107	0.115	2.954	C
HETATM	47	H	0	-3.015	1.178	3.229	H
HETATM	48	H	0	-3.788	-0.356	3.690	H
HETATM	49	H	0	-2.108	-0.331	3.071	H
HETATM	50	C	0	-6.552	1.373	-0.470	C
HETATM	51	H	0	-6.186	2.207	-1.098	H
HETATM	52	H	0	-7.293	0.824	-1.076	H
HETATM	53	H	0	-7.077	1.826	0.389	H
HETATM	54	C	0	0.954	-3.903	-1.404	C
HETATM	55	H	0	1.442	-4.571	-2.133	H
HETATM	56	H	0	0.176	-4.488	-0.879	H
HETATM	57	H	0	0.415	-3.115	-1.967	H
HETATM	58	C	0	1.982	-0.973	2.570	C
HETATM	59	H	0	1.149	-1.406	3.154	H
HETATM	60	H	0	2.801	-0.737	3.269	H
HETATM	61	H	0	1.586	-0.021	2.168	H
HETATM	62	C	0	5.734	-3.042	-0.070	C
HETATM	63	H	0	6.343	-2.755	0.804	H
HETATM	64	H	0	5.992	-4.081	-0.340	H
HETATM	65	H	0	6.066	-2.410	-0.917	H
HETATM	66	C	0	1.576	5.478	0.085	C
HETATM	67	H	0	1.223	6.314	0.714	H
HETATM	68	H	0	2.650	5.644	-0.107	H
HETATM	69	H	0	1.045	5.540	-0.881	H
HETATM	70	C	0	1.976	3.958	2.107	C
HETATM	71	H	0	3.067	4.116	2.078	H
HETATM	72	H	0	1.561	4.677	2.836	H
HETATM	73	H	0	1.771	2.943	2.487	H
HETATM	74	H	0	-2.626	-3.707	1.164	H

```
HETATM   75  H              0       -0.333  -4.403   1.313                     H
HETATM   76  C              0       -0.524   2.075  -2.237                     C
HETATM   77  H              0        0.476   2.513  -2.268                     H
HETATM   78  H              0       -0.908   1.587  -3.139                     H
HETATM   79  C              0       -1.350   2.376  -1.190                     C
HETATM   80  H              0       -1.130   3.055  -0.361                     H
HETATM   81  F              0       -2.665   2.062  -1.261                     F
END
```

st3d.pdb

```
TITLE      st3d.pdb
HETATM    1 Ru              0        0.713  -0.557  -0.457                    Ru
HETATM    2  C              0        0.735   3.532   1.092                     C
HETATM    3  C              0        2.203   3.125   1.223                     C
HETATM    4  H              0        0.293   3.887   2.041                     H
HETATM    5  H              0        2.568   3.150   2.268                     H
HETATM    6  C              0        0.964   1.304   0.403                     C
HETATM    7  N              0        2.202   1.754   0.715                     N
HETATM    8  N              0        0.084   2.290   0.658                     N
HETATM    9 Cl              0        0.161  -1.618   1.682                    Cl
HETATM   10 Cl              0        1.718   0.422  -2.474                    Cl
HETATM   11  C              0       -0.844  -0.600  -1.542                     C
HETATM   12  H              0       -0.555  -0.309  -2.572                     H
HETATM   13  C              0       -2.281  -0.787  -1.488                     C
HETATM   14  C              0       -3.049  -1.495  -0.509                     C
HETATM   15  C              0       -2.986  -0.165  -2.550                     C
HETATM   16  C              0       -4.453  -1.425  -0.559                     C
HETATM   17  C              0       -4.371  -0.122  -2.602                     C
HETATM   18  H              0       -2.395   0.327  -3.332                     H
HETATM   19  C              0       -5.099  -0.741  -1.584                     C
HETATM   20  H              0       -5.055  -1.940   0.191                     H
HETATM   21  H              0       -4.881   0.390  -3.426                     H
HETATM   22  H              0       -6.196  -0.718  -1.597                     H
HETATM   23  O              0       -2.356  -2.261   0.325                     O
HETATM   24  C              0       -2.877  -2.894   1.512                     C
HETATM   25  H              0       -1.940  -3.284   1.945                     H
HETATM   26  C              0        3.362   0.924   0.624                     C
HETATM   27  C              0        4.243   1.049  -0.470                     C
HETATM   28  C              0        3.613  -0.009   1.652                     C
HETATM   29  C              0        5.259   0.101  -0.611                     C
HETATM   30  C              0        4.639  -0.944   1.465                     C
HETATM   31  C              0        5.438  -0.931   0.317                     C
HETATM   32  H              0        5.920   0.163  -1.485                     H
HETATM   33  H              0        4.825  -1.689   2.249                     H
HETATM   34  C              0       -1.335   2.190   0.559                     C
HETATM   35  C              0       -1.975   2.548  -0.639                     C
HETATM   36  C              0       -2.069   1.785   1.690                     C
HETATM   37  C              0       -3.373   2.500  -0.686                     C
HETATM   38  C              0       -3.463   1.731   1.592                     C
HETATM   39  C              0       -4.130   2.091   0.415                     C
HETATM   40  H              0       -3.882   2.767  -1.621                     H
HETATM   41  H              0       -4.043   1.408   2.467                     H
HETATM   42  C              0        4.153   2.199  -1.425                     C
HETATM   43  H              0        4.472   1.902  -2.438                     H
HETATM   44  H              0        3.130   2.594  -1.515                     H
HETATM   45  H              0        4.819   3.024  -1.099                     H
HETATM   46  C              0        2.855   0.036   2.944                     C
HETATM   47  H              0        2.658   1.074   3.268                     H
HETATM   48  H              0        1.876  -0.473   2.871                     H
HETATM   49  H              0        3.426  -0.463   3.746                     H
```

```
HETATM   50  C              0        6.464  -1.996   0.079                       C
HETATM   51  H              0        6.077  -2.763  -0.618                       H
HETATM   52  H              0        7.383  -1.592  -0.380                       H
HETATM   53  H              0        6.745  -2.519   1.009                       H
HETATM   54  C              0       -1.172   2.946  -1.839                       C
HETATM   55  H              0       -1.815   3.075  -2.726                       H
HETATM   56  H              0       -0.639   3.904  -1.685                       H
HETATM   57  H              0       -0.390   2.199  -2.084                       H
HETATM   58  C              0       -1.370   1.407   2.959                       C
HETATM   59  H              0       -0.677   2.197   3.309                       H
HETATM   60  H              0       -2.091   1.219   3.772                       H
HETATM   61  H              0       -0.763   0.491   2.822                       H
HETATM   62  C              0       -5.628   2.061   0.353                       C
HETATM   63  H              0       -6.042   1.187   0.886                       H
HETATM   64  H              0       -6.074   2.957   0.826                       H
HETATM   65  H              0       -5.995   2.029  -0.688                       H
HETATM   66  C              0       -3.772  -4.081   1.204                       C
HETATM   67  H              0       -3.880  -4.697   2.114                       H
HETATM   68  H              0       -4.788  -3.812   0.871                       H
HETATM   69  H              0       -3.316  -4.720   0.428                       H
HETATM   70  C              0       -3.435  -1.885   2.493                       C
HETATM   71  H              0       -4.389  -1.432   2.171                       H
HETATM   72  H              0       -3.613  -2.376   3.466                       H
HETATM   73  H              0       -2.699  -1.079   2.647                       H
HETATM   74  H              0        2.882   3.758   0.623                       H
HETATM   75  H              0        0.575   4.327   0.339                       H
HETATM   76  C              0        0.474  -2.701  -1.349                       C
HETATM   77  H              0       -0.215  -3.140  -0.621                       H
HETATM   78  H              0        0.235  -2.783  -2.417                       H
HETATM   79  C              0        1.781  -2.409  -0.984                       C
HETATM   80  H              0        2.179  -2.607   0.023                       H
HETATM   81  F              0        2.751  -2.339  -1.900                       F
END
```

st3I.pdb

```
TITLE       st3I.pdb
HETATM    1 Ru              0        0.516  -0.509  -0.352                      Ru
HETATM    2  C              0        1.257   3.530   0.928                       C
HETATM    3  C              0        2.661   2.944   1.080                       C
HETATM    4  H              0        0.856   3.944   1.870                       H
HETATM    5  H              0        3.022   2.942   2.126                       H
HETATM    6  C              0        1.220   1.280   0.303                       C
HETATM    7  N              0        2.499   1.566   0.600                       N
HETATM    8  N              0        0.462   2.372   0.508                       N
HETATM    9 Cl              0       -0.529  -0.911   1.807                      Cl
HETATM   10 Cl              0        1.665   0.010  -2.454                      Cl
HETATM   11  C              0       -0.887  -1.332  -1.590                       C
HETATM   12  H              0       -0.524  -1.099  -2.602                       H
HETATM   13  C              0       -2.341  -1.128  -1.518                       C
HETATM   14  C              0       -3.257  -1.683  -0.578                       C
HETATM   15  C              0       -2.869  -0.310  -2.540                       C
HETATM   16  C              0       -4.627  -1.399  -0.706                       C
HETATM   17  C              0       -4.222  -0.016  -2.643                       C
HETATM   18  H              0       -2.164   0.109  -3.270                       H
HETATM   19  C              0       -5.104  -0.569  -1.715                       C
HETATM   20  H              0       -5.339  -1.863  -0.019                       H
HETATM   21  H              0       -4.587   0.633  -3.447                       H
HETATM   22  H              0       -6.181  -0.370  -1.783                       H
HETATM   23  O              0       -2.766  -2.528   0.341                       O
HETATM   24  C              0       -3.364  -2.748   1.639                       C
```

```
HETATM   25  H            0      -2.480  -3.065   2.220                    H
HETATM   26  C            0       3.592   0.652   0.483                    C
HETATM   27  C            0       4.462   0.744  -0.618                    C
HETATM   28  C            0       3.790  -0.312   1.490                    C
HETATM   29  C            0       5.486  -0.200  -0.735                    C
HETATM   30  C            0       4.827  -1.237   1.333                    C
HETATM   31  C            0       5.670  -1.208   0.217                    C
HETATM   32  H            0       6.152  -0.152  -1.607                    H
HETATM   33  H            0       4.981  -1.997   2.110                    H
HETATM   34  C            0      -0.965   2.403   0.428                    C
HETATM   35  C            0      -1.583   2.560  -0.826                    C
HETATM   36  C            0      -1.724   2.308   1.611                    C
HETATM   37  C            0      -2.981   2.592  -0.879                    C
HETATM   38  C            0      -3.116   2.325   1.508                    C
HETATM   39  C            0      -3.762   2.458   0.272                    C
HETATM   40  H            0      -3.471   2.716  -1.853                    H
HETATM   41  H            0      -3.716   2.221   2.423                    H
HETATM   42  C            0       4.332   1.838  -1.634                    C
HETATM   43  H            0       4.634   1.485  -2.634                    H
HETATM   44  H            0       3.296   2.202  -1.725                    H
HETATM   45  H            0       4.984   2.698  -1.383                    H
HETATM   46  C            0       2.919  -0.346   2.708                    C
HETATM   47  H            0       2.798   0.657   3.160                    H
HETATM   48  H            0       1.894  -0.703   2.490                    H
HETATM   49  H            0       3.343  -1.009   3.480                    H
HETATM   50  C            0       6.735  -2.246   0.032                    C
HETATM   51  H            0       6.387  -3.056  -0.637                    H
HETATM   52  H            0       7.646  -1.827  -0.430                    H
HETATM   53  H            0       7.022  -2.720   0.986                    H
HETATM   54  C            0      -0.770   2.689  -2.077                    C
HETATM   55  H            0      -1.402   2.983  -2.932                    H
HETATM   56  H            0       0.033   3.443  -1.978                    H
HETATM   57  H            0      -0.253   1.747  -2.349                    H
HETATM   58  C            0      -1.070   2.210   2.956                    C
HETATM   59  H            0      -0.933   3.209   3.416                    H
HETATM   60  H            0      -1.689   1.618   3.651                    H
HETATM   61  H            0      -0.085   1.715   2.907                    H
HETATM   62  C            0      -5.259   2.451   0.198                    C
HETATM   63  H            0      -5.674   1.510   0.605                    H
HETATM   64  H            0      -5.705   3.272   0.789                    H
HETATM   65  H            0      -5.621   2.549  -0.840                    H
HETATM   66  C            0      -4.348  -3.902   1.600                    C
HETATM   67  H            0      -4.632  -4.186   2.628                    H
HETATM   68  H            0      -5.277  -3.659   1.056                    H
HETATM   69  H            0      -3.897  -4.787   1.120                    H
HETATM   70  C            0      -3.897  -1.488   2.297                    C
HETATM   71  H            0      -4.893  -1.178   1.936                    H
HETATM   72  H            0      -3.986  -1.667   3.383                    H
HETATM   73  H            0      -3.187  -0.657   2.152                    H
HETATM   74  H            0       3.418   3.469   0.470                    H
HETATM   75  H            0       1.199   4.327   0.164                    H
HETATM   76  C            0      -0.118  -2.552  -1.019                    C
HETATM   77  H            0      -0.772  -3.133  -0.355                    H
HETATM   78  H            0       0.228  -3.120  -1.899                    H
HETATM   79  C            0       1.254  -2.290  -0.257                    C
HETATM   80  H            0       1.284  -2.762   0.741                    H
HETATM   81  F            0       2.338  -2.688  -0.960                    F
END
```

st3e.pdb

```
TITLE      st3e.pdb
HETATM    1 Ru            0      -0.484  -0.655   0.289                     Ru
HETATM    2 Cl            0      -1.508  -0.196   2.475                     Cl
HETATM    3 Cl            0       0.496  -0.862  -1.938                     Cl
HETATM    4  C            0      -1.163   1.235  -0.191                      C
HETATM    5  N            0      -2.434   1.592  -0.444                      N
HETATM    6  C            0      -2.564   3.013  -0.787                      C
HETATM    7  C            0      -1.146   3.543  -0.589                      C
HETATM    8  N            0      -0.384   2.328  -0.286                      N
HETATM    9  C            0      -3.577   0.732  -0.394                      C
HETATM   10  C            0      -4.419   0.760   0.730                      C
HETATM   11  C            0      -5.516  -0.110   0.757                      C
HETATM   12  C            0      -5.793  -0.979  -0.301                      C
HETATM   13  C            0      -4.963  -0.947  -1.429                      C
HETATM   14  C            0      -3.859  -0.094  -1.502                      C
HETATM   15  C            0      -4.200   1.712   1.866                      C
HETATM   16  C            0      -6.940  -1.940  -0.225                      C
HETATM   17  C            0      -3.003  -0.051  -2.730                      C
HETATM   18  C            0       1.048   2.322  -0.297                      C
HETATM   19  C            0       1.723   2.321  -1.532                      C
HETATM   20  C            0       3.121   2.332  -1.521                      C
HETATM   21  C            0       3.849   2.362  -0.328                      C
HETATM   22  C            0       3.148   2.403   0.882                      C
HETATM   23  C            0       1.751   2.383   0.923                      C
HETATM   24  C            0       0.989   2.331  -2.839                      C
HETATM   25  C            0       5.347   2.316  -0.343                      C
HETATM   26  C            0       1.020   2.456   2.227                      C
HETATM   27  H            0      -3.306   3.497  -0.126                      H
HETATM   28  H            0      -2.930   3.122  -1.826                      H
HETATM   29  H            0      -1.061   4.262   0.248                      H
HETATM   30  H            0      -0.736   4.036  -1.489                      H
HETATM   31  H            0      -6.168  -0.109   1.640                      H
HETATM   32  H            0      -5.187  -1.599  -2.284                      H
HETATM   33  H            0      -4.395   1.223   2.834                      H
HETATM   34  H            0      -4.887   2.579   1.795                      H
HETATM   35  H            0      -3.166   2.089   1.909                      H
HETATM   36  H            0      -6.612  -2.925   0.160                      H
HETATM   37  H            0      -7.734  -1.584   0.453                      H
HETATM   38  H            0      -7.392  -2.124  -1.215                      H
HETATM   39  H            0      -1.957  -0.357  -2.535                      H
HETATM   40  H            0      -2.945   0.970  -3.155                      H
HETATM   41  H            0      -3.404  -0.711  -3.517                      H
HETATM   42  H            0       3.658   2.306  -2.480                      H
HETATM   43  H            0       3.703   2.451   1.827                      H
HETATM   44  H            0      -0.039   1.943  -2.752                      H
HETATM   45  H            0       1.503   1.697  -3.582                      H
HETATM   46  H            0       0.938   3.352  -3.266                      H
HETATM   47  H            0       5.771   2.841  -1.216                      H
HETATM   48  H            0       5.709   1.270  -0.392                      H
HETATM   49  H            0       5.783   2.758   0.570                      H
HETATM   50  H            0       0.356   3.341   2.275                      H
HETATM   51  H            0       1.724   2.521   3.074                      H
HETATM   52  H            0       0.362   1.582   2.399                      H
HETATM   53  C            0       0.809  -1.898   1.497                      C
HETATM   54  C            0       2.186  -1.389   1.529                      C
HETATM   55  C            0       3.209  -1.655   0.575                      C
HETATM   56  C            0       4.508  -1.180   0.819                      C
HETATM   57  C            0       4.802  -0.442   1.963                      C
HETATM   58  C            0       3.805  -0.160   2.898                      C
HETATM   59  C            0       2.520  -0.634   2.673                      C
HETATM   60  O            0       2.872  -2.432  -0.463                      O
HETATM   61  C            0       3.580  -2.480  -1.720                      C
```

```
HETATM   62  C             0       3.987  -1.120  -2.258                      C
HETATM   63  C             0       4.703  -3.499  -1.678                      C
HETATM   64  H             0       5.314  -1.420   0.122                      H
HETATM   65  H             0       5.829  -0.092   2.124                      H
HETATM   66  H             0       4.031   0.422   3.799                      H
HETATM   67  H             0       1.720  -0.432   3.397                      H
HETATM   68  H             0       2.786  -2.865  -2.383                      H
HETATM   69  H             0       3.182  -0.385  -2.091                      H
HETATM   70  H             0       4.141  -1.203  -3.349                      H
HETATM   71  H             0       4.926  -0.730  -1.830                      H
HETATM   72  H             0       5.550  -3.180  -1.046                      H
HETATM   73  H             0       5.097  -3.664  -2.696                      H
HETATM   74  H             0       4.342  -4.469  -1.297                      H
HETATM   75  H             0       0.348  -1.868   2.494                      H
HETATM   76  C             0       0.161  -2.812   0.595                      C
HETATM   77  C             0      -1.598  -2.071  -0.106                      C
HETATM   78  F             0      -2.591  -2.556   0.635                      F
HETATM   79  H             0      -0.475  -3.558   1.092                      H
HETATM   80  H             0       0.683  -3.155  -0.304                      H
HETATM   81  H             0      -1.629  -2.556  -1.106                      H
END
```

st3f.pdb

```
TITLE      st3f.pdb
HETATM    1 Ru             0      -0.471  -0.723   0.438                     Ru
HETATM    2  C             0      -1.150   3.432  -0.456                      C
HETATM    3  C             0      -2.602   2.991  -0.328                      C
HETATM    4  H             0      -0.897   3.793  -1.472                      H
HETATM    5  H             0      -3.216   3.239  -1.212                      H
HETATM    6  C             0      -1.209   1.115  -0.060                      C
HETATM    7  N             0      -2.492   1.537  -0.175                      N
HETATM    8  N             0      -0.407   2.203  -0.172                      N
HETATM    9 Cl             0       0.125  -1.187  -1.887                     Cl
HETATM   10 Cl             0      -0.857  -0.039   2.763                     Cl
HETATM   11  C             0       1.092  -2.160   1.628                      C
HETATM   12  C             0       2.367  -1.442   1.527                      C
HETATM   13  C             0       3.313  -1.604   0.474                      C
HETATM   14  C             0       2.712  -0.619   2.619                      C
HETATM   15  C             0       4.559  -0.963   0.574                      C
HETATM   16  C             0       3.943   0.019   2.700                      C
HETATM   17  H             0       1.967  -0.492   3.414                      H
HETATM   18  C             0       4.867  -0.164   1.672                      C
HETATM   19  H             0       5.314  -1.112  -0.202                      H
HETATM   20  H             0       4.183   0.652   3.562                      H
HETATM   21  H             0       5.851   0.320   1.719                      H
HETATM   22  O             0       2.960  -2.439  -0.513                      O
HETATM   23  C             0       3.583  -2.508  -1.810                      C
HETATM   24  H             0       2.819  -3.078  -2.368                      H
HETATM   25  C             0      -3.653   0.719  -0.309                      C
HETATM   26  C             0      -4.499   0.485   0.792                      C
HETATM   27  C             0      -3.959   0.204  -1.585                      C
HETATM   28  C             0      -5.627  -0.322   0.601                      C
HETATM   29  C             0      -5.098  -0.593  -1.731                      C
HETATM   30  C             0      -5.936  -0.877  -0.646                      C
HETATM   31  H             0      -6.282  -0.523   1.459                      H
HETATM   32  H             0      -5.335  -1.003  -2.722                      H
HETATM   33  C             0       1.016   2.198  -0.329                      C
HETATM   34  C             0       1.838   2.493   0.780                      C
HETATM   35  C             0       1.567   2.056  -1.620                      C
HETATM   36  C             0       3.220   2.571   0.582                      C
```

```
HETATM   37  C              0       2.956   2.122  -1.762                       C
HETATM   38  C              0       3.799   2.364  -0.674                       C
HETATM   39  H              0       3.862   2.800   1.443                       H
HETATM   40  H              0       3.390   1.991  -2.763                       H
HETATM   41  C              0      -4.204   1.079   2.132                       C
HETATM   42  H              0      -4.883   0.679   2.903                       H
HETATM   43  H              0      -3.165   0.874   2.451                       H
HETATM   44  H              0      -4.330   2.179   2.128                       H
HETATM   45  C              0      -3.053   0.469  -2.746                       C
HETATM   46  H              0      -2.830   1.546  -2.865                       H
HETATM   47  H              0      -2.075  -0.040  -2.620                       H
HETATM   48  H              0      -3.496   0.112  -3.690                       H
HETATM   49  C              0      -7.129  -1.770  -0.811                       C
HETATM   50  H              0      -6.879  -2.821  -0.573                       H
HETATM   51  H              0      -7.954  -1.483  -0.137                       H
HETATM   52  H              0      -7.511  -1.761  -1.845                       H
HETATM   53  C              0       1.251   2.770   2.128                       C
HETATM   54  H              0       2.044   2.911   2.881                       H
HETATM   55  H              0       0.635   3.690   2.125                       H
HETATM   56  H              0       0.591   1.953   2.475                       H
HETATM   57  C              0       0.708   1.932  -2.842                       C
HETATM   58  H              0       0.581   2.917  -3.333                       H
HETATM   59  H              0       1.168   1.260  -3.585                       H
HETATM   60  H              0      -0.290   1.524  -2.624                       H
HETATM   61  C              0       5.286   2.379  -0.847                       C
HETATM   62  H              0       5.701   1.353  -0.782                       H
HETATM   63  H              0       5.587   2.781  -1.831                       H
HETATM   64  H              0       5.789   2.973  -0.065                       H
HETATM   65  C              0       4.856  -3.334  -1.786                       C
HETATM   66  H              0       5.165  -3.568  -2.820                       H
HETATM   67  H              0       5.702  -2.819  -1.300                       H
HETATM   68  H              0       4.694  -4.291  -1.261                       H
HETATM   69  C              0       3.710  -1.158  -2.488                       C
HETATM   70  H              0       4.533  -0.537  -2.094                       H
HETATM   71  H              0       3.902  -1.312  -3.565                       H
HETATM   72  H              0       2.765  -0.600  -2.385                       H
HETATM   73  H              0      -3.106   3.426   0.556                       H
HETATM   74  H              0      -0.870   4.227   0.257                       H
HETATM   75  C              0       0.447  -2.985   0.740                       C
HETATM   76  H              0       0.831  -3.221  -0.254                       H
HETATM   77  C              0      -2.023  -1.630   0.308                       C
HETATM   78  H              0       0.654  -2.096   2.631                       H
HETATM   79  H              0      -0.401  -3.575   1.106                       H
HETATM   80  F              0      -2.617  -2.296   1.294                       F
HETATM   81  H              0      -2.594  -1.816  -0.628                       H
END
```

ac3a.pdb

```
TITLE       ac3a.pdb
HETATM    1 Ru              0       0.053   0.289   0.120                      Ru
HETATM    2  C              0       1.012  -3.902   0.265                       C
HETATM    3  C              0      -0.506  -3.916   0.241                       C
HETATM    4  H              0       1.433  -4.280   1.218                       H
HETATM    5  H              0      -0.956  -4.450   1.098                       H
HETATM    6  C              0       0.234  -1.669   0.140                       C
HETATM    7  N              0      -0.846  -2.498   0.293                       N
HETATM    8  N              0       1.328  -2.484   0.100                       N
HETATM    9 Cl              0      -0.424   0.397   2.440                      Cl
HETATM   10 Cl              0      -0.710   0.492  -2.120                      Cl
HETATM   11  C              0       1.814   0.823  -0.036                       C
```

HETATM	12	H	0	2.666	0.132	-0.118	H
HETATM	13	C	0	2.179	2.216	-0.095	C
HETATM	14	C	0	1.174	3.206	0.041	C
HETATM	15	C	0	3.515	2.623	-0.282	C
HETATM	16	C	0	1.504	4.560	-0.018	C
HETATM	17	C	0	3.846	3.973	-0.332	C
HETATM	18	H	0	4.287	1.850	-0.387	H
HETATM	19	C	0	2.839	4.932	-0.200	C
HETATM	20	H	0	0.738	5.333	0.068	H
HETATM	21	H	0	4.887	4.282	-0.476	H
HETATM	22	H	0	3.089	5.999	-0.245	H
HETATM	23	O	0	-0.063	2.681	0.201	O
HETATM	24	C	0	-1.213	3.430	0.692	C
HETATM	25	H	0	-1.919	2.603	0.870	H
HETATM	26	C	0	-2.228	-2.150	0.180	C
HETATM	27	C	0	-2.829	-2.132	-1.099	C
HETATM	28	C	0	-3.011	-2.024	1.347	C
HETATM	29	C	0	-4.200	-1.877	-1.186	C
HETATM	30	C	0	-4.380	-1.768	1.207	C
HETATM	31	C	0	-4.988	-1.675	-0.048	C
HETATM	32	H	0	-4.669	-1.840	-2.179	H
HETATM	33	H	0	-4.990	-1.652	2.113	H
HETATM	34	C	0	2.698	-2.126	-0.050	C
HETATM	35	C	0	3.231	-2.017	-1.347	C
HETATM	36	C	0	3.488	-1.900	1.091	C
HETATM	37	C	0	4.584	-1.688	-1.484	C
HETATM	38	C	0	4.836	-1.573	0.910	C
HETATM	39	C	0	5.399	-1.462	-0.368	C
HETATM	40	H	0	5.009	-1.595	-2.492	H
HETATM	41	H	0	5.460	-1.385	1.793	H
HETATM	42	C	0	-2.067	-2.472	-2.344	C
HETATM	43	H	0	-2.377	-1.837	-3.190	H
HETATM	44	H	0	-0.979	-2.343	-2.236	H
HETATM	45	H	0	-2.262	-3.522	-2.641	H
HETATM	46	C	0	-2.438	-2.247	2.713	C
HETATM	47	H	0	-2.769	-1.470	3.422	H
HETATM	48	H	0	-2.783	-3.219	3.119	H
HETATM	49	H	0	-1.339	-2.239	2.716	H
HETATM	50	C	0	-6.442	-1.339	-0.174	C
HETATM	51	H	0	-6.582	-0.248	-0.299	H
HETATM	52	H	0	-6.904	-1.818	-1.055	H
HETATM	53	H	0	-7.016	-1.634	0.720	H
HETATM	54	C	0	2.342	-2.175	-2.541	C
HETATM	55	H	0	2.916	-2.137	-3.481	H
HETATM	56	H	0	1.781	-3.128	-2.528	H
HETATM	57	H	0	1.581	-1.370	-2.578	H
HETATM	58	C	0	2.872	-1.924	2.456	C
HETATM	59	H	0	2.363	-2.880	2.676	H
HETATM	60	H	0	3.629	-1.763	3.241	H
HETATM	61	H	0	2.100	-1.138	2.562	H
HETATM	62	C	0	6.836	-1.070	-0.539	C
HETATM	63	H	0	7.454	-1.374	0.323	H
HETATM	64	H	0	7.279	-1.509	-1.449	H
HETATM	65	H	0	6.945	0.028	-0.634	H
HETATM	66	C	0	-1.794	4.319	-0.387	C
HETATM	67	H	0	-2.831	4.588	-0.123	H
HETATM	68	H	0	-1.236	5.260	-0.535	H
HETATM	69	H	0	-1.829	3.782	-1.350	H
HETATM	70	C	0	-0.935	4.098	2.020	C
HETATM	71	H	0	-0.270	4.974	1.948	H
HETATM	72	H	0	-1.891	4.448	2.448	H
HETATM	73	H	0	-0.494	3.374	2.725	H

```
HETATM   74  H             0       -0.917  -4.372  -0.682                       H
HETATM   75  H             0        1.472  -4.496  -0.547                       H
HETATM   76  C             0       -3.928   1.933  -1.295                       C
HETATM   77  C             0       -3.805   1.368  -0.099                       C
HETATM   78  H             0       -3.080   0.556   0.034                       H
HETATM   79  H             0       -4.407   1.685   0.762                       H
HETATM   80  H             0       -3.346   1.656  -2.182                       H
HETATM   81  F             0       -4.779   2.941  -1.532                       F
END
```

ac3b.pdb

```
TITLE       ac3b.pdb
HETATM    1 Ru             0       -0.561   0.333  -0.496                      Ru
HETATM    2  C             0       -0.682  -3.449   1.587                       C
HETATM    3  C             0       -2.145  -3.031   1.523                       C
HETATM    4  H             0       -0.275  -3.430   2.618                       H
HETATM    5  H             0       -2.635  -2.995   2.513                       H
HETATM    6  C             0       -0.828  -1.401   0.447                       C
HETATM    7  N             0       -2.064  -1.696   0.939                       N
HETATM    8  N             0       -0.011  -2.431   0.772                       N
HETATM    9 Cl             0       -0.703   1.401   1.693                      Cl
HETATM   10 Cl             0       -1.013  -0.746  -2.662                      Cl
HETATM   11  C             0        1.252   0.119  -0.851                       C
HETATM   12  H             0        1.474  -0.827  -1.387                       H
HETATM   13  C             0        2.466   0.891  -0.690                       C
HETATM   14  C             0        2.572   2.203  -0.126                       C
HETATM   15  C             0        3.664   0.249  -1.092                       C
HETATM   16  C             0        3.844   2.781   0.035                       C
HETATM   17  C             0        4.913   0.827  -0.924                       C
HETATM   18  H             0        3.577  -0.749  -1.540                       H
HETATM   19  C             0        4.994   2.098  -0.350                       C
HETATM   20  H             0        3.939   3.787   0.446                       H
HETATM   21  H             0        5.819   0.297  -1.239                       H
HETATM   22  H             0        5.969   2.581  -0.215                       H
HETATM   23  O             0        1.428   2.814   0.166                       O
HETATM   24  C             0        1.293   3.951   1.043                       C
HETATM   25  H             0        0.203   3.931   1.218                       H
HETATM   26  C             0       -3.240  -0.950   0.602                       C
HETATM   27  C             0       -3.882  -1.191  -0.634                       C
HETATM   28  C             0       -3.780  -0.035   1.529                       C
HETATM   29  C             0       -4.959  -0.375  -0.992                       C
HETATM   30  C             0       -4.864   0.755   1.128                       C
HETATM   31  C             0       -5.442   0.624  -0.138                       C
HETATM   32  H             0       -5.447  -0.541  -1.962                       H
HETATM   33  H             0       -5.273   1.487   1.837                       H
HETATM   34  C             0        1.394  -2.557   0.554                       C
HETATM   35  C             0        1.834  -3.344  -0.529                       C
HETATM   36  C             0        2.303  -1.963   1.448                       C
HETATM   37  C             0        3.208  -3.541  -0.692                       C
HETATM   38  C             0        3.670  -2.184   1.242                       C
HETATM   39  C             0        4.140  -2.970   0.187                       C
HETATM   40  H             0        3.561  -4.149  -1.535                       H
HETATM   41  H             0        4.390  -1.715   1.927                       H
HETATM   42  C             0       -3.526  -2.366  -1.493                       C
HETATM   43  H             0       -3.675  -2.144  -2.562                       H
HETATM   44  H             0       -2.480  -2.688  -1.380                       H
HETATM   45  H             0       -4.182  -3.224  -1.243                       H
HETATM   46  C             0       -3.298   0.038   2.945                       C
HETATM   47  H             0       -3.223   1.080   3.296                       H
HETATM   48  H             0       -4.015  -0.481   3.613                       H
```

```
HETATM   49  H              0       -2.306  -0.418   3.078                        H
HETATM   50  C              0       -6.560   1.525  -0.569                        C
HETATM   51  H              0       -6.173   2.411  -1.109                        H
HETATM   52  H              0       -7.258   1.019  -1.258                        H
HETATM   53  H              0       -7.139   1.905   0.289                        H
HETATM   54  C              0        0.848  -3.884  -1.517                        C
HETATM   55  H              0        1.336  -4.531  -2.264                        H
HETATM   56  H              0        0.043  -4.472  -1.037                        H
HETATM   57  H              0        0.340  -3.058  -2.056                        H
HETATM   58  C              0        1.826  -1.072   2.550                        C
HETATM   59  H              0        0.966  -1.496   3.099                        H
HETATM   60  H              0        2.629  -0.864   3.277                        H
HETATM   61  H              0        1.462  -0.104   2.153                        H
HETATM   62  C              0        5.609  -3.155  -0.042                        C
HETATM   63  H              0        6.204  -2.877   0.844                        H
HETATM   64  H              0        5.859  -4.196  -0.309                        H
HETATM   65  H              0        5.965  -2.525  -0.880                        H
HETATM   66  C              0        1.637   5.255   0.348                        C
HETATM   67  H              0        1.275   6.102   0.958                        H
HETATM   68  H              0        2.720   5.404   0.194                        H
HETATM   69  H              0        1.144   5.315  -0.636                        H
HETATM   70  C              0        1.951   3.739   2.391                        C
HETATM   71  H              0        3.050   3.831   2.376                        H
HETATM   72  H              0        1.569   4.493   3.101                        H
HETATM   73  H              0        1.684   2.744   2.786                        H
HETATM   74  H              0       -2.753  -3.696   0.876                        H
HETATM   75  H              0       -0.493  -4.459   1.182                        H
HETATM   76  C              0       -0.570   2.374  -2.283                        C
HETATM   77  C              0       -1.453   2.452  -1.262                        C
HETATM   78  H              0       -2.407   1.914  -1.360                        H
HETATM   79  H              0       -1.354   3.192  -0.461                        H
HETATM   80  H              0       -0.664   1.699  -3.141                        H
HETATM   81  F              0        0.489   3.171  -2.374                        F
END
```

ac3c.pdb

```
TITLE       ac3c.pdb
HETATM    1 Ru              0       -0.552   0.366  -0.494                       Ru
HETATM    2  C              0       -0.787  -3.401   1.616                        C
HETATM    3  C              0       -2.238  -2.942   1.553                        C
HETATM    4  H              0       -0.378  -3.394   2.645                        H
HETATM    5  H              0       -2.713  -2.861   2.548                        H
HETATM    6  C              0       -0.880  -1.359   0.453                        C
HETATM    7  N              0       -2.131  -1.628   0.926                        N
HETATM    8  N              0       -0.088  -2.404   0.799                        N
HETATM    9 Cl              0       -0.687   1.432   1.699                       Cl
HETATM   10 Cl              0       -1.068  -0.688  -2.655                       Cl
HETATM   11  C              0        1.251   0.078  -0.863                        C
HETATM   12  H              0        1.429  -0.879  -1.395                        H
HETATM   13  C              0        2.495   0.804  -0.723                        C
HETATM   14  C              0        2.663   2.099  -0.134                        C
HETATM   15  C              0        3.656   0.136  -1.187                        C
HETATM   16  C              0        3.957   2.634  -0.011                        C
HETATM   17  C              0        4.927   0.676  -1.064                        C
HETATM   18  H              0        3.523  -0.851  -1.649                        H
HETATM   19  C              0        5.069   1.931  -0.466                        C
HETATM   20  H              0        4.100   3.625   0.423                        H
HETATM   21  H              0        5.803   0.130  -1.432                        H
HETATM   22  H              0        6.063   2.382  -0.362                        H
HETATM   23  O              0        1.549   2.728   0.225                        O
```

```
HETATM   24  C            0       1.478   3.871   1.100                       C
HETATM   25  H            0       0.393   3.892   1.303                       H
HETATM   26  C            0      -3.291  -0.869   0.578                       C
HETATM   27  C            0      -3.940  -1.122  -0.651                       C
HETATM   28  C            0      -3.820   0.065   1.492                       C
HETATM   29  C            0      -5.016  -0.307  -1.017                       C
HETATM   30  C            0      -4.899   0.859   1.081                       C
HETATM   31  C            0      -5.486   0.711  -0.179                       C
HETATM   32  H            0      -5.509  -0.486  -1.982                       H
HETATM   33  H            0      -5.299   1.605   1.780                       H
HETATM   34  C            0       1.312  -2.562   0.584                       C
HETATM   35  C            0       1.737  -3.371  -0.488                       C
HETATM   36  C            0       2.236  -1.976   1.467                       C
HETATM   37  C            0       3.106  -3.595  -0.654                       C
HETATM   38  C            0       3.599  -2.220   1.260                       C
HETATM   39  C            0       4.050  -3.026   0.212                       C
HETATM   40  H            0       3.444  -4.221  -1.491                       H
HETATM   41  H            0       4.329  -1.755   1.936                       H
HETATM   42  C            0      -3.594  -2.311  -1.496                       C
HETATM   43  H            0      -3.746  -2.103  -2.567                       H
HETATM   44  H            0      -2.549  -2.637  -1.381                       H
HETATM   45  H            0      -4.253  -3.163  -1.231                       H
HETATM   46  C            0      -3.345   0.150   2.910                       C
HETATM   47  H            0      -3.260   1.196   3.247                       H
HETATM   48  H            0      -4.074  -0.351   3.579                       H
HETATM   49  H            0      -2.359  -0.314   3.058                       H
HETATM   50  C            0      -6.600   1.610  -0.619                       C
HETATM   51  H            0      -6.217   2.458  -1.219                       H
HETATM   52  H            0      -7.331   1.082  -1.256                       H
HETATM   53  H            0      -7.143   2.046   0.236                       H
HETATM   54  C            0       0.738  -3.906  -1.467                       C
HETATM   55  H            0       1.215  -4.564  -2.212                       H
HETATM   56  H            0      -0.071  -4.481  -0.979                       H
HETATM   57  H            0       0.238  -3.079  -2.011                       H
HETATM   58  C            0       1.779  -1.065   2.561                       C
HETATM   59  H            0       0.917  -1.471   3.121                       H
HETATM   60  H            0       2.589  -0.859   3.280                       H
HETATM   61  H            0       1.426  -0.097   2.153                       H
HETATM   62  C            0       5.515  -3.241  -0.019                       C
HETATM   63  H            0       6.120  -2.940   0.852                       H
HETATM   64  H            0       5.749  -4.296  -0.249                       H
HETATM   65  H            0       5.874  -2.648  -0.883                       H
HETATM   66  C            0       1.851   5.162   0.394                       C
HETATM   67  H            0       1.523   6.021   1.005                       H
HETATM   68  H            0       2.934   5.280   0.220                       H
HETATM   69  H            0       1.342   5.231  -0.582                       H
HETATM   70  C            0       2.164   3.633   2.431                       C
HETATM   71  H            0       3.265   3.647   2.378                       H
HETATM   72  H            0       1.859   4.420   3.142                       H
HETATM   73  H            0       1.844   2.663   2.848                       H
HETATM   74  H            0      -2.873  -3.608   0.935                       H
HETATM   75  H            0      -0.629  -4.416   1.210                       H
HETATM   76  C            0      -0.438   2.388  -2.250                       C
HETATM   77  C            0      -1.313   2.534  -1.229                       C
HETATM   78  H            0      -2.308   2.078  -1.324                       H
HETATM   79  H            0      -1.156   3.266  -0.429                       H
HETATM   80  H            0      -0.584   1.725  -3.110                       H
HETATM   81  F            0       0.677   3.106  -2.342                       F
END
```

ac3d.pdb

```
TITLE      ac3d.pdb
HETATM    1 Ru            0        0.627  -0.692  -0.392                   Ru
HETATM    2  C            0        1.046   3.335   1.187                    C
HETATM    3  C            0        2.445   2.795   1.452                    C
HETATM    4  H            0        0.564   3.767   2.081                    H
HETATM    5  H            0        2.666   2.681   2.532                    H
HETATM    6  C            0        1.155   1.116   0.457                    C
HETATM    7  N            0        2.405   1.477   0.814                    N
HETATM    8  N            0        0.322   2.139   0.735                    N
HETATM    9 Cl            0        0.270  -1.522   1.867                   Cl
HETATM   10 Cl            0        1.809   0.006  -2.440                   Cl
HETATM   11  C            0       -0.882  -0.723  -1.569                    C
HETATM   12  H            0       -0.572  -0.501  -2.609                    H
HETATM   13  C            0       -2.333  -0.735  -1.487                    C
HETATM   14  C            0       -3.133  -1.074  -0.354                    C
HETATM   15  C            0       -2.998  -0.327  -2.668                    C
HETATM   16  C            0       -4.533  -0.993  -0.457                    C
HETATM   17  C            0       -4.380  -0.242  -2.756                    C
HETATM   18  H            0       -2.381  -0.065  -3.537                    H
HETATM   19  C            0       -5.145  -0.583  -1.637                    C
HETATM   20  H            0       -5.153  -1.233   0.409                    H
HETATM   21  H            0       -4.859   0.080  -3.686                    H
HETATM   22  H            0       -6.240  -0.522  -1.677                    H
HETATM   23  O            0       -2.478  -1.430   0.743                    O
HETATM   24  C            0       -3.021  -2.239   1.807                    C
HETATM   25  H            0       -2.086  -2.554   2.301                    H
HETATM   26  C            0        3.589   0.725   0.538                    C
HETATM   27  C            0        4.344   1.061  -0.606                    C
HETATM   28  C            0        4.008  -0.301   1.405                    C
HETATM   29  C            0        5.464   0.289  -0.918                    C
HETATM   30  C            0        5.128  -1.062   1.042                    C
HETATM   31  C            0        5.853  -0.796  -0.121                    C
HETATM   32  H            0        6.039   0.531  -1.821                    H
HETATM   33  H            0        5.448  -1.877   1.706                    H
HETATM   34  C            0       -1.090   2.170   0.520                    C
HETATM   35  C            0       -1.599   2.510  -0.746                    C
HETATM   36  C            0       -1.946   1.932   1.612                    C
HETATM   37  C            0       -2.988   2.592  -0.904                    C
HETATM   38  C            0       -3.326   2.027   1.410                    C
HETATM   39  C            0       -3.864   2.355   0.160                    C
HETATM   40  H            0       -3.395   2.845  -1.892                    H
HETATM   41  H            0       -4.001   1.837   2.256                    H
HETATM   42  C            0        3.996   2.247  -1.454                    C
HETATM   43  H            0        4.309   2.091  -2.499                    H
HETATM   44  H            0        2.913   2.448  -1.471                    H
HETATM   45  H            0        4.513   3.160  -1.094                    H
HETATM   46  C            0        3.358  -0.552   2.732                    C
HETATM   47  H            0        2.435   0.028   2.882                    H
HETATM   48  H            0        3.081  -1.612   2.856                    H
HETATM   49  H            0        4.061  -0.304   3.551                    H
HETATM   50  C            0        7.017  -1.654  -0.516                    C
HETATM   51  H            0        6.714  -2.422  -1.253                    H
HETATM   52  H            0        7.822  -1.066  -0.991                    H
HETATM   53  H            0        7.447  -2.190   0.346                    H
HETATM   54  C            0       -0.681   2.772  -1.901                    C
HETATM   55  H            0       -1.251   2.974  -2.823                    H
HETATM   56  H            0       -0.030   3.649  -1.722                    H
HETATM   57  H            0        0.001   1.923  -2.105                    H
HETATM   58  C            0       -1.389   1.560   2.951                    C
HETATM   59  H            0       -0.598   2.255   3.289                    H
HETATM   60  H            0       -2.174   1.554   3.725                    H
HETATM   61  H            0       -0.924   0.555   2.917                    H
```

```
HETATM   62  C              0       -5.346   2.479  -0.021                       C
HETATM   63  H              0       -5.890   1.672   0.503                       H
HETATM   64  H              0       -5.728   3.434   0.388                       H
HETATM   65  H              0       -5.635   2.442  -1.085                       H
HETATM   66  C              0       -3.729  -3.485   1.310                       C
HETATM   67  H              0       -3.858  -4.182   2.156                       H
HETATM   68  H              0       -4.730  -3.298   0.887                       H
HETATM   69  H              0       -3.121  -3.998   0.547                       H
HETATM   70  C              0       -3.811  -1.415   2.806                       C
HETATM   71  H              0       -4.789  -1.071   2.426                       H
HETATM   72  H              0       -4.005  -2.021   3.709                       H
HETATM   73  H              0       -3.231  -0.531   3.117                       H
HETATM   74  H              0        3.244   3.415   1.010                       H
HETATM   75  H              0        1.025   4.108   0.393                       H
HETATM   76  C              0       -0.005  -2.643  -1.424                       C
HETATM   77  C              0        1.317  -2.614  -0.895                       C
HETATM   78  H              0        2.150  -2.512  -1.603                       H
HETATM   79  H              0        1.492  -3.191   0.023                       H
HETATM   80  F              0       -0.937  -3.382  -0.793                       F
HETATM   81  H              0       -0.148  -2.667  -2.513                       H
END
```

ac3I.pdb

```
TITLE       ac3I.pdb
HETATM    1 Ru              0       -0.477  -0.672   0.063                      Ru
HETATM    2  C              0       -1.340   3.474  -0.290                       C
HETATM    3  C              0       -2.735   2.937  -0.592                       C
HETATM    4  H              0       -0.945   4.134  -1.081                       H
HETATM    5  H              0       -3.035   3.085  -1.648                       H
HETATM    6  C              0       -1.314   1.144  -0.171                       C
HETATM    7  N              0       -2.603   1.501  -0.318                       N
HETATM    8  N              0       -0.542   2.248  -0.205                       N
HETATM    9 Cl              0        0.258  -0.581  -2.226                      Cl
HETATM   10 Cl              0       -1.847  -0.682   2.135                      Cl
HETATM   11  C              0        0.854  -1.550   1.338                       C
HETATM   12  H              0        0.380  -1.586   2.331                       H
HETATM   13  C              0        2.285  -1.253   1.494                       C
HETATM   14  C              0        3.236  -1.168   0.437                       C
HETATM   15  C              0        2.735  -1.004   2.807                       C
HETATM   16  C              0        4.582  -0.899   0.737                       C
HETATM   17  C              0        4.067  -0.731   3.095                       C
HETATM   18  H              0        2.000  -1.049   3.621                       H
HETATM   19  C              0        4.993  -0.688   2.050                       C
HETATM   20  H              0        5.309  -0.816  -0.075                       H
HETATM   21  H              0        4.384  -0.555   4.129                       H
HETATM   22  H              0        6.049  -0.471   2.253                       H
HETATM   23  O              0        2.739  -1.271  -0.790                       O
HETATM   24  C              0        3.439  -1.754  -1.948                       C
HETATM   25  H              0        2.593  -2.104  -2.566                       H
HETATM   26  C              0       -3.746   0.647  -0.252                       C
HETATM   27  C              0       -4.580   0.723   0.881                       C
HETATM   28  C              0       -4.052  -0.215  -1.319                       C
HETATM   29  C              0       -5.698  -0.110   0.945                       C
HETATM   30  C              0       -5.180  -1.039  -1.210                       C
HETATM   31  C              0       -6.006  -1.008  -0.084                       C
HETATM   32  H              0       -6.338  -0.070   1.836                       H
HETATM   33  H              0       -5.421  -1.716  -2.041                       H
HETATM   34  C              0        0.886   2.259  -0.118                       C
HETATM   35  C              0        1.511   2.095   1.134                       C
HETATM   36  C              0        1.642   2.504  -1.283                       C
```

```
HETATM   37  C             0       2.909   2.137   1.190                       C
HETATM   38  C             0       3.034   2.545  -1.177                       C
HETATM   39  C             0       3.684   2.359   0.049                       C
HETATM   40  H             0       3.403   1.989   2.159                       H
HETATM   41  H             0       3.628   2.726  -2.083                       H
HETATM   42  C             0      -4.292   1.672   2.005                       C
HETATM   43  H             0      -4.646   1.263   2.965                       H
HETATM   44  H             0      -3.211   1.864   2.116                       H
HETATM   45  H             0      -4.804   2.646   1.865                       H
HETATM   46  C             0      -3.212  -0.274  -2.557                       C
HETATM   47  H             0      -2.701   0.679  -2.774                       H
HETATM   48  H             0      -2.405  -1.024  -2.476                       H
HETATM   49  H             0      -3.824  -0.546  -3.434                       H
HETATM   50  C             0      -7.183  -1.928   0.033                       C
HETATM   51  H             0      -6.932  -2.826   0.629                       H
HETATM   52  H             0      -8.037  -1.445   0.540                       H
HETATM   53  H             0      -7.529  -2.285  -0.952                       H
HETATM   54  C             0       0.712   1.939   2.393                       C
HETATM   55  H             0       1.371   1.946   3.277                       H
HETATM   56  H             0      -0.022   2.758   2.517                       H
HETATM   57  H             0       0.120   1.004   2.424                       H
HETATM   58  C             0       0.983   2.754  -2.606                       C
HETATM   59  H             0       0.845   3.838  -2.790                       H
HETATM   60  H             0       1.600   2.366  -3.434                       H
HETATM   61  H             0      -0.001   2.265  -2.681                       H
HETATM   62  C             0       5.179   2.425   0.130                       C
HETATM   63  H             0       5.658   1.662  -0.512                       H
HETATM   64  H             0       5.562   3.404  -0.216                       H
HETATM   65  H             0       5.543   2.268   1.160                       H
HETATM   66  C             0       4.333  -2.946  -1.664                       C
HETATM   67  H             0       4.583  -3.442  -2.618                       H
HETATM   68  H             0       5.287  -2.687  -1.173                       H
HETATM   69  H             0       3.809  -3.684  -1.034                       H
HETATM   70  C             0       4.107  -0.615  -2.693                       C
HETATM   71  H             0       4.942  -0.163  -2.128                       H
HETATM   72  H             0       4.509  -0.977  -3.656                       H
HETATM   73  H             0       3.365   0.173  -2.906                       H
HETATM   74  H             0      -3.519   3.379   0.045                       H
HETATM   75  H             0      -1.287   4.028   0.668                       H
HETATM   76  C             0       0.283  -2.786   0.527                       C
HETATM   77  C             0      -1.090  -2.546  -0.151                       C
HETATM   78  H             0      -1.947  -2.859   0.465                       H
HETATM   79  H             0      -1.104  -2.921  -1.187                       H
HETATM   80  F             0       1.184  -3.370  -0.324                       F
HETATM   81  H             0       0.080  -3.507   1.342                       H
END
```

ac3e.pdb

```
TITLE       ac3e.pdb
HETATM    1 Ru             0      -0.558  -0.621   0.344                      Ru
HETATM    2 Cl             0      -1.606  -0.165   2.529                      Cl
HETATM    3 Cl             0       0.423  -0.926  -1.850                      Cl
HETATM    4  C             0      -1.243   1.216  -0.243                       C
HETATM    5  N             0      -2.526   1.542  -0.498                       N
HETATM    6  C             0      -2.679   2.938  -0.921                       C
HETATM    7  C             0      -1.268   3.498  -0.770                       C
HETATM    8  N             0      -0.482   2.312  -0.419                       N
HETATM    9  C             0      -3.651   0.664  -0.408                       C
HETATM   10  C             0      -4.495   0.728   0.714                       C
HETATM   11  C             0      -5.565  -0.172   0.789                       C
```

HETATM	12	C	0	-5.815	-1.106	-0.221	C
HETATM	13	C	0	-4.986	-1.109	-1.350	C
HETATM	14	C	0	-3.907	-0.227	-1.470	C
HETATM	15	C	0	-4.298	1.750	1.791	C
HETATM	16	C	0	-6.941	-2.088	-0.094	C
HETATM	17	C	0	-3.051	-0.227	-2.697	C
HETATM	18	C	0	0.948	2.354	-0.392	C
HETATM	19	C	0	1.658	2.336	-1.608	C
HETATM	20	C	0	3.052	2.425	-1.562	C
HETATM	21	C	0	3.743	2.548	-0.352	C
HETATM	22	C	0	3.007	2.594	0.836	C
HETATM	23	C	0	1.612	2.495	0.842	C
HETATM	24	C	0	0.958	2.246	-2.930	C
HETATM	25	C	0	5.240	2.604	-0.330	C
HETATM	26	C	0	0.846	2.568	2.127	C
HETATM	27	H	0	-3.421	3.450	-0.283	H
HETATM	28	H	0	-3.056	2.981	-1.961	H
HETATM	29	H	0	-1.183	4.258	0.031	H
HETATM	30	H	0	-0.877	3.953	-1.698	H
HETATM	31	H	0	-6.218	-0.142	1.671	H
HETATM	32	H	0	-5.190	-1.811	-2.169	H
HETATM	33	H	0	-3.250	2.078	1.869	H
HETATM	34	H	0	-4.581	1.347	2.778	H
HETATM	35	H	0	-4.932	2.642	1.614	H
HETATM	36	H	0	-6.624	-2.993	0.458	H
HETATM	37	H	0	-7.796	-1.666	0.461	H
HETATM	38	H	0	-7.305	-2.427	-1.078	H
HETATM	39	H	0	-2.023	-0.589	-2.498	H
HETATM	40	H	0	-2.936	0.789	-3.120	H
HETATM	41	H	0	-3.483	-0.867	-3.484	H
HETATM	42	H	0	3.616	2.390	-2.504	H
HETATM	43	H	0	3.532	2.714	1.792	H
HETATM	44	H	0	1.540	1.636	-3.641	H
HETATM	45	H	0	0.833	3.246	-3.392	H
HETATM	46	H	0	-0.036	1.775	-2.848	H
HETATM	47	H	0	5.678	1.593	-0.445	H
HETATM	48	H	0	5.645	3.218	-1.155	H
HETATM	49	H	0	5.625	3.012	0.620	H
HETATM	50	H	0	1.527	2.688	2.987	H
HETATM	51	H	0	0.230	1.667	2.311	H
HETATM	52	H	0	0.139	3.420	2.138	H
HETATM	53	C	0	0.788	-1.834	1.576	C
HETATM	54	C	0	2.160	-1.307	1.581	C
HETATM	55	C	0	3.188	-1.567	0.630	C
HETATM	56	C	0	4.463	-1.014	0.837	C
HETATM	57	C	0	4.733	-0.221	1.949	C
HETATM	58	C	0	3.733	0.050	2.883	C
HETATM	59	C	0	2.468	-0.488	2.689	C
HETATM	60	O	0	2.878	-2.401	-0.362	O
HETATM	61	C	0	3.544	-2.469	-1.637	C
HETATM	62	C	0	4.696	-3.455	-1.593	C
HETATM	63	C	0	3.902	-1.116	-2.226	C
HETATM	64	H	0	0.311	-1.693	2.556	H
HETATM	65	H	0	5.270	-1.244	0.137	H
HETATM	66	H	0	5.744	0.184	2.085	H
HETATM	67	H	0	3.938	0.676	3.759	H
HETATM	68	H	0	1.666	-0.283	3.410	H
HETATM	69	H	0	5.078	-3.633	-2.614	H
HETATM	70	H	0	4.370	-4.425	-1.182	H
HETATM	71	H	0	5.542	-3.093	-0.982	H
HETATM	72	H	0	2.739	-2.897	-2.261	H
HETATM	73	H	0	4.863	-0.712	-1.865	H

```
HETATM   74  H           0       3.101  -0.387  -2.019                       H
HETATM   75  H           0       3.985  -1.216  -3.323                       H
HETATM   76  C           0       0.056  -2.830   0.858                       C
HETATM   77  C           0      -1.759  -2.006   0.102                       C
HETATM   78  F           0       0.527  -3.548  -0.158                       F
HETATM   79  H           0      -0.631  -3.434   1.470                       H
HETATM   80  H           0      -1.782  -2.604  -0.833                       H
HETATM   81  H           0      -2.546  -2.238   0.849                       H
END
```

ac3f.pdb

```
TITLE      ac3f.pdb
HETATM    1 Ru           0      -0.539  -0.605   0.434                      Ru
HETATM    2 Cl           0      -1.201   0.013   2.715                      Cl
HETATM    3 Cl           0       0.284  -1.243  -1.742                      Cl
HETATM    4  C           0      -1.348   1.102  -0.192                       C
HETATM    5  N           0      -2.647   1.426  -0.407                       N
HETATM    6  C           0      -2.834   2.849  -0.697                       C
HETATM    7  C           0      -1.406   3.352  -0.858                       C
HETATM    8  N           0      -0.608   2.223  -0.382                       N
HETATM    9  C           0      -3.772   0.551  -0.359                       C
HETATM   10  C           0      -4.628   0.565   0.759                       C
HETATM   11  C           0      -5.733  -0.294   0.756                       C
HETATM   12  C           0      -6.006  -1.142  -0.322                       C
HETATM   13  C           0      -5.157  -1.106  -1.435                       C
HETATM   14  C           0      -4.043  -0.263  -1.480                       C
HETATM   15  C           0      -4.374   1.473   1.921                       C
HETATM   16  C           0      -7.172  -2.083  -0.283                       C
HETATM   17  C           0      -3.159  -0.211  -2.686                       C
HETATM   18  C           0       0.819   2.273  -0.486                       C
HETATM   19  C           0       1.427   2.095  -1.745                       C
HETATM   20  C           0       2.817   2.225  -1.834                       C
HETATM   21  C           0       3.600   2.553  -0.723                       C
HETATM   22  C           0       2.963   2.766   0.504                       C
HETATM   23  C           0       1.579   2.629   0.647                       C
HETATM   24  C           0       0.633   1.828  -2.987                       C
HETATM   25  C           0       5.090   2.648  -0.838                       C
HETATM   26  C           0       0.920   2.878   1.969                       C
HETATM   27  H           0      -3.367   3.339   0.140                       H
HETATM   28  H           0      -3.451   2.978  -1.604                       H
HETATM   29  H           0      -1.189   4.258  -0.263                       H
HETATM   30  H           0      -1.147   3.579  -1.912                       H
HETATM   31  H           0      -6.397  -0.302   1.631                       H
HETATM   32  H           0      -5.372  -1.747  -2.301                       H
HETATM   33  H           0      -3.295   1.623   2.096                       H
HETATM   34  H           0      -4.803   1.060   2.849                       H
HETATM   35  H           0      -4.841   2.467   1.771                       H
HETATM   36  H           0      -6.867  -3.086   0.074                       H
HETATM   37  H           0      -7.964  -1.731   0.399                       H
HETATM   38  H           0      -7.619  -2.228  -1.281                       H
HETATM   39  H           0      -2.130  -0.560  -2.466                       H
HETATM   40  H           0      -3.058   0.820  -3.075                       H
HETATM   41  H           0      -3.557  -0.838  -3.501                       H
HETATM   42  H           0       3.300   2.070  -2.808                       H
HETATM   43  H           0       3.559   3.050   1.380                       H
HETATM   44  H           0       1.159   1.115  -3.644                       H
HETATM   45  H           0      -0.355   1.388  -2.778                       H
HETATM   46  H           0       0.483   2.757  -3.573                       H
HETATM   47  H           0       5.561   1.656  -0.692                       H
HETATM   48  H           0       5.409   3.008  -1.832                       H
```

```
HETATM   49  H              0        5.522   3.319  -0.075                      H
HETATM   50  H              0        1.649   3.236   2.714                      H
HETATM   51  H              0        0.437   1.968   2.374                      H
HETATM   52  H              0        0.114   3.632   1.894                      H
HETATM   53  C              0        1.120  -2.011   1.717                      C
HETATM   54  C              0        2.371  -1.252   1.647                      C
HETATM   55  C              0        3.395  -1.435   0.677                      C
HETATM   56  C              0        4.581  -0.690   0.786                      C
HETATM   57  C              0        4.755   0.228   1.819                      C
HETATM   58  C              0        3.751   0.430   2.766                      C
HETATM   59  C              0        2.575  -0.304   2.671                      C
HETATM   60  O              0        3.172  -2.383  -0.236                      O
HETATM   61  C              0        3.778  -2.445  -1.540                      C
HETATM   62  C              0        5.088  -3.209  -1.499                      C
HETATM   63  C              0        3.852  -1.104  -2.243                      C
HETATM   64  H              0        0.548  -1.786   2.626                      H
HETATM   65  H              0        5.391  -0.856   0.073                      H
HETATM   66  H              0        5.695   0.791   1.880                      H
HETATM   67  H              0        3.882   1.161   3.573                      H
HETATM   68  H              0        1.771  -0.155   3.403                      H
HETATM   69  H              0        5.434  -3.420  -2.526                      H
HETATM   70  H              0        4.964  -4.176  -0.981                      H
HETATM   71  H              0        5.891  -2.650  -0.988                      H
HETATM   72  H              0        3.032  -3.057  -2.079                      H
HETATM   73  H              0        4.707  -0.479  -1.931                      H
HETATM   74  H              0        2.918  -0.542  -2.074                      H
HETATM   75  H              0        3.952  -1.272  -3.330                      H
HETATM   76  C              0        0.517  -3.039   1.049                      C
HETATM   77  C              0       -2.041  -1.614   0.297                      C
HETATM   78  H              0       -2.823  -1.644   1.086                      H
HETATM   79  H              0       -2.190  -2.312  -0.558                      H
HETATM   80  H              0       -0.398  -3.481   1.461                      H
HETATM   81  F              0        0.933  -3.704  -0.007                      F
END
```

at3a.pdb

```
TITLE      at3a.pdb
HETATM    1 Ru              0       -0.019   0.361   0.139                     Ru
HETATM    2  C              0        0.268  -3.886  -0.482                      C
HETATM    3  C              0       -1.236  -3.660  -0.448                      C
HETATM    4  H              0        0.620  -4.600   0.286                      H
HETATM    5  H              0       -1.739  -4.210   0.371                      H
HETATM    6  C              0       -0.148  -1.589  -0.081                      C
HETATM    7  N              0       -1.349  -2.221  -0.228                      N
HETATM    8  N              0        0.810  -2.553  -0.219                      N
HETATM    9 Cl              0       -0.726   0.360   2.424                     Cl
HETATM   10 Cl              0       -0.189   0.748  -2.207                     Cl
HETATM   11  C              0        1.804   0.569   0.288                      C
HETATM   12  H              0        2.531  -0.198   0.600                      H
HETATM   13  C              0        2.375   1.838  -0.109                      C
HETATM   14  C              0        1.530   2.965  -0.206                      C
HETATM   15  C              0        3.723   1.965  -0.493                      C
HETATM   16  C              0        2.001   4.169  -0.721                      C
HETATM   17  C              0        4.201   3.173  -0.994                      C
HETATM   18  H              0        4.380   1.089  -0.412                      H
HETATM   19  C              0        3.339   4.266  -1.117                      C
HETATM   20  H              0        1.326   5.022  -0.833                      H
HETATM   21  H              0        5.248   3.264  -1.304                      H
HETATM   22  H              0        3.709   5.212  -1.529                      H
HETATM   23  O              0        0.243   2.782   0.249                      O
```

```
HETATM   24  C              0       -0.144   3.518   1.468                       C
HETATM   25  H              0       -0.978   2.903   1.836                       H
HETATM   26  C              0       -2.660  -1.669  -0.102                       C
HETATM   27  C              0       -3.369  -1.283  -1.258                       C
HETATM   28  C              0       -3.299  -1.707   1.157                       C
HETATM   29  C              0       -4.697  -0.861  -1.117                       C
HETATM   30  C              0       -4.622  -1.264   1.249                       C
HETATM   31  C              0       -5.334  -0.832   0.125                       C
HETATM   32  H              0       -5.245  -0.544  -2.014                       H
HETATM   33  H              0       -5.116  -1.280   2.230                       H
HETATM   34  C              0        2.220  -2.421  -0.090                       C
HETATM   35  C              0        3.006  -2.177  -1.230                       C
HETATM   36  C              0        2.801  -2.554   1.183                       C
HETATM   37  C              0        4.392  -2.070  -1.072                       C
HETATM   38  C              0        4.191  -2.441   1.300                       C
HETATM   39  C              0        5.000  -2.196   0.184                       C
HETATM   40  H              0        5.012  -1.872  -1.956                       H
HETATM   41  H              0        4.651  -2.535   2.292                       H
HETATM   42  C              0       -2.784  -1.377  -2.634                       C
HETATM   43  H              0       -2.844  -0.407  -3.155                       H
HETATM   44  H              0       -1.723  -1.669  -2.632                       H
HETATM   45  H              0       -3.349  -2.109  -3.244                       H
HETATM   46  C              0       -2.643  -2.301   2.367                       C
HETATM   47  H              0       -2.831  -1.689   3.265                       H
HETATM   48  H              0       -3.052  -3.311   2.570                       H
HETATM   49  H              0       -1.551  -2.388   2.267                       H
HETATM   50  C              0       -6.737  -0.321   0.256                       C
HETATM   51  H              0       -6.747   0.761   0.493                       H
HETATM   52  H              0       -7.311  -0.445  -0.678                       H
HETATM   53  H              0       -7.288  -0.825   1.068                       H
HETATM   54  C              0        2.363  -1.974  -2.566                       C
HETATM   55  H              0        3.117  -1.832  -3.357                       H
HETATM   56  H              0        1.727  -2.829  -2.862                       H
HETATM   57  H              0        1.699  -1.088  -2.562                       H
HETATM   58  C              0        1.931  -2.712   2.392                       C
HETATM   59  H              0        1.218  -3.552   2.295                       H
HETATM   60  H              0        2.529  -2.883   3.302                       H
HETATM   61  H              0        1.314  -1.806   2.556                       H
HETATM   62  C              0        6.484  -2.038   0.329                       C
HETATM   63  H              0        6.851  -2.443   1.287                       H
HETATM   64  H              0        7.034  -2.539  -0.486                       H
HETATM   65  H              0        6.781  -0.972   0.296                       H
HETATM   66  C              0       -0.645   4.906   1.135                       C
HETATM   67  H              0       -1.176   5.322   2.009                       H
HETATM   68  H              0        0.172   5.607   0.890                       H
HETATM   69  H              0       -1.357   4.884   0.292                       H
HETATM   70  C              0        0.948   3.483   2.510                       C
HETATM   71  H              0        1.827   4.089   2.223                       H
HETATM   72  H              0        0.556   3.891   3.458                       H
HETATM   73  H              0        1.268   2.446   2.704                       H
HETATM   74  H              0       -1.742  -3.942  -1.391                       H
HETATM   75  H              0        0.629  -4.259  -1.460                       H
HETATM   76  C              0       -2.867   2.362  -1.069                       C
HETATM   77  C              0       -3.259   2.065   0.168                       C
HETATM   78  H              0       -4.152   1.464   0.365                       H
HETATM   79  H              0       -2.662   2.400   1.019                       H
HETATM   80  F              0       -3.549   1.968  -2.149                       F
HETATM   81  H              0       -1.966   2.926  -1.335                       H
END
```

at3b.pdb

```
TITLE      at3b.pdb
HETATM    1 Ru          0       -0.421   0.246  -0.235               Ru
HETATM    2  C          0       -0.256  -3.791   1.187                C
HETATM    3  C          0       -1.745  -3.461   1.184                C
HETATM    4  H          0        0.148  -3.940   2.207                H
HETATM    5  H          0       -2.218  -3.581   2.176                H
HETATM    6  C          0       -0.536  -1.597   0.379                C
HETATM    7  N          0       -1.762  -2.065   0.770                N
HETATM    8  N          0        0.355  -2.607   0.578                N
HETATM    9 Cl          0       -0.553   1.035   2.048               Cl
HETATM   10 Cl          0       -0.868  -0.341  -2.536               Cl
HETATM   11  C          0        1.386   0.262  -0.630                C
HETATM   12  H          0        1.787  -0.658  -1.092                H
HETATM   13  C          0        2.405   1.291  -0.592                C
HETATM   14  C          0        2.209   2.641  -0.175                C
HETATM   15  C          0        3.708   0.903  -0.992                C
HETATM   16  C          0        3.301   3.521  -0.161                C
HETATM   17  C          0        4.783   1.777  -0.956                C
HETATM   18  H          0        3.848  -0.133  -1.328                H
HETATM   19  C          0        4.572   3.094  -0.537                C
HETATM   20  H          0        3.153   4.565   0.125                H
HETATM   21  H          0        5.780   1.445  -1.266                H
HETATM   22  H          0        5.403   3.808  -0.518                H
HETATM   23  O          0        0.952   3.014   0.122                O
HETATM   24  C          0        0.650   4.012   1.136                C
HETATM   25  H          0       -0.361   3.694   1.445                H
HETATM   26  C          0       -2.983  -1.367   0.516                C
HETATM   27  C          0       -3.599  -1.489  -0.750                C
HETATM   28  C          0       -3.597  -0.623   1.546                C
HETATM   29  C          0       -4.738  -0.722  -1.014                C
HETATM   30  C          0       -4.738   0.127   1.236                C
HETATM   31  C          0       -5.300   0.118  -0.045                C
HETATM   32  H          0       -5.201  -0.790  -2.007                H
HETATM   33  H          0       -5.204   0.729   2.027                H
HETATM   34  C          0        1.764  -2.588   0.372                C
HETATM   35  C          0        2.272  -3.111  -0.831                C
HETATM   36  C          0        2.618  -2.072   1.364                C
HETATM   37  C          0        3.657  -3.108  -1.029                C
HETATM   38  C          0        3.997  -2.084   1.124                C
HETATM   39  C          0        4.532  -2.592  -0.065                C
HETATM   40  H          0        4.060  -3.503  -1.971                H
HETATM   41  H          0        4.671  -1.673   1.887                H
HETATM   42  C          0       -3.133  -2.493  -1.759                C
HETATM   43  H          0       -3.322  -2.147  -2.787                H
HETATM   44  H          0       -2.056  -2.708  -1.688                H
HETATM   45  H          0       -3.684  -3.446  -1.625                H
HETATM   46  C          0       -3.127  -0.699   2.966                C
HETATM   47  H          0       -3.164   0.286   3.459                H
HETATM   48  H          0       -3.786  -1.377   3.545                H
HETATM   49  H          0       -2.093  -1.065   3.054                H
HETATM   50  C          0       -6.473   0.990  -0.374                C
HETATM   51  H          0       -6.140   1.966  -0.777                H
HETATM   52  H          0       -7.123   0.538  -1.143                H
HETATM   53  H          0       -7.090   1.208   0.514                H
HETATM   54  C          0        1.335  -3.559  -1.909                C
HETATM   55  H          0        1.880  -3.999  -2.761                H
HETATM   56  H          0        0.603  -4.308  -1.555                H
HETATM   57  H          0        0.740  -2.705  -2.290                H
HETATM   58  C          0        2.066  -1.463   2.613                C
HETATM   59  H          0        1.245  -2.060   3.050                H
HETATM   60  H          0        2.847  -1.343   3.382                H
HETATM   61  H          0        1.629  -0.465   2.414                H
```

```
HETATM   62  C              0        6.007  -2.529  -0.321                      C
HETATM   63  H              0        6.596  -2.758   0.585                      H
HETATM   64  H              0        6.321  -3.224  -1.118                      H
HETATM   65  H              0        6.310  -1.512  -0.641                      H
HETATM   66  C              0        0.555   5.403   0.540                      C
HETATM   67  H              0        0.124   6.093   1.286                      H
HETATM   68  H              0        1.531   5.817   0.236                      H
HETATM   69  H              0       -0.109   5.415  -0.341                      H
HETATM   70  C              0        1.532   3.902   2.362                      C
HETATM   71  H              0        2.546   4.312   2.222                      H
HETATM   72  H              0        1.061   4.461   3.189                      H
HETATM   73  H              0        1.612   2.848   2.676                      H
HETATM   74  H              0       -2.319  -4.081   0.466                      H
HETATM   75  H              0       -0.007  -4.697   0.604                      H
HETATM   76  C              0       -1.472   2.849  -1.844                      C
HETATM   77  H              0       -0.556   3.453  -1.828                      H
HETATM   78  C              0       -2.096   2.371  -0.760                      C
HETATM   79  H              0       -3.008   1.767  -0.857                      H
HETATM   80  F              0       -1.902   2.624  -3.076                      F
HETATM   81  H              0       -1.752   2.648   0.241                      H
END
```

at3c.pdb

```
TITLE       at3c.pdb
HETATM    1 Ru              0        0.583   0.318   0.459                     Ru
HETATM    2  C              0        0.495  -3.521  -1.524                      C
HETATM    3  C              0        1.963  -3.113  -1.570                      C
HETATM    4  H              0        0.031  -3.572  -2.528                      H
HETATM    5  H              0        2.373  -3.076  -2.596                      H
HETATM    6  C              0        0.727  -1.435  -0.465                      C
HETATM    7  N              0        1.943  -1.781  -0.976                      N
HETATM    8  N              0       -0.130  -2.448  -0.742                      N
HETATM    9 Cl              0        0.557   1.390  -1.741                     Cl
HETATM   10 Cl              0        1.067  -0.725   2.596                     Cl
HETATM   11  C              0       -1.233   0.226   0.844                      C
HETATM   12  H              0       -1.496  -0.707   1.383                      H
HETATM   13  C              0       -2.424   1.036   0.660                      C
HETATM   14  C              0       -2.500   2.374   0.160                      C
HETATM   15  C              0       -3.645   0.390   0.981                      C
HETATM   16  C              0       -3.758   2.967  -0.033                      C
HETATM   17  C              0       -4.882   0.980   0.773                      C
HETATM   18  H              0       -3.586  -0.629   1.386                      H
HETATM   19  C              0       -4.932   2.276   0.256                      C
HETATM   20  H              0       -3.824   3.998  -0.384                      H
HETATM   21  H              0       -5.804   0.440   1.019                      H
HETATM   22  H              0       -5.897   2.772   0.096                      H
HETATM   23  O              0       -1.345   3.026  -0.018                      O
HETATM   24  C              0       -1.163   4.137  -0.927                      C
HETATM   25  H              0       -0.075   4.078  -1.103                      H
HETATM   26  C              0        3.142  -1.056  -0.692                      C
HETATM   27  C              0        3.832  -1.311   0.514                      C
HETATM   28  C              0        3.658  -0.147  -1.639                      C
HETATM   29  C              0        4.944  -0.523   0.825                      C
HETATM   30  C              0        4.775   0.619  -1.284                      C
HETATM   31  C              0        5.408   0.469  -0.047                      C
HETATM   32  H              0        5.469  -0.701   1.773                      H
HETATM   33  H              0        5.166   1.347  -2.008                      H
HETATM   34  C              0       -1.532  -2.520  -0.496                      C
HETATM   35  C              0       -1.983  -3.242   0.627                      C
HETATM   36  C              0       -2.435  -1.953  -1.411                      C
```

```
HETATM   37  C              0       -3.360  -3.400   0.807                       C
HETATM   38  C              0       -3.807  -2.137  -1.193                       C
HETATM   39  C              0       -4.285  -2.859  -0.096                       C
HETATM   40  H              0       -3.720  -3.957   1.683                       H
HETATM   41  H              0       -4.519  -1.692  -1.900                       H
HETATM   42  C              0        3.482  -2.473   1.394                       C
HETATM   43  H              0        3.660  -2.242   2.456                       H
HETATM   44  H              0        2.429  -2.780   1.310                       H
HETATM   45  H              0        4.118  -3.344   1.134                       H
HETATM   46  C              0        3.117  -0.061  -3.033                       C
HETATM   47  H              0        3.047   0.984  -3.377                       H
HETATM   48  H              0        3.796  -0.591  -3.731                       H
HETATM   49  H              0        2.112  -0.498  -3.128                       H
HETATM   50  C              0        6.563   1.344   0.337                       C
HETATM   51  H              0        6.225   2.207   0.942                       H
HETATM   52  H              0        7.305   0.804   0.950                       H
HETATM   53  H              0        7.081   1.758  -0.545                       H
HETATM   54  C              0       -1.002  -3.764   1.630                       C
HETATM   55  H              0       -1.511  -4.310   2.442                       H
HETATM   56  H              0       -0.260  -4.451   1.181                       H
HETATM   57  H              0       -0.420  -2.937   2.084                       H
HETATM   58  C              0       -1.945  -1.130  -2.560                       C
HETATM   59  H              0       -1.112  -1.613  -3.104                       H
HETATM   60  H              0       -2.751  -0.926  -3.284                       H
HETATM   61  H              0       -1.539  -0.160  -2.214                       H
HETATM   62  C              0       -5.756  -3.014   0.145                       C
HETATM   63  H              0       -6.355  -2.686  -0.721                       H
HETATM   64  H              0       -6.031  -4.060   0.371                       H
HETATM   65  H              0       -6.086  -2.414   1.015                       H
HETATM   66  C              0       -1.464   5.459  -0.246                       C
HETATM   67  H              0       -1.107   6.292  -0.876                       H
HETATM   68  H              0       -2.540   5.622  -0.065                       H
HETATM   69  H              0       -0.942   5.528   0.725                       H
HETATM   70  C              0       -1.830   3.941  -2.274                       C
HETATM   71  H              0       -2.914   4.145  -2.276                       H
HETATM   72  H              0       -1.368   4.631  -3.001                       H
HETATM   73  H              0       -1.657   2.914  -2.635                       H
HETATM   74  H              0        2.614  -3.786  -0.977                       H
HETATM   75  H              0        0.327  -4.500  -1.038                       H
HETATM   76  C              0        0.661   2.492   2.159                       C
HETATM   77  H              0       -0.362   2.865   2.047                       H
HETATM   78  C              0        1.576   2.435   1.166                       C
HETATM   79  H              0        2.610   2.138   1.389                       H
HETATM   80  F              0        0.948   2.158   3.404                       F
HETATM   81  H              0        1.352   2.882   0.193                       H
END
```

at3d.pdb

```
TITLE       at3d.pdb
HETATM    1 Ru              0        0.648  -0.715  -0.192                      Ru
HETATM    2  C              0        1.079   3.582   0.459                       C
HETATM    3  C              0        2.503   3.098   0.721                       C
HETATM    4  H              0        0.634   4.110   1.321                       H
HETATM    5  H              0        2.829   3.251   1.767                       H
HETATM    6  C              0        1.163   1.262   0.145                       C
HETATM    7  N              0        2.420   1.665   0.423                       N
HETATM    8  N              0        0.351   2.335   0.199                       N
HETATM    9 Cl              0       -0.275  -0.691   2.068                      Cl
HETATM   10 Cl              0        1.947  -0.709  -2.265                      Cl
HETATM   11  C              0       -0.823  -1.182  -1.294                       C
```

HETATM	12	H	0	-0.438	-1.352	-2.319	H
HETATM	13	C	0	-2.278	-1.161	-1.307	C
HETATM	14	C	0	-3.177	-1.458	-0.240	C
HETATM	15	C	0	-2.833	-0.794	-2.556	C
HETATM	16	C	0	-4.557	-1.277	-0.437	C
HETATM	17	C	0	-4.196	-0.616	-2.739	C
HETATM	18	H	0	-2.141	-0.620	-3.390	H
HETATM	19	C	0	-5.056	-0.848	-1.664	C
HETATM	20	H	0	-5.258	-1.514	0.366	H
HETATM	21	H	0	-4.589	-0.306	-3.714	H
HETATM	22	H	0	-6.139	-0.723	-1.784	H
HETATM	23	O	0	-2.637	-2.008	0.849	O
HETATM	24	C	0	-3.262	-2.047	2.151	C
HETATM	25	H	0	-2.387	-2.282	2.779	H
HETATM	26	C	0	3.583	0.832	0.379	C
HETATM	27	C	0	4.399	0.855	-0.768	C
HETATM	28	C	0	3.914	0.035	1.492	C
HETATM	29	C	0	5.509	0.009	-0.812	C
HETATM	30	C	0	5.030	-0.805	1.400	C
HETATM	31	C	0	5.828	-0.843	0.251	C
HETATM	32	H	0	6.134	0.003	-1.715	H
HETATM	33	H	0	5.291	-1.434	2.261	H
HETATM	34	C	0	-1.069	2.320	0.071	C
HETATM	35	C	0	-1.646	2.243	-1.209	C
HETATM	36	C	0	-1.866	2.446	1.225	C
HETATM	37	C	0	-3.041	2.276	-1.313	C
HETATM	38	C	0	-3.255	2.462	1.074	C
HETATM	39	C	0	-3.860	2.371	-0.185	C
HETATM	40	H	0	-3.499	2.217	-2.309	H
HETATM	41	H	0	-3.884	2.537	1.972	H
HETATM	42	C	0	4.112	1.775	-1.915	C
HETATM	43	H	0	4.451	1.335	-2.867	H
HETATM	44	H	0	3.034	1.979	-2.024	H
HETATM	45	H	0	4.638	2.743	-1.798	H
HETATM	46	C	0	3.128	0.109	2.765	C
HETATM	47	H	0	3.054	1.147	3.142	H
HETATM	48	H	0	2.086	-0.246	2.653	H
HETATM	49	H	0	3.603	-0.494	3.557	H
HETATM	50	C	0	6.992	-1.782	0.154	C
HETATM	51	H	0	6.722	-2.694	-0.411	H
HETATM	52	H	0	7.846	-1.327	-0.378	H
HETATM	53	H	0	7.342	-2.110	1.147	H
HETATM	54	C	0	-0.786	2.147	-2.433	C
HETATM	55	H	0	-1.395	2.168	-3.351	H
HETATM	56	H	0	-0.062	2.982	-2.493	H
HETATM	57	H	0	-0.173	1.223	-2.455	H
HETATM	58	C	0	-1.257	2.595	2.587	C
HETATM	59	H	0	-1.129	3.662	2.861	H
HETATM	60	H	0	-1.901	2.146	3.361	H
HETATM	61	H	0	-0.274	2.101	2.659	H
HETATM	62	C	0	-5.354	2.385	-0.310	C
HETATM	63	H	0	-5.815	1.566	0.274	H
HETATM	64	H	0	-5.791	3.326	0.073	H
HETATM	65	H	0	-5.679	2.266	-1.358	H
HETATM	66	C	0	-4.255	-3.188	2.275	C
HETATM	67	H	0	-4.504	-3.343	3.339	H
HETATM	68	H	0	-5.204	-3.013	1.740	H
HETATM	69	H	0	-3.820	-4.129	1.897	H
HETATM	70	C	0	-3.776	-0.692	2.594	C
HETATM	71	H	0	-4.720	-0.388	2.108	H
HETATM	72	H	0	-3.958	-0.711	3.683	H
HETATM	73	H	0	-3.014	0.078	2.388	H

```
HETATM   74  H              0         3.251   3.579   0.067                       H
HETATM   75  H              0         1.002   4.253  -0.417                       H
HETATM   76  C              0         0.132  -2.995  -0.431                       C
HETATM   77  H              0        -0.759  -3.239   0.162                       H
HETATM   78  C              0         1.375  -2.676   0.154                       C
HETATM   79  H              0         2.279  -2.897  -0.432                       H
HETATM   80  H              0         1.449  -2.719   1.249                       H
HETATM   81  F              0         0.140  -3.604  -1.630                       F
END
```

at3I.pdb

```
TITLE      at3I.pdb
HETATM    1 Ru              0         0.585  -0.498  -0.348                      Ru
HETATM    2  C              0         1.380   3.574   0.740                       C
HETATM    3  C              0         2.712   2.920   1.098                       C
HETATM    4  H              0         0.922   4.122   1.581                       H
HETATM    5  H              0         2.917   2.921   2.187                       H
HETATM    6  C              0         1.266   1.298   0.253                       C
HETATM    7  N              0         2.537   1.544   0.622                       N
HETATM    8  N              0         0.544   2.428   0.367                       N
HETATM    9 Cl              0        -0.605  -0.491   1.796                      Cl
HETATM   10 Cl              0         1.897  -0.436  -2.371                      Cl
HETATM   11  C              0        -0.914  -1.347  -1.418                       C
HETATM   12  H              0        -0.589  -1.209  -2.465                       H
HETATM   13  C              0        -2.371  -1.176  -1.322                       C
HETATM   14  C              0        -3.231  -1.703  -0.318                       C
HETATM   15  C              0        -2.965  -0.466  -2.386                       C
HETATM   16  C              0        -4.617  -1.507  -0.427                       C
HETATM   17  C              0        -4.333  -0.246  -2.468                       C
HETATM   18  H              0        -2.302  -0.072  -3.168                       H
HETATM   19  C              0        -5.162  -0.777  -1.480                       C
HETATM   20  H              0        -5.286  -1.962   0.307                       H
HETATM   21  H              0        -4.754   0.321  -3.306                       H
HETATM   22  H              0        -6.249  -0.643  -1.534                       H
HETATM   23  O              0        -2.666  -2.445   0.648                       O
HETATM   24  C              0        -3.208  -2.584   1.983                       C
HETATM   25  H              0        -2.285  -2.765   2.561                       H
HETATM   26  C              0         3.616   0.612   0.522                       C
HETATM   27  C              0         4.496   0.698  -0.573                       C
HETATM   28  C              0         3.786  -0.365   1.521                       C
HETATM   29  C              0         5.519  -0.248  -0.679                       C
HETATM   30  C              0         4.820  -1.296   1.371                       C
HETATM   31  C              0         5.685  -1.261   0.272                       C
HETATM   32  H              0         6.193  -0.203  -1.544                       H
HETATM   33  H              0         4.954  -2.066   2.142                       H
HETATM   34  C              0        -0.872   2.528   0.214                       C
HETATM   35  C              0        -1.434   2.524  -1.074                       C
HETATM   36  C              0        -1.680   2.632   1.364                       C
HETATM   37  C              0        -2.827   2.598  -1.194                       C
HETATM   38  C              0        -3.065   2.682   1.198                       C
HETATM   39  C              0        -3.657   2.653  -0.071                       C
HETATM   40  H              0        -3.273   2.590  -2.196                       H
HETATM   41  H              0        -3.703   2.727   2.091                       H
HETATM   42  C              0         4.360   1.776  -1.604                       C
HETATM   43  H              0         4.724   1.429  -2.585                       H
HETATM   44  H              0         3.312   2.090  -1.745                       H
HETATM   45  H              0         4.954   2.673  -1.337                       H
HETATM   46  C              0         2.901  -0.395   2.730                       C
HETATM   47  H              0         2.929   0.565   3.281                       H
HETATM   48  H              0         1.837  -0.570   2.482                       H
```

```
HETATM   49  H             0        3.217  -1.184   3.432                      H
HETATM   50  C             0        6.755  -2.296   0.103                      C
HETATM   51  H             0        6.433  -3.090  -0.598                      H
HETATM   52  H             0        7.683  -1.869  -0.314                      H
HETATM   53  H             0        7.004  -2.793   1.056                      H
HETATM   54  C             0       -0.563   2.443  -2.291                      C
HETATM   55  H             0       -1.141   2.638  -3.210                      H
HETATM   56  H             0        0.268   3.172  -2.254                      H
HETATM   57  H             0       -0.077   1.453  -2.414                      H
HETATM   58  C             0       -1.086   2.691   2.739                      C
HETATM   59  H             0       -0.912   3.736   3.066                      H
HETATM   60  H             0       -1.765   2.235   3.479                      H
HETATM   61  H             0       -0.131   2.144   2.801                      H
HETATM   62  C             0       -5.149   2.669  -0.210                      C
HETATM   63  H             0       -5.599   1.760   0.234                      H
HETATM   64  H             0       -5.605   3.531   0.311                      H
HETATM   65  H             0       -5.464   2.706  -1.266                      H
HETATM   66  C             0       -4.089  -3.815   2.080                      C
HETATM   67  H             0       -4.328  -4.023   3.138                      H
HETATM   68  H             0       -5.046  -3.699   1.541                      H
HETATM   69  H             0       -3.575  -4.702   1.671                      H
HETATM   70  C             0       -3.843  -1.322   2.539                      C
HETATM   71  H             0       -4.891  -1.172   2.226                      H
HETATM   72  H             0       -3.842  -1.385   3.642                      H
HETATM   73  H             0       -3.253  -0.435   2.257                      H
HETATM   74  H             0        3.575   3.390   0.596                      H
HETATM   75  H             0        1.457   4.276  -0.114                      H
HETATM   76  C             0       -0.134  -2.605  -0.887                      C
HETATM   77  H             0       -0.875  -3.216  -0.345                      H
HETATM   78  C             0        1.058  -2.366   0.079                      C
HETATM   79  H             0        1.995  -2.743  -0.365                      H
HETATM   80  H             0        0.868  -2.682   1.117                      H
HETATM   81  F             0        0.318  -3.301  -1.986                      F
END
```

at3e.pdb

```
TITLE       at3e.pdb
HETATM    1 Ru             0        0.552  -0.651  -0.240                     Ru
HETATM    2 Cl             0        1.644  -0.418  -2.413                     Cl
HETATM    3 Cl             0       -0.452  -0.783   1.976                     Cl
HETATM    4  C             0        1.198   1.257   0.191                      C
HETATM    5  N             0        2.471   1.614   0.446                      N
HETATM    6  C             0        2.603   3.038   0.771                      C
HETATM    7  C             0        1.185   3.568   0.573                      C
HETATM    8  N             0        0.421   2.350   0.284                      N
HETATM    9  C             0        3.616   0.757   0.395                      C
HETATM   10  C             0        4.439   0.771  -0.745                      C
HETATM   11  C             0        5.536  -0.098  -0.777                      C
HETATM   12  C             0        5.832  -0.952   0.290                      C
HETATM   13  C             0        5.019  -0.908   1.428                      C
HETATM   14  C             0        3.914  -0.054   1.508                      C
HETATM   15  C             0        4.187   1.704  -1.889                      C
HETATM   16  C             0        6.985  -1.906   0.207                      C
HETATM   17  C             0        3.077   0.007   2.748                      C
HETATM   18  C             0       -1.008   2.372   0.219                      C
HETATM   19  C             0       -1.752   2.420   1.415                      C
HETATM   20  C             0       -3.145   2.474   1.323                      C
HETATM   21  C             0       -3.802   2.515   0.088                      C
HETATM   22  C             0       -3.033   2.501  -1.078                      C
HETATM   23  C             0       -1.637   2.422  -1.039                      C
```

```
HETATM   24  C              0      -1.089   2.441   2.758                       C
HETATM   25  C              0      -5.298   2.563   0.026                       C
HETATM   26  C              0      -0.832   2.415  -2.303                       C
HETATM   27  H              0       3.342   3.512   0.101                       H
HETATM   28  H              0       2.974   3.159   1.807                       H
HETATM   29  H              0       1.099   4.279  -0.270                       H
HETATM   30  H              0       0.778   4.069   1.469                       H
HETATM   31  H              0       6.173  -0.110  -1.671                       H
HETATM   32  H              0       5.255  -1.550   2.287                       H
HETATM   33  H              0       3.118   1.953  -1.997                       H
HETATM   34  H              0       4.506   1.256  -2.843                       H
HETATM   35  H              0       4.756   2.648  -1.769                       H
HETATM   36  H              0       7.838  -1.477  -0.347                       H
HETATM   37  H              0       7.342  -2.210   1.205                       H
HETATM   38  H              0       6.700  -2.833  -0.327                       H
HETATM   39  H              0       2.022  -0.272   2.565                       H
HETATM   40  H              0       3.054   1.027   3.177                       H
HETATM   41  H              0       3.471  -0.667   3.527                       H
HETATM   42  H              0      -3.735   2.485   2.250                       H
HETATM   43  H              0      -3.532   2.548  -2.053                       H
HETATM   44  H              0      -1.681   1.876   3.498                       H
HETATM   45  H              0      -0.086   1.984   2.740                       H
HETATM   46  H              0      -0.993   3.475   3.146                       H
HETATM   47  H              0      -5.747   1.646   0.452                       H
HETATM   48  H              0      -5.709   3.409   0.609                       H
HETATM   49  H              0      -5.664   2.658  -1.010                       H
HETATM   50  H              0      -1.487   2.459  -3.189                       H
HETATM   51  H              0      -0.194   1.514  -2.401                       H
HETATM   52  H              0      -0.140   3.278  -2.356                       H
HETATM   53  C              0      -0.788  -1.912  -1.384                       C
HETATM   54  C              0      -2.151  -1.367  -1.415                       C
HETATM   55  C              0      -3.169  -1.608  -0.451                       C
HETATM   56  C              0      -4.468  -1.136  -0.700                       C
HETATM   57  C              0      -4.763  -0.426  -1.862                       C
HETATM   58  C              0      -3.770  -0.170  -2.807                       C
HETATM   59  C              0      -2.483  -0.638  -2.575                       C
HETATM   60  O              0      -2.825  -2.369   0.597                       O
HETATM   61  C              0      -3.516  -2.377   1.865                       C
HETATM   62  C              0      -4.637  -3.399   1.868                       C
HETATM   63  C              0      -3.920  -1.001   2.362                       C
HETATM   64  H              0      -0.345  -1.970  -2.389                       H
HETATM   65  H              0      -5.272  -1.360   0.004                       H
HETATM   66  H              0      -5.791  -0.081  -2.029                       H
HETATM   67  H              0      -3.999   0.387  -3.723                       H
HETATM   68  H              0      -1.688  -0.465  -3.311                       H
HETATM   69  H              0      -5.023  -3.529   2.895                       H
HETATM   70  H              0      -4.278  -4.381   1.518                       H
HETATM   71  H              0      -5.488  -3.102   1.231                       H
HETATM   72  H              0      -2.712  -2.738   2.531                       H
HETATM   73  H              0      -4.870  -0.632   1.937                       H
HETATM   74  H              0      -3.123  -0.270   2.149                       H
HETATM   75  H              0      -4.052  -1.044   3.458                       H
HETATM   76  C              0      -0.158  -2.850  -0.507                       C
HETATM   77  C              0       1.644  -2.069   0.219                       C
HETATM   78  F              0       0.517  -3.846  -1.144                       F
HETATM   79  H              0      -0.616  -3.195   0.427                       H
HETATM   80  H              0       2.383  -2.490  -0.490                       H
HETATM   81  H              0       1.651  -2.494   1.244                       H
END
```

at3f.pdb

```
TITLE      at3f.pdb
HETATM    1 Ru           0      -0.495  -0.716   0.249              Ru
HETATM    2  C           0      -1.180   3.478  -0.398               C
HETATM    3  C           0      -2.628   3.017  -0.292               C
HETATM    4  H           0      -0.929   3.887  -1.396               H
HETATM    5  H           0      -3.243   3.300  -1.165               H
HETATM    6  C           0      -1.217   1.145  -0.101               C
HETATM    7  N           0      -2.504   1.560  -0.209               N
HETATM    8  N           0      -0.428   2.243  -0.167               N
HETATM    9 Cl           0       0.289  -0.960  -2.052              Cl
HETATM   10 Cl           0      -1.223  -0.378   2.575              Cl
HETATM   11  C           0       0.910  -2.339   1.352               C
HETATM   12  C           0       2.123  -1.522   1.466               C
HETATM   13  C           0       3.187  -1.535   0.518               C
HETATM   14  C           0       2.292  -0.793   2.660               C
HETATM   15  C           0       4.374  -0.847   0.816               C
HETATM   16  C           0       3.469  -0.109   2.939               C
HETATM   17  H           0       1.458  -0.781   3.372               H
HETATM   18  C           0       4.511  -0.146   2.013               C
HETATM   19  H           0       5.213  -0.878   0.119               H
HETATM   20  H           0       3.574   0.448   3.877               H
HETATM   21  H           0       5.451   0.381   2.216               H
HETATM   22  O           0       2.985  -2.293  -0.570               O
HETATM   23  C           0       3.782  -2.257  -1.769               C
HETATM   24  H           0       3.133  -2.827  -2.458               H
HETATM   25  C           0      -3.669   0.745  -0.270               C
HETATM   26  C           0      -4.517   0.637   0.847               C
HETATM   27  C           0      -3.984   0.110  -1.491               C
HETATM   28  C           0      -5.654  -0.172   0.740               C
HETATM   29  C           0      -5.131  -0.689  -1.552               C
HETATM   30  C           0      -5.968  -0.852  -0.442               C
HETATM   31  H           0      -6.311  -0.275   1.614               H
HETATM   32  H           0      -5.378  -1.190  -2.496               H
HETATM   33  C           0       0.998   2.266  -0.245               C
HETATM   34  C           0       1.746   2.421   0.941               C
HETATM   35  C           0       1.620   2.283  -1.507               C
HETATM   36  C           0       3.133   2.552   0.839               C
HETATM   37  C           0       3.015   2.396  -1.557               C
HETATM   38  C           0       3.785   2.522  -0.399               C
HETATM   39  H           0       3.720   2.676   1.758               H
HETATM   40  H           0       3.510   2.385  -2.538               H
HETATM   41  C           0      -4.232   1.375   2.118               C
HETATM   42  H           0      -4.667   0.852   2.986               H
HETATM   43  H           0      -3.149   1.470   2.303               H
HETATM   44  H           0      -4.675   2.391   2.104               H
HETATM   45  C           0      -3.114   0.304  -2.693               C
HETATM   46  H           0      -2.948   1.378  -2.908               H
HETATM   47  H           0      -2.107  -0.141  -2.562               H
HETATM   48  H           0      -3.566  -0.150  -3.590               H
HETATM   49  C           0      -7.169  -1.746  -0.516               C
HETATM   50  H           0      -6.902  -2.797  -0.296               H
HETATM   51  H           0      -7.946  -1.457   0.212               H
HETATM   52  H           0      -7.624  -1.742  -1.522               H
HETATM   53  C           0       1.066   2.486   2.273               C
HETATM   54  H           0       1.803   2.562   3.090               H
HETATM   55  H           0       0.398   3.366   2.349               H
HETATM   56  H           0       0.430   1.602   2.468               H
HETATM   57  C           0       0.836   2.244  -2.784               C
HETATM   58  H           0       0.730   3.259  -3.217               H
HETATM   59  H           0       1.345   1.622  -3.539               H
HETATM   60  H           0      -0.171   1.817  -2.653               H
HETATM   61  C           0       5.280   2.593  -0.471               C
```

```
HETATM   62  H             0       5.734   1.606  -0.252                  H
HETATM   63  H             0       5.635   2.895  -1.471                  H
HETATM   64  H             0       5.699   3.298   0.269                  H
HETATM   65  C             0       5.081  -3.027  -1.613                  C
HETATM   66  H             0       5.544  -3.173  -2.605                  H
HETATM   67  H             0       5.821  -2.511  -0.979                  H
HETATM   68  H             0       4.898  -4.025  -1.180                  H
HETATM   69  C             0       3.926  -0.864  -2.347                  C
HETATM   70  H             0       4.648  -0.229  -1.806                  H
HETATM   71  H             0       4.275  -0.940  -3.393                  H
HETATM   72  H             0       2.943  -0.363  -2.347                  H
HETATM   73  H             0      -3.136   3.406   0.611                  H
HETATM   74  H             0      -0.907   4.242   0.351                  H
HETATM   75  C             0       0.459  -3.089   0.301                  C
HETATM   76  C             0      -2.051  -1.599  -0.052                  C
HETATM   77  H             0      -2.196  -2.201  -0.976                  H
HETATM   78  H             0      -2.869  -1.656   0.695                  H
HETATM   79  H             0       0.373  -2.497   2.296                  H
HETATM   80  F             0      -0.517  -3.980   0.527                  F
HETATM   81  H             0       0.875  -3.160  -0.705                  H
END
```

s2g.pdb

```
TITLE       s2g.pdb
HETATM    1 Ru             0      -0.299  -0.773  -0.087                 Ru
HETATM    2  C             0       0.035   3.385   0.099                  C
HETATM    3  C             0       1.536   3.101   0.176                  C
HETATM    4  H             0      -0.344   3.942   0.976                  H
HETATM    5  H             0       1.984   3.415   1.138                  H
HETATM    6  C             0       0.399   1.061   0.011                  C
HETATM    7  N             0       1.618   1.642   0.042                  N
HETATM    8  N             0      -0.538   2.046   0.054                  N
HETATM    9 Cl             0      -0.698  -1.100   2.268                 Cl
HETATM   10 Cl             0      -0.388  -0.744  -2.496                 Cl
HETATM   11  C             0      -2.683  -2.553  -0.271                  C
HETATM   12  C             0       2.878   0.979   0.077                  C
HETATM   13  C             0       3.616   0.854  -1.116                  C
HETATM   14  C             0       3.376   0.497   1.300                  C
HETATM   15  C             0       4.871   0.243  -1.062                  C
HETATM   16  C             0       4.630  -0.128   1.306                  C
HETATM   17  C             0       5.389  -0.259   0.139                  C
HETATM   18  H             0       5.450   0.137  -1.988                  H
HETATM   19  H             0       5.025  -0.513   2.256                  H
HETATM   20  C             0      -1.931   1.735   0.087                  C
HETATM   21  C             0      -2.654   1.614  -1.121                  C
HETATM   22  C             0      -2.566   1.545   1.335                  C
HETATM   23  C             0      -3.975   1.156  -1.059                  C
HETATM   24  C             0      -3.888   1.093   1.346                  C
HETATM   25  C             0      -4.593   0.859   0.159                  C
HETATM   26  H             0      -4.531   1.023  -1.996                  H
HETATM   27  H             0      -4.374   0.907   2.312                  H
HETATM   28  C             0       3.019   1.285  -2.419                  C
HETATM   29  H             0       3.740   1.181  -3.246                  H
HETATM   30  H             0       2.131   0.669  -2.663                  H
HETATM   31  H             0       2.674   2.335  -2.406                  H
HETATM   32  C             0       2.577   0.626   2.560                  C
HETATM   33  H             0       2.179   1.648   2.698                  H
HETATM   34  H             0       1.697  -0.047   2.566                  H
HETATM   35  H             0       3.186   0.381   3.445                  H
HETATM   36  C             0       6.720  -0.949   0.161                  C
```

```
HETATM   37  H             0        6.647  -1.975  -0.247                         H
HETATM   38  H             0        7.467  -0.421  -0.457                         H
HETATM   39  H             0        7.123  -1.037   1.183                         H
HETATM   40  C             0       -2.075   2.046  -2.433                         C
HETATM   41  H             0       -2.405   1.392  -3.255                         H
HETATM   42  H             0       -2.413   3.075  -2.672                         H
HETATM   43  H             0       -0.975   2.040  -2.439                         H
HETATM   44  C             0       -1.877   1.885   2.620                         C
HETATM   45  H             0       -2.082   2.939   2.897                         H
HETATM   46  H             0       -2.232   1.250   3.446                         H
HETATM   47  H             0       -0.784   1.757   2.563                         H
HETATM   48  C             0       -5.970   0.272   0.193                         C
HETATM   49  H             0       -5.923  -0.834   0.184                         H
HETATM   50  H             0       -6.521   0.556   1.106                         H
HETATM   51  H             0       -6.572   0.572  -0.681                         H
HETATM   52  H             0        2.112   3.594  -0.627                         H
HETATM   53  H             0       -0.249   3.959  -0.805                         H
HETATM   54  C             0       -1.462  -3.106  -0.331                         C
HETATM   55  H             0       -1.027  -3.521   0.581                         H
HETATM   56  C             0        1.281  -1.675  -0.067                         C
HETATM   57  H             0       -1.021  -3.295  -1.312                         H
HETATM   58  F             0       -3.380  -2.390   0.820                         F
HETATM   59  F             0       -3.357  -2.122  -1.304                         F
HETATM   60  F             0        2.227  -1.690  -0.980                         F
HETATM   61  F             0        1.641  -2.574   0.833                         F
END
```

s2h.pdb

```
TITLE       s2h.pdb
HETATM    1 Ru             0        0.202  -0.802   0.007                        Ru
HETATM    2 Cl             0        1.033  -0.673  -2.277                        Cl
HETATM    3 Cl             0       -0.621  -0.865   2.288                        Cl
HETATM    4  C             0       -0.145   1.220   0.009                         C
HETATM    5  N             0        0.880   2.086   0.078                         N
HETATM    6  C             0        0.433   3.473   0.215                         C
HETATM    7  C             0       -1.089   3.348   0.062                         C
HETATM    8  N             0       -1.302   1.896   0.020                         N
HETATM    9  C             0        2.225   1.605   0.121                         C
HETATM   10  C             0        3.005   1.652  -1.055                         C
HETATM   11  C             0        4.245   1.018  -1.054                         C
HETATM   12  C             0        4.723   0.336   0.075                         C
HETATM   13  C             0        3.963   0.365   1.244                         C
HETATM   14  C             0        2.719   1.011   1.301                         C
HETATM   15  C             0       -2.587   1.277  -0.047                         C
HETATM   16  C             0       -3.361   1.157   1.120                         C
HETATM   17  C             0       -4.587   0.488   1.036                         C
HETATM   18  C             0       -5.051  -0.049  -0.170                         C
HETATM   19  C             0       -4.275   0.121  -1.323                         C
HETATM   20  C             0       -3.046   0.787  -1.286                         C
HETATM   21  C             0        2.501   2.353  -2.278                         C
HETATM   22  C             0        6.001  -0.441   0.010                         C
HETATM   23  C             0        2.023   1.125   2.624                         C
HETATM   24  C             0       -2.910   1.739   2.425                         C
HETATM   25  C             0       -6.339  -0.813  -0.222                         C
HETATM   26  C             0       -2.240   0.975  -2.535                         C
HETATM   27  H             0        0.894   4.111  -0.560                         H
HETATM   28  H             0        0.736   3.872   1.202                         H
HETATM   29  H             0       -1.468   3.812  -0.868                         H
HETATM   30  H             0       -1.647   3.792   0.906                         H
HETATM   31  H             0        4.840   1.018  -1.976                         H
```

```
HETATM   32  H              0       4.338  -0.130   2.150                      H
HETATM   33  H              0      -5.189   0.372   1.946                      H
HETATM   34  H              0      -4.637  -0.270  -2.283                      H
HETATM   35  H              0       1.430   2.147  -2.447                      H
HETATM   36  H              0       3.043   2.021  -3.178                      H
HETATM   37  H              0       2.631   3.451  -2.203                      H
HETATM   38  H              0       5.816  -1.458  -0.387                      H
HETATM   39  H              0       6.464  -0.565   1.003                      H
HETATM   40  H              0       6.740   0.029  -0.661                      H
HETATM   41  H              0       2.641   1.725   3.319                      H
HETATM   42  H              0       1.869   0.138   3.093                      H
HETATM   43  H              0       1.030   1.595   2.560                      H
HETATM   44  H              0      -1.812   1.763   2.510                      H
HETATM   45  H              0      -3.285   1.145   3.274                      H
HETATM   46  H              0      -3.291   2.772   2.559                      H
HETATM   47  H              0      -6.169  -1.893  -0.048                      H
HETATM   48  H              0      -6.832  -0.726  -1.205                      H
HETATM   49  H              0      -7.051  -0.478   0.550                      H
HETATM   50  H              0      -2.823   0.697  -3.428                      H
HETATM   51  H              0      -1.313   0.369  -2.542                      H
HETATM   52  H              0      -1.915   2.025  -2.661                      H
HETATM   53  C              0      -1.217  -1.903  -0.524                      C
HETATM   54  F              0      -1.511  -2.248  -1.758                      F
HETATM   55  F              0      -2.190  -2.363   0.225                      F
HETATM   56  C              0       1.620  -2.325   0.385                      C
HETATM   57  C              0       0.450  -3.051   0.009                      C
HETATM   58  F              0       2.693  -2.324  -0.399                      F
HETATM   59  F              0       2.035  -2.336   1.653                      F
HETATM   60  H              0       0.499  -3.540  -0.972                      H
HETATM   61  H              0      -0.070  -3.572   0.823                      H
END
```

s2NA.pdb

```
TITLE      s2NA.pdb
HETATM    1 Ru              0      -0.002  -0.799  -0.005                     Ru
HETATM    2  C              0      -0.761   3.425  -0.033                      C
HETATM    3  C              0       0.768   3.419   0.089                      C
HETATM    4  H              0      -1.262   3.915   0.821                      H
HETATM    5  H              0       1.131   3.884   1.025                      H
HETATM    6  C              0      -0.002   1.229   0.004                      C
HETATM    7  N              0       1.097   1.989   0.078                      N
HETATM    8  N              0      -1.097   1.995  -0.057                      N
HETATM    9 Cl              0      -0.815  -0.764   2.278                     Cl
HETATM   10 Cl              0       0.814  -0.755  -2.287                     Cl
HETATM   11  C              0      -1.281  -2.221  -0.428                      C
HETATM   12  C              0       2.422   1.447   0.101                      C
HETATM   13  C              0       3.174   1.410  -1.090                      C
HETATM   14  C              0       2.938   0.940   1.309                      C
HETATM   15  C              0       4.422   0.783  -1.066                      C
HETATM   16  C              0       4.195   0.323   1.286                      C
HETATM   17  C              0       4.937   0.216   0.106                      C
HETATM   18  H              0       5.002   0.726  -1.996                      H
HETATM   19  H              0       4.600  -0.083   2.221                      H
HETATM   20  C              0      -2.423   1.456  -0.086                      C
HETATM   21  C              0      -2.940   0.974  -1.306                      C
HETATM   22  C              0      -3.173   1.394   1.104                      C
HETATM   23  C              0      -4.191   0.347  -1.293                      C
HETATM   24  C              0      -4.418   0.761   1.068                      C
HETATM   25  C              0      -4.930   0.212  -0.113                      C
HETATM   26  H              0      -4.596  -0.041  -2.236                      H
```

```
HETATM   27  H              0       -4.997   0.682   1.997                       H
HETATM   28  C              0        2.675   2.046  -2.351                       C
HETATM   29  H              0        3.089   1.542  -3.239                       H
HETATM   30  H              0        1.578   1.999  -2.439                       H
HETATM   31  H              0        2.980   3.111  -2.412                       H
HETATM   32  C              0        2.185   1.052   2.598                       C
HETATM   33  H              0        1.577   1.972   2.654                       H
HETATM   34  H              0        1.473   0.217   2.742                       H
HETATM   35  H              0        2.876   1.047   3.457                       H
HETATM   36  C              0        6.247  -0.511   0.090                       C
HETATM   37  H              0        6.101  -1.580  -0.155                       H
HETATM   38  H              0        6.938  -0.105  -0.669                       H
HETATM   39  H              0        6.752  -0.476   1.069                       H
HETATM   40  C              0       -2.189   1.140  -2.591                       C
HETATM   41  H              0       -2.845   0.948  -3.455                       H
HETATM   42  H              0       -1.779   2.161  -2.701                       H
HETATM   43  H              0       -1.325   0.452  -2.673                       H
HETATM   44  C              0       -2.679   2.011   2.376                       C
HETATM   45  H              0       -3.001   3.069   2.457                       H
HETATM   46  H              0       -3.082   1.483   3.255                       H
HETATM   47  H              0       -1.581   1.980   2.459                       H
HETATM   48  C              0       -6.233  -0.527  -0.110                       C
HETATM   49  H              0       -6.075  -1.603   0.096                       H
HETATM   50  H              0       -6.920  -0.154   0.669                       H
HETATM   51  H              0       -6.748  -0.463  -1.083                       H
HETATM   52  H              0        1.271   3.929  -0.752                       H
HETATM   53  H              0       -1.121   3.915  -0.956                       H
HETATM   54  C              0       -0.002  -3.040  -0.008                       C
HETATM   55  H              0       -0.292  -3.639   0.870                       H
HETATM   56  H              0        0.286  -3.638  -0.888                       H
HETATM   57  C              0        1.277  -2.223   0.412                       C
HETATM   58  F              0        2.341  -2.465  -0.355                       F
HETATM   59  F              0        1.665  -2.457   1.670                       F
HETATM   60  F              0       -1.666  -2.450  -1.687                       F
HETATM   61  F              0       -2.346  -2.464   0.336                       F
END
```

s2i.pdb

```
TITLE      s2i.pdb
HETATM    1 Ru              0        0.202  -0.802   0.007                      Ru
HETATM    2 Cl              0        1.033  -0.673  -2.277                      Cl
HETATM    3 Cl              0       -0.621  -0.865   2.288                      Cl
HETATM    4  C              0       -0.145   1.220   0.009                       C
HETATM    5  N              0        0.880   2.086   0.078                       N
HETATM    6  C              0        0.433   3.473   0.215                       C
HETATM    7  C              0       -1.089   3.348   0.062                       C
HETATM    8  N              0       -1.302   1.896   0.020                       N
HETATM    9  C              0        2.225   1.605   0.121                       C
HETATM   10  C              0        3.005   1.652  -1.055                       C
HETATM   11  C              0        4.245   1.018  -1.054                       C
HETATM   12  C              0        4.723   0.336   0.075                       C
HETATM   13  C              0        3.963   0.365   1.244                       C
HETATM   14  C              0        2.719   1.011   1.301                       C
HETATM   15  C              0       -2.587   1.277  -0.047                       C
HETATM   16  C              0       -3.361   1.157   1.120                       C
HETATM   17  C              0       -4.587   0.488   1.036                       C
HETATM   18  C              0       -5.051  -0.049  -0.170                       C
HETATM   19  C              0       -4.275   0.121  -1.323                       C
HETATM   20  C              0       -3.046   0.787  -1.286                       C
HETATM   21  C              0        2.501   2.353  -2.278                       C
HETATM   22  C              0        6.001  -0.441   0.010                       C
```

```
HETATM   23  C              0       2.023   1.125   2.624                        C
HETATM   24  C              0      -2.910   1.739   2.425                        C
HETATM   25  C              0      -6.339  -0.813  -0.222                        C
HETATM   26  C              0      -2.240   0.975  -2.535                        C
HETATM   27  H              0       0.894   4.111  -0.560                        H
HETATM   28  H              0       0.736   3.872   1.202                        H
HETATM   29  H              0      -1.468   3.812  -0.868                        H
HETATM   30  H              0      -1.647   3.792   0.906                        H
HETATM   31  H              0       4.840   1.018  -1.976                        H
HETATM   32  H              0       4.338  -0.130   2.150                        H
HETATM   33  H              0      -5.189   0.372   1.946                        H
HETATM   34  H              0      -4.637  -0.270  -2.283                        H
HETATM   35  H              0       1.430   2.147  -2.447                        H
HETATM   36  H              0       3.043   2.021  -3.178                        H
HETATM   37  H              0       2.631   3.451  -2.203                        H
HETATM   38  H              0       5.816  -1.458  -0.387                        H
HETATM   39  H              0       6.464  -0.565   1.003                        H
HETATM   40  H              0       6.740   0.029  -0.661                        H
HETATM   41  H              0       2.641   1.725   3.319                        H
HETATM   42  H              0       1.869   0.138   3.093                        H
HETATM   43  H              0       1.030   1.595   2.560                        H
HETATM   44  H              0      -1.812   1.763   2.510                        H
HETATM   45  H              0      -3.285   1.145   3.274                        H
HETATM   46  H              0      -3.291   2.772   2.559                        H
HETATM   47  H              0      -6.169  -1.893  -0.048                        H
HETATM   48  H              0      -6.832  -0.726  -1.205                        H
HETATM   49  H              0      -7.051  -0.478   0.550                        H
HETATM   50  H              0      -2.823   0.697  -3.428                        H
HETATM   51  H              0      -1.313   0.369  -2.542                        H
HETATM   52  H              0      -1.915   2.025  -2.661                        H
HETATM   53  C              0      -1.217  -1.903  -0.524                        C
HETATM   54  F              0      -1.511  -2.248  -1.758                        F
HETATM   55  F              0      -2.190  -2.363   0.225                        F
HETATM   56  C              0       1.620  -2.325   0.385                        C
HETATM   57  C              0       0.450  -3.051   0.009                        C
HETATM   58  F              0       2.693  -2.324  -0.399                        F
HETATM   59  F              0       2.035  -2.336   1.653                        F
HETATM   60  H              0       0.499  -3.540  -0.972                        H
HETATM   61  H              0      -0.070  -3.572   0.823                        H
END
```

s2j.pdb

```
TITLE       s2j.pdb
HETATM    1 Ru              0      -0.299  -0.773  -0.087                       Ru
HETATM    2  C              0       0.035   3.385   0.099                        C
HETATM    3  C              0       1.536   3.101   0.176                        C
HETATM    4  H              0      -0.344   3.942   0.976                        H
HETATM    5  H              0       1.984   3.415   1.138                        H
HETATM    6  C              0       0.399   1.061   0.011                        C
HETATM    7  N              0       1.618   1.642   0.042                        N
HETATM    8  N              0      -0.538   2.046   0.054                        N
HETATM    9 Cl              0      -0.698  -1.100   2.268                       Cl
HETATM   10 Cl              0      -0.388  -0.744  -2.496                       Cl
HETATM   11  C              0      -2.683  -2.553  -0.271                        C
HETATM   12  C              0       2.878   0.979   0.077                        C
HETATM   13  C              0       3.616   0.854  -1.116                        C
HETATM   14  C              0       3.376   0.497   1.300                        C
HETATM   15  C              0       4.871   0.243  -1.062                        C
HETATM   16  C              0       4.630  -0.128   1.306                        C
HETATM   17  C              0       5.389  -0.259   0.139                        C
HETATM   18  H              0       5.450   0.137  -1.988                        H
```

```
HETATM   19  H            0       5.025  -0.513   2.256                          H
HETATM   20  C            0      -1.931   1.735   0.087                          C
HETATM   21  C            0      -2.654   1.614  -1.121                          C
HETATM   22  C            0      -2.566   1.545   1.335                          C
HETATM   23  C            0      -3.975   1.156  -1.059                          C
HETATM   24  C            0      -3.888   1.093   1.346                          C
HETATM   25  C            0      -4.593   0.859   0.159                          C
HETATM   26  H            0      -4.531   1.023  -1.996                          H
HETATM   27  H            0      -4.374   0.907   2.312                          H
HETATM   28  C            0       3.019   1.285  -2.419                          C
HETATM   29  H            0       3.740   1.181  -3.246                          H
HETATM   30  H            0       2.131   0.669  -2.663                          H
HETATM   31  H            0       2.674   2.335  -2.406                          H
HETATM   32  C            0       2.577   0.626   2.560                          C
HETATM   33  H            0       2.179   1.648   2.698                          H
HETATM   34  H            0       1.697  -0.047   2.566                          H
HETATM   35  H            0       3.186   0.381   3.445                          H
HETATM   36  C            0       6.720  -0.949   0.161                          C
HETATM   37  H            0       6.647  -1.975  -0.247                          H
HETATM   38  H            0       7.467  -0.421  -0.457                          H
HETATM   39  H            0       7.123  -1.037   1.183                          H
HETATM   40  C            0      -2.075   2.046  -2.433                          C
HETATM   41  H            0      -2.405   1.392  -3.255                          H
HETATM   42  H            0      -2.413   3.075  -2.672                          H
HETATM   43  H            0      -0.975   2.040  -2.439                          H
HETATM   44  C            0      -1.877   1.885   2.620                          C
HETATM   45  H            0      -2.082   2.939   2.897                          H
HETATM   46  H            0      -2.232   1.250   3.446                          H
HETATM   47  H            0      -0.784   1.757   2.563                          H
HETATM   48  C            0      -5.970   0.272   0.193                          C
HETATM   49  H            0      -5.923  -0.834   0.184                          H
HETATM   50  H            0      -6.521   0.556   1.106                          H
HETATM   51  H            0      -6.572   0.572  -0.681                          H
HETATM   52  H            0       2.112   3.594  -0.627                          H
HETATM   53  H            0      -0.249   3.959  -0.805                          H
HETATM   54  C            0      -1.462  -3.106  -0.331                          C
HETATM   55  H            0      -1.027  -3.521   0.581                          H
HETATM   56  C            0       1.281  -1.675  -0.067                          C
HETATM   57  H            0      -1.021  -3.295  -1.312                          H
HETATM   58  F            0      -3.380  -2.390   0.820                          F
HETATM   59  F            0      -3.357  -2.122  -1.304                          F
HETATM   60  F            0       2.227  -1.690  -0.980                          F
HETATM   61  F            0       1.641  -2.574   0.833                          F
END
```

a2g.pdb

```
TITLE       a2g.pdb
HETATM    1 Ru            0      -0.383   0.750   0.013                         Ru
HETATM    2  C            0      -0.545  -3.446   0.043                          C
HETATM    3  C            0       0.980  -3.330   0.112                          C
HETATM    4  H            0      -0.897  -3.944  -0.881                          H
HETATM    5  H            0       1.493  -3.844  -0.721                          H
HETATM    6  C            0       0.068  -1.176   0.028                          C
HETATM    7  N            0       1.215  -1.885   0.038                          N
HETATM    8  N            0      -0.970  -2.050   0.062                          N
HETATM    9 Cl            0      -0.573   0.926  -2.399                         Cl
HETATM   10 Cl            0      -0.615   0.840   2.424                         Cl
HETATM   11  C            0      -1.899   2.641   0.024                          C
HETATM   12  H            0      -2.394   2.428   0.976                          H
HETATM   13  C            0       2.535  -1.348   0.003                          C
HETATM   14  C            0       3.209  -1.086   1.212                          C
```

```
HETATM   15  C              0        3.148  -1.123  -1.243                         C
HETATM   16  C              0        4.511  -0.585   1.150                         C
HETATM   17  C              0        4.454  -0.622  -1.260                         C
HETATM   18  C              0        5.147  -0.346  -0.076                         C
HETATM   19  H              0        5.040  -0.364   2.086                         H
HETATM   20  H              0        4.937  -0.433  -2.227                         H
HETATM   21  C              0       -2.322  -1.593   0.008                         C
HETATM   22  C              0       -3.033  -1.378   1.209                         C
HETATM   23  C              0       -2.931  -1.368  -1.247                         C
HETATM   24  C              0       -4.307  -0.808   1.132                         C
HETATM   25  C              0       -4.206  -0.795  -1.273                         C
HETATM   26  C              0       -4.895  -0.483  -0.096                         C
HETATM   27  H              0       -4.857  -0.616   2.063                         H
HETATM   28  H              0       -4.675  -0.595  -2.246                         H
HETATM   29  C              0        2.516  -1.283   2.524                         C
HETATM   30  H              0        3.190  -1.069   3.369                         H
HETATM   31  H              0        1.635  -0.619   2.619                         H
HETATM   32  H              0        2.141  -2.316   2.653                         H
HETATM   33  C              0        2.391  -1.353  -2.512                         C
HETATM   34  H              0        1.573  -0.613  -2.627                         H
HETATM   35  H              0        3.047  -1.268  -3.394                         H
HETATM   36  H              0        1.911  -2.349  -2.544                         H
HETATM   37  C              0        6.539   0.209  -0.108                         C
HETATM   38  H              0        6.576   1.230   0.315                         H
HETATM   39  H              0        7.236  -0.401   0.494                         H
HETATM   40  H              0        6.939   0.263  -1.134                         H
HETATM   41  C              0       -2.495  -1.828   2.532                         C
HETATM   42  H              0       -2.780  -1.134   3.339                         H
HETATM   43  H              0       -2.907  -2.824   2.790                         H
HETATM   44  H              0       -1.397  -1.898   2.544                         H
HETATM   45  C              0       -2.285  -1.821  -2.520                         C
HETATM   46  H              0       -2.622  -2.847  -2.771                         H
HETATM   47  H              0       -2.558  -1.168  -3.364                         H
HETATM   48  H              0       -1.185  -1.828  -2.464                         H
HETATM   49  C              0       -6.232   0.192  -0.148                         C
HETATM   50  H              0       -6.120   1.294  -0.157                         H
HETATM   51  H              0       -6.795  -0.074  -1.059                         H
HETATM   52  H              0       -6.856  -0.055   0.727                         H
HETATM   53  H              0        1.398  -3.736   1.053                         H
HETATM   54  H              0       -0.977  -3.994   0.901                         H
HETATM   55  C              0       -0.813   3.446   0.034                         C
HETATM   56  C              0        1.326   1.403   0.035                         C
HETATM   57  H              0       -2.385   2.439  -0.935                         H
HETATM   58  F              0        2.026   1.753   1.095                         F
HETATM   59  F              0        2.058   1.745  -1.004                         F
HETATM   60  F              0       -0.247   3.918   1.111                         F
HETATM   61  F              0       -0.233   3.930  -1.030                         F
END
```

a2i.pdb

```
TITLE      a2i.pdb
HETATM    1 Ru              0        0.057   0.902   0.236                        Ru
HETATM    2 Cl              0       -0.844   0.611   2.429                        Cl
HETATM    3 Cl              0        1.031   0.978  -2.027                        Cl
HETATM    4  C              0        0.227  -1.184  -0.024                         C
HETATM    5  N              0        1.376  -1.880  -0.050                         N
HETATM    6  C              0        1.152  -3.325  -0.185                         C
HETATM    7  C              0       -0.369  -3.428  -0.305                         C
HETATM    8  N              0       -0.803  -2.033  -0.157                         N
HETATM    9  C              0        2.685  -1.314   0.028                         C
HETATM   10  C              0        3.186  -0.905   1.279                         C
```

```
HETATM   11  C              0       4.447  -0.301   1.329                       C
HETATM   12  C              0       5.216  -0.117   0.174                       C
HETATM   13  C              0       4.714  -0.584  -1.046                       C
HETATM   14  C              0       3.456  -1.189  -1.142                       C
HETATM   15  C              0      -2.180  -1.649  -0.174                       C
HETATM   16  C              0      -2.738  -1.141  -1.364                       C
HETATM   17  C              0      -4.083  -0.756  -1.354                       C
HETATM   18  C              0      -4.870  -0.872  -0.204                       C
HETATM   19  C              0      -4.297  -1.418   0.950                       C
HETATM   20  C              0      -2.960  -1.821   0.985                       C
HETATM   21  C              0       2.398  -1.127   2.534                       C
HETATM   22  C              0       6.543   0.576   0.238                       C
HETATM   23  C              0       2.978  -1.707  -2.464                       C
HETATM   24  C              0      -1.922  -1.023  -2.614                       C
HETATM   25  C              0      -6.292  -0.399  -0.197                       C
HETATM   26  C              0      -2.392  -2.443   2.225                       C
HETATM   27  H              0       1.555  -3.852   0.700                       H
HETATM   28  H              0       1.686  -3.713  -1.071                       H
HETATM   29  H              0      -0.826  -4.053   0.483                       H
HETATM   30  H              0      -0.705  -3.826  -1.280                       H
HETATM   31  H              0       4.842   0.022   2.301                       H
HETATM   32  H              0       5.312  -0.463  -1.958                       H
HETATM   33  H              0      -4.524  -0.353  -2.275                       H
HETATM   34  H              0      -4.906  -1.527   1.857                       H
HETATM   35  H              0       1.490  -0.496   2.593                       H
HETATM   36  H              0       3.006  -0.909   3.427                       H
HETATM   37  H              0       2.042  -2.171   2.620                       H
HETATM   38  H              0       7.015   0.475   1.230                       H
HETATM   39  H              0       6.437   1.661   0.045                       H
HETATM   40  H              0       7.248   0.191  -0.517                       H
HETATM   41  H              0       3.334  -1.069  -3.290                       H
HETATM   42  H              0       1.879  -1.734  -2.532                       H
HETATM   43  H              0       3.363  -2.729  -2.656                       H
HETATM   44  H              0      -1.140  -0.243  -2.537                       H
HETATM   45  H              0      -2.557  -0.768  -3.478                       H
HETATM   46  H              0      -1.390  -1.963  -2.854                       H
HETATM   47  H              0      -6.369   0.621   0.225                       H
HETATM   48  H              0      -6.939  -1.044   0.423                       H
HETATM   49  H              0      -6.720  -0.359  -1.212                       H
HETATM   50  H              0      -2.880  -2.041   3.128                       H
HETATM   51  H              0      -1.312  -2.253   2.331                       H
HETATM   52  H              0      -2.551  -3.540   2.234                       H
HETATM   53  C              0      -1.417   2.131  -0.514                       C
HETATM   54  F              0      -2.584   1.830   0.067                       F
HETATM   55  F              0      -1.609   2.302  -1.818                       F
HETATM   56  C              0       1.447   2.071   0.614                       C
HETATM   57  C              0      -0.558   3.055   0.191                       C
HETATM   58  F              0      -0.995   3.605   1.315                       F
HETATM   59  F              0       0.062   3.963  -0.565                       F
HETATM   60  H              0       1.931   2.788  -0.071                       H
HETATM   61  H              0       1.908   1.942   1.617                       H
END
```

a2j.pdb

```
TITLE       a2j.pdb
HETATM    1 Ru              0       0.066   0.660   0.012                      Ru
HETATM    2  C              0      -0.554  -3.469   0.201                       C
HETATM    3  C              0      -2.027  -3.055   0.161                       C
HETATM    4  H              0      -0.291  -4.041   1.111                       H
HETATM    5  H              0      -2.584  -3.356   1.068                       H
HETATM    6  C              0      -0.697  -1.118   0.082                       C
```

```
HETATM    7  N             0       -1.966  -1.596   0.066                          N
HETATM    8  N             0        0.143  -2.190   0.182                          N
HETATM    9 Cl             0        0.590   0.758   2.315                         Cl
HETATM   10 Cl             0        0.650   0.684  -2.279                         Cl
HETATM   11  C             0        2.842   2.428   0.004                          C
HETATM   12  C             0       -3.158  -0.821   0.012                          C
HETATM   13  C             0       -3.704  -0.490  -1.241                          C
HETATM   14  C             0       -3.757  -0.391   1.209                          C
HETATM   15  C             0       -4.880   0.266  -1.276                          C
HETATM   16  C             0       -4.933   0.362   1.132                          C
HETATM   17  C             0       -5.507   0.699  -0.100                          C
HETATM   18  H             0       -5.312   0.531  -2.250                          H
HETATM   19  H             0       -5.407   0.705   2.061                          H
HETATM   20  C             0        1.562  -2.057   0.078                          C
HETATM   21  C             0        2.160  -2.048  -1.200                          C
HETATM   22  C             0        2.345  -1.929   1.244                          C
HETATM   23  C             0        3.519  -1.741  -1.297                          C
HETATM   24  C             0        3.702  -1.618   1.099                          C
HETATM   25  C             0        4.292  -1.477  -0.161                          C
HETATM   26  H             0        3.980  -1.689  -2.292                          H
HETATM   27  H             0        4.309  -1.472   2.002                          H
HETATM   28  C             0       -2.993  -0.884  -2.499                          C
HETATM   29  H             0       -3.583  -0.625  -3.393                          H
HETATM   30  H             0       -2.014  -0.371  -2.580                          H
HETATM   31  H             0       -2.778  -1.968  -2.541                          H
HETATM   32  C             0       -3.102  -0.678   2.525                          C
HETATM   33  H             0       -2.926  -1.758   2.685                          H
HETATM   34  H             0       -2.110  -0.190   2.591                          H
HETATM   35  H             0       -3.713  -0.313   3.367                          H
HETATM   36  C             0       -6.749   1.536  -0.165                          C
HETATM   37  H             0       -6.506   2.605  -0.316                          H
HETATM   38  H             0       -7.402   1.242  -1.005                          H
HETATM   39  H             0       -7.339   1.472   0.765                          H
HETATM   40  C             0        1.386  -2.433  -2.424                          C
HETATM   41  H             0        1.797  -1.955  -3.327                          H
HETATM   42  H             0        1.435  -3.529  -2.582                          H
HETATM   43  H             0        0.322  -2.150  -2.365                          H
HETATM   44  C             0        1.783  -2.205   2.604                          C
HETATM   45  H             0        2.013  -3.247   2.903                          H
HETATM   46  H             0        2.223  -1.542   3.367                          H
HETATM   47  H             0        0.692  -2.073   2.650                          H
HETATM   48  C             0        5.717  -1.033  -0.291                          C
HETATM   49  H             0        5.778   0.071  -0.360                          H
HETATM   50  H             0        6.327  -1.330   0.579                          H
HETATM   51  H             0        6.195  -1.433  -1.202                          H
HETATM   52  H             0       -2.572  -3.472  -0.707                          H
HETATM   53  H             0       -0.259  -4.084  -0.671                          H
HETATM   54  C             0        1.865   3.342  -0.021                          C
HETATM   55  C             0       -1.363   1.763  -0.025                          C
HETATM   56  H             0       -2.445   1.536  -0.027                          H
HETATM   57  H             0       -1.111   2.851  -0.047                          H
HETATM   58  F             0        1.378   3.851  -1.129                          F
HETATM   59  F             0        1.357   3.884   1.063                          F
HETATM   60  F             0        3.397   1.938  -1.076                          F
HETATM   61  F             0        3.374   1.975   1.110                          F
END
```

s2k.pdb

```
TITLE      s2k.pdb
HETATM    1 Ru             0        0.113  -0.961  -0.252                         Ru
HETATM    2  C             0       -0.078   2.977   1.153                          C
```

```
HETATM    3  C              0      -1.578   2.693   1.189                      C
HETATM    4  H              0       0.191   3.737   0.392                      H
HETATM    5  H              0      -2.175   3.428   0.621                      H
HETATM    6  C              0      -0.468   0.799   0.377                      C
HETATM    7  N              0      -1.682   1.369   0.571                      N
HETATM    8  N              0       0.485   1.678   0.791                      N
HETATM    9 Cl              0       0.552  -0.462  -2.574                     Cl
HETATM   10 Cl              0      -0.088  -1.873   1.991                     Cl
HETATM   11  C              0       2.682  -2.473  -0.325                      C
HETATM   12  C              0      -2.944   0.794   0.245                      C
HETATM   13  C              0      -3.716   0.177   1.248                      C
HETATM   14  C              0      -3.413   0.892  -1.082                      C
HETATM   15  C              0      -4.962  -0.353   0.896                      C
HETATM   16  C              0      -4.663   0.344  -1.390                      C
HETATM   17  C              0      -5.447  -0.285  -0.416                      C
HETATM   18  H              0      -5.566  -0.844   1.670                      H
HETATM   19  H              0      -5.033   0.416  -2.421                      H
HETATM   20  C              0       1.870   1.486   0.484                      C
HETATM   21  C              0       2.732   0.953   1.467                      C
HETATM   22  C              0       2.361   1.879  -0.779                      C
HETATM   23  C              0       4.075   0.748   1.129                      C
HETATM   24  C              0       3.709   1.653  -1.071                      C
HETATM   25  C              0       4.574   1.074  -0.137                      C
HETATM   26  H              0       4.749   0.322   1.883                      H
HETATM   27  H              0       4.089   1.934  -2.061                      H
HETATM   28  C              0      -3.197   0.052   2.646                      C
HETATM   29  H              0      -3.863  -0.574   3.262                      H
HETATM   30  H              0      -2.191  -0.408   2.661                      H
HETATM   31  H              0      -3.116   1.032   3.154                      H
HETATM   32  C              0      -2.578   1.549  -2.136                      C
HETATM   33  H              0      -2.221   2.547  -1.819                      H
HETATM   34  H              0      -1.673   0.953  -2.372                      H
HETATM   35  H              0      -3.145   1.676  -3.073                      H
HETATM   36  C              0      -6.767  -0.894  -0.779                      C
HETATM   37  H              0      -6.639  -1.916  -1.185                      H
HETATM   38  H              0      -7.437  -0.980   0.093                      H
HETATM   39  H              0      -7.290  -0.312  -1.556                      H
HETATM   40  C              0       2.237   0.639   2.843                      C
HETATM   41  H              0       3.071   0.357   3.508                      H
HETATM   42  H              0       1.722   1.503   3.301                      H
HETATM   43  H              0       1.507  -0.193   2.837                      H
HETATM   44  C              0       1.489   2.577  -1.778                      C
HETATM   45  H              0       1.551   3.677  -1.656                      H
HETATM   46  H              0       1.802   2.341  -2.807                      H
HETATM   47  H              0       0.431   2.282  -1.696                      H
HETATM   48  C              0       5.998   0.777  -0.495                      C
HETATM   49  H              0       6.087  -0.224  -0.958                      H
HETATM   50  H              0       6.401   1.499  -1.225                      H
HETATM   51  H              0       6.657   0.777   0.390                      H
HETATM   52  H              0      -1.978   2.668   2.221                      H
HETATM   53  H              0       0.322   3.322   2.122                      H
HETATM   54  C              0       1.536  -3.068  -0.693                      C
HETATM   55  H              0       1.269  -3.082  -1.753                      H
HETATM   56  C              0      -1.550  -1.563  -0.647                      C
HETATM   57  H              0      -2.238  -1.975   0.121                      H
HETATM   58  H              0      -1.920  -1.586  -1.696                      H
HETATM   59  H              0       0.995  -3.667   0.043                      H
HETATM   60  F              0       3.470  -1.819  -1.133                      F
HETATM   61  F              0       3.167  -2.473   0.885                      F
END
```

s2l.pdb

```
TITLE       s21.pdb
HETATM    1 Ru           0       0.154  -0.908  -0.130                       Ru
HETATM    2 Cl           0      -0.620  -1.389   2.134                       Cl
HETATM    3 Cl           0       0.861  -0.602  -2.440                       Cl
HETATM    4  C           0      -0.302   1.027   0.188                        C
HETATM    5  N           0      -1.491   1.655   0.259                        N
HETATM    6  C           0      -1.346   3.070   0.621                        C
HETATM    7  C           0       0.174   3.257   0.715                        C
HETATM    8  N           0       0.679   1.905   0.475                        N
HETATM    9  C           0      -2.751   1.021   0.054                        C
HETATM   10  C           0      -3.546   0.654   1.155                        C
HETATM   11  C           0      -4.749  -0.018   0.910                        C
HETATM   12  C           0      -5.172  -0.325  -0.387                        C
HETATM   13  C           0      -4.377   0.084  -1.465                        C
HETATM   14  C           0      -3.171   0.763  -1.268                        C
HETATM   15  C           0       2.053   1.535   0.327                        C
HETATM   16  C           0       2.695   1.746  -0.911                        C
HETATM   17  C           0       3.996   1.267  -1.075                        C
HETATM   18  C           0       4.666   0.595  -0.047                        C
HETATM   19  C           0       4.028   0.452   1.189                        C
HETATM   20  C           0       2.727   0.921   1.404                        C
HETATM   21  C           0      -3.142   0.988   2.558                        C
HETATM   22  C           0      -6.440  -1.090  -0.618                        C
HETATM   23  C           0      -2.342   1.213  -2.431                        C
HETATM   24  C           0       2.019   2.485  -2.024                        C
HETATM   25  C           0       6.029   0.016  -0.275                        C
HETATM   26  C           0       2.090   0.785   2.754                        C
HETATM   27  H           0      -1.860   3.273   1.578                        H
HETATM   28  H           0      -1.815   3.710  -0.149                        H
HETATM   29  H           0       0.505   3.627   1.702                        H
HETATM   30  H           0       0.568   3.954  -0.049                        H
HETATM   31  H           0      -5.367  -0.319   1.766                        H
HETATM   32  H           0      -4.708  -0.121  -2.492                        H
HETATM   33  H           0       4.489   1.399  -2.047                        H
HETATM   34  H           0       4.556  -0.038   2.018                        H
HETATM   35  H           0      -3.544   1.972   2.870                        H
HETATM   36  H           0      -2.048   1.006   2.681                        H
HETATM   37  H           0      -3.532   0.242   3.270                        H
HETATM   38  H           0      -7.177  -0.923   0.186                        H
HETATM   39  H           0      -6.915  -0.822  -1.578                        H
HETATM   40  H           0      -6.251  -2.180  -0.653                        H
HETATM   41  H           0      -2.877   1.062  -3.383                        H
HETATM   42  H           0      -1.379   0.668  -2.499                        H
HETATM   43  H           0      -2.085   2.286  -2.358                        H
HETATM   44  H           0       0.928   2.326  -2.033                        H
HETATM   45  H           0       2.396   2.152  -3.004                        H
HETATM   46  H           0       2.203   3.576  -1.953                        H
HETATM   47  H           0       5.956  -1.020  -0.660                        H
HETATM   48  H           0       6.604   0.590  -1.022                        H
HETATM   49  H           0       6.621  -0.032   0.654                        H
HETATM   50  H           0       2.854   0.620   3.531                        H
HETATM   51  H           0       1.376  -0.060   2.800                        H
HETATM   52  H           0       1.512   1.685   3.033                        H
HETATM   53  C           0      -1.332  -1.812  -0.736                        C
HETATM   54  H           0      -2.094  -2.229  -0.043                        H
HETATM   55  H           0      -1.505  -1.977  -1.821                        H
HETATM   56  C           0       1.666  -2.583   0.040                        C
HETATM   57  C           0       0.525  -3.227  -0.425                        C
HETATM   58  F           0       2.066  -2.677   1.299                        F
HETATM   59  F           0       2.715  -2.351  -0.730                        F
HETATM   60  H           0      -0.073  -3.792   0.296                        H
```

```
HETATM   61  H             0        0.484  -3.478  -1.489                       H
END
```

s2PB.pdb

```
TITLE       s2PB.pdb
HETATM    1 Ru             0       -0.033  -0.964   0.195                      Ru
HETATM    2  C             0        1.145   3.165  -0.193                       C
HETATM    3  C             0       -0.373   3.267  -0.374                       C
HETATM    4  H             0        1.711   3.551  -1.059                       H
HETATM    5  H             0       -0.668   3.635  -1.375                       H
HETATM    6  C             0        0.213   1.038  -0.035                       C
HETATM    7  N             0       -0.817   1.881  -0.197                       N
HETATM    8  N             0        1.368   1.722  -0.047                       N
HETATM    9 Cl             0        1.025  -1.351  -1.957                      Cl
HETATM   10 Cl             0       -0.843  -0.624   2.455                      Cl
HETATM   11  C             0        1.059  -2.499   0.854                       C
HETATM   12  H             0        1.259  -2.474   1.936                       H
HETATM   13  C             0       -2.179   1.450  -0.246                       C
HETATM   14  C             0       -3.002   1.627   0.883                       C
HETATM   15  C             0       -2.666   0.847  -1.423                       C
HETATM   16  C             0       -4.306   1.126   0.836                       C
HETATM   17  C             0       -3.979   0.362  -1.423                       C
HETATM   18  C             0       -4.803   0.475  -0.299                       C
HETATM   19  H             0       -4.946   1.235   1.721                       H
HETATM   20  H             0       -4.364  -0.117  -2.333                       H
HETATM   21  C             0        2.665   1.124   0.019                       C
HETATM   22  C             0        3.175   0.713   1.264                       C
HETATM   23  C             0        3.405   0.958  -1.167                       C
HETATM   24  C             0        4.424   0.082   1.296                       C
HETATM   25  C             0        4.651   0.329  -1.088                       C
HETATM   26  C             0        5.167  -0.129   0.129                       C
HETATM   27  H             0        4.828  -0.244   2.263                       H
HETATM   28  H             0        5.225   0.176  -2.011                       H
HETATM   29  C             0       -2.511   2.346   2.101                       C
HETATM   30  H             0       -3.031   1.993   3.006                       H
HETATM   31  H             0       -1.433   2.189   2.268                       H
HETATM   32  H             0       -2.692   3.437   2.027                       H
HETATM   33  C             0       -1.817   0.730  -2.651                       C
HETATM   34  H             0       -1.209   1.636  -2.826                       H
HETATM   35  H             0       -1.100  -0.112  -2.594                       H
HETATM   36  H             0       -2.441   0.566  -3.545                       H
HETATM   37  C             0       -6.182  -0.110  -0.298                       C
HETATM   38  H             0       -6.176  -1.133   0.124                       H
HETATM   39  H             0       -6.882   0.481   0.317                       H
HETATM   40  H             0       -6.599  -0.188  -1.316                       H
HETATM   41  C             0        2.410   0.954   2.529                       C
HETATM   42  H             0        3.030   0.738   3.414                       H
HETATM   43  H             0        2.065   2.002   2.609                       H
HETATM   44  H             0        1.496   0.331   2.604                       H
HETATM   45  C             0        2.886   1.444  -2.485                       C
HETATM   46  H             0        3.153   2.505  -2.665                       H
HETATM   47  H             0        3.313   0.861  -3.317                       H
HETATM   48  H             0        1.789   1.348  -2.555                       H
HETATM   49  C             0        6.481  -0.849   0.178                       C
HETATM   50  H             0        6.350  -1.931  -0.015                       H
HETATM   51  H             0        7.186  -0.477  -0.585                       H
HETATM   52  H             0        6.966  -0.759   1.165                       H
HETATM   53  H             0       -0.855   3.920   0.375                       H
HETATM   54  H             0        1.510   3.695   0.706                       H
HETATM   55  C             0       -0.257  -3.170   0.460                       C
```

```
HETATM   56  H              0       -0.077  -3.890  -0.355                         H
HETATM   57  H              0       -0.731  -3.613   1.351                         H
HETATM   58  C              0       -1.439  -2.234  -0.143                         C
HETATM   59  H              0        1.920  -2.757   0.220                         H
HETATM   60  F              0       -2.557  -2.312   0.583                         F
HETATM   61  F              0       -1.787  -2.586  -1.389                         F
END
```

s2m.pdb

```
TITLE       s2m.pdb
HETATM    1 Ru              0        0.114  -1.017   0.206                        Ru
HETATM    2 Cl              0        1.115  -1.361  -1.977                        Cl
HETATM    3 Cl              0       -0.651  -0.704   2.490                        Cl
HETATM    4  C              0        0.135   1.030  -0.017                         C
HETATM    5  N              0        1.252   1.779   0.000                         N
HETATM    6  C              0        0.968   3.212  -0.120                         C
HETATM    7  C              0       -0.558   3.249  -0.246                         C
HETATM    8  N              0       -0.933   1.833  -0.141                         N
HETATM    9  C              0        2.569   1.222   0.016                         C
HETATM   10  C              0        3.268   1.070  -1.197                         C
HETATM   11  C              0        4.521   0.454  -1.169                         C
HETATM   12  C              0        5.090  -0.001   0.026                         C
HETATM   13  C              0        4.394   0.208   1.222                         C
HETATM   14  C              0        3.139   0.826   1.243                         C
HETATM   15  C              0       -2.282   1.370  -0.205                         C
HETATM   16  C              0       -3.112   1.479   0.925                         C
HETATM   17  C              0       -4.417   0.980   0.840                         C
HETATM   18  C              0       -4.905   0.394  -0.332                         C
HETATM   19  C              0       -4.066   0.330  -1.451                         C
HETATM   20  C              0       -2.756   0.815  -1.411                         C
HETATM   21  C              0        2.698   1.558  -2.494                         C
HETATM   22  C              0        6.409  -0.711   0.020                         C
HETATM   23  C              0        2.438   1.080   2.542                         C
HETATM   24  C              0       -2.635   2.130   2.187                         C
HETATM   25  C              0       -6.288  -0.181  -0.382                         C
HETATM   26  C              0       -1.879   0.745  -2.625                         C
HETATM   27  H              0        1.485   3.630  -1.002                         H
HETATM   28  H              0        1.342   3.752   0.770                         H
HETATM   29  H              0       -0.905   3.662  -1.211                         H
HETATM   30  H              0       -1.045   3.831   0.557                         H
HETATM   31  H              0        5.059   0.308  -2.114                         H
HETATM   32  H              0        4.843  -0.109   2.173                         H
HETATM   33  H              0       -5.065   1.043   1.724                         H
HETATM   34  H              0       -4.440  -0.108  -2.385                         H
HETATM   35  H              0        3.016   2.598  -2.709                         H
HETATM   36  H              0        1.596   1.532  -2.501                         H
HETATM   37  H              0        3.039   0.934  -3.335                         H
HETATM   38  H              0        7.090  -0.313  -0.751                         H
HETATM   39  H              0        6.280  -1.788  -0.201                         H
HETATM   40  H              0        6.920  -0.645   0.996                         H
HETATM   41  H              0        3.105   0.874   3.395                         H
HETATM   42  H              0        1.532   0.456   2.670                         H
HETATM   43  H              0        2.099   2.129   2.629                         H
HETATM   44  H              0       -1.548   2.016   2.327                         H
HETATM   45  H              0       -3.119   1.681   3.070                         H
HETATM   46  H              0       -2.875   3.212   2.201                         H
HETATM   47  H              0       -6.278  -1.265  -0.158                         H
HETATM   48  H              0       -6.746  -0.070  -1.379                         H
HETATM   49  H              0       -6.958   0.289   0.358                         H
HETATM   50  H              0       -2.462   0.466  -3.517                         H
```

```
HETATM   51  H            0       -1.064   0.002  -2.518                      H
HETATM   52  H            0       -1.387   1.713  -2.836                      H
HETATM   53  C            0        1.264  -2.624   0.860                      C
HETATM   54  H            0        2.138  -2.730   0.201                      H
HETATM   55  H            0        1.466  -2.507   1.935                      H
HETATM   56  C            0       -1.389  -2.046  -0.183                      C
HETATM   57  C            0        0.029  -3.253   0.464                      C
HETATM   58  F            0       -1.738  -2.472  -1.387                      F
HETATM   59  F            0       -2.434  -2.257   0.597                      F
HETATM   60  H            0        0.062  -3.851  -0.458                      H
HETATM   61  H            0       -0.594  -3.651   1.278                      H
END
```

s2n.pdb

```
TITLE       s2n.pdb
HETATM    1 Ru            0        0.390  -0.978   0.190                     Ru
HETATM    2  C            0        0.591   3.216  -0.114                      C
HETATM    3  C            0       -0.936   3.126  -0.065                      C
HETATM    4  H            0        0.965   3.594  -1.086                      H
HETATM    5  H            0       -1.428   3.587  -0.940                      H
HETATM    6  C            0       -0.052   0.962   0.041                      C
HETATM    7  N            0       -1.188   1.682  -0.045                      N
HETATM    8  N            0        0.997   1.825   0.078                      N
HETATM    9 Cl            0        0.857  -1.384  -2.161                     Cl
HETATM   10 Cl            0        0.250  -0.856   2.611                     Cl
HETATM   11  C            0        1.996  -2.711   0.535                      C
HETATM   12  C            0       -2.510   1.151  -0.123                      C
HETATM   13  C            0       -3.304   1.088   1.037                      C
HETATM   14  C            0       -3.000   0.722  -1.371                      C
HETATM   15  C            0       -4.602   0.584   0.926                      C
HETATM   16  C            0       -4.300   0.208  -1.434                      C
HETATM   17  C            0       -5.113   0.133  -0.297                      C
HETATM   18  H            0       -5.227   0.526   1.827                      H
HETATM   19  H            0       -4.688  -0.135  -2.402                      H
HETATM   20  C            0        2.339   1.341  -0.036                      C
HETATM   21  C            0        3.111   1.144   1.131                      C
HETATM   22  C            0        2.882   1.091  -1.316                      C
HETATM   23  C            0        4.375   0.557   0.997                      C
HETATM   24  C            0        4.150   0.508  -1.399                      C
HETATM   25  C            0        4.894   0.204  -0.254                      C
HETATM   26  H            0        4.975   0.385   1.900                      H
HETATM   27  H            0        4.562   0.282  -2.391                      H
HETATM   28  C            0       -2.742   1.477   2.369                      C
HETATM   29  H            0       -3.487   1.344   3.170                      H
HETATM   30  H            0       -1.857   0.862   2.623                      H
HETATM   31  H            0       -2.413   2.533   2.401                      H
HETATM   32  C            0       -2.139   0.787  -2.594                      C
HETATM   33  H            0       -1.658   1.776  -2.712                      H
HETATM   34  H            0       -1.315   0.046  -2.560                      H
HETATM   35  H            0       -2.725   0.588  -3.506                      H
HETATM   36  C            0       -6.493  -0.447  -0.380                      C
HETATM   37  H            0       -6.482  -1.537  -0.188                      H
HETATM   38  H            0       -7.173  -0.002   0.366                      H
HETATM   39  H            0       -6.941  -0.307  -1.379                      H
HETATM   40  C            0        2.625   1.608   2.468                      C
HETATM   41  H            0        3.378   1.416   3.249                      H
HETATM   42  H            0        2.421   2.695   2.464                      H
HETATM   43  H            0        1.692   1.098   2.767                      H
HETATM   44  C            0        2.164   1.507  -2.563                      C
HETATM   45  H            0        2.432   2.549  -2.833                      H
HETATM   46  H            0        2.439   0.864  -3.414                      H
```

```
HETATM   47  H            0       1.069   1.451  -2.465                       H
HETATM   48  C            0       6.218  -0.488  -0.366                       C
HETATM   49  H            0       6.089  -1.588  -0.380                       H
HETATM   50  H            0       6.748  -0.223  -1.297                       H
HETATM   51  H            0       6.880  -0.257   0.485                       H
HETATM   52  H            0      -1.361   3.601   0.839                       H
HETATM   53  H            0       1.012   3.863   0.676                       H
HETATM   54  C            0       0.831  -3.410   0.473                       C
HETATM   55  H            0       0.475  -3.829  -0.477                       H
HETATM   56  C            0      -1.306  -1.665   0.057                       C
HETATM   57  H            0       2.409  -2.394   1.502                       H
HETATM   58  H            0       0.279  -3.671   1.386                       H
HETATM   59  H            0       2.610  -2.560  -0.363                       H
HETATM   60  F            0      -1.805  -2.337  -0.960                       F
HETATM   61  F            0      -2.237  -1.680   0.983                       F
END
```

a2k.pdb

```
TITLE      a2k.pdb
HETATM    1 Ru            0      -0.264   0.867   0.062                      Ru
HETATM    2  C            0      -0.350  -3.333   0.224                       C
HETATM    3  C            0       1.169  -3.180   0.310                       C
HETATM    4  H            0      -0.676  -3.814  -0.719                       H
HETATM    5  H            0       1.708  -3.735  -0.478                       H
HETATM    6  C            0       0.203  -1.053   0.140                       C
HETATM    7  N            0       1.370  -1.737   0.143                       N
HETATM    8  N            0      -0.812  -1.949   0.272                       N
HETATM    9 Cl            0      -0.679   1.061  -2.320                      Cl
HETATM   10 Cl            0      -0.141   0.978   2.489                      Cl
HETATM   11  C            0      -1.852   2.709   0.180                       C
HETATM   12  C            0       2.673  -1.181  -0.006                       C
HETATM   13  C            0       3.462  -0.932   1.133                       C
HETATM   14  C            0       3.152  -0.903  -1.302                       C
HETATM   15  C            0       4.741  -0.395   0.951                       C
HETATM   16  C            0       4.436  -0.364  -1.437                       C
HETATM   17  C            0       5.241  -0.101  -0.323                       C
HETATM   18  H            0       5.359  -0.189   1.835                       H
HETATM   19  H            0       4.815  -0.145  -2.444                       H
HETATM   20  C            0      -2.178  -1.561   0.095                       C
HETATM   21  C            0      -2.983  -1.314   1.230                       C
HETATM   22  C            0      -2.712  -1.470  -1.208                       C
HETATM   23  C            0      -4.291  -0.861   1.029                       C
HETATM   24  C            0      -4.027  -1.017  -1.359                       C
HETATM   25  C            0      -4.821  -0.686  -0.256                       C
HETATM   26  H            0      -4.919  -0.657   1.905                       H
HETATM   27  H            0      -4.441  -0.925  -2.371                       H
HETATM   28  C            0       2.918  -1.178   2.506                       C
HETATM   29  H            0       3.621  -0.830   3.280                       H
HETATM   30  H            0       1.958  -0.646   2.655                       H
HETATM   31  H            0       2.725  -2.250   2.700                       H
HETATM   32  C            0       2.290  -1.155  -2.499                       C
HETATM   33  H            0       1.878  -2.182  -2.507                       H
HETATM   34  H            0       1.419  -0.469  -2.523                       H
HETATM   35  H            0       2.853  -1.012  -3.435                       H
HETATM   36  C            0       6.599   0.512  -0.491                       C
HETATM   37  H            0       6.536   1.616  -0.542                       H
HETATM   38  H            0       7.269   0.270   0.350                       H
HETATM   39  H            0       7.087   0.182  -1.424                       H
HETATM   40  C            0      -2.475  -1.589   2.609                       C
HETATM   41  H            0      -3.237  -1.345   3.367                       H
HETATM   42  H            0      -2.214  -2.656   2.737                       H
```

```
HETATM   43  H              0       -1.570  -0.999   2.837                     H
HETATM   44  C              0       -1.937  -1.922  -2.408                     C
HETATM   45  H              0       -2.142  -2.991  -2.620                     H
HETATM   46  H              0       -2.219  -1.347  -3.303                     H
HETATM   47  H              0       -0.849  -1.801  -2.286                     H
HETATM   48  C              0       -6.210  -0.156  -0.444                     C
HETATM   49  H              0       -6.208   0.946  -0.544                     H
HETATM   50  H              0       -6.683  -0.553  -1.358                     H
HETATM   51  H              0       -6.863  -0.395   0.413                     H
HETATM   52  H              0        1.574  -3.514   1.284                     H
HETATM   53  H              0       -0.774  -3.919   1.058                     H
HETATM   54  C              0       -0.834   3.595   0.114                     C
HETATM   55  C              0        1.422   1.520  -0.107                     C
HETATM   56  H              0        2.195   1.427   0.684                     H
HETATM   57  H              0        1.717   2.104  -1.008                     H
HETATM   58  H              0       -2.265   2.471   1.164                     H
HETATM   59  H              0       -2.394   2.472  -0.739                     H
HETATM   60  F              0       -0.368   4.112  -0.988                     F
HETATM   61  F              0       -0.230   4.111   1.149                     F
END
```

a2l.pdb

```
TITLE      a2l.pdb
HETATM    1 Ru              0       -0.119   0.897   0.039                    Ru
HETATM    2 Cl              0       -0.993   0.983  -2.224                    Cl
HETATM    3 Cl              0        0.667   0.910   2.338                    Cl
HETATM    4  C              0        0.065  -1.129   0.026                     C
HETATM    5  N              0       -0.997  -1.945   0.162                     N
HETATM    6  C              0       -0.617  -3.361   0.158                     C
HETATM    7  C              0        0.908  -3.304   0.026                     C
HETATM    8  N              0        1.186  -1.865  -0.047                     N
HETATM    9  C              0       -2.342  -1.461   0.140                     C
HETATM   10  C              0       -3.047  -1.427  -1.080                     C
HETATM   11  C              0       -4.329  -0.872  -1.091                     C
HETATM   12  C              0       -4.922  -0.367   0.073                     C
HETATM   13  C              0       -4.220  -0.464   1.279                     C
HETATM   14  C              0       -2.935  -1.016   1.339                     C
HETATM   15  C              0        2.505  -1.327  -0.137                     C
HETATM   16  C              0        3.312  -1.262   1.012                     C
HETATM   17  C              0        4.582  -0.686   0.898                     C
HETATM   18  C              0        5.058  -0.188  -0.319                     C
HETATM   19  C              0        4.243  -0.296  -1.453                     C
HETATM   20  C              0        2.968  -0.865  -1.385                     C
HETATM   21  C              0       -2.462  -1.996  -2.337                     C
HETATM   22  C              0       -6.278   0.269   0.019                     C
HETATM   23  C              0       -2.229  -1.152   2.653                     C
HETATM   24  C              0        2.852  -1.813   2.327                     C
HETATM   25  C              0        6.404   0.467  -0.405                     C
HETATM   26  C              0        2.111  -0.975  -2.608                     C
HETATM   27  H              0       -1.105  -3.881  -0.688                     H
HETATM   28  H              0       -0.956  -3.854   1.088                     H
HETATM   29  H              0        1.285  -3.810  -0.882                     H
HETATM   30  H              0        1.430  -3.748   0.894                     H
HETATM   31  H              0       -4.873  -0.819  -2.043                     H
HETATM   32  H              0       -4.687  -0.109   2.207                     H
HETATM   33  H              0        5.213  -0.617   1.794                     H
HETATM   34  H              0        4.611   0.067  -2.422                     H
HETATM   35  H              0       -2.738  -3.063  -2.458                     H
HETATM   36  H              0       -1.363  -1.923  -2.359                     H
HETATM   37  H              0       -2.834  -1.459  -3.224                     H
HETATM   38  H              0       -6.969  -0.289  -0.636                     H
```

```
HETATM   39  H             0       -6.221   1.295  -0.389                      H
HETATM   40  H             0       -6.739   0.344   1.018                      H
HETATM   41  H             0       -2.914  -0.940   3.491                      H
HETATM   42  H             0       -1.365  -0.465   2.745                      H
HETATM   43  H             0       -1.827  -2.171   2.801                      H
HETATM   44  H             0        1.760  -1.729   2.455                      H
HETATM   45  H             0        3.315  -1.269   3.167                      H
HETATM   46  H             0        3.134  -2.879   2.440                      H
HETATM   47  H             0        7.104   0.074   0.351                      H
HETATM   48  H             0        6.329   1.557  -0.230                      H
HETATM   49  H             0        6.864   0.338  -1.399                      H
HETATM   50  H             0        2.692  -0.766  -3.521                      H
HETATM   51  H             0        1.256  -0.269  -2.590                      H
HETATM   52  H             0        1.669  -1.984  -2.715                      H
HETATM   53  C             0        1.325   1.953  -0.430                      C
HETATM   54  H             0        1.482   2.256  -1.484                      H
HETATM   55  H             0        2.095   2.249   0.309                      H
HETATM   56  C             0       -1.435   2.495   0.499                      C
HETATM   57  H             0       -2.261   2.462  -0.223                      H
HETATM   58  H             0       -1.657   2.438   1.572                      H
HETATM   59  C             0       -0.306   3.269   0.130                      C
HETATM   60  F             0       -0.321   3.960  -1.000                      F
HETATM   61  F             0        0.370   3.933   1.055                      F
END
```

a2NB.pdb

```
TITLE      a2NB.pdb
HETATM    1 Ru             0        0.223   0.833   0.151                     Ru
HETATM    2  C             0        0.384  -3.334  -0.401                      C
HETATM    3  C             0       -1.067  -3.229   0.067                      C
HETATM    4  H             0        0.988  -4.031   0.204                      H
HETATM    5  H             0       -1.220  -3.631   1.089                      H
HETATM    6  C             0       -0.153  -1.105  -0.033                      C
HETATM    7  N             0       -1.311  -1.781   0.066                      N
HETATM    8  N             0        0.863  -1.960  -0.238                      N
HETATM    9 Cl             0        1.322   1.215   2.197                     Cl
HETATM   10 Cl             0       -0.927   0.610  -2.058                     Cl
HETATM   11  C             0        1.387   2.118  -0.911                      C
HETATM   12  H             0        1.401   1.979  -2.001                      H
HETATM   13  C             0       -2.608  -1.201   0.203                      C
HETATM   14  C             0       -3.532  -1.324  -0.852                      C
HETATM   15  C             0       -2.943  -0.520   1.386                      C
HETATM   16  C             0       -4.789  -0.731  -0.706                      C
HETATM   17  C             0       -4.209   0.069   1.486                      C
HETATM   18  C             0       -5.140  -0.021   0.448                      C
HETATM   19  H             0       -5.508  -0.809  -1.531                      H
HETATM   20  H             0       -4.473   0.602   2.408                      H
HETATM   21  C             0        2.231  -1.541  -0.178                      C
HETATM   22  C             0        2.883  -1.099  -1.347                      C
HETATM   23  C             0        2.890  -1.567   1.066                      C
HETATM   24  C             0        4.198  -0.635  -1.232                      C
HETATM   25  C             0        4.203  -1.098   1.133                      C
HETATM   26  C             0        4.865  -0.614  -0.002                      C
HETATM   27  H             0        4.714  -0.280  -2.133                      H
HETATM   28  H             0        4.715  -1.092   2.103                      H
HETATM   29  C             0       -3.190  -2.054  -2.115                      C
HETATM   30  H             0       -3.745  -1.638  -2.972                      H
HETATM   31  H             0       -2.116  -1.974  -2.352                      H
HETATM   32  H             0       -3.455  -3.129  -2.059                      H
HETATM   33  C             0       -1.977  -0.424   2.528                      C
HETATM   34  H             0       -1.410  -1.360   2.680                      H
```

```
HETATM   35  H              0      -1.219   0.373   2.390                       H
HETATM   36  H              0      -2.499  -0.187   3.470                       H
HETATM   37  C              0      -6.479   0.644   0.555                       C
HETATM   38  H              0      -6.497   1.598  -0.005                       H
HETATM   39  H              0      -7.286   0.019   0.134                       H
HETATM   40  H              0      -6.741   0.881   1.600                       H
HETATM   41  C              0       2.197  -1.142  -2.676                       C
HETATM   42  H              0       2.828  -0.699  -3.463                       H
HETATM   43  H              0       1.971  -2.181  -2.983                       H
HETATM   44  H              0       1.232  -0.600  -2.667                       H
HETATM   45  C              0       2.197  -2.083   2.290                       C
HETATM   46  H              0       2.067  -3.183   2.260                       H
HETATM   47  H              0       2.772  -1.846   3.199                       H
HETATM   48  H              0       1.198  -1.632   2.416                       H
HETATM   49  C              0       6.253  -0.059   0.105                       C
HETATM   50  H              0       6.229   1.014   0.374                       H
HETATM   51  H              0       6.842  -0.566   0.888                       H
HETATM   52  H              0       6.805  -0.137  -0.846                       H
HETATM   53  H              0      -1.776  -3.742  -0.603                       H
HETATM   54  H              0       0.470  -3.643  -1.461                       H
HETATM   55  C              0       0.364   3.193  -0.493                       C
HETATM   56  C              0      -0.863   2.551   0.188                       C
HETATM   57  H              0      -1.748   2.577  -0.462                       H
HETATM   58  H              0      -1.031   2.912   1.215                       H
HETATM   59  H              0       2.378   2.247  -0.447                       H
HETATM   60  F              0      -0.046   3.885  -1.598                       F
HETATM   61  F              0       0.949   4.104   0.341                       F
END
```

a2m.pdb

```
TITLE      a2m.pdb
HETATM    1 Ru              0      -0.119   0.897   0.039                      Ru
HETATM    2 Cl              0      -0.993   0.983  -2.224                      Cl
HETATM    3 Cl              0       0.667   0.910   2.338                      Cl
HETATM    4  C              0       0.065  -1.129   0.026                       C
HETATM    5  N              0      -0.997  -1.945   0.162                       N
HETATM    6  C              0      -0.617  -3.361   0.158                       C
HETATM    7  C              0       0.908  -3.304   0.026                       C
HETATM    8  N              0       1.186  -1.865  -0.047                       N
HETATM    9  C              0      -2.342  -1.461   0.140                       C
HETATM   10  C              0      -3.047  -1.427  -1.080                       C
HETATM   11  C              0      -4.329  -0.872  -1.091                       C
HETATM   12  C              0      -4.922  -0.367   0.073                       C
HETATM   13  C              0      -4.220  -0.464   1.279                       C
HETATM   14  C              0      -2.935  -1.016   1.339                       C
HETATM   15  C              0       2.505  -1.327  -0.137                       C
HETATM   16  C              0       3.312  -1.262   1.012                       C
HETATM   17  C              0       4.582  -0.686   0.898                       C
HETATM   18  C              0       5.058  -0.188  -0.319                       C
HETATM   19  C              0       4.243  -0.296  -1.453                       C
HETATM   20  C              0       2.968  -0.865  -1.385                       C
HETATM   21  C              0      -2.462  -1.996  -2.337                       C
HETATM   22  C              0      -6.278   0.269   0.019                       C
HETATM   23  C              0      -2.229  -1.152   2.653                       C
HETATM   24  C              0       2.852  -1.813   2.327                       C
HETATM   25  C              0       6.404   0.467  -0.405                       C
HETATM   26  C              0       2.111  -0.975  -2.608                       C
HETATM   27  H              0      -1.105  -3.881  -0.688                       H
HETATM   28  H              0      -0.956  -3.854   1.088                       H
HETATM   29  H              0       1.285  -3.810  -0.882                       H
HETATM   30  H              0       1.430  -3.748   0.894                       H
```

```
HETATM   31  H             0       -4.873  -0.819  -2.043                  H
HETATM   32  H             0       -4.687  -0.109   2.207                  H
HETATM   33  H             0        5.213  -0.617   1.794                  H
HETATM   34  H             0        4.611   0.067  -2.422                  H
HETATM   35  H             0       -2.738  -3.063  -2.458                  H
HETATM   36  H             0       -1.363  -1.923  -2.359                  H
HETATM   37  H             0       -2.834  -1.459  -3.224                  H
HETATM   38  H             0       -6.969  -0.289  -0.636                  H
HETATM   39  H             0       -6.221   1.295  -0.389                  H
HETATM   40  H             0       -6.739   0.344   1.018                  H
HETATM   41  H             0       -2.914  -0.940   3.491                  H
HETATM   42  H             0       -1.365  -0.465   2.745                  H
HETATM   43  H             0       -1.827  -2.171   2.801                  H
HETATM   44  H             0        1.760  -1.729   2.455                  H
HETATM   45  H             0        3.315  -1.269   3.167                  H
HETATM   46  H             0        3.134  -2.879   2.440                  H
HETATM   47  H             0        7.104   0.074   0.351                  H
HETATM   48  H             0        6.329   1.557  -0.230                  H
HETATM   49  H             0        6.864   0.338  -1.399                  H
HETATM   50  H             0        2.692  -0.766  -3.521                  H
HETATM   51  H             0        1.256  -0.269  -2.590                  H
HETATM   52  H             0        1.669  -1.984  -2.715                  H
HETATM   53  C             0        1.325   1.953  -0.430                  C
HETATM   54  H             0        1.482   2.256  -1.484                  H
HETATM   55  H             0        2.095   2.249   0.309                  H
HETATM   56  C             0       -1.435   2.495   0.499                  C
HETATM   57  H             0       -2.261   2.462  -0.223                  H
HETATM   58  H             0       -1.657   2.438   1.572                  H
HETATM   59  C             0       -0.306   3.269   0.130                  C
HETATM   60  F             0       -0.321   3.960  -1.000                  F
HETATM   61  F             0        0.370   3.933   1.055                  F
END
```

a2n.pdb

```
TITLE      a2n.pdb
HETATM    1 Ru             0       -0.264   0.867   0.062                 Ru
HETATM    2  C             0       -0.350  -3.333   0.224                  C
HETATM    3  C             0        1.169  -3.180   0.310                  C
HETATM    4  H             0       -0.676  -3.814  -0.719                  H
HETATM    5  H             0        1.708  -3.735  -0.478                  H
HETATM    6  C             0        0.203  -1.053   0.140                  C
HETATM    7  N             0        1.370  -1.737   0.143                  N
HETATM    8  N             0       -0.812  -1.949   0.272                  N
HETATM    9 Cl             0       -0.679   1.061  -2.320                 Cl
HETATM   10 Cl             0       -0.141   0.978   2.489                 Cl
HETATM   11  C             0       -1.852   2.709   0.180                  C
HETATM   12  C             0        2.673  -1.181  -0.006                  C
HETATM   13  C             0        3.462  -0.932   1.133                  C
HETATM   14  C             0        3.152  -0.903  -1.302                  C
HETATM   15  C             0        4.741  -0.395   0.951                  C
HETATM   16  C             0        4.436  -0.364  -1.437                  C
HETATM   17  C             0        5.241  -0.101  -0.323                  C
HETATM   18  H             0        5.359  -0.189   1.835                  H
HETATM   19  H             0        4.815  -0.145  -2.444                  H
HETATM   20  C             0       -2.178  -1.561   0.095                  C
HETATM   21  C             0       -2.983  -1.314   1.230                  C
HETATM   22  C             0       -2.712  -1.470  -1.208                  C
HETATM   23  C             0       -4.291  -0.861   1.029                  C
HETATM   24  C             0       -4.027  -1.017  -1.359                  C
HETATM   25  C             0       -4.821  -0.686  -0.256                  C
HETATM   26  H             0       -4.919  -0.657   1.905                  H
```

```
HETATM   27  H              0       -4.441  -0.925  -2.371                        H
HETATM   28  C              0        2.918  -1.178   2.506                        C
HETATM   29  H              0        3.621  -0.830   3.280                        H
HETATM   30  H              0        1.958  -0.646   2.655                        H
HETATM   31  H              0        2.725  -2.250   2.700                        H
HETATM   32  C              0        2.290  -1.155  -2.499                        C
HETATM   33  H              0        1.878  -2.182  -2.507                        H
HETATM   34  H              0        1.419  -0.469  -2.523                        H
HETATM   35  H              0        2.853  -1.012  -3.435                        H
HETATM   36  C              0        6.599   0.512  -0.491                        C
HETATM   37  H              0        6.536   1.616  -0.542                        H
HETATM   38  H              0        7.269   0.270   0.350                        H
HETATM   39  H              0        7.087   0.182  -1.424                        H
HETATM   40  C              0       -2.475  -1.589   2.609                        C
HETATM   41  H              0       -3.237  -1.345   3.367                        H
HETATM   42  H              0       -2.214  -2.656   2.737                        H
HETATM   43  H              0       -1.570  -0.999   2.837                        H
HETATM   44  C              0       -1.937  -1.922  -2.408                        C
HETATM   45  H              0       -2.142  -2.991  -2.620                        H
HETATM   46  H              0       -2.219  -1.347  -3.303                        H
HETATM   47  H              0       -0.849  -1.801  -2.286                        H
HETATM   48  C              0       -6.210  -0.156  -0.444                        C
HETATM   49  H              0       -6.208   0.946  -0.544                        H
HETATM   50  H              0       -6.683  -0.553  -1.358                        H
HETATM   51  H              0       -6.863  -0.395   0.413                        H
HETATM   52  H              0        1.574  -3.514   1.284                        H
HETATM   53  H              0       -0.774  -3.919   1.058                        H
HETATM   54  C              0       -0.834   3.595   0.114                        C
HETATM   55  C              0        1.422   1.520  -0.107                        C
HETATM   56  H              0        2.195   1.427   0.684                        H
HETATM   57  H              0        1.717   2.104  -1.008                        H
HETATM   58  H              0       -2.265   2.471   1.164                        H
HETATM   59  H              0       -2.394   2.472  -0.739                        H
HETATM   60  F              0       -0.368   4.112  -0.988                        F
HETATM   61  F              0       -0.230   4.111   1.149                        F
END
```

sc3g.pdb

```
TITLE       sc3g.pdb
HETATM    1 Ru              0       -0.262   0.936  -0.279                       Ru
HETATM    2  C              0       -0.244  -2.860   1.519                        C
HETATM    3  C              0        1.276  -2.689   1.492                        C
HETATM    4  H              0       -0.587  -3.747   0.953                        H
HETATM    5  H              0        1.796  -3.495   0.942                        H
HETATM    6  C              0        0.288  -0.824   0.460                        C
HETATM    7  N              0        1.460  -1.407   0.803                        N
HETATM    8  N              0       -0.718  -1.636   0.883                        N
HETATM    9 Cl              0       -0.825   0.126  -2.500                       Cl
HETATM   10 Cl              0       -0.236   1.907   1.918                       Cl
HETATM   11  C              0       -2.202   2.550  -0.778                        C
HETATM   12  C              0        2.753  -0.970   0.396                        C
HETATM   13  C              0        3.539  -0.186   1.260                        C
HETATM   14  C              0        3.214  -1.332  -0.883                        C
HETATM   15  C              0        4.797   0.237   0.815                        C
HETATM   16  C              0        4.479  -0.895  -1.288                        C
HETATM   17  C              0        5.280  -0.106  -0.453                        C
HETATM   18  H              0        5.413   0.855   1.480                        H
HETATM   19  H              0        4.843  -1.171  -2.286                        H
HETATM   20  C              0       -2.084  -1.399   0.532                        C
HETATM   21  C              0       -2.923  -0.654   1.386                        C
HETATM   22  C              0       -2.578  -1.930  -0.682                        C
```

```
HETATM   23  C              0       -4.217  -0.345   0.943                       C
HETATM   24  C              0       -3.877  -1.610  -1.073                       C
HETATM   25  C              0       -4.702  -0.793  -0.286                       C
HETATM   26  H              0       -4.863   0.261   1.593                       H
HETATM   27  H              0       -4.252  -1.997  -2.029                       H
HETATM   28  C              0        3.016   0.218   2.603                       C
HETATM   29  H              0        3.754   0.822   3.156                       H
HETATM   30  H              0        2.085   0.811   2.514                       H
HETATM   31  H              0        2.766  -0.657   3.232                       H
HETATM   32  C              0        2.329  -2.098  -1.818                       C
HETATM   33  H              0        1.899  -3.003  -1.349                       H
HETATM   34  H              0        1.468  -1.482  -2.151                       H
HETATM   35  H              0        2.877  -2.416  -2.720                       H
HETATM   36  C              0        6.615   0.392  -0.921                       C
HETATM   37  H              0        6.528   1.390  -1.391                       H
HETATM   38  H              0        7.331   0.498  -0.088                       H
HETATM   39  H              0        7.064  -0.276  -1.675                       H
HETATM   40  C              0       -2.523  -0.270   2.777                       C
HETATM   41  H              0       -2.723   0.796   2.975                       H
HETATM   42  H              0       -3.113  -0.855   3.509                       H
HETATM   43  H              0       -1.455  -0.436   2.981                       H
HETATM   44  C              0       -1.749  -2.853  -1.521                       C
HETATM   45  H              0       -1.878  -3.906  -1.200                       H
HETATM   46  H              0       -2.043  -2.797  -2.581                       H
HETATM   47  H              0       -0.674  -2.617  -1.474                       H
HETATM   48  C              0       -6.067  -0.405  -0.765                       C
HETATM   49  H              0       -6.015   0.445  -1.472                       H
HETATM   50  H              0       -6.566  -1.229  -1.305                       H
HETATM   51  H              0       -6.723  -0.092   0.064                       H
HETATM   52  H              0        1.722  -2.645   2.503                       H
HETATM   53  H              0       -0.650  -2.945   2.544                       H
HETATM   54  C              0       -1.067   3.230  -1.043                       C
HETATM   55  H              0       -0.615   3.131  -2.033                       H
HETATM   56  C              0        1.377   1.535  -0.735                       C
HETATM   57  H              0        2.210   0.948  -1.177                       H
HETATM   58  H              0       -0.666   3.961  -0.334                       H
HETATM   59  H              0       -2.687   1.849  -1.471                       H
HETATM   60  F              0        1.769   2.806  -0.660                       F
HETATM   61  F              0       -2.907   2.742   0.332                       F
END
```

sc3h.pdb

```
TITLE       sc3h.pdb
HETATM    1 Ru              0        0.221  -0.949  -0.134                      Ru
HETATM    2 Cl              0        0.935  -0.741  -2.470                      Cl
HETATM    3 Cl              0       -0.425  -1.262   2.189                      Cl
HETATM    4  C              0       -0.119   1.031   0.216                       C
HETATM    5  N              0        0.921   1.836   0.508                       N
HETATM    6  C              0        0.506   3.211   0.790                       C
HETATM    7  C              0       -1.024   3.120   0.725                       C
HETATM    8  N              0       -1.262   1.728   0.323                       N
HETATM    9  C              0        2.266   1.393   0.290                       C
HETATM   10  C              0        2.843   1.553  -0.988                       C
HETATM   11  C              0        4.112   1.017  -1.216                       C
HETATM   12  C              0        4.817   0.345  -0.210                       C
HETATM   13  C              0        4.246   0.252   1.063                       C
HETATM   14  C              0        2.979   0.781   1.343                       C
HETATM   15  C              0       -2.559   1.186   0.082                       C
HETATM   16  C              0       -3.402   0.857   1.159                       C
HETATM   17  C              0       -4.644   0.277   0.877                       C
HETATM   18  C              0       -5.058   0.026  -0.434                       C
```

```
HETATM   19  C              0       -4.214   0.398  -1.488                      C
HETATM   20  C              0       -2.967   0.984  -1.252                      C
HETATM   21  C              0        2.136   2.308  -2.071                      C
HETATM   22  C              0        6.150  -0.274  -0.502                      C
HETATM   23  C              0        2.424   0.728   2.731                      C
HETATM   24  C              0       -3.002   1.130   2.577                      C
HETATM   25  C              0       -6.369  -0.648  -0.708                      C
HETATM   26  C              0       -2.082   1.388  -2.391                      C
HETATM   27  H              0        0.927   3.900   0.033                      H
HETATM   28  H              0        0.878   3.537   1.778                      H
HETATM   29  H              0       -1.469   3.810  -0.015                      H
HETATM   30  H              0       -1.506   3.322   1.699                      H
HETATM   31  H              0        4.554   1.114  -2.216                      H
HETATM   32  H              0        4.801  -0.239   1.872                      H
HETATM   33  H              0       -5.299   0.002   1.714                      H
HETATM   34  H              0       -4.536   0.237  -2.525                      H
HETATM   35  H              0        1.043   2.168  -2.035                      H
HETATM   36  H              0        2.468   1.978  -3.067                      H
HETATM   37  H              0        2.340   3.395  -1.997                      H
HETATM   38  H              0        6.034  -1.247  -1.016                      H
HETATM   39  H              0        6.728  -0.464   0.418                      H
HETATM   40  H              0        6.763   0.358  -1.168                      H
HETATM   41  H              0        3.167   0.322   3.437                      H
HETATM   42  H              0        1.517   0.097   2.798                      H
HETATM   43  H              0        2.137   1.733   3.092                      H
HETATM   44  H              0       -1.913   1.046   2.721                      H
HETATM   45  H              0       -3.474   0.411   3.266                      H
HETATM   46  H              0       -3.320   2.141   2.900                      H
HETATM   47  H              0       -6.247  -1.745  -0.785                      H
HETATM   48  H              0       -7.103  -0.467   0.095                      H
HETATM   49  H              0       -6.815  -0.312  -1.661                      H
HETATM   50  H              0       -2.627   1.352  -3.349                      H
HETATM   51  H              0       -1.197   0.729  -2.488                      H
HETATM   52  H              0       -1.690   2.415  -2.265                      H
HETATM   53  C              0       -1.254  -1.909  -0.690                      C
HETATM   54  F              0       -2.206  -2.454   0.043                      F
HETATM   55  H              0       -1.504  -2.046  -1.765                      H
HETATM   56  C              0        1.645  -2.548  -0.013                      C
HETATM   57  C              0        0.500  -3.193  -0.530                      C
HETATM   58  F              0        2.076  -2.845   1.221                      F
HETATM   59  H              0        2.466  -2.214  -0.665                      H
HETATM   60  H              0        0.482  -3.385  -1.610                      H
HETATM   61  H              0       -0.022  -3.895   0.133                      H
END
```

sc3NA.pdb

```
TITLE      sc3NA.pdb
HETATM    1 Ru              0        0.042  -0.963  -0.133                     Ru
HETATM    2  C              0        0.734   3.199   0.607                      C
HETATM    3  C              0       -0.777   3.233   0.360                      C
HETATM    4  H              0        1.299   3.865  -0.068                      H
HETATM    5  H              0       -1.056   3.811  -0.542                      H
HETATM    6  C              0       -0.013   1.046   0.131                      C
HETATM    7  N              0       -1.111   1.816   0.169                      N
HETATM    8  N              0        1.079   1.798   0.343                      N
HETATM    9 Cl              0        1.029  -0.787  -2.370                     Cl
HETATM   10 Cl              0       -0.945  -1.171   2.066                     Cl
HETATM   11  C              0        1.365  -2.368   0.169                      C
HETATM   12  C              0       -2.441   1.319   0.003                      C
HETATM   13  C              0       -3.277   1.192   1.128                      C
HETATM   14  C              0       -2.885   0.961  -1.284                      C
```

```
HETATM   15  C              0       -4.550   0.643   0.947                       C
HETATM   16  C              0       -4.164   0.408  -1.416                       C
HETATM   17  C              0       -5.001   0.228  -0.310                       C
HETATM   18  H              0       -5.200   0.519   1.823                       H
HETATM   19  H              0       -4.517   0.122  -2.416                       H
HETATM   20  C              0        2.416   1.297   0.254                       C
HETATM   21  C              0        2.979   0.616   1.352                       C
HETATM   22  C              0        3.134   1.484  -0.941                       C
HETATM   23  C              0        4.263   0.077   1.208                       C
HETATM   24  C              0        4.416   0.936  -1.037                       C
HETATM   25  C              0        4.989   0.218   0.020                       C
HETATM   26  H              0        4.708  -0.461   2.054                       H
HETATM   27  H              0        4.975   1.059  -1.973                       H
HETATM   28  C              0       -2.834   1.641   2.486                       C
HETATM   29  H              0       -3.357   1.080   3.278                       H
HETATM   30  H              0       -1.753   1.481   2.639                       H
HETATM   31  H              0       -3.055   2.714   2.653                       H
HETATM   32  C              0       -2.026   1.189  -2.491                       C
HETATM   33  H              0       -1.601   2.211  -2.508                       H
HETATM   34  H              0       -1.161   0.501  -2.545                       H
HETATM   35  H              0       -2.606   1.057  -3.419                       H
HETATM   36  C              0       -6.346  -0.414  -0.465                       C
HETATM   37  H              0       -6.289  -1.504  -0.285                       H
HETATM   38  H              0       -7.081  -0.014   0.254                       H
HETATM   39  H              0       -6.754  -0.281  -1.482                       H
HETATM   40  C              0        2.231   0.475   2.642                       C
HETATM   41  H              0        2.871   0.033   3.422                       H
HETATM   42  H              0        1.871   1.451   3.017                       H
HETATM   43  H              0        1.333  -0.166   2.548                       H
HETATM   44  C              0        2.556   2.255  -2.088                       C
HETATM   45  H              0        2.761   3.341  -1.995                       H
HETATM   46  H              0        2.992   1.925  -3.044                       H
HETATM   47  H              0        1.464   2.121  -2.170                       H
HETATM   48  C              0        6.344  -0.405  -0.126                       C
HETATM   49  H              0        6.273  -1.411  -0.583                       H
HETATM   50  H              0        7.006   0.190  -0.778                       H
HETATM   51  H              0        6.846  -0.534   0.848                       H
HETATM   52  H              0       -1.344   3.649   1.209                       H
HETATM   53  H              0        1.008   3.467   1.645                       H
HETATM   54  C              0        0.170  -3.153  -0.474                       C
HETATM   55  H              0        0.560  -3.578  -1.415                       H
HETATM   56  H              0       -0.143  -3.912   0.262                       H
HETATM   57  C              0       -1.123  -2.353  -0.867                       C
HETATM   58  H              0       -1.313  -2.305  -1.954                       H
HETATM   59  H              0        2.279  -2.335  -0.447                       H
HETATM   60  F              0        1.677  -2.782   1.415                       F
HETATM   61  F              0       -2.234  -2.778  -0.240                       F
END
```

sc3i.pdb

```
TITLE      sc3i.pdb
HETATM    1 Ru              0        0.221  -0.949  -0.134                      Ru
HETATM    2 Cl              0        0.935  -0.741  -2.470                      Cl
HETATM    3 Cl              0       -0.425  -1.262   2.189                      Cl
HETATM    4  C              0       -0.119   1.031   0.216                       C
HETATM    5  N              0        0.921   1.836   0.508                       N
HETATM    6  C              0        0.506   3.211   0.790                       C
HETATM    7  C              0       -1.024   3.120   0.725                       C
HETATM    8  N              0       -1.262   1.728   0.323                       N
HETATM    9  C              0        2.266   1.393   0.290                       C
```

```
HETATM   10  C            0       2.843   1.553  -0.988                        C
HETATM   11  C            0       4.112   1.017  -1.216                        C
HETATM   12  C            0       4.817   0.345  -0.210                        C
HETATM   13  C            0       4.246   0.252   1.063                        C
HETATM   14  C            0       2.979   0.781   1.343                        C
HETATM   15  C            0      -2.559   1.186   0.082                        C
HETATM   16  C            0      -3.402   0.857   1.159                        C
HETATM   17  C            0      -4.644   0.277   0.877                        C
HETATM   18  C            0      -5.058   0.026  -0.434                        C
HETATM   19  C            0      -4.214   0.398  -1.488                        C
HETATM   20  C            0      -2.967   0.984  -1.252                        C
HETATM   21  C            0       2.136   2.308  -2.071                        C
HETATM   22  C            0       6.150  -0.274  -0.502                        C
HETATM   23  C            0       2.424   0.728   2.731                        C
HETATM   24  C            0      -3.002   1.130   2.577                        C
HETATM   25  C            0      -6.369  -0.648  -0.708                        C
HETATM   26  C            0      -2.082   1.388  -2.391                        C
HETATM   27  H            0       0.927   3.900   0.033                        H
HETATM   28  H            0       0.878   3.537   1.778                        H
HETATM   29  H            0      -1.469   3.810  -0.015                        H
HETATM   30  H            0      -1.506   3.322   1.699                        H
HETATM   31  H            0       4.554   1.114  -2.216                        H
HETATM   32  H            0       4.801  -0.239   1.872                        H
HETATM   33  H            0      -5.299   0.002   1.714                        H
HETATM   34  H            0      -4.536   0.237  -2.525                        H
HETATM   35  H            0       1.043   2.168  -2.035                        H
HETATM   36  H            0       2.468   1.978  -3.067                        H
HETATM   37  H            0       2.340   3.395  -1.997                        H
HETATM   38  H            0       6.034  -1.247  -1.016                        H
HETATM   39  H            0       6.728  -0.464   0.418                        H
HETATM   40  H            0       6.763   0.358  -1.168                        H
HETATM   41  H            0       3.167   0.322   3.437                        H
HETATM   42  H            0       1.517   0.097   2.798                        H
HETATM   43  H            0       2.137   1.733   3.092                        H
HETATM   44  H            0      -1.913   1.046   2.721                        H
HETATM   45  H            0      -3.474   0.411   3.266                        H
HETATM   46  H            0      -3.320   2.141   2.900                        H
HETATM   47  H            0      -6.247  -1.745  -0.785                        H
HETATM   48  H            0      -7.103  -0.467   0.095                        H
HETATM   49  H            0      -6.815  -0.312  -1.661                        H
HETATM   50  H            0      -2.627   1.352  -3.349                        H
HETATM   51  H            0      -1.197   0.729  -2.488                        H
HETATM   52  H            0      -1.690   2.415  -2.265                        H
HETATM   53  C            0      -1.254  -1.909  -0.690                        C
HETATM   54  F            0      -2.206  -2.454   0.043                        F
HETATM   55  H            0      -1.504  -2.046  -1.765                        H
HETATM   56  C            0       1.645  -2.548  -0.013                        C
HETATM   57  C            0       0.500  -3.193  -0.530                        C
HETATM   58  F            0       2.076  -2.845   1.221                        F
HETATM   59  H            0       2.466  -2.214  -0.665                        H
HETATM   60  H            0       0.482  -3.385  -1.610                        H
HETATM   61  H            0      -0.022  -3.895   0.133                        H
END
```

sc3j.pdb

```
TITLE      sc3j.pdb
HETATM    1 Ru            0      -0.262   0.936  -0.279                       Ru
HETATM    2  C            0      -0.244  -2.860   1.519                        C
HETATM    3  C            0       1.276  -2.689   1.492                        C
HETATM    4  H            0      -0.587  -3.747   0.953                        H
```

```
HETATM    5  H             0       1.796  -3.495   0.942                        H
HETATM    6  C             0       0.288  -0.824   0.460                        C
HETATM    7  N             0       1.460  -1.407   0.803                        N
HETATM    8  N             0      -0.718  -1.636   0.883                        N
HETATM    9 Cl             0      -0.825   0.126  -2.500                       Cl
HETATM   10 Cl             0      -0.236   1.907   1.918                       Cl
HETATM   11  C             0      -2.202   2.550  -0.778                        C
HETATM   12  C             0       2.753  -0.970   0.396                        C
HETATM   13  C             0       3.539  -0.186   1.260                        C
HETATM   14  C             0       3.214  -1.332  -0.883                        C
HETATM   15  C             0       4.797   0.237   0.815                        C
HETATM   16  C             0       4.479  -0.895  -1.288                        C
HETATM   17  C             0       5.280  -0.106  -0.453                        C
HETATM   18  H             0       5.413   0.855   1.480                        H
HETATM   19  H             0       4.843  -1.171  -2.286                        H
HETATM   20  C             0      -2.084  -1.399   0.532                        C
HETATM   21  C             0      -2.923  -0.654   1.386                        C
HETATM   22  C             0      -2.578  -1.930  -0.682                        C
HETATM   23  C             0      -4.217  -0.345   0.943                        C
HETATM   24  C             0      -3.877  -1.610  -1.073                        C
HETATM   25  C             0      -4.702  -0.793  -0.286                        C
HETATM   26  H             0      -4.863   0.261   1.593                        H
HETATM   27  H             0      -4.252  -1.997  -2.029                        H
HETATM   28  C             0       3.016   0.218   2.603                        C
HETATM   29  H             0       3.754   0.822   3.156                        H
HETATM   30  H             0       2.085   0.811   2.514                        H
HETATM   31  H             0       2.766  -0.657   3.232                        H
HETATM   32  C             0       2.329  -2.098  -1.818                        C
HETATM   33  H             0       1.899  -3.003  -1.349                        H
HETATM   34  H             0       1.468  -1.482  -2.151                        H
HETATM   35  H             0       2.877  -2.416  -2.720                        H
HETATM   36  C             0       6.615   0.392  -0.921                        C
HETATM   37  H             0       6.528   1.390  -1.391                        H
HETATM   38  H             0       7.331   0.498  -0.088                        H
HETATM   39  H             0       7.064  -0.276  -1.675                        H
HETATM   40  C             0      -2.523  -0.270   2.777                        C
HETATM   41  H             0      -2.723   0.796   2.975                        H
HETATM   42  H             0      -3.113  -0.855   3.509                        H
HETATM   43  H             0      -1.455  -0.436   2.981                        H
HETATM   44  C             0      -1.749  -2.853  -1.521                        C
HETATM   45  H             0      -1.878  -3.906  -1.200                        H
HETATM   46  H             0      -2.043  -2.797  -2.581                        H
HETATM   47  H             0      -0.674  -2.617  -1.474                        H
HETATM   48  C             0      -6.067  -0.405  -0.765                        C
HETATM   49  H             0      -6.015   0.445  -1.472                        H
HETATM   50  H             0      -6.566  -1.229  -1.305                        H
HETATM   51  H             0      -6.723  -0.092   0.064                        H
HETATM   52  H             0       1.722  -2.645   2.503                        H
HETATM   53  H             0      -0.650  -2.945   2.544                        H
HETATM   54  C             0      -1.067   3.230  -1.043                        C
HETATM   55  H             0      -0.615   3.131  -2.033                        H
HETATM   56  C             0       1.377   1.535  -0.735                        C
HETATM   57  H             0       2.210   0.948  -1.177                        H
HETATM   58  H             0      -0.666   3.961  -0.334                        H
HETATM   59  H             0      -2.687   1.849  -1.471                        H
HETATM   60  F             0       1.769   2.806  -0.660                        F
HETATM   61  F             0      -2.907   2.742   0.332                        F
END
```

st3g.pdb

```
TITLE      st3g.pdb
```

```
HETATM    1 Ru           0        0.238  -0.976   0.076                        Ru
HETATM    2  C           0        0.326   3.236   0.200                         C
HETATM    3  C           0       -1.196   3.094   0.269                         C
HETATM    4  H           0        0.666   3.742  -0.724                         H
HETATM    5  H           0       -1.720   3.622  -0.548                         H
HETATM    6  C           0       -0.242   0.956   0.123                         C
HETATM    7  N           0       -1.403   1.648   0.149                         N
HETATM    8  N           0        0.779   1.848   0.213                         N
HETATM    9 Cl           0        0.756  -1.132  -2.297                        Cl
HETATM   10 Cl           0        0.054  -1.049   2.495                        Cl
HETATM   11  C           0        2.089  -2.697   0.130                         C
HETATM   12  C           0       -2.706   1.085   0.029                         C
HETATM   13  C           0       -3.482   0.869   1.183                         C
HETATM   14  C           0       -3.184   0.737  -1.250                         C
HETATM   15  C           0       -4.748   0.289   1.033                         C
HETATM   16  C           0       -4.453   0.157  -1.353                         C
HETATM   17  C           0       -5.245  -0.078  -0.223                         C
HETATM   18  H           0       -5.356   0.108   1.929                         H
HETATM   19  H           0       -4.829  -0.121  -2.346                         H
HETATM   20  C           0        2.143   1.454   0.030                         C
HETATM   21  C           0        2.950   1.184   1.157                         C
HETATM   22  C           0        2.672   1.383  -1.276                         C
HETATM   23  C           0        4.253   0.722   0.944                         C
HETATM   24  C           0        3.981   0.919  -1.439                         C
HETATM   25  C           0        4.774   0.557  -0.345                         C
HETATM   26  H           0        4.879   0.490   1.815                         H
HETATM   27  H           0        4.389   0.836  -2.455                         H
HETATM   28  C           0       -2.943   1.192   2.541                         C
HETATM   29  H           0       -3.641   0.874   3.332                         H
HETATM   30  H           0       -1.975   0.683   2.719                         H
HETATM   31  H           0       -2.768   2.276   2.681                         H
HETATM   32  C           0       -2.332   0.946  -2.463                         C
HETATM   33  H           0       -1.911   1.968  -2.505                         H
HETATM   34  H           0       -1.466   0.253  -2.483                         H
HETATM   35  H           0       -2.908   0.782  -3.389                         H
HETATM   36  C           0       -6.585  -0.737  -0.355                         C
HETATM   37  H           0       -6.488  -1.839  -0.385                         H
HETATM   38  H           0       -7.248  -0.499   0.494                         H
HETATM   39  H           0       -7.100  -0.442  -1.285                         H
HETATM   40  C           0        2.457   1.450   2.544                         C
HETATM   41  H           0        3.206   1.148   3.294                         H
HETATM   42  H           0        2.257   2.528   2.696                         H
HETATM   43  H           0        1.523   0.907   2.767                         H
HETATM   44  C           0        1.897   1.865  -2.464                         C
HETATM   45  H           0        2.102   2.939  -2.652                         H
HETATM   46  H           0        2.180   1.311  -3.374                         H
HETATM   47  H           0        0.809   1.741  -2.345                         H
HETATM   48  C           0        6.150   0.000  -0.546                         C
HETATM   49  H           0        6.126  -1.105  -0.605                         H
HETATM   50  H           0        6.608   0.357  -1.483                         H
HETATM   51  H           0        6.826   0.256   0.288                         H
HETATM   52  H           0       -1.615   3.466   1.223                         H
HETATM   53  H           0        0.747   3.793   1.056                         H
HETATM   54  C           0        0.931  -3.390   0.210                         C
HETATM   55  H           0        0.432  -3.681  -0.718                         H
HETATM   56  C           0       -1.432  -1.639  -0.119                         C
HETATM   57  H           0       -2.291  -1.442   0.557                         H
HETATM   58  H           0        0.569  -3.776   1.169                         H
HETATM   59  H           0        2.560  -2.361  -0.803                         H
HETATM   60  F           0        2.832  -2.441   1.204                         F
HETATM   61  F           0       -1.820  -2.512  -1.042                         F
END
```

st3h.pdb

```
TITLE      st3h.pdb
HETATM    1 Ru            0        0.221  -0.949  -0.134                     Ru
HETATM    2 Cl            0        0.935  -0.741  -2.470                     Cl
HETATM    3 Cl            0       -0.425  -1.262   2.189                     Cl
HETATM    4  C            0       -0.119   1.031   0.216                      C
HETATM    5  N            0        0.921   1.836   0.508                      N
HETATM    6  C            0        0.506   3.211   0.790                      C
HETATM    7  C            0       -1.024   3.120   0.725                      C
HETATM    8  N            0       -1.262   1.728   0.323                      N
HETATM    9  C            0        2.266   1.393   0.290                      C
HETATM   10  C            0        2.843   1.553  -0.988                      C
HETATM   11  C            0        4.112   1.017  -1.216                      C
HETATM   12  C            0        4.817   0.345  -0.210                      C
HETATM   13  C            0        4.246   0.252   1.063                      C
HETATM   14  C            0        2.979   0.781   1.343                      C
HETATM   15  C            0       -2.559   1.186   0.082                      C
HETATM   16  C            0       -3.402   0.857   1.159                      C
HETATM   17  C            0       -4.644   0.277   0.877                      C
HETATM   18  C            0       -5.058   0.026  -0.434                      C
HETATM   19  C            0       -4.214   0.398  -1.488                      C
HETATM   20  C            0       -2.967   0.984  -1.252                      C
HETATM   21  C            0        2.136   2.308  -2.071                      C
HETATM   22  C            0        6.150  -0.274  -0.502                      C
HETATM   23  C            0        2.424   0.728   2.731                      C
HETATM   24  C            0       -3.002   1.130   2.577                      C
HETATM   25  C            0       -6.369  -0.648  -0.708                      C
HETATM   26  C            0       -2.082   1.388  -2.391                      C
HETATM   27  H            0        0.927   3.900   0.033                      H
HETATM   28  H            0        0.878   3.537   1.778                      H
HETATM   29  H            0       -1.469   3.810  -0.015                      H
HETATM   30  H            0       -1.506   3.322   1.699                      H
HETATM   31  H            0        4.554   1.114  -2.216                      H
HETATM   32  H            0        4.801  -0.239   1.872                      H
HETATM   33  H            0       -5.299   0.002   1.714                      H
HETATM   34  H            0       -4.536   0.237  -2.525                      H
HETATM   35  H            0        1.043   2.168  -2.035                      H
HETATM   36  H            0        2.468   1.978  -3.067                      H
HETATM   37  H            0        2.340   3.395  -1.997                      H
HETATM   38  H            0        6.034  -1.247  -1.016                      H
HETATM   39  H            0        6.728  -0.464   0.418                      H
HETATM   40  H            0        6.763   0.358  -1.168                      H
HETATM   41  H            0        3.167   0.322   3.437                      H
HETATM   42  H            0        1.517   0.097   2.798                      H
HETATM   43  H            0        2.137   1.733   3.092                      H
HETATM   44  H            0       -1.913   1.046   2.721                      H
HETATM   45  H            0       -3.474   0.411   3.266                      H
HETATM   46  H            0       -3.320   2.141   2.900                      H
HETATM   47  H            0       -6.247  -1.745  -0.785                      H
HETATM   48  H            0       -7.103  -0.467   0.095                      H
HETATM   49  H            0       -6.815  -0.312  -1.661                      H
HETATM   50  H            0       -2.627   1.352  -3.349                      H
HETATM   51  H            0       -1.197   0.729  -2.488                      H
HETATM   52  H            0       -1.690   2.415  -2.265                      H
HETATM   53  C            0       -1.254  -1.909  -0.690                      C
HETATM   54  F            0       -2.206  -2.454   0.043                      F
HETATM   55  H            0       -1.504  -2.046  -1.765                      H
HETATM   56  C            0        1.645  -2.548  -0.013                      C
HETATM   57  C            0        0.500  -3.193  -0.530                      C
HETATM   58  F            0        2.076  -2.845   1.221                      F
```

```
HETATM   59  H              0       2.466  -2.214  -0.665                      H
HETATM   60  H              0       0.482  -3.385  -1.610                      H
HETATM   61  H              0      -0.022  -3.895   0.133                      H
END
```

st3NA.pdb

```
TITLE       st3NA
HETATM    1 Ru              0       0.004  -0.965   0.007                     Ru
HETATM    2  C              0       0.755   3.262   0.085                      C
HETATM    3  C              0      -0.772   3.258  -0.044                      C
HETATM    4  H              0       1.258   3.798  -0.739                      H
HETATM    5  H              0      -1.128   3.707  -0.990                      H
HETATM    6  C              0      -0.003   1.061   0.010                      C
HETATM    7  N              0      -1.103   1.829  -0.013                      N
HETATM    8  N              0       1.093   1.835   0.041                      N
HETATM    9 Cl              0       0.935  -1.001  -2.242                     Cl
HETATM   10 Cl              0      -0.921  -0.984   2.259                     Cl
HETATM   11  C              0       1.304  -2.377   0.370                      C
HETATM   12  C              0      -2.433   1.310  -0.070                      C
HETATM   13  C              0      -3.225   1.308   1.096                      C
HETATM   14  C              0      -2.921   0.798  -1.287                      C
HETATM   15  C              0      -4.494   0.729   1.033                      C
HETATM   16  C              0      -4.199   0.226  -1.302                      C
HETATM   17  C              0      -4.990   0.170  -0.151                      C
HETATM   18  H              0      -5.108   0.704   1.943                      H
HETATM   19  H              0      -4.584  -0.181  -2.246                      H
HETATM   20  C              0       2.426   1.319   0.071                      C
HETATM   21  C              0       2.939   0.805   1.277                      C
HETATM   22  C              0       3.193   1.321  -1.111                      C
HETATM   23  C              0       4.218   0.235   1.264                      C
HETATM   24  C              0       4.464   0.744  -1.076                      C
HETATM   25  C              0       4.986   0.183   0.097                      C
HETATM   26  H              0       4.622  -0.175   2.198                      H
HETATM   27  H              0       5.059   0.721  -1.998                      H
HETATM   28  C              0      -2.734   1.920   2.372                      C
HETATM   29  H              0      -3.209   1.446   3.246                      H
HETATM   30  H              0      -1.644   1.803   2.493                      H
HETATM   31  H              0      -2.972   3.002   2.422                      H
HETATM   32  C              0      -2.108   0.857  -2.544                      C
HETATM   33  H              0      -1.524   1.791  -2.624                      H
HETATM   34  H              0      -1.369   0.035  -2.608                      H
HETATM   35  H              0      -2.755   0.784  -3.434                      H
HETATM   36  C              0      -6.337  -0.487  -0.174                      C
HETATM   37  H              0      -6.288  -1.512   0.241                      H
HETATM   38  H              0      -7.075   0.061   0.437                      H
HETATM   39  H              0      -6.737  -0.575  -1.197                      H
HETATM   40  C              0       2.151   0.860   2.550                      C
HETATM   41  H              0       2.811   0.740   3.425                      H
HETATM   42  H              0       1.606   1.814   2.665                      H
HETATM   43  H              0       1.382   0.065   2.607                      H
HETATM   44  C              0       2.670   1.931  -2.375                      C
HETATM   45  H              0       2.879   3.019  -2.420                      H
HETATM   46  H              0       3.146   1.476  -3.260                      H
HETATM   47  H              0       1.582   1.785  -2.484                      H
HETATM   48  C              0       6.333  -0.472   0.090                      C
HETATM   49  H              0       6.278  -1.494  -0.330                      H
HETATM   50  H              0       7.059   0.082  -0.531                      H
HETATM   51  H              0       6.753  -0.565   1.106                      H
HETATM   52  H              0      -1.278   3.785   0.785                      H
HETATM   53  H              0       1.108   3.704   1.035                      H
HETATM   54  C              0       0.008  -3.184   0.015                      C
```

```
HETATM   55  H             0         0.230  -3.790  -0.879                         H
HETATM   56  H             0        -0.212  -3.786   0.913                         H
HETATM   57  C             0        -1.291  -2.383  -0.342                         C
HETATM   58  H             0        -2.117  -2.497   0.379                         H
HETATM   59  H             0         2.132  -2.493  -0.349                         H
HETATM   60  F             0         1.754  -2.606   1.622                         F
HETATM   61  F             0        -1.743  -2.622  -1.592                         F
END
```

st3i.pdb

```
TITLE       st3i.pdb
HETATM    1 Ru             0         0.221  -0.949  -0.134                        Ru
HETATM    2 Cl             0         0.935  -0.741  -2.470                        Cl
HETATM    3 Cl             0        -0.425  -1.262   2.189                        Cl
HETATM    4  C             0        -0.119   1.031   0.216                         C
HETATM    5  N             0         0.921   1.836   0.508                         N
HETATM    6  C             0         0.506   3.211   0.790                         C
HETATM    7  C             0        -1.024   3.120   0.725                         C
HETATM    8  N             0        -1.262   1.728   0.323                         N
HETATM    9  C             0         2.266   1.393   0.290                         C
HETATM   10  C             0         2.843   1.553  -0.988                         C
HETATM   11  C             0         4.112   1.017  -1.216                         C
HETATM   12  C             0         4.817   0.345  -0.210                         C
HETATM   13  C             0         4.246   0.252   1.063                         C
HETATM   14  C             0         2.979   0.781   1.343                         C
HETATM   15  C             0        -2.559   1.186   0.082                         C
HETATM   16  C             0        -3.402   0.857   1.159                         C
HETATM   17  C             0        -4.644   0.277   0.877                         C
HETATM   18  C             0        -5.058   0.026  -0.434                         C
HETATM   19  C             0        -4.214   0.398  -1.488                         C
HETATM   20  C             0        -2.967   0.984  -1.252                         C
HETATM   21  C             0         2.136   2.308  -2.071                         C
HETATM   22  C             0         6.150  -0.274  -0.502                         C
HETATM   23  C             0         2.424   0.728   2.731                         C
HETATM   24  C             0        -3.002   1.130   2.577                         C
HETATM   25  C             0        -6.369  -0.648  -0.708                         C
HETATM   26  C             0        -2.082   1.388  -2.391                         C
HETATM   27  H             0         0.927   3.900   0.033                         H
HETATM   28  H             0         0.878   3.537   1.778                         H
HETATM   29  H             0        -1.469   3.810  -0.015                         H
HETATM   30  H             0        -1.506   3.322   1.699                         H
HETATM   31  H             0         4.554   1.114  -2.216                         H
HETATM   32  H             0         4.801  -0.239   1.872                         H
HETATM   33  H             0        -5.299   0.002   1.714                         H
HETATM   34  H             0        -4.536   0.237  -2.525                         H
HETATM   35  H             0         1.043   2.168  -2.035                         H
HETATM   36  H             0         2.468   1.978  -3.067                         H
HETATM   37  H             0         2.340   3.395  -1.997                         H
HETATM   38  H             0         6.034  -1.247  -1.016                         H
HETATM   39  H             0         6.728  -0.464   0.418                         H
HETATM   40  H             0         6.763   0.358  -1.168                         H
HETATM   41  H             0         3.167   0.322   3.437                         H
HETATM   42  H             0         1.517   0.097   2.798                         H
HETATM   43  H             0         2.137   1.733   3.092                         H
HETATM   44  H             0        -1.913   1.046   2.721                         H
HETATM   45  H             0        -3.474   0.411   3.266                         H
HETATM   46  H             0        -3.320   2.141   2.900                         H
HETATM   47  H             0        -6.247  -1.745  -0.785                         H
HETATM   48  H             0        -7.103  -0.467   0.095                         H
HETATM   49  H             0        -6.815  -0.312  -1.661                         H
HETATM   50  H             0        -2.627   1.352  -3.349                         H
```

```
HETATM   51  H              0       -1.197   0.729  -2.488                       H
HETATM   52  H              0       -1.690   2.415  -2.265                       H
HETATM   53  C              0       -1.254  -1.909  -0.690                       C
HETATM   54  F              0       -2.206  -2.454   0.043                       F
HETATM   55  H              0       -1.504  -2.046  -1.765                       H
HETATM   56  C              0        1.645  -2.548  -0.013                       C
HETATM   57  C              0        0.500  -3.193  -0.530                       C
HETATM   58  F              0        2.076  -2.845   1.221                       F
HETATM   59  H              0        2.466  -2.214  -0.665                       H
HETATM   60  H              0        0.482  -3.385  -1.610                       H
HETATM   61  H              0       -0.022  -3.895   0.133                       H
END
```

st3j.pdb

```
TITLE       st3j.pdb
HETATM    1 Ru              0        0.238  -0.976   0.076                      Ru
HETATM    2  C              0        0.326   3.236   0.200                       C
HETATM    3  C              0       -1.196   3.094   0.269                       C
HETATM    4  H              0        0.666   3.742  -0.724                       H
HETATM    5  H              0       -1.720   3.622  -0.548                       H
HETATM    6  C              0       -0.242   0.956   0.123                       C
HETATM    7  N              0       -1.403   1.648   0.149                       N
HETATM    8  N              0        0.779   1.848   0.213                       N
HETATM    9 Cl              0        0.756  -1.132  -2.297                      Cl
HETATM   10 Cl              0        0.054  -1.049   2.495                      Cl
HETATM   11  C              0        2.089  -2.697   0.130                       C
HETATM   12  C              0       -2.706   1.085   0.029                       C
HETATM   13  C              0       -3.482   0.869   1.183                       C
HETATM   14  C              0       -3.184   0.737  -1.250                       C
HETATM   15  C              0       -4.748   0.289   1.033                       C
HETATM   16  C              0       -4.453   0.157  -1.353                       C
HETATM   17  C              0       -5.245  -0.078  -0.223                       C
HETATM   18  H              0       -5.356   0.108   1.929                       H
HETATM   19  H              0       -4.829  -0.121  -2.346                       H
HETATM   20  C              0        2.143   1.454   0.030                       C
HETATM   21  C              0        2.950   1.184   1.157                       C
HETATM   22  C              0        2.672   1.383  -1.276                       C
HETATM   23  C              0        4.253   0.722   0.944                       C
HETATM   24  C              0        3.981   0.919  -1.439                       C
HETATM   25  C              0        4.774   0.557  -0.345                       C
HETATM   26  H              0        4.879   0.490   1.815                       H
HETATM   27  H              0        4.389   0.836  -2.455                       H
HETATM   28  C              0       -2.943   1.192   2.541                       C
HETATM   29  H              0       -3.641   0.874   3.332                       H
HETATM   30  H              0       -1.975   0.683   2.719                       H
HETATM   31  H              0       -2.768   2.276   2.681                       H
HETATM   32  C              0       -2.332   0.946  -2.463                       C
HETATM   33  H              0       -1.911   1.968  -2.505                       H
HETATM   34  H              0       -1.466   0.253  -2.483                       H
HETATM   35  H              0       -2.908   0.782  -3.389                       H
HETATM   36  C              0       -6.585  -0.737  -0.355                       C
HETATM   37  H              0       -6.488  -1.839  -0.385                       H
HETATM   38  H              0       -7.248  -0.499   0.494                       H
HETATM   39  H              0       -7.100  -0.442  -1.285                       H
HETATM   40  C              0        2.457   1.450   2.544                       C
HETATM   41  H              0        3.206   1.148   3.294                       H
HETATM   42  H              0        2.257   2.528   2.696                       H
HETATM   43  H              0        1.523   0.907   2.767                       H
HETATM   44  C              0        1.897   1.865  -2.464                       C
HETATM   45  H              0        2.102   2.939  -2.652                       H
HETATM   46  H              0        2.180   1.311  -3.374                       H
```

```
HETATM   47  H             0        0.809   1.741  -2.345                       H
HETATM   48  C             0        6.150   0.000  -0.546                       C
HETATM   49  H             0        6.126  -1.105  -0.605                       H
HETATM   50  H             0        6.608   0.357  -1.483                       H
HETATM   51  H             0        6.826   0.256   0.288                       H
HETATM   52  H             0       -1.615   3.466   1.223                       H
HETATM   53  H             0        0.747   3.793   1.056                       H
HETATM   54  C             0        0.931  -3.390   0.210                       C
HETATM   55  H             0        0.432  -3.681  -0.718                       H
HETATM   56  C             0       -1.432  -1.639  -0.119                       C
HETATM   57  H             0       -2.291  -1.442   0.557                       H
HETATM   58  H             0        0.569  -3.776   1.169                       H
HETATM   59  H             0        2.560  -2.361  -0.803                       H
HETATM   60  F             0        2.832  -2.441   1.204                       F
HETATM   61  F             0       -1.820  -2.512  -1.042                       F
END
```

ac3g.pdb

```
TITLE      ac3g.pdb
HETATM    1 Ru             0       -0.286   0.907   0.257                      Ru
HETATM    2  C             0       -0.525  -3.285  -0.211                       C
HETATM    3  C             0        0.999  -3.198  -0.113                       C
HETATM    4  H             0       -0.869  -3.633  -1.204                       H
HETATM    5  H             0        1.518  -3.650  -0.976                       H
HETATM    6  C             0        0.108  -1.034   0.020                       C
HETATM    7  N             0        1.248  -1.755  -0.071                       N
HETATM    8  N             0       -0.938  -1.902   0.011                       N
HETATM    9 Cl             0       -0.866   1.359  -2.048                      Cl
HETATM   10 Cl             0        0.017   0.694   2.677                      Cl
HETATM   11  C             0       -1.837   2.665   0.680                       C
HETATM   12  C             0        2.565  -1.218  -0.137                       C
HETATM   13  C             0        3.370  -1.187   1.018                       C
HETATM   14  C             0        3.034  -0.720  -1.369                       C
HETATM   15  C             0        4.649  -0.626   0.921                       C
HETATM   16  C             0        4.318  -0.166  -1.420                       C
HETATM   17  C             0        5.134  -0.104  -0.285                       C
HETATM   18  H             0        5.279  -0.589   1.819                       H
HETATM   19  H             0        4.686   0.228  -2.376                       H
HETATM   20  C             0       -2.291  -1.444  -0.080                       C
HETATM   21  C             0       -3.043  -1.264   1.101                       C
HETATM   22  C             0       -2.859  -1.196  -1.347                       C
HETATM   23  C             0       -4.324  -0.712   0.994                       C
HETATM   24  C             0       -4.144  -0.649  -1.405                       C
HETATM   25  C             0       -4.878  -0.374  -0.247                       C
HETATM   26  H             0       -4.911  -0.558   1.909                       H
HETATM   27  H             0       -4.578  -0.426  -2.388                       H
HETATM   28  C             0        2.856  -1.700   2.327                       C
HETATM   29  H             0        3.541  -1.442   3.152                       H
HETATM   30  H             0        1.865  -1.270   2.567                       H
HETATM   31  H             0        2.747  -2.802   2.334                       H
HETATM   32  C             0        2.163  -0.753  -2.585                       C
HETATM   33  H             0        1.677  -1.736  -2.722                       H
HETATM   34  H             0        1.343  -0.008  -2.523                       H
HETATM   35  H             0        2.741  -0.531  -3.497                       H
HETATM   36  C             0        6.490   0.531  -0.352                       C
HETATM   37  H             0        6.429   1.623  -0.178                       H
HETATM   38  H             0        7.175   0.126   0.411                       H
HETATM   39  H             0        6.960   0.398  -1.341                       H
HETATM   40  C             0       -2.510  -1.700   2.431                       C
HETATM   41  H             0       -3.259  -1.549   3.224                       H
```

```
HETATM   42  H            0       -2.246  -2.774   2.426                     H
HETATM   43  H            0       -1.598  -1.144   2.718                     H
HETATM   44  C            0       -2.141  -1.559  -2.611                     C
HETATM   45  H            0       -2.378  -2.601  -2.909                     H
HETATM   46  H            0       -2.442  -0.903  -3.443                     H
HETATM   47  H            0       -1.047  -1.471  -2.518                     H
HETATM   48  C            0       -6.226   0.273  -0.333                     C
HETATM   49  H            0       -6.137   1.376  -0.365                     H
HETATM   50  H            0       -6.769  -0.023  -1.247                     H
HETATM   51  H            0       -6.860   0.030   0.536                     H
HETATM   52  H            0        1.393  -3.681   0.801                     H
HETATM   53  H            0       -0.969  -3.955   0.547                     H
HETATM   54  C            0       -0.701   3.382   0.865                     C
HETATM   55  C            0        1.413   1.513   0.090                     C
HETATM   56  H            0        2.252   1.280   0.783                     H
HETATM   57  H            0       -2.261   2.136   1.542                     H
HETATM   58  H            0       -0.122   3.408   1.796                     H
HETATM   59  H            0       -2.427   2.777  -0.236                     H
HETATM   60  F            0        1.847   2.368  -0.823                     F
HETATM   61  F            0       -0.247   4.218  -0.055                     F
END
```

ac3h.pdb

```
TITLE       ac3h.pdb
HETATM    1 Ru            0       -0.038   0.956   0.255                    Ru
HETATM    2 Cl            0        0.843   0.654   2.523                    Cl
HETATM    3 Cl            0       -1.100   1.326  -1.883                    Cl
HETATM    4  C            0       -0.094  -1.076  -0.043                     C
HETATM    5  N            0        0.982  -1.871  -0.176                     N
HETATM    6  C            0        0.616  -3.281  -0.356                     C
HETATM    7  C            0       -0.908  -3.260  -0.217                     C
HETATM    8  N            0       -1.206  -1.831  -0.079                     N
HETATM    9  C            0        2.329  -1.400  -0.224                     C
HETATM   10  C            0        3.157  -1.538   0.905                     C
HETATM   11  C            0        4.453  -1.015   0.844                     C
HETATM   12  C            0        4.935  -0.377  -0.305                     C
HETATM   13  C            0        4.099  -0.287  -1.424                     C
HETATM   14  C            0        2.797  -0.796  -1.407                     C
HETATM   15  C            0       -2.532  -1.300  -0.019                     C
HETATM   16  C            0       -3.270  -1.159  -1.209                     C
HETATM   17  C            0       -4.540  -0.581  -1.138                     C
HETATM   18  C            0       -5.081  -0.143   0.076                     C
HETATM   19  C            0       -4.339  -0.328   1.247                     C
HETATM   20  C            0       -3.067  -0.913   1.224                     C
HETATM   21  C            0        2.683  -2.243   2.140                     C
HETATM   22  C            0        6.311   0.217  -0.326                     C
HETATM   23  C            0        1.919  -0.689  -2.616                     C
HETATM   24  C            0       -2.713  -1.605  -2.526                     C
HETATM   25  C            0       -6.420   0.529   0.114                     C
HETATM   26  C            0       -2.306  -1.132   2.496                     C
HETATM   27  H            0        1.112  -3.903   0.410                     H
HETATM   28  H            0        0.957  -3.639  -1.345                     H
HETATM   29  H            0       -1.270  -3.811   0.671                     H
HETATM   30  H            0       -1.429  -3.676  -1.098                     H
HETATM   31  H            0        5.099  -1.101   1.727                     H
HETATM   32  H            0        4.469   0.191  -2.341                     H
HETATM   33  H            0       -5.111  -0.445  -2.066                     H
HETATM   34  H            0       -4.762  -0.018   2.212                     H
HETATM   35  H            0        1.597  -2.131   2.288                     H
HETATM   36  H            0        3.174  -1.837   3.040                     H
HETATM   37  H            0        2.917  -3.325   2.102                     H
```

```
HETATM   38  H             0       7.022  -0.365   0.287                     H
HETATM   39  H             0       6.311   1.245   0.085                     H
HETATM   40  H             0       6.717   0.284  -1.349                     H
HETATM   41  H             0       2.497  -0.371  -3.499                     H
HETATM   42  H             0       1.098   0.041  -2.479                     H
HETATM   43  H             0       1.433  -1.652  -2.863                     H
HETATM   44  H             0      -1.622  -1.451  -2.580                     H
HETATM   45  H             0      -3.161  -1.036  -3.357                     H
HETATM   46  H             0      -2.921  -2.677  -2.719                     H
HETATM   47  H             0      -7.115   0.108  -0.633                     H
HETATM   48  H             0      -6.331   1.608  -0.114                     H
HETATM   49  H             0      -6.895   0.450   1.106                     H
HETATM   50  H             0      -2.945  -0.945   3.375                     H
HETATM   51  H             0      -1.419  -0.476   2.589                     H
HETATM   52  H             0      -1.923  -2.167   2.575                     H
HETATM   53  C             0      -1.108   2.588   0.977                     C
HETATM   54  H             0      -1.343   2.380   2.030                     H
HETATM   55  H             0      -1.936   2.859   0.307                     H
HETATM   56  C             0       1.517   1.908  -0.072                     C
HETATM   57  C             0       0.160   3.198   0.689                     C
HETATM   58  F             0       1.880   2.422  -1.237                     F
HETATM   59  F             0       0.192   4.106  -0.300                     F
HETATM   60  H             0       2.366   1.952   0.641                     H
HETATM   61  H             0       0.817   3.473   1.528                     H
END
```

ac3PA.pdb

```
TITLE      ac3PA.pdb
HETATM    1 Ru             0       0.039   0.905   0.204                    Ru
HETATM    2 Cl             0       0.937   0.439   2.452                    Cl
HETATM    3 Cl             0      -1.011   1.413  -1.888                    Cl
HETATM    4  C             0      -0.128  -1.086  -0.080                     C
HETATM    5  N             0       0.935  -1.889  -0.250                     N
HETATM    6  C             0       0.534  -3.275  -0.511                     C
HETATM    7  C             0      -0.977  -3.242  -0.270                     C
HETATM    8  N             0      -1.257  -1.809  -0.121                     N
HETATM    9  C             0       2.283  -1.417  -0.289                     C
HETATM   10  C             0       3.130  -1.661   0.811                     C
HETATM   11  C             0       4.422  -1.132   0.782                     C
HETATM   12  C             0       4.886  -0.380  -0.306                     C
HETATM   13  C             0       4.039  -0.191  -1.400                     C
HETATM   14  C             0       2.737  -0.709  -1.420                     C
HETATM   15  C             0      -2.574  -1.258  -0.033                     C
HETATM   16  C             0      -3.320  -1.076  -1.213                     C
HETATM   17  C             0      -4.583  -0.488  -1.116                     C
HETATM   18  C             0      -5.112  -0.082   0.114                     C
HETATM   19  C             0      -4.361  -0.306   1.274                     C
HETATM   20  C             0      -3.095  -0.899   1.224                     C
HETATM   21  C             0       2.673  -2.478   1.980                     C
HETATM   22  C             0       6.261   0.215  -0.286                     C
HETATM   23  C             0       1.876  -0.515  -2.630                     C
HETATM   24  C             0      -2.778  -1.494  -2.545                     C
HETATM   25  C             0      -6.444   0.600   0.183                     C
HETATM   26  C             0      -2.321  -1.154   2.481                     C
HETATM   27  H             0       1.066  -3.964   0.169                     H
HETATM   28  H             0       0.801  -3.556  -1.548                     H
HETATM   29  H             0      -1.280  -3.782   0.647                     H
HETATM   30  H             0      -1.559  -3.660  -1.110                     H
HETATM   31  H             0       5.081  -1.300   1.644                     H
HETATM   32  H             0       4.396   0.369  -2.273                     H
HETATM   33  H             0      -5.161  -0.322  -2.035                     H
```

```
HETATM   34  H             0       -4.773  -0.017   2.250                       H
HETATM   35  H             0        1.599  -2.336   2.185                       H
HETATM   36  H             0        3.218  -2.198   2.896                       H
HETATM   37  H             0        2.852  -3.559   1.816                       H
HETATM   38  H             0        7.003  -0.472   0.158                       H
HETATM   39  H             0        6.289   1.139   0.323                       H
HETATM   40  H             0        6.608   0.487  -1.297                       H
HETATM   41  H             0        2.495  -0.410  -3.537                       H
HETATM   42  H             0        1.243   0.391  -2.562                       H
HETATM   43  H             0        1.178  -1.356  -2.790                       H
HETATM   44  H             0       -1.697  -1.288  -2.627                       H
HETATM   45  H             0       -3.274  -0.946  -3.363                       H
HETATM   46  H             0       -2.938  -2.574  -2.737                       H
HETATM   47  H             0       -7.151   0.203  -0.565                       H
HETATM   48  H             0       -6.348   1.683  -0.020                       H
HETATM   49  H             0       -6.909   0.502   1.179                       H
HETATM   50  H             0       -2.924  -0.917   3.373                       H
HETATM   51  H             0       -1.391  -0.556   2.540                       H
HETATM   52  H             0       -2.007  -2.211   2.565                       H
HETATM   53  C             0       -1.027   2.329   1.092                       C
HETATM   54  H             0       -1.228   2.175   2.164                       H
HETATM   55  H             0       -1.870   2.721   0.501                       H
HETATM   56  C             0        1.493   2.190   0.176                       C
HETATM   57  C             0        0.282   3.072   0.787                       C
HETATM   58  F             0        1.928   2.704  -0.989                       F
HETATM   59  F             0        0.078   4.126  -0.053                       F
HETATM   60  H             0        2.339   2.057   0.870                       H
HETATM   61  H             0        0.700   3.424   1.748                       H
END
```

ac3i.pdb

```
TITLE       ac3i.pdb
HETATM    1 Ru             0       -0.201   0.866  -0.170                      Ru
HETATM    2 Cl             0       -0.909   0.547  -2.500                      Cl
HETATM    3 Cl             0        0.494   1.365   2.102                      Cl
HETATM    4  C             0        0.234  -1.072   0.237                       C
HETATM    5  N             0       -0.772  -1.909   0.555                       N
HETATM    6  C             0       -0.303  -3.256   0.878                       C
HETATM    7  C             0        1.222  -3.107   0.801                       C
HETATM    8  N             0        1.405  -1.718   0.363                       N
HETATM    9  C             0       -2.133  -1.518   0.348                       C
HETATM   10  C             0       -2.732  -1.758  -0.909                       C
HETATM   11  C             0       -4.020  -1.272  -1.135                       C
HETATM   12  C             0       -4.725  -0.572  -0.147                       C
HETATM   13  C             0       -4.129  -0.394   1.104                       C
HETATM   14  C             0       -2.838  -0.866   1.381                       C
HETATM   15  C             0        2.681  -1.132   0.114                       C
HETATM   16  C             0        3.496  -0.730   1.187                       C
HETATM   17  C             0        4.716  -0.108   0.896                       C
HETATM   18  C             0        5.137   0.109  -0.420                       C
HETATM   19  C             0        4.318  -0.332  -1.468                       C
HETATM   20  C             0        3.092  -0.957  -1.222                       C
HETATM   21  C             0       -2.017  -2.533  -1.972                       C
HETATM   22  C             0       -6.089  -0.022  -0.438                       C
HETATM   23  C             0       -2.252  -0.683   2.746                       C
HETATM   24  C             0        3.090  -0.968   2.609                       C
HETATM   25  C             0        6.425   0.820  -0.705                       C
HETATM   26  C             0        2.225  -1.420  -2.352                       C
HETATM   27  H             0       -0.701  -3.984   0.147                       H
HETATM   28  H             0       -0.657  -3.564   1.878                       H
HETATM   29  H             0        1.690  -3.798   0.076                       H
```

```
HETATM   30  H              0       1.717  -3.266   1.777                      H
HETATM   31  H              0      -4.479  -1.431  -2.120                      H
HETATM   32  H              0      -4.681   0.123   1.899                      H
HETATM   33  H              0       5.350   0.224   1.728                      H
HETATM   34  H              0       4.644  -0.192  -2.507                      H
HETATM   35  H              0      -0.930  -2.346  -1.966                      H
HETATM   36  H              0      -2.382  -2.259  -2.974                      H
HETATM   37  H              0      -2.175  -3.624  -1.849                      H
HETATM   38  H              0      -6.716  -0.745  -0.988                      H
HETATM   39  H              0      -6.029   0.882  -1.073                      H
HETATM   40  H              0      -6.625   0.263   0.483                      H
HETATM   41  H              0      -3.047  -0.553   3.498                      H
HETATM   42  H              0      -1.590   0.203   2.797                      H
HETATM   43  H              0      -1.629  -1.542   3.052                      H
HETATM   44  H              0       1.996  -0.943   2.734                      H
HETATM   45  H              0       3.508  -0.195   3.275                      H
HETATM   46  H              0       3.461  -1.945   2.977                      H
HETATM   47  H              0       7.162   0.681   0.104                      H
HETATM   48  H              0       6.268   1.911  -0.805                      H
HETATM   49  H              0       6.885   0.479  -1.649                      H
HETATM   50  H              0       2.767  -1.383  -3.312                      H
HETATM   51  H              0       1.314  -0.798  -2.461                      H
HETATM   52  H              0       1.874  -2.459  -2.205                      H
HETATM   53  C              0       1.242   1.848  -0.755                      C
HETATM   54  H              0       1.521   1.868  -1.830                      H
HETATM   55  H              0       1.858   2.454  -0.062                      H
HETATM   56  C              0      -1.731   2.387  -0.117                      C
HETATM   57  C              0      -0.679   3.128  -0.691                      C
HETATM   58  F              0      -2.189   2.764   1.082                      F
HETATM   59  F              0      -0.170   4.130   0.019                      F
HETATM   60  H              0      -2.494   1.913  -0.752                      H
HETATM   61  H              0      -0.580   3.229  -1.778                      H
END
```

ac3j.pdb

```
TITLE       ac3j.pdb
HETATM    1 Ru              0      -0.252   0.834  -0.151                     Ru
HETATM    2  C              0       0.071  -3.225   0.807                      C
HETATM    3  C              0       1.562  -2.899   0.906                      C
HETATM    4  H              0      -0.163  -3.882  -0.055                      H
HETATM    5  H              0       2.193  -3.545   0.270                      H
HETATM    6  C              0       0.402  -0.956   0.319                      C
HETATM    7  N              0       1.629  -1.507   0.448                      N
HETATM    8  N              0      -0.528  -1.906   0.617                      N
HETATM    9 Cl              0      -0.358   0.558  -2.579                     Cl
HETATM   10 Cl              0      -0.377   1.561   2.145                     Cl
HETATM   11  C              0      -2.434   2.163  -0.520                      C
HETATM   12  C              0       2.872  -0.850   0.217                      C
HETATM   13  C              0       3.536  -0.207   1.280                      C
HETATM   14  C              0       3.432  -0.897  -1.075                      C
HETATM   15  C              0       4.769   0.401   1.022                      C
HETATM   16  C              0       4.669  -0.280  -1.287                      C
HETATM   17  C              0       5.347   0.376  -0.253                      C
HETATM   18  H              0       5.290   0.909   1.844                      H
HETATM   19  H              0       5.112  -0.312  -2.291                      H
HETATM   20  C              0      -1.908  -1.656   0.328                      C
HETATM   21  C              0      -2.770  -1.249   1.371                      C
HETATM   22  C              0      -2.379  -1.795  -0.994                      C
HETATM   23  C              0      -4.074  -0.870   1.041                      C
HETATM   24  C              0      -3.691  -1.394  -1.279                      C
HETATM   25  C              0      -4.537  -0.900  -0.281                      C
```

```
HETATM   26  H              0       -4.742  -0.525   1.840                    H
HETATM   27  H              0       -4.053  -1.469  -2.312                    H
HETATM   28  C              0        2.913  -0.146   2.639                    C
HETATM   29  H              0        3.555   0.403   3.348                    H
HETATM   30  H              0        1.928   0.360   2.614                    H
HETATM   31  H              0        2.739  -1.152   3.065                    H
HETATM   32  C              0        2.693  -1.554  -2.198                    C
HETATM   33  H              0        2.335  -2.566  -1.931                    H
HETATM   34  H              0        1.793  -0.968  -2.477                    H
HETATM   35  H              0        3.325  -1.644  -3.096                    H
HETATM   36  C              0        6.654   1.064  -0.511                    C
HETATM   37  H              0        6.502   2.132  -0.759                    H
HETATM   38  H              0        7.315   1.038   0.372                    H
HETATM   39  H              0        7.196   0.615  -1.360                    H
HETATM   40  C              0       -2.305  -1.245   2.792                    C
HETATM   41  H              0       -3.132  -1.000   3.478                    H
HETATM   42  H              0       -1.900  -2.229   3.092                    H
HETATM   43  H              0       -1.505  -0.498   2.948                    H
HETATM   44  C              0       -1.540  -2.424  -2.063                    C
HETATM   45  H              0       -1.697  -3.521  -2.080                    H
HETATM   46  H              0       -1.805  -2.036  -3.059                    H
HETATM   47  H              0       -0.463  -2.238  -1.927                    H
HETATM   48  C              0       -5.907  -0.391  -0.611                    C
HETATM   49  H              0       -5.931   0.716  -0.585                    H
HETATM   50  H              0       -6.235  -0.700  -1.617                    H
HETATM   51  H              0       -6.663  -0.732   0.118                    H
HETATM   52  H              0        1.945  -2.979   1.941                    H
HETATM   53  H              0       -0.326  -3.713   1.714                    H
HETATM   54  C              0       -1.492   3.081  -0.838                    C
HETATM   55  H              0       -0.910   3.025  -1.764                    H
HETATM   56  C              0        1.390   1.599  -0.300                    C
HETATM   57  H              0        1.905   2.069   0.566                    H
HETATM   58  H              0        1.918   1.661  -1.276                    H
HETATM   59  H              0       -2.688   1.321  -1.179                    H
HETATM   60  F              0       -3.217   2.311   0.536                    F
HETATM   61  F              0       -1.292   4.148  -0.087                    F
END
```

at3g.pdb

```
TITLE      at3g.pdb
HETATM    1 Ru              0       -0.302   0.913   0.061                   Ru
HETATM    2  C              0       -0.492  -3.301   0.074                    C
HETATM    3  C              0        1.029  -3.198  -0.050                    C
HETATM    4  H              0       -0.955  -3.873  -0.751                    H
HETATM    5  H              0        1.410  -3.592  -1.013                    H
HETATM    6  C              0        0.133  -1.032   0.028                    C
HETATM    7  N              0        1.275  -1.755   0.030                    N
HETATM    8  N              0       -0.909  -1.903   0.029                    N
HETATM    9 Cl              0       -0.822   1.096  -2.306                   Cl
HETATM   10 Cl              0       -0.211   0.932   2.484                   Cl
HETATM   11  C              0       -1.880   2.729   0.319                    C
HETATM   12  C              0        2.593  -1.222  -0.028                    C
HETATM   13  C              0        3.329  -1.088   1.164                    C
HETATM   14  C              0        3.124  -0.819  -1.268                    C
HETATM   15  C              0        4.613  -0.540   1.094                    C
HETATM   16  C              0        4.411  -0.272  -1.293                    C
HETATM   17  C              0        5.166  -0.121  -0.124                    C
HETATM   18  H              0        5.192  -0.424   2.019                    H
HETATM   19  H              0        4.831   0.052  -2.255                    H
HETATM   20  C              0       -2.264  -1.452   0.010                    C
HETATM   21  C              0       -2.929  -1.165   1.221                    C
```

```
HETATM   22  C              0       -2.920  -1.303  -1.233                        C
HETATM   23  C              0       -4.210  -0.600   1.156                        C
HETATM   24  C              0       -4.200  -0.747  -1.247                        C
HETATM   25  C              0       -4.847  -0.361  -0.065                        C
HETATM   26  H              0       -4.725  -0.355   2.094                        H
HETATM   27  H              0       -4.704  -0.605  -2.212                        H
HETATM   28  C              0        2.710  -1.452   2.478                        C
HETATM   29  H              0        3.431  -1.344   3.305                        H
HETATM   30  H              0        1.841  -0.799   2.696                        H
HETATM   31  H              0        2.333  -2.491   2.496                        H
HETATM   32  C              0        2.305  -0.921  -2.517                        C
HETATM   33  H              0        1.822  -1.910  -2.623                        H
HETATM   34  H              0        1.483  -0.177  -2.529                        H
HETATM   35  H              0        2.921  -0.750  -3.415                        H
HETATM   36  C              0        6.528   0.504  -0.167                        C
HETATM   37  H              0        6.476   1.591   0.030                        H
HETATM   38  H              0        7.202   0.078   0.596                        H
HETATM   39  H              0        7.007   0.383  -1.153                        H
HETATM   40  C              0       -2.358  -1.544   2.553                        C
HETATM   41  H              0       -2.520  -0.754   3.303                        H
HETATM   42  H              0       -2.856  -2.461   2.926                        H
HETATM   43  H              0       -1.274  -1.732   2.521                        H
HETATM   44  C              0       -2.296  -1.795  -2.502                        C
HETATM   45  H              0       -2.549  -2.861  -2.672                        H
HETATM   46  H              0       -2.661  -1.229  -3.373                        H
HETATM   47  H              0       -1.198  -1.703  -2.493                        H
HETATM   48  C              0       -6.192   0.297  -0.115                        C
HETATM   49  H              0       -6.098   1.378  -0.334                        H
HETATM   50  H              0       -6.831  -0.128  -0.908                        H
HETATM   51  H              0       -6.731   0.211   0.843                        H
HETATM   52  H              0        1.567  -3.728   0.755                        H
HETATM   53  H              0       -0.816  -3.768   1.024                        H
HETATM   54  C              0       -0.802   3.447  -0.074                        C
HETATM   55  H              0       -0.486   3.590  -1.114                        H
HETATM   56  C              0        1.398   1.517  -0.063                        C
HETATM   57  H              0        2.228   1.296   0.642                        H
HETATM   58  H              0       -2.188   2.713   1.370                        H
HETATM   59  H              0       -2.545   2.324  -0.452                        H
HETATM   60  F              0        1.846   2.385  -0.968                        F
HETATM   61  F              0       -0.067   4.132   0.795                        F
END
```

at3h.pdb

```
TITLE       at3h.pdb
HETATM    1 Ru              0       -0.090   0.967   0.066                       Ru
HETATM    2 Cl              0        0.719   0.980   2.348                       Cl
HETATM    3 Cl              0       -1.072   1.016  -2.167                       Cl
HETATM    4  C              0       -0.067  -1.083   0.083                        C
HETATM    5  N              0        1.013  -1.880   0.035                        N
HETATM    6  C              0        0.657  -3.302   0.112                        C
HETATM    7  C              0       -0.868  -3.272   0.249                        C
HETATM    8  N              0       -1.172  -1.838   0.209                        N
HETATM    9  C              0        2.355  -1.405  -0.070                        C
HETATM   10  C              0        3.171  -1.364   1.076                        C
HETATM   11  C              0        4.457  -0.830   0.953                        C
HETATM   12  C              0        4.941  -0.349  -0.269                        C
HETATM   13  C              0        4.118  -0.434  -1.397                        C
HETATM   14  C              0        2.826  -0.965  -1.322                        C
HETATM   15  C              0       -2.495  -1.296   0.161                        C
HETATM   16  C              0       -3.192  -1.289  -1.063                        C
HETATM   17  C              0       -4.449  -0.682  -1.107                        C
```

```
HETATM   18  C              0      -5.022  -0.094   0.027                     C
HETATM   19  C              0      -4.327  -0.156   1.239                     C
HETATM   20  C              0      -3.068  -0.762   1.332                     C
HETATM   21  C              0       2.693  -1.888   2.395                     C
HETATM   22  C              0       6.307   0.264  -0.357                     C
HETATM   23  C              0       1.962  -1.056  -2.542                     C
HETATM   24  C              0      -2.621  -1.926  -2.293                     C
HETATM   25  C              0      -6.348   0.599  -0.064                     C
HETATM   26  C              0      -2.368  -0.856   2.654                     C
HETATM   27  H              0       1.158  -3.773   0.977                     H
HETATM   28  H              0       1.002  -3.829  -0.797                     H
HETATM   29  H              0      -1.229  -3.713   1.196                     H
HETATM   30  H              0      -1.387  -3.793  -0.577                     H
HETATM   31  H              0       5.095  -0.777   1.846                     H
HETATM   32  H              0       4.489  -0.080  -2.368                     H
HETATM   33  H              0      -4.988  -0.652  -2.063                     H
HETATM   34  H              0      -4.778   0.267   2.145                     H
HETATM   35  H              0       1.610  -1.734   2.533                     H
HETATM   36  H              0       3.198  -1.376   3.231                     H
HETATM   37  H              0       2.906  -2.970   2.504                     H
HETATM   38  H              0       7.044  -0.281   0.258                     H
HETATM   39  H              0       6.302   1.307   0.012                     H
HETATM   40  H              0       6.681   0.291  -1.394                     H
HETATM   41  H              0       2.546  -0.859  -3.456                     H
HETATM   42  H              0       1.125  -0.329  -2.525                     H
HETATM   43  H              0       1.500  -2.055  -2.647                     H
HETATM   44  H              0      -1.519  -1.909  -2.300                     H
HETATM   45  H              0      -2.952  -1.396  -3.200                     H
HETATM   46  H              0      -2.949  -2.980  -2.390                     H
HETATM   47  H              0      -7.047   0.067  -0.733                     H
HETATM   48  H              0      -6.237   1.620  -0.475                     H
HETATM   49  H              0      -6.830   0.701   0.923                     H
HETATM   50  H              0      -3.055  -0.608   3.480                     H
HETATM   51  H              0      -1.499  -0.175   2.730                     H
HETATM   52  H              0      -1.974  -1.872   2.840                     H
HETATM   53  C              0      -1.276   2.660   0.410                     C
HETATM   54  H              0      -1.549   2.739   1.472                     H
HETATM   55  H              0      -2.086   2.650  -0.331                     H
HETATM   56  C              0       1.399   1.923  -0.472                     C
HETATM   57  C              0      -0.035   3.269   0.039                     C
HETATM   58  F              0       1.785   2.154  -1.724                     F
HETATM   59  H              0       2.200   2.245   0.227                     H
HETATM   60  H              0       0.059   3.751  -0.946                     H
HETATM   61  F              0       0.628   3.942   0.997                     F
END
```

at3PA.pdb

```
TITLE       at3PA.pdb
HETATM    1 Ru              0       0.064   0.916   0.047                    Ru
HETATM    2 Cl              0       0.928   0.983   2.294                    Cl
HETATM    3 Cl              0      -0.909   0.897  -2.192                    Cl
HETATM    4  C              0      -0.160  -1.086   0.046                     C
HETATM    5  N              0       0.878  -1.935  -0.007                     N
HETATM    6  C              0       0.442  -3.334  -0.037                     C
HETATM    7  C              0      -1.078  -3.221   0.124                     C
HETATM    8  N              0      -1.311  -1.773   0.096                     N
HETATM    9  C              0       2.238  -1.495  -0.068                     C
HETATM   10  C              0       3.015  -1.496   1.108                     C
HETATM   11  C              0       4.308  -0.974   1.047                     C
HETATM   12  C              0       4.842  -0.469  -0.147                     C
HETATM   13  C              0       4.065  -0.528  -1.307                     C
```

```
HETATM   14  C             0        2.762  -1.043  -1.294                      C
HETATM   15  C             0       -2.609  -1.175   0.140                      C
HETATM   16  C             0       -3.401  -1.158  -1.024                      C
HETATM   17  C             0       -4.644  -0.522  -0.970                      C
HETATM   18  C             0       -5.113   0.080   0.204                      C
HETATM   19  C             0       -4.321   0.011   1.354                      C
HETATM   20  C             0       -3.071  -0.618   1.348                      C
HETATM   21  C             0        2.479  -2.049   2.392                      C
HETATM   22  C             0        6.214   0.132  -0.168                      C
HETATM   23  C             0        1.969  -1.101  -2.562                      C
HETATM   24  C             0       -2.947  -1.818  -2.289                      C
HETATM   25  C             0       -6.429   0.796   0.218                      C
HETATM   26  C             0       -2.262  -0.704   2.606                      C
HETATM   27  H             0        0.923  -3.900   0.781                      H
HETATM   28  H             0        0.743  -3.806  -0.991                      H
HETATM   29  H             0       -1.445  -3.642   1.079                      H
HETATM   30  H             0       -1.638  -3.712  -0.692                      H
HETATM   31  H             0        4.912  -0.947   1.963                      H
HETATM   32  H             0        4.479  -0.165  -2.256                      H
HETATM   33  H             0       -5.259  -0.489  -1.878                      H
HETATM   34  H             0       -4.689   0.448   2.292                      H
HETATM   35  H             0        1.403  -1.843   2.512                      H
HETATM   36  H             0        2.990  -1.603   3.260                      H
HETATM   37  H             0        2.628  -3.146   2.456                      H
HETATM   38  H             0        6.928  -0.446   0.444                      H
HETATM   39  H             0        6.206   1.158   0.246                      H
HETATM   40  H             0        6.619   0.202  -1.192                      H
HETATM   41  H             0        2.635  -1.194  -3.437                      H
HETATM   42  H             0        1.354  -0.193  -2.711                      H
HETATM   43  H             0        1.260  -1.947  -2.579                      H
HETATM   44  H             0       -1.851  -1.793  -2.399                      H
HETATM   45  H             0       -3.370  -1.312  -3.172                      H
HETATM   46  H             0       -3.277  -2.876  -2.336                      H
HETATM   47  H             0       -7.182   0.289  -0.410                      H
HETATM   48  H             0       -6.330   1.824  -0.180                      H
HETATM   49  H             0       -6.839   0.886   1.238                      H
HETATM   50  H             0       -2.890  -0.512   3.492                      H
HETATM   51  H             0       -1.430   0.027   2.632                      H
HETATM   52  H             0       -1.792  -1.696   2.734                      H
HETATM   53  C             0       -1.058   2.546   0.291                      C
HETATM   54  H             0       -1.330   2.764   1.337                      H
HETATM   55  H             0       -1.856   2.691  -0.452                      H
HETATM   56  C             0        1.455   2.145  -0.517                      C
HETATM   57  C             0        0.296   3.162  -0.085                      C
HETATM   58  F             0        1.835   2.294  -1.805                      F
HETATM   59  H             0        2.335   2.248   0.144                      H
HETATM   60  H             0        0.168   3.773  -0.997                      H
HETATM   61  F             0        0.779   3.939   0.935                      F
END
```

at3i.pdb

```
TITLE      at3i.pdb
HETATM    1 Ru             0       -0.188   0.908  -0.009                     Ru
HETATM    2 Cl             0       -1.062   0.842  -2.279                     Cl
HETATM    3 Cl             0        0.667   1.087   2.268                     Cl
HETATM    4  C             0        0.216  -1.076   0.067                      C
HETATM    5  N             0       -0.790  -1.960   0.212                      N
HETATM    6  C             0       -0.312  -3.311   0.499                      C
HETATM    7  C             0        1.194  -3.184   0.255                      C
HETATM    8  N             0        1.388  -1.735   0.125                      N
HETATM    9  C             0       -2.155  -1.540   0.162                      C
```

```
HETATM   10  C              0       -2.865  -1.712  -1.047                      C
HETATM   11  C              0       -4.159  -1.205  -1.136                      C
HETATM   12  C              0       -4.758  -0.524  -0.065                      C
HETATM   13  C              0       -4.047  -0.396   1.127                      C
HETATM   14  C              0       -2.749  -0.914   1.274                      C
HETATM   15  C              0        2.669  -1.131  -0.043                      C
HETATM   16  C              0        3.504  -0.951   1.073                      C
HETATM   17  C              0        4.736  -0.312   0.883                      C
HETATM   18  C              0        5.147   0.138  -0.375                      C
HETATM   19  C              0        4.306  -0.083  -1.473                      C
HETATM   20  C              0        3.068  -0.718  -1.331                      C
HETATM   21  C              0       -2.234  -2.406  -2.213                      C
HETATM   22  C              0       -6.131   0.057  -0.215                      C
HETATM   23  C              0       -2.086  -0.852   2.617                      C
HETATM   24  C              0        3.110  -1.440   2.432                      C
HETATM   25  C              0        6.449   0.860  -0.546                      C
HETATM   26  C              0        2.188  -0.954  -2.520                      C
HETATM   27  H              0       -0.798  -4.051  -0.160                      H
HETATM   28  H              0       -0.555  -3.584   1.545                      H
HETATM   29  H              0        1.523  -3.693  -0.672                      H
HETATM   30  H              0        1.803  -3.582   1.085                      H
HETATM   31  H              0       -4.707  -1.314  -2.081                      H
HETATM   32  H              0       -4.508   0.112   1.985                      H
HETATM   33  H              0        5.388  -0.154   1.753                      H
HETATM   34  H              0        4.624   0.241  -2.473                      H
HETATM   35  H              0       -1.192  -2.075  -2.363                      H
HETATM   36  H              0       -2.782  -2.192  -3.145                      H
HETATM   37  H              0       -2.225  -3.507  -2.085                      H
HETATM   38  H              0       -6.845  -0.678  -0.627                      H
HETATM   39  H              0       -6.130   0.914  -0.915                      H
HETATM   40  H              0       -6.535   0.419   0.745                      H
HETATM   41  H              0       -2.658  -1.451   3.351                      H
HETATM   42  H              0       -2.045   0.181   3.003                      H
HETATM   43  H              0       -1.047  -1.215   2.608                      H
HETATM   44  H              0        2.018  -1.423   2.574                      H
HETATM   45  H              0        3.548  -0.807   3.222                      H
HETATM   46  H              0        3.470  -2.473   2.611                      H
HETATM   47  H              0        7.158   0.634   0.268                      H
HETATM   48  H              0        6.302   1.957  -0.546                      H
HETATM   49  H              0        6.938   0.611  -1.504                      H
HETATM   50  H              0        2.728  -0.754  -3.460                      H
HETATM   51  H              0        1.284  -0.312  -2.515                      H
HETATM   52  H              0        1.820  -1.996  -2.562                      H
HETATM   53  C              0        1.307   1.858  -0.517                      C
HETATM   54  H              0        1.432   2.223  -1.558                      H
HETATM   55  H              0        2.115   2.103   0.203                      H
HETATM   56  C              0       -1.638   2.503   0.321                      C
HETATM   57  C              0       -0.497   3.265   0.030                      C
HETATM   58  F              0       -2.081   2.453   1.592                      F
HETATM   59  H              0       -2.435   2.375  -0.425                      H
HETATM   60  H              0        0.101   3.728   0.825                      H
HETATM   61  F              0       -0.429   3.882  -1.151                      F
END
```

at3j.pdb

```
TITLE      at3j.pdb
HETATM    1 Ru              0       -0.201   0.982   0.087                     Ru
HETATM    2  C              0       -0.244  -3.260   0.062                      C
HETATM    3  C              0        1.270  -3.097   0.178                      C
HETATM    4  H              0       -0.555  -3.710  -0.901                      H
HETATM    5  H              0        1.828  -3.605  -0.629                      H
```

```
HETATM    6  C              0        0.284  -0.973   0.083                          C
HETATM    7  N              0        1.456  -1.644   0.084                          N
HETATM    8  N              0       -0.721  -1.881   0.147                          N
HETATM    9 Cl              0       -0.542   1.201  -2.325                         Cl
HETATM   10 Cl              0       -0.070   0.944   2.518                         Cl
HETATM   11  C              0       -1.908   2.637   0.011                          C
HETATM   12  C              0        2.757  -1.072  -0.022                          C
HETATM   13  C              0        3.529  -0.873   1.138                          C
HETATM   14  C              0        3.250  -0.732  -1.297                          C
HETATM   15  C              0        4.802  -0.309   0.999                          C
HETATM   16  C              0        4.527  -0.168  -1.389                          C
HETATM   17  C              0        5.313   0.056  -0.253                          C
HETATM   18  H              0        5.407  -0.140   1.899                          H
HETATM   19  H              0        4.917   0.102  -2.379                          H
HETATM   20  C              0       -2.097  -1.519  -0.003                          C
HETATM   21  C              0       -2.907  -1.382   1.145                          C
HETATM   22  C              0       -2.638  -1.371  -1.297                          C
HETATM   23  C              0       -4.234  -0.981   0.976                          C
HETATM   24  C              0       -3.967  -0.950  -1.416                          C
HETATM   25  C              0       -4.770  -0.728  -0.293                          C
HETATM   26  H              0       -4.867  -0.859   1.864                          H
HETATM   27  H              0       -4.387  -0.805  -2.420                          H
HETATM   28  C              0        2.978  -1.201   2.490                          C
HETATM   29  H              0        3.672  -0.892   3.288                          H
HETATM   30  H              0        2.011  -0.688   2.663                          H
HETATM   31  H              0        2.795  -2.284   2.621                          H
HETATM   32  C              0        2.413  -0.950  -2.518                          C
HETATM   33  H              0        2.000  -1.975  -2.561                          H
HETATM   34  H              0        1.543  -0.263  -2.545                          H
HETATM   35  H              0        2.996  -0.784  -3.438                          H
HETATM   36  C              0        6.663   0.696  -0.372                          C
HETATM   37  H              0        6.583   1.800  -0.388                          H
HETATM   38  H              0        7.320   0.437   0.475                          H
HETATM   39  H              0        7.176   0.406  -1.305                          H
HETATM   40  C              0       -2.371  -1.692   2.507                          C
HETATM   41  H              0       -3.152  -1.574   3.275                          H
HETATM   42  H              0       -1.997  -2.732   2.571                          H
HETATM   43  H              0       -1.529  -1.028   2.772                          H
HETATM   44  C              0       -1.863  -1.745  -2.524                          C
HETATM   45  H              0       -2.068  -2.799  -2.798                          H
HETATM   46  H              0       -2.149  -1.119  -3.384                          H
HETATM   47  H              0       -0.774  -1.630  -2.400                          H
HETATM   48  C              0       -6.174  -0.225  -0.439                          C
HETATM   49  H              0       -6.217   0.873  -0.311                          H
HETATM   50  H              0       -6.592  -0.446  -1.436                          H
HETATM   51  H              0       -6.849  -0.655   0.321                          H
HETATM   52  H              0        1.666  -3.473   1.140                          H
HETATM   53  H              0       -0.679  -3.875   0.870                          H
HETATM   54  C              0       -0.863   3.356   0.515                          C
HETATM   55  C              0        1.472   1.671  -0.080                          C
HETATM   56  H              0        2.284   1.458   0.645                          H
HETATM   57  H              0        1.717   2.389  -0.892                          H
HETATM   58  H              0       -0.617   3.450   1.578                          H
HETATM   59  H              0       -2.244   2.676  -1.030                          H
HETATM   60  F              0       -2.748   2.009   0.847                          F
HETATM   61  F              0       -0.193   4.172  -0.300                          F
END
```

s3k.pdb

```
TITLE       s3k.pdb
HETATM    1 Ru              0        0.220  -1.025   0.003                         Ru
```

```
HETATM    2  C              0       0.114   3.159   0.278                       C
HETATM    3  C              0      -1.396   2.933   0.348                       C
HETATM    4  H              0       0.424   3.664  -0.659                       H
HETATM    5  H              0      -1.955   3.473  -0.437                       H
HETATM    6  C              0      -0.327   0.857   0.170                       C
HETATM    7  N              0      -1.527   1.484   0.162                       N
HETATM    8  N              0       0.642   1.799   0.320                       N
HETATM    9 Cl              0       0.416  -1.010  -2.404                      Cl
HETATM   10 Cl              0       0.236  -1.476   2.402                      Cl
HETATM   11  C              0       2.349  -2.605   0.114                       C
HETATM   12  C              0      -2.803   0.863   0.048                       C
HETATM   13  C              0      -3.490   0.455   1.209                       C
HETATM   14  C              0      -3.376   0.712  -1.230                       C
HETATM   15  C              0      -4.761  -0.111   1.066                       C
HETATM   16  C              0      -4.648   0.138  -1.328                       C
HETATM   17  C              0      -5.352  -0.280  -0.193                       C
HETATM   18  H              0      -5.303  -0.431   1.965                       H
HETATM   19  H              0      -5.099   0.016  -2.321                       H
HETATM   20  C              0       2.025   1.462   0.184                       C
HETATM   21  C              0       2.781   1.181   1.343                       C
HETATM   22  C              0       2.618   1.441  -1.096                       C
HETATM   23  C              0       4.107   0.764   1.184                       C
HETATM   24  C              0       3.948   1.022  -1.204                       C
HETATM   25  C              0       4.694   0.651  -0.082                       C
HETATM   26  H              0       4.698   0.529   2.078                       H
HETATM   27  H              0       4.406   0.972  -2.201                       H
HETATM   28  C              0      -2.847   0.577   2.555                       C
HETATM   29  H              0      -3.559   0.340   3.362                       H
HETATM   30  H              0      -1.985  -0.115   2.650                       H
HETATM   31  H              0      -2.448   1.590   2.744                       H
HETATM   32  C              0      -2.611   1.114  -2.452                       C
HETATM   33  H              0      -2.231   2.151  -2.389                       H
HETATM   34  H              0      -1.723   0.466  -2.593                       H
HETATM   35  H              0      -3.234   1.039  -3.359                       H
HETATM   36  C              0      -6.701  -0.920  -0.322                       C
HETATM   37  H              0      -6.618  -2.023  -0.361                       H
HETATM   38  H              0      -7.353  -0.684   0.536                       H
HETATM   39  H              0      -7.220  -0.607  -1.243                       H
HETATM   40  C              0       2.194   1.371   2.707                       C
HETATM   41  H              0       2.947   1.184   3.490                       H
HETATM   42  H              0       1.818   2.402   2.846                       H
HETATM   43  H              0       1.346   0.685   2.887                       H
HETATM   44  C              0       1.885   1.924  -2.309                       C
HETATM   45  H              0       2.071   3.005  -2.469                       H
HETATM   46  H              0       2.223   1.396  -3.215                       H
HETATM   47  H              0       0.797   1.769  -2.240                       H
HETATM   48  C              0       6.085   0.118  -0.235                       C
HETATM   49  H              0       6.065  -0.970  -0.439                       H
HETATM   50  H              0       6.620   0.586  -1.079                       H
HETATM   51  H              0       6.689   0.262   0.678                       H
HETATM   52  H              0      -1.828   3.233   1.323                       H
HETATM   53  H              0       0.501   3.759   1.120                       H
HETATM   54  C              0       1.294  -3.390  -0.172                       C
HETATM   55  H              0       1.119  -3.760  -1.188                       H
HETATM   56  C              0      -1.464  -1.693  -0.112                       C
HETATM   57  H              0      -2.036  -2.020   0.785                       H
HETATM   58  H              0      -1.969  -1.841  -1.091                       H
HETATM   59  H              0       2.602  -2.232   1.116                       H
HETATM   60  H              0       0.660  -3.729   0.652                       H
HETATM   61  F              0       3.233  -2.244  -0.807                       F
END
```

s3l.pdb

```
TITLE      s3l.pdb
REMARK   1 File created by GaussView 5.0.8
HETATM    1 Ru           0        0.242  -0.972   0.083                     Ru
HETATM    2 Cl           0       -0.283  -1.097   2.475                     Cl
HETATM    3 Cl           0        0.779  -1.104  -2.285                     Cl
HETATM    4  C           0       -0.255   0.983   0.001                      C
HETATM    5  N           0       -1.443   1.611  -0.085                      N
HETATM    6  C           0       -1.296   3.070  -0.006                      C
HETATM    7  C           0        0.220   3.273  -0.088                      C
HETATM    8  N           0        0.724   1.911   0.074                      N
HETATM    9  H           0       -1.721   3.443   0.946                      H
HETATM   10  H           0       -1.847   3.557  -0.830                      H
HETATM   11  H           0        0.612   3.938   0.701                      H
HETATM   12  H           0        0.542   3.687  -1.064                      H
HETATM   13  C           0       -2.709   0.957  -0.119                      C
HETATM   14  C           0       -3.444   0.783   1.069                      C
HETATM   15  C           0       -4.672   0.115   0.998                      C
HETATM   16  C           0       -5.175  -0.372  -0.213                      C
HETATM   17  C           0       -4.431  -0.166  -1.381                      C
HETATM   18  C           0       -3.199   0.496  -1.356                      C
HETATM   19  C           0        2.093   1.496   0.067                      C
HETATM   20  C           0        2.780   1.351  -1.156                      C
HETATM   21  C           0        4.052   0.775  -1.136                      C
HETATM   22  C           0        4.654   0.366   0.060                      C
HETATM   23  C           0        3.990   0.613   1.267                      C
HETATM   24  C           0        2.714   1.188   1.297                      C
HETATM   25  C           0       -2.938   1.307   2.378                      C
HETATM   26  C           0       -6.473  -1.121  -0.256                      C
HETATM   27  C           0       -2.411   0.706  -2.611                      C
HETATM   28  C           0        2.183   1.833  -2.442                      C
HETATM   29  C           0        5.971  -0.346   0.043                      C
HETATM   30  C           0        2.045   1.502   2.599                      C
HETATM   31  H           0       -5.246  -0.034   1.922                      H
HETATM   32  H           0       -4.820  -0.526  -2.343                      H
HETATM   33  H           0        4.575   0.618  -2.087                      H
HETATM   34  H           0        4.478   0.357   2.217                      H
HETATM   35  H           0       -3.162   2.386   2.500                      H
HETATM   36  H           0       -1.849   1.168   2.483                      H
HETATM   37  H           0       -3.415   0.786   3.224                      H
HETATM   38  H           0       -7.172  -0.782   0.528                      H
HETATM   39  H           0       -6.318  -2.205  -0.093                      H
HETATM   40  H           0       -6.978  -1.017  -1.231                      H
HETATM   41  H           0       -3.004   0.443  -3.503                      H
HETATM   42  H           0       -1.488   0.091  -2.627                      H
HETATM   43  H           0       -2.081   1.756  -2.722                      H
HETATM   44  H           0        1.090   1.698  -2.471                      H
HETATM   45  H           0        2.596   1.282  -3.302                      H
HETATM   46  H           0        2.403   2.908  -2.599                      H
HETATM   47  H           0        6.634   0.023  -0.759                      H
HETATM   48  H           0        5.825  -1.427  -0.142                      H
HETATM   49  H           0        6.507  -0.254   1.003                      H
HETATM   50  H           0        2.755   1.411   3.437                      H
HETATM   51  H           0        1.198   0.819   2.804                      H
HETATM   52  H           0        1.640   2.531   2.611                      H
HETATM   53  C           0       -1.232  -2.015  -0.265                      C
HETATM   54  H           0       -1.428  -2.411  -1.283                      H
HETATM   55  H           0       -1.955  -2.293   0.530                      H
HETATM   56  C           0        1.805  -2.499   0.466                      C
HETATM   57  C           0        0.670  -3.289   0.322                      C
HETATM   58  F           0        2.790  -2.541  -0.438                      F
```

```
HETATM   59  H             0        2.151  -2.135   1.444                       H
HETATM   60  H             0        0.565  -3.898  -0.584                       H
HETATM   61  H             0        0.113  -3.568   1.222                       H
END
```

s3PB.pdb

```
TITLE       s3PB.pdb
HETATM    1 Ru             0        0.012  -1.031  -0.083                      Ru
HETATM    2  C             0       -0.967   3.145  -0.150                       C
HETATM    3  C             0        0.535   3.219   0.133                       C
HETATM    4  H             0       -1.577   3.708   0.578                       H
HETATM    5  H             0        0.769   3.667   1.119                       H
HETATM    6  C             0       -0.119   0.986   0.035                       C
HETATM    7  N             0        0.939   1.810   0.111                       N
HETATM    8  N             0       -1.248   1.708  -0.054                       N
HETATM    9 Cl             0       -0.861  -1.471   2.149                      Cl
HETATM   10 Cl             0        0.775  -0.753  -2.370                      Cl
HETATM   11  C             0       -1.136  -2.514  -0.717                       C
HETATM   12  H             0       -1.311  -2.524  -1.804                       H
HETATM   13  C             0        2.284   1.340   0.220                       C
HETATM   14  C             0        3.128   1.380  -0.907                       C
HETATM   15  C             0        2.731   0.819   1.450                       C
HETATM   16  C             0        4.401   0.815  -0.800                       C
HETATM   17  C             0        4.017   0.271   1.512                       C
HETATM   18  C             0        4.854   0.239   0.392                       C
HETATM   19  H             0        5.054   0.814  -1.683                       H
HETATM   20  H             0        4.372  -0.141   2.466                       H
HETATM   21  C             0       -2.560   1.136  -0.067                       C
HETATM   22  C             0       -3.129   0.709  -1.281                       C
HETATM   23  C             0       -3.258   1.018   1.151                       C
HETATM   24  C             0       -4.391   0.104  -1.245                       C
HETATM   25  C             0       -4.515   0.409   1.141                       C
HETATM   26  C             0       -5.088  -0.068  -0.044                       C
HETATM   27  H             0       -4.841  -0.235  -2.187                       H
HETATM   28  H             0       -5.058   0.301   2.088                       H
HETATM   29  C             0        2.695   2.025  -2.188                       C
HETATM   30  H             0        3.183   1.550  -3.054                       H
HETATM   31  H             0        1.608   1.942  -2.348                       H
HETATM   32  H             0        2.968   3.099  -2.209                       H
HETATM   33  C             0        1.860   0.841   2.670                       C
HETATM   34  H             0        1.265   1.770   2.745                       H
HETATM   35  H             0        1.128   0.009   2.679                       H
HETATM   36  H             0        2.465   0.754   3.587                       H
HETATM   37  C             0        6.197  -0.420   0.456                       C
HETATM   38  H             0        6.144  -1.461   0.083                       H
HETATM   39  H             0        6.945   0.099  -0.168                       H
HETATM   40  H             0        6.586  -0.470   1.487                       H
HETATM   41  C             0       -2.410   0.893  -2.582                       C
HETATM   42  H             0       -3.071   0.666  -3.435                       H
HETATM   43  H             0       -2.042   1.927  -2.711                       H
HETATM   44  H             0       -1.517   0.243  -2.666                       H
HETATM   45  C             0       -2.652   1.516   2.427                       C
HETATM   46  H             0       -2.391   2.590   2.377                       H
HETATM   47  H             0       -3.345   1.384   3.274                       H
HETATM   48  H             0       -1.729   0.958   2.665                       H
HETATM   49  C             0       -6.417  -0.760  -0.022                       C
HETATM   50  H             0       -6.302  -1.836   0.210                       H
HETATM   51  H             0       -7.087  -0.343   0.749                       H
HETATM   52  H             0       -6.933  -0.697  -0.995                       H
HETATM   53  H             0        1.093   3.788  -0.631                       H
HETATM   54  H             0       -1.232   3.515  -1.159                       H
```

```
HETATM   55  C             0        0.154  -3.246  -0.277                     C
HETATM   56  H             0       -0.108  -3.924   0.552                     H
HETATM   57  H             0        0.590  -3.757  -1.152                     H
HETATM   58  C             0        1.366  -2.371   0.274                     C
HETATM   59  H             0        1.603  -2.565   1.335                     H
HETATM   60  H             0       -2.016  -2.735  -0.093                     H
HETATM   61  F             0        2.488  -2.496  -0.462                     F
END
```

s3m.pdb

```
TITLE      s3m.pdb
HETATM    1 Ru             0        0.219  -1.089  -0.013                    Ru
HETATM    2 Cl             0        0.931  -0.973  -2.345                    Cl
HETATM    3 Cl             0       -0.281  -1.382   2.350                    Cl
HETATM    4  C             0       -0.007   0.926   0.232                     C
HETATM    5  N             0        1.061   1.721   0.441                     N
HETATM    6  C             0        0.692   3.128   0.615                     C
HETATM    7  C             0       -0.832   3.097   0.481                     C
HETATM    8  N             0       -1.126   1.671   0.287                     N
HETATM    9  C             0        2.399   1.246   0.254                     C
HETATM   10  C             0        2.985   1.335  -1.024                     C
HETATM   11  C             0        4.254   0.782  -1.214                     C
HETATM   12  C             0        4.948   0.160  -0.170                     C
HETATM   13  C             0        4.368   0.141   1.103                     C
HETATM   14  C             0        3.103   0.690   1.344                     C
HETATM   15  C             0       -2.447   1.185   0.045                     C
HETATM   16  C             0       -3.312   0.939   1.127                     C
HETATM   17  C             0       -4.598   0.461   0.854                     C
HETATM   18  C             0       -5.032   0.223  -0.456                     C
HETATM   19  C             0       -4.158   0.499  -1.512                     C
HETATM   20  C             0       -2.866   0.989  -1.285                     C
HETATM   21  C             0        2.297   2.038  -2.154                     C
HETATM   22  C             0        6.279  -0.482  -0.415                     C
HETATM   23  C             0        2.533   0.714   2.728                     C
HETATM   24  C             0       -2.862   1.164   2.537                     C
HETATM   25  C             0       -6.400  -0.331  -0.716                     C
HETATM   26  C             0       -1.951   1.294  -2.430                     C
HETATM   27  H             0        1.181   3.749  -0.158                     H
HETATM   28  H             0        1.032   3.498   1.600                     H
HETATM   29  H             0       -1.204   3.681  -0.383                     H
HETATM   30  H             0       -1.351   3.477   1.379                     H
HETATM   31  H             0        4.706   0.826  -2.214                     H
HETATM   32  H             0        4.919  -0.302   1.943                     H
HETATM   33  H             0       -5.276   0.260   1.693                     H
HETATM   34  H             0       -4.490   0.342  -2.547                     H
HETATM   35  H             0        2.573   3.112  -2.180                     H
HETATM   36  H             0        1.199   1.975  -2.086                     H
HETATM   37  H             0        2.583   1.603  -3.125                     H
HETATM   38  H             0        6.871   0.066  -1.168                     H
HETATM   39  H             0        6.160  -1.513  -0.799                     H
HETATM   40  H             0        6.881  -0.553   0.507                     H
HETATM   41  H             0        2.219   1.732   3.023                     H
HETATM   42  H             0        3.277   0.369   3.465                     H
HETATM   43  H             0        1.639   0.068   2.826                     H
HETATM   44  H             0       -2.608   2.223   2.732                     H
HETATM   45  H             0       -1.964   0.562   2.766                     H
HETATM   46  H             0       -3.650   0.883   3.255                     H
HETATM   47  H             0       -7.151   0.079  -0.019                     H
HETATM   48  H             0       -6.740  -0.126  -1.745                     H
HETATM   49  H             0       -6.419  -1.430  -0.585                     H
HETATM   50  H             0       -1.520   2.311  -2.357                     H
```

```
HETATM   51  H             0       -2.483   1.228  -3.393                         H
HETATM   52  H             0       -1.092   0.596  -2.477                         H
HETATM   53  C             0       -1.302  -1.982  -0.553                         C
HETATM   54  F             0       -2.314  -2.390   0.199                         F
HETATM   55  H             0       -1.552  -2.153  -1.622                         H
HETATM   56  C             0        1.482  -2.795   0.146                         C
HETATM   57  C             0        0.254  -3.367  -0.283                         C
HETATM   58  H             0        2.293  -2.676  -0.586                         H
HETATM   59  H             0        1.760  -2.881   1.205                         H
HETATM   60  H             0        0.189  -3.705  -1.326                         H
HETATM   61  H             0       -0.351  -3.912   0.454                         H
END
```

s3n.pdb

```
TITLE       s3n.pdb
HETATM    1 Ru             0       -0.325   1.016   0.272                        Ru
HETATM    2  C             0       -0.465  -3.175  -0.206                         C
HETATM    3  C             0        1.057  -3.058  -0.292                         C
HETATM    4  H             0       -0.913  -3.653  -1.098                         H
HETATM    5  H             0        1.459  -3.366  -1.277                         H
HETATM    6  C             0        0.153  -0.911  -0.016                         C
HETATM    7  N             0        1.297  -1.627  -0.080                         N
HETATM    8  N             0       -0.885  -1.781  -0.105                         N
HETATM    9 Cl             0       -0.790   1.541  -2.049                        Cl
HETATM   10 Cl             0       -0.277   0.641   2.673                        Cl
HETATM   11  C             0       -2.011   2.645   0.703                         C
HETATM   12  C             0        2.611  -1.082  -0.141                         C
HETATM   13  C             0        3.399  -1.047   1.024                         C
HETATM   14  C             0        3.088  -0.570  -1.363                         C
HETATM   15  C             0        4.677  -0.481   0.947                         C
HETATM   16  C             0        4.369  -0.010  -1.396                         C
HETATM   17  C             0        5.173   0.048  -0.252                         C
HETATM   18  H             0        5.296  -0.443   1.853                         H
HETATM   19  H             0        4.746   0.395  -2.344                         H
HETATM   20  C             0       -2.239  -1.329  -0.156                         C
HETATM   21  C             0       -2.988  -1.195   1.032                         C
HETATM   22  C             0       -2.810  -1.023  -1.412                         C
HETATM   23  C             0       -4.264  -0.623   0.951                         C
HETATM   24  C             0       -4.088  -0.463  -1.444                         C
HETATM   25  C             0       -4.816  -0.226  -0.271                         C
HETATM   26  H             0       -4.844  -0.495   1.875                         H
HETATM   27  H             0       -4.524  -0.195  -2.415                         H
HETATM   28  C             0        2.847  -1.535   2.328                         C
HETATM   29  H             0        3.592  -1.448   3.135                         H
HETATM   30  H             0        1.953  -0.951   2.625                         H
HETATM   31  H             0        2.528  -2.593   2.284                         H
HETATM   32  C             0        2.223  -0.584  -2.586                         C
HETATM   33  H             0        1.745  -1.567  -2.749                         H
HETATM   34  H             0        1.397   0.152  -2.512                         H
HETATM   35  H             0        2.804  -0.339  -3.490                         H
HETATM   36  C             0        6.527   0.689  -0.301                         C
HETATM   37  H             0        6.460   1.780  -0.126                         H
HETATM   38  H             0        7.205   0.286   0.470                         H
HETATM   39  H             0        7.011   0.558  -1.284                         H
HETATM   40  C             0       -2.503  -1.739   2.341                         C
HETATM   41  H             0       -2.778  -1.079   3.180                         H
HETATM   42  H             0       -2.967  -2.727   2.533                         H
HETATM   43  H             0       -1.411  -1.857   2.379                         H
HETATM   44  C             0       -2.101  -1.353  -2.690                         C
HETATM   45  H             0       -2.338  -2.389  -3.007                         H
HETATM   46  H             0       -2.412  -0.680  -3.505                         H
```

```
HETATM   47  H            0      -1.007  -1.268  -2.604                     H
HETATM   48  C            0      -6.152   0.449  -0.327                     C
HETATM   49  H            0      -6.039   1.550  -0.342                     H
HETATM   50  H            0      -6.714   0.180  -1.238                     H
HETATM   51  H            0      -6.778   0.204   0.548                     H
HETATM   52  H            0       1.581  -3.656   0.474                     H
HETATM   53  H            0      -0.804  -3.745   0.681                     H
HETATM   54  C            0      -0.878   3.397   0.700                     C
HETATM   55  H            0      -0.537   3.900  -0.212                     H
HETATM   56  C            0       1.362   1.670   0.239                     C
HETATM   57  H            0       2.227   1.241   0.787                     H
HETATM   58  H            0      -2.413   2.235   1.640                     H
HETATM   59  H            0      -0.331   3.605   1.629                     H
HETATM   60  H            0      -2.623   2.541  -0.202                     H
HETATM   61  F            0       1.775   2.768  -0.384                     F
END
```

a3k.pdb

```
TITLE      a3k.pdb
HETATM    1 Ru            0      -0.271   0.974   0.170                    Ru
HETATM    2  C            0      -0.372  -3.236  -0.070                     C
HETATM    3  C            0       1.147  -3.094   0.027                     C
HETATM    4  H            0      -0.703  -3.623  -1.054                     H
HETATM    5  H            0       1.684  -3.569  -0.813                     H
HETATM    6  C            0       0.185  -0.959   0.068                     C
HETATM    7  N            0       1.351  -1.642   0.003                     N
HETATM    8  N            0      -0.831  -1.861   0.112                     N
HETATM    9 Cl            0      -0.701   1.339  -2.187                    Cl
HETATM   10 Cl            0      -0.080   0.922   2.606                    Cl
HETATM   11  C            0      -1.890   2.715   0.425                     C
HETATM   12  C            0       2.653  -1.073  -0.091                     C
HETATM   13  C            0       3.442  -0.934   1.067                     C
HETATM   14  C            0       3.131  -0.668  -1.353                     C
HETATM   15  C            0       4.717  -0.374   0.940                     C
HETATM   16  C            0       4.411  -0.108  -1.434                     C
HETATM   17  C            0       5.214   0.051  -0.299                     C
HETATM   18  H            0       5.335  -0.256   1.839                     H
HETATM   19  H            0       4.788   0.212  -2.414                     H
HETATM   20  C            0      -2.193  -1.444  -0.013                     C
HETATM   21  C            0      -2.970  -1.264   1.153                     C
HETATM   22  C            0      -2.745  -1.237  -1.294                     C
HETATM   23  C            0      -4.269  -0.763   1.012                     C
HETATM   24  C            0      -4.049  -0.738  -1.385                     C
HETATM   25  C            0      -4.813  -0.471  -0.244                     C
HETATM   26  H            0      -4.877  -0.611   1.914                     H
HETATM   27  H            0      -4.475  -0.553  -2.379                     H
HETATM   28  C            0       2.901  -1.313   2.410                     C
HETATM   29  H            0       3.636  -1.111   3.207                     H
HETATM   30  H            0       1.982  -0.740   2.644                     H
HETATM   31  H            0       2.636  -2.384   2.476                     H
HETATM   32  C            0       2.274  -0.812  -2.572                     C
HETATM   33  H            0       1.840  -1.826  -2.659                     H
HETATM   34  H            0       1.417  -0.108  -2.554                     H
HETATM   35  H            0       2.849  -0.612  -3.491                     H
HETATM   36  C            0       6.567   0.686  -0.401                     C
HETATM   37  H            0       6.512   1.777  -0.223                     H
HETATM   38  H            0       7.272   0.281   0.345                     H
HETATM   39  H            0       7.013   0.552  -1.401                     H
HETATM   40  C            0      -2.441  -1.649   2.499                     C
HETATM   41  H            0      -3.206  -1.504   3.279                     H
HETATM   42  H            0      -2.138  -2.712   2.524                     H
```

```
HETATM   43  H              0       -1.555  -1.051   2.780                      H
HETATM   44  C              0       -2.000  -1.609  -2.539                      C
HETATM   45  H              0       -2.227  -2.655  -2.828                      H
HETATM   46  H              0       -2.288  -0.964  -3.384                      H
HETATM   47  H              0       -0.908  -1.516  -2.429                      H
HETATM   48  C              0       -6.187   0.116  -0.366                      C
HETATM   49  H              0       -6.146   1.222  -0.405                      H
HETATM   50  H              0       -6.697  -0.212  -1.287                      H
HETATM   51  H              0       -6.827  -0.147   0.493                      H
HETATM   52  H              0        1.554  -3.523   0.963                      H
HETATM   53  H              0       -0.795  -3.898   0.706                      H
HETATM   54  C              0       -0.795   3.485   0.640                      C
HETATM   55  C              0        1.414   1.636   0.007                      C
HETATM   56  H              0        2.194   1.505   0.787                      H
HETATM   57  H              0        1.700   2.257  -0.871                      H
HETATM   58  H              0       -2.338   2.195   1.281                      H
HETATM   59  H              0       -0.253   3.559   1.591                      H
HETATM   60  H              0       -2.437   2.787  -0.521                      H
HETATM   61  F              0       -0.335   4.308  -0.291                      F
END
```

a3l.pdb

```
TITLE      a3l.pdb
HETATM    1 Ru              0       -0.239   0.952  -0.163                     Ru
HETATM    2 Cl              0       -0.674   0.713  -2.563                     Cl
HETATM    3 Cl              0        0.049   1.406   2.194                     Cl
HETATM    4  C              0        0.131  -1.030   0.029                      C
HETATM    5  N              0       -0.900  -1.897   0.087                      N
HETATM    6  C              0       -0.463  -3.276   0.300                      C
HETATM    7  C              0        1.052  -3.170   0.107                      C
HETATM    8  N              0        1.285  -1.720   0.092                      N
HETATM    9  C              0       -2.241  -1.410   0.169                      C
HETATM   10  C              0       -3.039  -1.382  -0.995                      C
HETATM   11  C              0       -4.296  -0.776  -0.921                      C
HETATM   12  C              0       -4.778  -0.223   0.273                      C
HETATM   13  C              0       -3.995  -0.331   1.427                      C
HETATM   14  C              0       -2.732  -0.934   1.403                      C
HETATM   15  C              0        2.589  -1.139   0.052                      C
HETATM   16  C              0        3.247  -0.864   1.264                      C
HETATM   17  C              0        4.513  -0.271   1.214                      C
HETATM   18  C              0        5.127   0.045  -0.003                      C
HETATM   19  C              0        4.456  -0.261  -1.194                      C
HETATM   20  C              0        3.189  -0.855  -1.189                      C
HETATM   21  C              0       -2.585  -2.033  -2.264                      C
HETATM   22  C              0       -6.098   0.485   0.307                      C
HETATM   23  C              0       -1.991  -1.159   2.687                      C
HETATM   24  C              0        2.589  -1.163   2.575                      C
HETATM   25  C              0        6.464   0.721  -0.032                      C
HETATM   26  C              0        2.475  -1.158  -2.470                      C
HETATM   27  H              0       -0.947  -3.955  -0.424                      H
HETATM   28  H              0       -0.744  -3.612   1.318                      H
HETATM   29  H              0        1.392  -3.617  -0.847                      H
HETATM   30  H              0        1.630  -3.647   0.918                      H
HETATM   31  H              0       -4.914  -0.730  -1.827                      H
HETATM   32  H              0       -4.376   0.058   2.380                      H
HETATM   33  H              0        5.031  -0.048   2.156                      H
HETATM   34  H              0        4.931  -0.034  -2.157                      H
HETATM   35  H              0       -2.889  -3.099  -2.286                      H
HETATM   36  H              0       -1.493  -1.988  -2.391                      H
HETATM   37  H              0       -3.034  -1.547  -3.145                      H
HETATM   38  H              0       -6.805   0.079  -0.436                      H
```

```
HETATM   39  H              0       -5.979   1.561   0.076                      H
HETATM   40  H              0       -6.573   0.426   1.301                      H
HETATM   41  H              0       -2.341  -2.096   3.165                      H
HETATM   42  H              0       -0.901  -1.231   2.555                      H
HETATM   43  H              0       -2.167  -0.340   3.402                      H
HETATM   44  H              0        2.202  -2.198   2.627                      H
HETATM   45  H              0        1.725  -0.488   2.737                      H
HETATM   46  H              0        3.288  -1.024   3.415                      H
HETATM   47  H              0        7.077   0.463   0.848                      H
HETATM   48  H              0        6.356   1.823  -0.030                      H
HETATM   49  H              0        7.040   0.462  -0.937                      H
HETATM   50  H              0        3.109  -0.931  -3.343                      H
HETATM   51  H              0        1.541  -0.569  -2.569                      H
HETATM   52  H              0        2.181  -2.222  -2.543                      H
HETATM   53  C              0        1.247   1.964  -0.583                      C
HETATM   54  H              0        1.607   2.068  -1.627                      H
HETATM   55  H              0        1.838   2.471   0.205                      H
HETATM   56  C              0       -1.683   2.537  -0.260                      C
HETATM   57  C              0       -0.518   3.281  -0.536                      C
HETATM   58  H              0       -2.322   2.256  -1.106                      H
HETATM   59  H              0       -2.141   2.680   0.729                      H
HETATM   60  H              0       -0.256   3.579  -1.561                      H
HETATM   61  F              0       -0.066   4.138   0.381                      F
END
```

a3NB.pdb

```
TITLE      a3NB.pdb
HETATM    1 Ru              0        0.046   0.971  -0.140                     Ru
HETATM    2  C              0       -0.857  -3.176   0.384                      C
HETATM    3  C              0        0.662  -3.243   0.189                      C
HETATM    4  H              0       -1.414  -3.759  -0.370                      H
HETATM    5  H              0        0.959  -3.816  -0.709                      H
HETATM    6  C              0       -0.032  -1.022   0.068                      C
HETATM    7  N              0        1.038  -1.834   0.040                      N
HETATM    8  N              0       -1.150  -1.747   0.234                      N
HETATM    9 Cl              0       -0.872   0.638  -2.394                     Cl
HETATM   10 Cl              0        0.967   1.411   2.028                     Cl
HETATM   11  C              0       -1.231   2.471  -0.056                      C
HETATM   12  H              0       -1.468   2.818   0.964                      H
HETATM   13  C              0        2.384  -1.350  -0.009                      C
HETATM   14  C              0        3.093  -1.170   1.194                      C
HETATM   15  C              0        2.958  -1.031  -1.255                      C
HETATM   16  C              0        4.374  -0.615   1.132                      C
HETATM   17  C              0        4.243  -0.478  -1.269                      C
HETATM   18  C              0        4.956  -0.248  -0.087                      C
HETATM   19  H              0        4.923  -0.448   2.067                      H
HETATM   20  H              0        4.698  -0.220  -2.235                      H
HETATM   21  C              0       -2.468  -1.195   0.271                      C
HETATM   22  C              0       -2.910  -0.549   1.442                      C
HETATM   23  C              0       -3.303  -1.313  -0.857                      C
HETATM   24  C              0       -4.181   0.034   1.441                      C
HETATM   25  C              0       -4.566  -0.717  -0.812                      C
HETATM   26  C              0       -5.014  -0.025   0.319                      C
HETATM   27  H              0       -4.531   0.542   2.350                      H
HETATM   28  H              0       -5.213  -0.786  -1.696                      H
HETATM   29  C              0        2.503  -1.570   2.511                      C
HETATM   30  H              0        2.945  -0.989   3.336                      H
HETATM   31  H              0        1.414  -1.395   2.544                      H
HETATM   32  H              0        2.685  -2.641   2.731                      H
HETATM   33  C              0        2.214  -1.274  -2.531                      C
HETATM   34  H              0        1.864  -2.320  -2.613                      H
```

```
HETATM   35  H              0        1.311  -0.639  -2.619                         H
HETATM   36  H              0        2.853  -1.070  -3.407                         H
HETATM   37  C              0        6.307   0.399  -0.119                         C
HETATM   38  H              0        6.232   1.485   0.081                         H
HETATM   39  H              0        6.983  -0.014   0.649                         H
HETATM   40  H              0        6.797   0.288  -1.101                         H
HETATM   41  C              0       -2.052  -0.487   2.669                         C
HETATM   42  H              0       -2.651  -0.223   3.556                         H
HETATM   43  H              0       -1.550  -1.449   2.877                         H
HETATM   44  H              0       -1.243   0.267   2.586                         H
HETATM   45  C              0       -2.873  -2.076  -2.073                         C
HETATM   46  H              0       -3.181  -3.139  -2.013                         H
HETATM   47  H              0       -3.334  -1.659  -2.983                         H
HETATM   48  H              0       -1.782  -2.039  -2.221                         H
HETATM   49  C              0       -6.353   0.648   0.321                         C
HETATM   50  H              0       -6.286   1.669  -0.100                         H
HETATM   51  H              0       -7.091   0.103  -0.293                         H
HETATM   52  H              0       -6.763   0.750   1.339                         H
HETATM   53  H              0        1.189  -3.686   1.054                         H
HETATM   54  H              0       -1.182  -3.527   1.382                         H
HETATM   55  C              0        0.036   3.199  -0.611                         C
HETATM   56  H              0       -0.287   3.674  -1.557                         H
HETATM   57  C              0        1.248   2.291  -0.988                         C
HETATM   58  H              0        2.143   2.564  -0.406                         H
HETATM   59  H              0        1.402   2.182  -2.073                         H
HETATM   60  H              0       -2.078   2.443  -0.758                         H
HETATM   61  F              0        0.436   4.170   0.271                         F
END
```

a3m.pdb

```
TITLE       a3m.pdb
HETATM    1 Ru              0       -0.239   0.952  -0.163                        Ru
HETATM    2 Cl              0       -0.674   0.713  -2.563                        Cl
HETATM    3 Cl              0        0.049   1.406   2.194                        Cl
HETATM    4  C              0        0.131  -1.030   0.029                         C
HETATM    5  N              0       -0.900  -1.897   0.087                         N
HETATM    6  C              0       -0.463  -3.276   0.300                         C
HETATM    7  C              0        1.052  -3.170   0.107                         C
HETATM    8  N              0        1.285  -1.720   0.092                         N
HETATM    9  C              0       -2.241  -1.410   0.169                         C
HETATM   10  C              0       -3.039  -1.382  -0.995                         C
HETATM   11  C              0       -4.296  -0.776  -0.921                         C
HETATM   12  C              0       -4.778  -0.223   0.273                         C
HETATM   13  C              0       -3.995  -0.331   1.427                         C
HETATM   14  C              0       -2.732  -0.934   1.403                         C
HETATM   15  C              0        2.589  -1.139   0.052                         C
HETATM   16  C              0        3.247  -0.864   1.264                         C
HETATM   17  C              0        4.513  -0.271   1.214                         C
HETATM   18  C              0        5.127   0.045  -0.003                         C
HETATM   19  C              0        4.456  -0.261  -1.194                         C
HETATM   20  C              0        3.189  -0.855  -1.189                         C
HETATM   21  C              0       -2.585  -2.033  -2.264                         C
HETATM   22  C              0       -6.098   0.485   0.307                         C
HETATM   23  C              0       -1.991  -1.159   2.687                         C
HETATM   24  C              0        2.589  -1.163   2.575                         C
HETATM   25  C              0        6.464   0.721  -0.032                         C
HETATM   26  C              0        2.475  -1.158  -2.470                         C
HETATM   27  H              0       -0.947  -3.955  -0.424                         H
HETATM   28  H              0       -0.744  -3.612   1.318                         H
HETATM   29  H              0        1.392  -3.617  -0.847                         H
HETATM   30  H              0        1.630  -3.647   0.918                         H
```

```
HETATM   31  H              0       -4.914  -0.730  -1.827                 H
HETATM   32  H              0       -4.376   0.058   2.380                 H
HETATM   33  H              0        5.031  -0.048   2.156                 H
HETATM   34  H              0        4.931  -0.034  -2.157                 H
HETATM   35  H              0       -2.889  -3.099  -2.286                 H
HETATM   36  H              0       -1.493  -1.988  -2.391                 H
HETATM   37  H              0       -3.034  -1.547  -3.145                 H
HETATM   38  H              0       -6.805   0.079  -0.436                 H
HETATM   39  H              0       -5.979   1.561   0.076                 H
HETATM   40  H              0       -6.573   0.426   1.301                 H
HETATM   41  H              0       -2.341  -2.096   3.165                 H
HETATM   42  H              0       -0.901  -1.231   2.555                 H
HETATM   43  H              0       -2.167  -0.340   3.402                 H
HETATM   44  H              0        2.202  -2.198   2.627                 H
HETATM   45  H              0        1.725  -0.488   2.737                 H
HETATM   46  H              0        3.288  -1.024   3.415                 H
HETATM   47  H              0        7.077   0.463   0.848                 H
HETATM   48  H              0        6.356   1.823  -0.030                 H
HETATM   49  H              0        7.040   0.462  -0.937                 H
HETATM   50  H              0        3.109  -0.931  -3.343                 H
HETATM   51  H              0        1.541  -0.569  -2.569                 H
HETATM   52  H              0        2.181  -2.222  -2.543                 H
HETATM   53  C              0        1.247   1.964  -0.583                 C
HETATM   54  H              0        1.607   2.068  -1.627                 H
HETATM   55  H              0        1.838   2.471   0.205                 H
HETATM   56  C              0       -1.683   2.537  -0.260                 C
HETATM   57  C              0       -0.518   3.281  -0.536                 C
HETATM   58  H              0       -2.322   2.256  -1.106                 H
HETATM   59  H              0       -2.141   2.680   0.729                 H
HETATM   60  H              0       -0.256   3.579  -1.561                 H
HETATM   61  F              0       -0.066   4.138   0.381                 F
END
```

a3n.pdb

```
TITLE      a3n.pdb
HETATM    1 Ru              0       -0.271   0.974   0.170                Ru
HETATM    2  C              0       -0.372  -3.236  -0.070                 C
HETATM    3  C              0        1.147  -3.094   0.027                 C
HETATM    4  H              0       -0.703  -3.623  -1.054                 H
HETATM    5  H              0        1.684  -3.569  -0.813                 H
HETATM    6  C              0        0.185  -0.959   0.068                 C
HETATM    7  N              0        1.351  -1.642   0.003                 N
HETATM    8  N              0       -0.831  -1.861   0.112                 N
HETATM    9 Cl              0       -0.701   1.339  -2.187                Cl
HETATM   10 Cl              0       -0.080   0.922   2.606                Cl
HETATM   11  C              0       -1.890   2.715   0.425                 C
HETATM   12  C              0        2.653  -1.073  -0.091                 C
HETATM   13  C              0        3.442  -0.934   1.067                 C
HETATM   14  C              0        3.131  -0.668  -1.353                 C
HETATM   15  C              0        4.717  -0.374   0.940                 C
HETATM   16  C              0        4.411  -0.108  -1.434                 C
HETATM   17  C              0        5.214   0.051  -0.299                 C
HETATM   18  H              0        5.335  -0.256   1.839                 H
HETATM   19  H              0        4.788   0.212  -2.414                 H
HETATM   20  C              0       -2.193  -1.444  -0.013                 C
HETATM   21  C              0       -2.970  -1.264   1.153                 C
HETATM   22  C              0       -2.745  -1.237  -1.294                 C
HETATM   23  C              0       -4.269  -0.763   1.012                 C
HETATM   24  C              0       -4.049  -0.738  -1.385                 C
HETATM   25  C              0       -4.813  -0.471  -0.244                 C
HETATM   26  H              0       -4.877  -0.611   1.914                 H
```

```
HETATM   27  H              0       -4.475  -0.553  -2.379                 H
HETATM   28  C              0        2.901  -1.313   2.410                 C
HETATM   29  H              0        3.636  -1.111   3.207                 H
HETATM   30  H              0        1.982  -0.740   2.644                 H
HETATM   31  H              0        2.636  -2.384   2.476                 H
HETATM   32  C              0        2.274  -0.812  -2.572                 C
HETATM   33  H              0        1.840  -1.826  -2.659                 H
HETATM   34  H              0        1.417  -0.108  -2.554                 H
HETATM   35  H              0        2.849  -0.612  -3.491                 H
HETATM   36  C              0        6.567   0.686  -0.401                 C
HETATM   37  H              0        6.512   1.777  -0.223                 H
HETATM   38  H              0        7.272   0.281   0.345                 H
HETATM   39  H              0        7.013   0.552  -1.401                 H
HETATM   40  C              0       -2.441  -1.649   2.499                 C
HETATM   41  H              0       -3.206  -1.504   3.279                 H
HETATM   42  H              0       -2.138  -2.712   2.524                 H
HETATM   43  H              0       -1.555  -1.051   2.780                 H
HETATM   44  C              0       -2.000  -1.609  -2.539                 C
HETATM   45  H              0       -2.227  -2.655  -2.828                 H
HETATM   46  H              0       -2.288  -0.964  -3.384                 H
HETATM   47  H              0       -0.908  -1.516  -2.429                 H
HETATM   48  C              0       -6.187   0.116  -0.366                 C
HETATM   49  H              0       -6.146   1.222  -0.405                 H
HETATM   50  H              0       -6.697  -0.212  -1.287                 H
HETATM   51  H              0       -6.827  -0.147   0.493                 H
HETATM   52  H              0        1.554  -3.523   0.963                 H
HETATM   53  H              0       -0.795  -3.898   0.706                 H
HETATM   54  C              0       -0.795   3.485   0.640                 C
HETATM   55  C              0        1.414   1.636   0.007                 C
HETATM   56  H              0        2.194   1.505   0.787                 H
HETATM   57  H              0        1.700   2.257  -0.871                 H
HETATM   58  H              0       -2.338   2.195   1.281                 H
HETATM   59  H              0       -0.253   3.559   1.591                 H
HETATM   60  H              0       -2.437   2.787  -0.521                 H
HETATM   61  F              0       -0.335   4.308  -0.291                 F
END
```

4a.pdb

```
TITLE      4a.pdb
HETATM    1 Ru              0       -0.298   0.542   0.034                Ru
HETATM    2  C              0        0.227  -3.577   0.133                 C
HETATM    3  C              0        1.710  -3.236  -0.020                 C
HETATM    4  H              0       -0.153  -4.220  -0.682                 H
HETATM    5  H              0        2.134  -3.579  -0.984                 H
HETATM    6  C              0        0.493  -1.241   0.055                 C
HETATM    7  N              0        1.736  -1.769   0.037                 N
HETATM    8  N              0       -0.403  -2.263   0.088                 N
HETATM    9 Cl              0       -0.725   0.715  -2.317                Cl
HETATM   10 Cl              0       -0.403   0.673   2.428                Cl
HETATM   11  C              0       -2.624   2.351   0.194                 C
HETATM   12  C              0        2.972  -1.064  -0.035                 C
HETATM   13  C              0        3.703  -0.858   1.150                 C
HETATM   14  C              0        3.455  -0.626  -1.280                 C
HETATM   15  C              0        4.942  -0.219   1.066                 C
HETATM   16  C              0        4.693   0.027  -1.318                 C
HETATM   17  C              0        5.449   0.233  -0.159                 C
HETATM   18  H              0        5.518  -0.051   1.985                 H
HETATM   19  H              0        5.079   0.375  -2.286                 H
HETATM   20  C              0       -1.807  -2.011   0.012                 C
HETATM   21  C              0       -2.558  -1.832   1.197                 C
HETATM   22  C              0       -2.426  -1.951  -1.256                 C
```

```
HETATM   23  C              0      -3.898  -1.448   1.078                      C
HETATM   24  C              0      -3.769  -1.571  -1.323                      C
HETATM   25  C              0      -4.508  -1.282  -0.170                      C
HETATM   26  H              0      -4.480  -1.278   1.993                      H
HETATM   27  H              0      -4.246  -1.488  -2.308                      H
HETATM   28  C              0       3.114  -1.237   2.473                      C
HETATM   29  H              0       3.820  -1.050   3.297                      H
HETATM   30  H              0       2.195  -0.652   2.673                      H
HETATM   31  H              0       2.820  -2.303   2.522                      H
HETATM   32  C              0       2.655  -0.829  -2.529                      C
HETATM   33  H              0       2.286  -1.867  -2.626                      H
HETATM   34  H              0       1.756  -0.182  -2.554                      H
HETATM   35  H              0       3.252  -0.600  -3.428                      H
HETATM   36  C              0       6.762   0.955  -0.221                      C
HETATM   37  H              0       6.636   2.035  -0.017                      H
HETATM   38  H              0       7.478   0.574   0.527                      H
HETATM   39  H              0       7.231   0.873  -1.216                      H
HETATM   40  C              0      -1.985  -2.142   2.545                      C
HETATM   41  H              0      -2.374  -1.457   3.315                      H
HETATM   42  H              0      -2.266  -3.171   2.845                      H
HETATM   43  H              0      -0.888  -2.064   2.571                      H
HETATM   44  C              0      -1.700  -2.353  -2.502                      C
HETATM   45  H              0      -1.872  -3.428  -2.717                      H
HETATM   46  H              0      -2.055  -1.781  -3.374                      H
HETATM   47  H              0      -0.613  -2.193  -2.434                      H
HETATM   48  C              0      -5.918  -0.788  -0.268                      C
HETATM   49  H              0      -5.944   0.317  -0.309                      H
HETATM   50  H              0      -6.422  -1.150  -1.181                      H
HETATM   51  H              0      -6.524  -1.086   0.604                      H
HETATM   52  H              0       2.339  -3.662   0.782                      H
HETATM   53  H              0      -0.002  -4.082   1.091                      H
HETATM   54  C              0      -1.568   3.180   0.083                      C
HETATM   55  C              0       1.238   1.524  -0.029                      C
HETATM   56  F              0      -3.327   1.952  -0.835                      F
HETATM   57  F              0      -3.141   1.999   1.342                      F
HETATM   58  F              0      -0.952   3.689   1.122                      F
HETATM   59  F              0      -1.178   3.685  -1.057                      F
HETATM   60  F              0       2.186   1.599   0.877                      F
HETATM   61  F              0       1.559   2.402  -0.959                      F
END
```

4b.pdb

```
TITLE      4b.pdb
HETATM    1 Ru              0      -0.144   0.637  -0.001                     Ru
HETATM    2  C              0      -0.430  -3.665   0.169                      C
HETATM    3  C              0       1.091  -3.544   0.011                      C
HETATM    4  H              0      -0.899  -4.292  -0.609                      H
HETATM    5  H              0       1.464  -3.996  -0.927                      H
HETATM    6  C              0       0.153  -1.416  -0.015                      C
HETATM    7  N              0       1.308  -2.091  -0.014                      N
HETATM    8  N              0      -0.875  -2.274   0.047                      N
HETATM    9 Cl              0      -0.999   0.478  -2.267                     Cl
HETATM   10 Cl              0       0.717   0.585   2.256                     Cl
HETATM   11  C              0      -1.617   2.029   0.447                      C
HETATM   12  C              0       2.593  -1.469  -0.072                      C
HETATM   13  C              0       3.370  -1.368   1.096                      C
HETATM   14  C              0       3.051  -0.958  -1.303                      C
HETATM   15  C              0       4.588  -0.683   1.024                      C
HETATM   16  C              0       4.272  -0.276  -1.327                      C
HETATM   17  C              0       5.044  -0.116  -0.170                      C
HETATM   18  H              0       5.189  -0.580   1.936                      H
```

```
HETATM   19  H              0       4.633   0.130  -2.281                   H
HETATM   20  C              0      -2.222  -1.798   0.108                   C
HETATM   21  C              0      -2.707  -1.224   1.301                   C
HETATM   22  C              0      -3.013  -1.840  -1.059                   C
HETATM   23  C              0      -3.961  -0.594   1.267                   C
HETATM   24  C              0      -4.261  -1.219  -1.036                   C
HETATM   25  C              0      -4.734  -0.558   0.107                   C
HETATM   26  H              0      -4.330  -0.115   2.184                   H
HETATM   27  H              0      -4.865  -1.213  -1.952                   H
HETATM   28  C              0       2.935  -1.993   2.387                   C
HETATM   29  H              0       3.297  -1.410   3.250                   H
HETATM   30  H              0       1.838  -2.045   2.475                   H
HETATM   31  H              0       3.342  -3.018   2.495                   H
HETATM   32  C              0       2.259  -1.148  -2.561                   C
HETATM   33  H              0       1.943  -2.199  -2.693                   H
HETATM   34  H              0       1.328  -0.548  -2.580                   H
HETATM   35  H              0       2.851  -0.864  -3.446                   H
HETATM   36  C              0       6.322   0.666  -0.207                   C
HETATM   37  H              0       6.816   0.602  -1.191                   H
HETATM   38  H              0       6.138   1.740  -0.014                   H
HETATM   39  H              0       7.038   0.325   0.560                   H
HETATM   40  C              0      -1.989  -1.331   2.612                   C
HETATM   41  H              0      -2.604  -1.909   3.328                   H
HETATM   42  H              0      -1.005  -1.819   2.537                   H
HETATM   43  H              0      -1.809  -0.340   3.062                   H
HETATM   44  C              0      -2.520  -2.524  -2.296                   C
HETATM   45  H              0      -2.662  -3.622  -2.240                   H
HETATM   46  H              0      -3.061  -2.172  -3.188                   H
HETATM   47  H              0      -1.448  -2.326  -2.467                   H
HETATM   48  C              0      -6.020   0.206   0.066                   C
HETATM   49  H              0      -5.847   1.231  -0.318                   H
HETATM   50  H              0      -6.761  -0.260  -0.604                   H
HETATM   51  H              0      -6.474   0.312   1.066                   H
HETATM   52  H              0       1.652  -4.000   0.846                   H
HETATM   53  H              0      -0.732  -4.069   1.154                   H
HETATM   54  C              0      -0.506   2.880   0.087                   C
HETATM   55  C              0       1.228   1.806  -0.530                   C
HETATM   56  F              0      -2.692   2.056  -0.335                   F
HETATM   57  F              0      -2.002   2.022   1.726                   F
HETATM   58  F              0      -0.632   3.594  -1.027                   F
HETATM   59  F              0       0.066   3.593   1.050                   F
HETATM   60  F              0       1.464   2.212  -1.752                   F
HETATM   61  F              0       2.188   2.273   0.223                   F
END
```

4NA.pdb

```
TITLE       4NA.pdb
HETATM    1 Ru              0       0.017   0.582   0.002                  Ru
HETATM    2  C              0      -0.818  -3.603   0.081                   C
HETATM    3  C              0       0.708  -3.626  -0.077                   C
HETATM    4  H              0      -1.347  -4.108  -0.747                   H
HETATM    5  H              0       1.041  -4.092  -1.023                   H
HETATM    6  C              0      -0.023  -1.430   0.003                   C
HETATM    7  N              0       1.065  -2.200  -0.069                   N
HETATM    8  N              0      -1.133  -2.168   0.075                   N
HETATM    9 Cl              0      -0.797   0.498  -2.270                  Cl
HETATM   10 Cl              0       0.819   0.481   2.279                  Cl
HETATM   11  C              0      -1.244   2.037   0.420                   C
HETATM   12  C              0       2.393  -1.666  -0.108                   C
HETATM   13  C              0       3.156  -1.628   1.077                   C
HETATM   14  C              0       2.896  -1.157  -1.321                   C
```

```
HETATM   15  C              0       4.393  -0.984   1.045                       C
HETATM   16  C              0       4.143  -0.518  -1.303                       C
HETATM   17  C              0       4.889  -0.400  -0.128                       C
HETATM   18  H              0       4.980  -0.922   1.971                       H
HETATM   19  H              0       4.537  -0.106  -2.240                       H
HETATM   20  C              0      -2.450  -1.606   0.107                       C
HETATM   21  C              0      -2.951  -1.091   1.319                       C
HETATM   22  C              0      -3.211  -1.562  -1.079                       C
HETATM   23  C              0      -4.195  -0.449   1.301                       C
HETATM   24  C              0      -4.446  -0.913  -1.047                       C
HETATM   25  C              0      -4.943  -0.331   0.126                       C
HETATM   26  H              0      -4.589  -0.037   2.239                       H
HETATM   27  H              0      -5.033  -0.851  -1.972                       H
HETATM   28  C              0       2.679  -2.282   2.338                       C
HETATM   29  H              0       3.096  -1.781   3.226                       H
HETATM   30  H              0       1.582  -2.251   2.437                       H
HETATM   31  H              0       2.999  -3.343   2.383                       H
HETATM   32  C              0       2.151  -1.291  -2.613                       C
HETATM   33  H              0       1.472  -2.160  -2.622                       H
HETATM   34  H              0       1.512  -0.412  -2.822                       H
HETATM   35  H              0       2.854  -1.396  -3.457                       H
HETATM   36  C              0       6.182   0.356  -0.113                       C
HETATM   37  H              0       6.021   1.400   0.219                       H
HETATM   38  H              0       6.914  -0.085   0.584                       H
HETATM   39  H              0       6.644   0.407  -1.113                       H
HETATM   40  C              0      -2.202  -1.228   2.609                       C
HETATM   41  H              0      -2.901  -1.272   3.462                       H
HETATM   42  H              0      -1.570  -2.132   2.637                       H
HETATM   43  H              0      -1.515  -0.380   2.789                       H
HETATM   44  C              0      -2.737  -2.218  -2.339                       C
HETATM   45  H              0      -3.076  -3.272  -2.394                       H
HETATM   46  H              0      -3.139  -1.704  -3.227                       H
HETATM   47  H              0      -1.639  -2.205  -2.432                       H
HETATM   48  C              0      -6.239   0.419   0.115                       C
HETATM   49  H              0      -6.075   1.482  -0.144                       H
HETATM   50  H              0      -6.945   0.017  -0.631                       H
HETATM   51  H              0      -6.733   0.405   1.101                       H
HETATM   52  H              0       1.222  -4.146   0.751                       H
HETATM   53  H              0      -1.164  -4.060   1.026                       H
HETATM   54  C              0       0.060   2.876  -0.003                       C
HETATM   55  C              0       1.331   1.986  -0.425                       C
HETATM   56  F              0      -2.286   2.310  -0.356                       F
HETATM   57  F              0      -1.612   2.293   1.674                       F
HETATM   58  F              0      -0.269   3.659  -1.044                       F
HETATM   59  F              0       0.421   3.648   1.035                       F
HETATM   60  F              0       1.707   2.220  -1.681                       F
HETATM   61  F              0       2.384   2.226   0.349                       F
END
```

s5a.pdb

```
TITLE      s5a.pdb
HETATM    1 Ru              0      -0.232   0.443  -0.028                      Ru
HETATM    2  C              0       0.506  -3.629   0.239                       C
HETATM    3  C              0       1.976  -3.214   0.130                       C
HETATM    4  H              0       0.192  -4.312  -0.572                       H
HETATM    5  H              0       2.459  -3.579  -0.796                       H
HETATM    6  C              0       0.653  -1.288   0.086                       C
HETATM    7  N              0       1.921  -1.747   0.115                       N
HETATM    8  N              0      -0.190  -2.353   0.139                       N
HETATM    9 Cl              0      -0.596   0.497  -2.394                      Cl
HETATM   10 Cl              0      -0.375   0.687   2.355                      Cl
```

```
HETATM   11  C             0      -2.698   2.255  -0.102                C
HETATM   12  C             0       3.117  -0.974   0.046                C
HETATM   13  C             0       3.794  -0.663   1.239                C
HETATM   14  C             0       3.613  -0.571  -1.206                C
HETATM   15  C             0       4.993   0.049   1.159                C
HETATM   16  C             0       4.808   0.160  -1.241                C
HETATM   17  C             0       5.510   0.472  -0.072                C
HETATM   18  H             0       5.526   0.298   2.085                H
HETATM   19  H             0       5.202   0.483  -2.213                H
HETATM   20  C             0      -1.602  -2.164   0.033                C
HETATM   21  C             0      -2.376  -1.957   1.197                C
HETATM   22  C             0      -2.203  -2.178  -1.247                C
HETATM   23  C             0      -3.725  -1.621   1.042                C
HETATM   24  C             0      -3.554  -1.841  -1.349                C
HETATM   25  C             0      -4.320  -1.526  -0.221                C
HETATM   26  H             0      -4.325  -1.423   1.939                H
HETATM   27  H             0      -4.018  -1.813  -2.344                H
HETATM   28  C             0       3.183  -1.007   2.561                C
HETATM   29  H             0       3.847  -0.732   3.397                H
HETATM   30  H             0       2.223  -0.469   2.698                H
HETATM   31  H             0       2.952  -2.084   2.662                H
HETATM   32  C             0       2.871  -0.895  -2.466                C
HETATM   33  H             0       2.607  -1.967  -2.529                H
HETATM   34  H             0       1.916  -0.339  -2.541                H
HETATM   35  H             0       3.470  -0.646  -3.357                H
HETATM   36  C             0       6.775   1.274  -0.131                C
HETATM   37  H             0       6.579   2.348   0.051                H
HETATM   38  H             0       7.502   0.955   0.636                H
HETATM   39  H             0       7.265   1.202  -1.117                H
HETATM   40  C             0      -1.818  -2.191   2.566                C
HETATM   41  H             0      -2.235  -1.483   3.299                H
HETATM   42  H             0      -2.080  -3.213   2.907                H
HETATM   43  H             0      -0.723  -2.088   2.607                H
HETATM   44  C             0      -1.440  -2.612  -2.460                C
HETATM   45  H             0      -1.515  -3.711  -2.589                H
HETATM   46  H             0      -1.840  -2.144  -3.373                H
HETATM   47  H             0      -0.371  -2.350  -2.406                H
HETATM   48  C             0      -5.740  -1.075  -0.363                C
HETATM   49  H             0      -5.791   0.022  -0.497                H
HETATM   50  H             0      -6.232  -1.523  -1.243                H
HETATM   51  H             0      -6.343  -1.314   0.530                H
HETATM   52  H             0       2.590  -3.565   0.979                H
HETATM   53  H             0       0.270  -4.124   1.201                H
HETATM   54  C             0      -1.642   3.090  -0.209                C
HETATM   55  C             0       1.246   1.512  -0.095                C
HETATM   56  F             0      -3.296   1.784  -1.182                F
HETATM   57  F             0      -1.085   3.675   0.819                F
HETATM   58  F             0      -1.162   3.499  -1.351                F
HETATM   59  F             0       2.161   1.685   0.831                F
HETATM   60  F             0       1.535   2.373  -1.052                F
HETATM   61 Cl             0      -3.497   1.946   1.372               Cl
END
```

s5b.pdb

```
TITLE       s5b.pdb
HETATM    1 Ru             0      -0.232   0.443  -0.028               Ru
HETATM    2  C             0       0.506  -3.629   0.239                C
HETATM    3  C             0       1.976  -3.214   0.130                C
HETATM    4  H             0       0.192  -4.312  -0.572                H
HETATM    5  H             0       2.459  -3.579  -0.796                H
HETATM    6  C             0       0.653  -1.288   0.086                C
```

```
HETATM    7  N             0        1.921  -1.747   0.115                       N
HETATM    8  N             0       -0.190  -2.353   0.139                       N
HETATM    9 Cl             0       -0.596   0.497  -2.394                      Cl
HETATM   10 Cl             0       -0.375   0.687   2.355                      Cl
HETATM   11  C             0       -2.698   2.255  -0.102                       C
HETATM   12  C             0        3.117  -0.974   0.046                       C
HETATM   13  C             0        3.794  -0.663   1.239                       C
HETATM   14  C             0        3.613  -0.571  -1.206                       C
HETATM   15  C             0        4.993   0.049   1.159                       C
HETATM   16  C             0        4.808   0.160  -1.241                       C
HETATM   17  C             0        5.510   0.472  -0.072                       C
HETATM   18  H             0        5.526   0.298   2.085                       H
HETATM   19  H             0        5.202   0.483  -2.213                       H
HETATM   20  C             0       -1.602  -2.164   0.033                       C
HETATM   21  C             0       -2.376  -1.957   1.197                       C
HETATM   22  C             0       -2.203  -2.178  -1.247                       C
HETATM   23  C             0       -3.725  -1.621   1.042                       C
HETATM   24  C             0       -3.554  -1.841  -1.349                       C
HETATM   25  C             0       -4.320  -1.526  -0.221                       C
HETATM   26  H             0       -4.325  -1.423   1.939                       H
HETATM   27  H             0       -4.018  -1.813  -2.344                       H
HETATM   28  C             0        3.183  -1.007   2.561                       C
HETATM   29  H             0        3.847  -0.732   3.397                       H
HETATM   30  H             0        2.223  -0.469   2.698                       H
HETATM   31  H             0        2.952  -2.084   2.662                       H
HETATM   32  C             0        2.871  -0.895  -2.466                       C
HETATM   33  H             0        2.607  -1.967  -2.529                       H
HETATM   34  H             0        1.916  -0.339  -2.541                       H
HETATM   35  H             0        3.470  -0.646  -3.357                       H
HETATM   36  C             0        6.775   1.274  -0.131                       C
HETATM   37  H             0        6.579   2.348   0.051                       H
HETATM   38  H             0        7.502   0.955   0.636                       H
HETATM   39  H             0        7.265   1.202  -1.117                       H
HETATM   40  C             0       -1.818  -2.191   2.566                       C
HETATM   41  H             0       -2.235  -1.483   3.299                       H
HETATM   42  H             0       -2.080  -3.213   2.907                       H
HETATM   43  H             0       -0.723  -2.088   2.607                       H
HETATM   44  C             0       -1.440  -2.612  -2.460                       C
HETATM   45  H             0       -1.515  -3.711  -2.589                       H
HETATM   46  H             0       -1.840  -2.144  -3.373                       H
HETATM   47  H             0       -0.371  -2.350  -2.406                       H
HETATM   48  C             0       -5.740  -1.075  -0.363                       C
HETATM   49  H             0       -5.791   0.022  -0.497                       H
HETATM   50  H             0       -6.232  -1.523  -1.243                       H
HETATM   51  H             0       -6.343  -1.314   0.530                       H
HETATM   52  H             0        2.590  -3.565   0.979                       H
HETATM   53  H             0        0.270  -4.124   1.201                       H
HETATM   54  C             0       -1.642   3.090  -0.209                       C
HETATM   55  C             0        1.246   1.512  -0.095                       C
HETATM   56  F             0       -3.296   1.784  -1.182                       F
HETATM   57  F             0       -1.085   3.675   0.819                       F
HETATM   58  F             0       -1.162   3.499  -1.351                       F
HETATM   59  F             0        2.161   1.685   0.831                       F
HETATM   60  F             0        1.535   2.373  -1.052                       F
HETATM   61 Cl             0       -3.497   1.946   1.372                      Cl
END
```

s5PA.pdb

```
TITLE       s5PA.pdb
HETATM    1 Ru             0        0.019   0.558  -0.081                      Ru
HETATM    2  C             0       -0.479  -3.681   0.195                       C
```

```
HETATM    3  C              0       1.042  -3.593   0.021                     C
HETATM    4  H              0      -0.974  -4.271  -0.595                     H
HETATM    5  H              0       1.397  -4.047  -0.923                     H
HETATM    6  C              0       0.148  -1.456   0.021                     C
HETATM    7  N              0       1.292  -2.146  -0.000                     N
HETATM    8  N              0      -0.903  -2.276   0.109                     N
HETATM    9 Cl              0      -0.755   0.191  -2.347                    Cl
HETATM   10 Cl              0       0.932   0.671   2.148                    Cl
HETATM   11  C              0      -1.414   1.892   0.203                     C
HETATM   12  C              0       2.593  -1.549  -0.015                     C
HETATM   13  C              0       3.325  -1.476   1.189                     C
HETATM   14  C              0       3.114  -1.045  -1.221                     C
HETATM   15  C              0       4.557  -0.821   1.174                     C
HETATM   16  C              0       4.353  -0.390  -1.184                     C
HETATM   17  C              0       5.076  -0.255   0.003                     C
HETATM   18  H              0       5.121  -0.738   2.112                     H
HETATM   19  H              0       4.765   0.012  -2.119                     H
HETATM   20  C              0      -2.260  -1.818   0.113                     C
HETATM   21  C              0      -2.795  -1.275   1.299                     C
HETATM   22  C              0      -3.023  -1.898  -1.067                     C
HETATM   23  C              0      -4.077  -0.720   1.252                     C
HETATM   24  C              0      -4.299  -1.329  -1.066                     C
HETATM   25  C              0      -4.829  -0.712   0.073                     C
HETATM   26  H              0      -4.492  -0.274   2.165                     H
HETATM   27  H              0      -4.888  -1.353  -1.992                     H
HETATM   28  C              0       2.819  -2.098   2.454                     C
HETATM   29  H              0       3.187  -1.552   3.338                     H
HETATM   30  H              0       1.719  -2.098   2.513                     H
HETATM   31  H              0       3.167  -3.146   2.555                     H
HETATM   32  C              0       2.401  -1.204  -2.529                     C
HETATM   33  H              0       1.711  -2.065  -2.538                     H
HETATM   34  H              0       1.777  -0.326  -2.776                     H
HETATM   35  H              0       3.125  -1.334  -3.351                     H
HETATM   36  C              0       6.375   0.491   0.033                     C
HETATM   37  H              0       6.234   1.517   0.421                     H
HETATM   38  H              0       7.114   0.006   0.694                     H
HETATM   39  H              0       6.821   0.584  -0.971                     H
HETATM   40  C              0      -2.029  -1.308   2.587                     C
HETATM   41  H              0      -2.674  -1.017   3.432                     H
HETATM   42  H              0      -1.631  -2.317   2.804                     H
HETATM   43  H              0      -1.160  -0.622   2.592                     H
HETATM   44  C              0      -2.515  -2.598  -2.290                     C
HETATM   45  H              0      -2.844  -3.656  -2.307                     H
HETATM   46  H              0      -2.903  -2.125  -3.207                     H
HETATM   47  H              0      -1.416  -2.578  -2.362                     H
HETATM   48  C              0      -6.163  -0.033   0.024                     C
HETATM   49  H              0      -6.046   1.043  -0.209                     H
HETATM   50  H              0      -6.818  -0.457  -0.755                     H
HETATM   51  H              0      -6.693  -0.090   0.990                     H
HETATM   52  H              0       1.601  -4.061   0.850                     H
HETATM   53  H              0      -0.782  -4.106   1.170                     H
HETATM   54  C              0      -0.213   2.831  -0.249                     C
HETATM   55  C              0       1.159   2.042  -0.662                     C
HETATM   56  F              0      -2.435   1.982  -0.651                     F
HETATM   57  F              0      -0.610   3.508  -1.338                     F
HETATM   58  F              0       0.109   3.705   0.715                     F
HETATM   59  F              0       1.464   2.250  -1.940                     F
HETATM   60  F              0       2.193   2.461   0.054                     F
HETATM   61 Cl              0      -2.038   2.216   1.820                    Cl
END
```

s5c.pdb

```
TITLE      s5c.pdb
HETATM    1 Ru             0        0.136   0.620  -0.075                 Ru
HETATM    2 Cl             0       -0.541   0.212  -2.372                 Cl
HETATM    3 Cl             0        0.948   0.759   2.194                 Cl
HETATM    4  C             0       -0.013  -1.450   0.107                  C
HETATM    5  N             0       -1.128  -2.192   0.122                  N
HETATM    6  C             0       -0.830  -3.626   0.236                  C
HETATM    7  C             0        0.696  -3.670   0.116                  C
HETATM    8  N             0        1.061  -2.250   0.151                  N
HETATM    9  C             0       -2.449  -1.650   0.101                  C
HETATM   10  C             0       -3.207  -1.703  -1.083                  C
HETATM   11  C             0       -4.466  -1.093  -1.090                  C
HETATM   12  C             0       -4.982  -0.455   0.042                  C
HETATM   13  C             0       -4.226  -0.465   1.221                  C
HETATM   14  C             0       -2.964  -1.066   1.276                  C
HETATM   15  C             0        2.393  -1.733   0.092                  C
HETATM   16  C             0        3.121  -1.582   1.290                  C
HETATM   17  C             0        4.363  -0.948   1.233                  C
HETATM   18  C             0        4.894  -0.475   0.026                  C
HETATM   19  C             0        4.185  -0.707  -1.155                  C
HETATM   20  C             0        2.940  -1.349  -1.150                  C
HETATM   21  C             0       -2.711  -2.415  -2.304                  C
HETATM   22  C             0       -6.306   0.244  -0.009                  C
HETATM   23  C             0       -2.191  -1.101   2.559                  C
HETATM   24  C             0        2.596  -2.097   2.595                  C
HETATM   25  C             0        6.179   0.294   0.009                  C
HETATM   26  C             0        2.285  -1.688  -2.456                  C
HETATM   27  H             0       -1.347  -4.188  -0.561                  H
HETATM   28  H             0       -1.198  -4.010   1.207                  H
HETATM   29  H             0        1.043  -4.122  -0.834                  H
HETATM   30  H             0        1.184  -4.215   0.943                  H
HETATM   31  H             0       -5.054  -1.110  -2.017                  H
HETATM   32  H             0       -4.625   0.006   2.128                  H
HETATM   33  H             0        4.920  -0.791   2.166                  H
HETATM   34  H             0        4.605  -0.379  -2.115                  H
HETATM   35  H             0       -3.090  -1.939  -3.223                  H
HETATM   36  H             0       -1.611  -2.409  -2.371                  H
HETATM   37  H             0       -3.055  -3.469  -2.320                  H
HETATM   38  H             0       -6.181   1.317  -0.250                  H
HETATM   39  H             0       -6.968  -0.180  -0.783                  H
HETATM   40  H             0       -6.836   0.197   0.958                  H
HETATM   41  H             0       -1.254  -0.510   2.520                  H
HETATM   42  H             0       -1.894  -2.132   2.831                  H
HETATM   43  H             0       -2.792  -0.699   3.391                  H
HETATM   44  H             0        1.495  -2.077   2.641                  H
HETATM   45  H             0        2.963  -1.490   3.438                  H
HETATM   46  H             0        2.926  -3.140   2.773                  H
HETATM   47  H             0        5.993   1.370   0.193                  H
HETATM   48  H             0        6.875  -0.044   0.796                  H
HETATM   49  H             0        6.694   0.221  -0.964                  H
HETATM   50  H             0        2.322  -0.843  -3.163                  H
HETATM   51  H             0        2.817  -2.533  -2.936                  H
HETATM   52  H             0        1.224  -1.964  -2.358                  H
HETATM   53  C             0        1.502   2.036  -0.752                  C
HETATM   54  F             0        1.842   2.004  -2.038                  F
HETATM   55  F             0        2.606   2.104  -0.008                  F
HETATM   56  C             0        0.395   2.880  -0.372                  C
HETATM   57  C             0       -1.369   1.711   0.178                  C
HETATM   58  F             0       -0.240   3.520  -1.345                  F
HETATM   59  F             0        0.534   3.645   0.697                  F
HETATM   60  F             0       -2.238   2.039  -0.742                  F
HETATM   61 Cl             0       -1.950   2.310   1.705                 Cl
```

```
END
```

s5d.pdb

```
TITLE      s5d.pdb
HETATM    1 Ru            0       -0.267   0.513   0.097                    Ru
HETATM    2  C            0        0.099  -3.633   0.019                     C
HETATM    3  C            0        1.589  -3.340  -0.133                     C
HETATM    4  H            0       -0.308  -4.245  -0.806                     H
HETATM    5  H            0        1.987  -3.634  -1.124                     H
HETATM    6  C            0        0.445  -1.307   0.037                     C
HETATM    7  N            0        1.669  -1.882   0.018                     N
HETATM    8  N            0       -0.486  -2.298   0.008                     N
HETATM    9 Cl            0       -0.774   0.760  -2.221                    Cl
HETATM   10 Cl            0       -0.398   0.522   2.484                    Cl
HETATM   11  C            0       -2.699   2.277   0.414                     C
HETATM   12  C            0        2.934  -1.229  -0.046                     C
HETATM   13  C            0        3.706  -1.140   1.128                     C
HETATM   14  C            0        3.400  -0.718  -1.269                     C
HETATM   15  C            0        4.962  -0.535   1.056                     C
HETATM   16  C            0        4.656  -0.099  -1.293                     C
HETATM   17  C            0        5.449  -0.003  -0.146                     C
HETATM   18  H            0        5.568  -0.457   1.968                     H
HETATM   19  H            0        5.025   0.311  -2.243                     H
HETATM   20  C            0       -1.887  -2.030  -0.081                     C
HETATM   21  C            0       -2.658  -1.891   1.095                     C
HETATM   22  C            0       -2.488  -1.946  -1.357                     C
HETATM   23  C            0       -4.002  -1.523   0.965                     C
HETATM   24  C            0       -3.836  -1.591  -1.434                     C
HETATM   25  C            0       -4.598  -1.342  -0.287                     C
HETATM   26  H            0       -4.601  -1.385   1.876                     H
HETATM   27  H            0       -4.300  -1.493  -2.424                     H
HETATM   28  C            0        3.140  -1.599   2.436                     C
HETATM   29  H            0        3.867  -1.471   3.256                     H
HETATM   30  H            0        2.232  -1.021   2.696                     H
HETATM   31  H            0        2.839  -2.663   2.424                     H
HETATM   32  C            0        2.565  -0.800  -2.510                     C
HETATM   33  H            0        2.108  -1.798  -2.643                     H
HETATM   34  H            0        1.722  -0.081  -2.492                     H
HETATM   35  H            0        3.165  -0.582  -3.409                     H
HETATM   36  C            0        6.782   0.682  -0.188                     C
HETATM   37  H            0        6.720   1.705   0.229                     H
HETATM   38  H            0        7.539   0.146   0.411                     H
HETATM   39  H            0        7.165   0.777  -1.217                     H
HETATM   40  C            0       -2.109  -2.232   2.446                     C
HETATM   41  H            0       -2.498  -1.553   3.222                     H
HETATM   42  H            0       -2.414  -3.261   2.726                     H
HETATM   43  H            0       -1.011  -2.174   2.493                     H
HETATM   44  C            0       -1.739  -2.300  -2.604                     C
HETATM   45  H            0       -1.903  -3.366  -2.862                     H
HETATM   46  H            0       -2.085  -1.699  -3.460                     H
HETATM   47  H            0       -0.654  -2.138  -2.515                     H
HETATM   48  C            0       -6.015  -0.870  -0.399                     C
HETATM   49  H            0       -6.054   0.233  -0.476                     H
HETATM   50  H            0       -6.513  -1.266  -1.300                     H
HETATM   51  H            0       -6.617  -1.150   0.481                     H
HETATM   52  H            0        2.212  -3.841   0.629                     H
HETATM   53  H            0       -0.144  -4.150   0.969                     H
HETATM   54  C            0       -1.707   3.164   0.220                     C
HETATM   55  C            0        1.282   1.450   0.128                     C
HETATM   56  F            0       -3.431   1.815  -0.568                     F
HETATM   57  F            0       -3.114   1.905   1.597                     F
```

```
HETATM   58  F             0       -1.033   3.707   1.204                        F
HETATM   59  F             0       -1.440   3.675  -0.951                        F
HETATM   60  F             0        2.284   1.330   0.971                        F
HETATM   61 Cl             0        1.607   2.859  -0.869                       Cl
END
```

a5a.pdb

```
TITLE       a5a.pdb
HETATM    1 Ru             0       -0.282   0.419  -0.032                       Ru
HETATM    2  C             0        0.316  -3.675   0.338                        C
HETATM    3  C             0        1.792  -3.314   0.154                        C
HETATM    4  H             0       -0.055  -4.374  -0.434                        H
HETATM    5  H             0        2.220  -3.715  -0.785                        H
HETATM    6  C             0        0.533  -1.344   0.110                        C
HETATM    7  N             0        1.787  -1.848   0.111                        N
HETATM    8  N             0       -0.341  -2.380   0.220                        N
HETATM    9 Cl             0       -0.696   0.433  -2.388                       Cl
HETATM   10 Cl             0       -0.432   0.691   2.346                       Cl
HETATM   11  C             0       -2.657   2.264  -0.029                        C
HETATM   12  C             0        3.008  -1.122   0.007                        C
HETATM   13  C             0        3.718  -0.822   1.183                        C
HETATM   14  C             0        3.499  -0.757  -1.259                        C
HETATM   15  C             0        4.944  -0.162   1.073                        C
HETATM   16  C             0        4.723  -0.079  -1.325                        C
HETATM   17  C             0        5.458   0.220  -0.173                        C
HETATM   18  H             0        5.503   0.079   1.986                        H
HETATM   19  H             0        5.113   0.215  -2.308                        H
HETATM   20  C             0       -1.750  -2.162   0.131                        C
HETATM   21  C             0       -2.505  -1.926   1.302                        C
HETATM   22  C             0       -2.370  -2.188  -1.139                        C
HETATM   23  C             0       -3.851  -1.571   1.162                        C
HETATM   24  C             0       -3.718  -1.833  -1.228                        C
HETATM   25  C             0       -4.462  -1.489  -0.094                        C
HETATM   26  H             0       -4.436  -1.354   2.065                        H
HETATM   27  H             0       -4.196  -1.817  -2.215                        H
HETATM   28  C             0        3.116  -1.124   2.520                        C
HETATM   29  H             0        3.798  -0.848   3.341                        H
HETATM   30  H             0        2.172  -0.559   2.658                        H
HETATM   31  H             0        2.860  -2.193   2.646                        H
HETATM   32  C             0        2.721  -1.063  -2.502                        C
HETATM   33  H             0        2.408  -2.123  -2.547                        H
HETATM   34  H             0        1.789  -0.467  -2.565                        H
HETATM   35  H             0        3.313  -0.850  -3.407                        H
HETATM   36  C             0        6.754   0.967  -0.264                        C
HETATM   37  H             0        6.607   2.050  -0.087                        H
HETATM   38  H             0        7.483   0.623   0.489                        H
HETATM   39  H             0        7.219   0.868  -1.260                        H
HETATM   40  C             0       -1.929  -2.149   2.666                        C
HETATM   41  H             0       -2.326  -1.427   3.396                        H
HETATM   42  H             0       -2.197  -3.163   3.025                        H
HETATM   43  H             0       -0.833  -2.057   2.688                        H
HETATM   44  C             0       -1.636  -2.659  -2.356                        C
HETATM   45  H             0       -1.775  -3.751  -2.487                        H
HETATM   46  H             0       -2.012  -2.167  -3.267                        H
HETATM   47  H             0       -0.553  -2.462  -2.307                        H
HETATM   48  C             0       -5.881  -1.027  -0.222                        C
HETATM   49  H             0       -5.927   0.074  -0.330                        H
HETATM   50  H             0       -6.378  -1.452  -1.110                        H
HETATM   51  H             0       -6.483  -1.282   0.667                        H
HETATM   52  H             0        2.432  -3.671   0.981                        H
HETATM   53  H             0        0.104  -4.127   1.326                        H
```

```
HETATM   54  C              0       -1.623   3.103  -0.254                      C
HETATM   55  C              0        1.228   1.435  -0.134                      C
HETATM   56  F              0       -3.365   1.750  -1.001                      F
HETATM   57  F              0       -3.142   1.993   1.152                      F
HETATM   58  F              0       -1.312   3.443  -1.490                      F
HETATM   59  F              0        2.172   1.575   0.769                      F
HETATM   60  F              0        1.508   2.299  -1.089                      F
HETATM   61 Cl              0       -0.807   3.959   0.975                     Cl
END
```

a5b.pdb

```
TITLE       a5b.pdb
HETATM    1 Ru              0       -0.106   0.553  -0.060                     Ru
HETATM    2 Cl              0       -0.988   0.141  -2.292                     Cl
HETATM    3 Cl              0        0.790   0.661   2.169                     Cl
HETATM    4  C              0        0.122  -1.502   0.082                      C
HETATM    5  N              0       -0.915  -2.337   0.221                      N
HETATM    6  C              0       -0.485  -3.724   0.422                      C
HETATM    7  C              0        1.031  -3.638   0.216                      C
HETATM    8  N              0        1.269  -2.192   0.097                      N
HETATM    9  C              0       -2.264  -1.862   0.243                      C
HETATM   10  C              0       -3.059  -2.019  -0.911                      C
HETATM   11  C              0       -4.310  -1.405  -0.941                      C
HETATM   12  C              0       -4.782  -0.643   0.138                      C
HETATM   13  C              0       -4.006  -0.569   1.294                      C
HETATM   14  C              0       -2.749  -1.186   1.380                      C
HETATM   15  C              0        2.567  -1.600   0.002                      C
HETATM   16  C              0        3.342  -1.445   1.166                      C
HETATM   17  C              0        4.578  -0.800   1.057                      C
HETATM   18  C              0        5.056  -0.325  -0.169                      C
HETATM   19  C              0        4.285  -0.540  -1.317                      C
HETATM   20  C              0        3.044  -1.184  -1.257                      C
HETATM   21  C              0       -2.577  -2.820  -2.081                      C
HETATM   22  C              0       -6.072   0.109   0.029                      C
HETATM   23  C              0       -2.015  -1.161   2.687                      C
HETATM   24  C              0        2.889  -1.974   2.492                      C
HETATM   25  C              0        6.357   0.413  -0.248                      C
HETATM   26  C              0        2.257  -1.436  -2.506                      C
HETATM   27  H              0       -0.984  -4.394  -0.300                      H
HETATM   28  H              0       -0.765  -4.058   1.440                      H
HETATM   29  H              0        1.373  -4.152  -0.701                      H
HETATM   30  H              0        1.609  -4.050   1.063                      H
HETATM   31  H              0       -4.918  -1.487  -1.850                      H
HETATM   32  H              0       -4.374  -0.008   2.163                      H
HETATM   33  H              0        5.179  -0.653   1.964                      H
HETATM   34  H              0        4.663  -0.209  -2.294                      H
HETATM   35  H              0       -3.067  -2.494  -3.013                      H
HETATM   36  H              0       -1.490  -2.714  -2.233                      H
HETATM   37  H              0       -2.802  -3.898  -1.955                      H
HETATM   38  H              0       -5.902   1.101  -0.432                      H
HETATM   39  H              0       -6.533   0.290   1.015                      H
HETATM   40  H              0       -6.807  -0.413  -0.607                      H
HETATM   41  H              0       -1.773  -0.134   3.008                      H
HETATM   42  H              0       -1.059  -1.707   2.663                      H
HETATM   43  H              0       -2.644  -1.607   3.480                      H
HETATM   44  H              0        1.791  -2.020   2.573                      H
HETATM   45  H              0        3.244  -1.333   3.316                      H
HETATM   46  H              0        3.293  -2.990   2.677                      H
HETATM   47  H              0        6.209   1.498  -0.086                      H
HETATM   48  H              0        7.072   0.075   0.521                      H
HETATM   49  H              0        6.836   0.303  -1.236                      H
```

```
HETATM   50  H              0       1.345  -0.813  -2.574                       H
HETATM   51  H              0       2.865  -1.233  -3.403                       H
HETATM   52  H              0       1.909  -2.484  -2.570                       H
HETATM   53  C              0       1.212   1.704  -0.764                       C
HETATM   54  F              0       1.384   2.034  -2.017                       F
HETATM   55  F              0       2.240   2.164  -0.101                       F
HETATM   56  C              0      -0.486   2.803  -0.190                       C
HETATM   57  C              0      -1.593   1.944   0.230                       C
HETATM   58  F              0      -0.683   3.368  -1.388                       F
HETATM   59 Cl              0       0.161   3.930   0.973                      Cl
HETATM   60  F              0      -2.669   1.937  -0.550                       F
HETATM   61  F              0      -1.994   2.017   1.502                       F
END
```

a5NA.pdb

```
TITLE      a5NA.pdb
HETATM    1 Ru              0       0.026   0.493  -0.048                      Ru
HETATM    2  C              0      -0.900  -3.662   0.361                       C
HETATM    3  C              0       0.618  -3.734   0.147                       C
HETATM    4  H              0      -1.471  -4.249  -0.379                       H
HETATM    5  H              0       0.900  -4.261  -0.784                       H
HETATM    6  C              0      -0.067  -1.522   0.073                       C
HETATM    7  N              0       1.005  -2.320   0.058                       N
HETATM    8  N              0      -1.190  -2.232   0.203                       N
HETATM    9 Cl              0      -0.848   0.170  -2.289                      Cl
HETATM   10 Cl              0       0.875   0.585   2.198                      Cl
HETATM   11  C              0      -1.230   1.981   0.213                       C
HETATM   12  C              0       2.346  -1.825  -0.016                       C
HETATM   13  C              0       3.105  -1.724   1.168                       C
HETATM   14  C              0       2.869  -1.419  -1.260                       C
HETATM   15  C              0       4.362  -1.123   1.096                       C
HETATM   16  C              0       4.136  -0.823  -1.281                       C
HETATM   17  C              0       4.881  -0.642  -0.113                       C
HETATM   18  H              0       4.946  -1.008   2.018                       H
HETATM   19  H              0       4.545  -0.489  -2.244                       H
HETATM   20  C              0      -2.498  -1.650   0.222                       C
HETATM   21  C              0      -2.951  -0.992   1.381                       C
HETATM   22  C              0      -3.299  -1.730  -0.937                       C
HETATM   23  C              0      -4.188  -0.337   1.329                       C
HETATM   24  C              0      -4.527  -1.069  -0.938                       C
HETATM   25  C              0      -4.976  -0.346   0.176                       C
HETATM   26  H              0      -4.542   0.191   2.224                       H
HETATM   27  H              0      -5.143  -1.102  -1.845                       H
HETATM   28  C              0       2.597  -2.263   2.470                       C
HETATM   29  H              0       3.053  -1.733   3.322                       H
HETATM   30  H              0       1.505  -2.152   2.570                       H
HETATM   31  H              0       2.847  -3.337   2.586                       H
HETATM   32  C              0       2.116  -1.612  -2.540                       C
HETATM   33  H              0       1.416  -2.465  -2.496                       H
HETATM   34  H              0       1.494  -0.735  -2.798                       H
HETATM   35  H              0       2.812  -1.782  -3.378                       H
HETATM   36  C              0       6.195   0.075  -0.147                       C
HETATM   37  H              0       6.057   1.154   0.053                       H
HETATM   38  H              0       6.893  -0.301   0.621                       H
HETATM   39  H              0       6.687  -0.007  -1.131                       H
HETATM   40  C              0      -2.170  -0.985   2.660                       C
HETATM   41  H              0      -2.848  -1.075   3.526                       H
HETATM   42  H              0      -1.430  -1.801   2.718                       H
HETATM   43  H              0      -1.595  -0.051   2.792                       H
HETATM   44  C              0      -2.865  -2.518  -2.135                       C
HETATM   45  H              0      -3.182  -3.578  -2.058                       H
```

```
HETATM   46  H              0       -3.314  -2.115  -3.057                          H
HETATM   47  H              0       -1.772  -2.494  -2.275                          H
HETATM   48  C              0       -6.271   0.405   0.119                          C
HETATM   49  H              0       -6.160   1.340  -0.462                          H
HETATM   50  H              0       -7.065  -0.179  -0.379                          H
HETATM   51  H              0       -6.630   0.687   1.122                          H
HETATM   52  H              0        1.151  -4.220   0.982                          H
HETATM   53  H              0       -1.211  -3.996   1.368                          H
HETATM   54  C              0        0.082   2.772  -0.298                          C
HETATM   55  C              0        1.334   1.814  -0.678                          C
HETATM   56  F              0       -2.263   2.218  -0.583                          F
HETATM   57  F              0       -1.619   2.287   1.450                          F
HETATM   58  F              0       -0.274   3.393  -1.440                          F
HETATM   59  F              0        1.654   1.956  -1.962                          F
HETATM   60  F              0        2.438   2.052   0.018                          F
HETATM   61 Cl              0        0.595   3.972   0.909                         Cl
END
```

a5d.pdb

```
TITLE       a5d.pdb
HETATM    1 Ru              0       -0.106   0.553  -0.060                         Ru
HETATM    2 Cl              0       -0.988   0.141  -2.292                         Cl
HETATM    3 Cl              0        0.790   0.661   2.169                         Cl
HETATM    4  C              0        0.122  -1.502   0.082                          C
HETATM    5  N              0       -0.915  -2.337   0.221                          N
HETATM    6  C              0       -0.485  -3.724   0.422                          C
HETATM    7  C              0        1.031  -3.638   0.216                          C
HETATM    8  N              0        1.269  -2.192   0.097                          N
HETATM    9  C              0       -2.264  -1.862   0.243                          C
HETATM   10  C              0       -3.059  -2.019  -0.911                          C
HETATM   11  C              0       -4.310  -1.405  -0.941                          C
HETATM   12  C              0       -4.782  -0.643   0.138                          C
HETATM   13  C              0       -4.006  -0.569   1.294                          C
HETATM   14  C              0       -2.749  -1.186   1.380                          C
HETATM   15  C              0        2.567  -1.600   0.002                          C
HETATM   16  C              0        3.342  -1.445   1.166                          C
HETATM   17  C              0        4.578  -0.800   1.057                          C
HETATM   18  C              0        5.056  -0.325  -0.169                          C
HETATM   19  C              0        4.285  -0.540  -1.317                          C
HETATM   20  C              0        3.044  -1.184  -1.257                          C
HETATM   21  C              0       -2.577  -2.820  -2.081                          C
HETATM   22  C              0       -6.072   0.109   0.029                          C
HETATM   23  C              0       -2.015  -1.161   2.687                          C
HETATM   24  C              0        2.889  -1.974   2.492                          C
HETATM   25  C              0        6.357   0.413  -0.248                          C
HETATM   26  C              0        2.257  -1.436  -2.506                          C
HETATM   27  H              0       -0.984  -4.394  -0.300                          H
HETATM   28  H              0       -0.765  -4.058   1.440                          H
HETATM   29  H              0        1.373  -4.152  -0.701                          H
HETATM   30  H              0        1.609  -4.050   1.063                          H
HETATM   31  H              0       -4.918  -1.487  -1.850                          H
HETATM   32  H              0       -4.374  -0.008   2.163                          H
HETATM   33  H              0        5.179  -0.653   1.964                          H
HETATM   34  H              0        4.663  -0.209  -2.294                          H
HETATM   35  H              0       -3.067  -2.494  -3.013                          H
HETATM   36  H              0       -1.490  -2.714  -2.233                          H
HETATM   37  H              0       -2.802  -3.898  -1.955                          H
HETATM   38  H              0       -5.902   1.101  -0.432                          H
HETATM   39  H              0       -6.533   0.290   1.015                          H
HETATM   40  H              0       -6.807  -0.413  -0.607                          H
HETATM   41  H              0       -1.773  -0.134   3.008                          H
```

```
HETATM   42  H              0      -1.059  -1.707   2.663                      H
HETATM   43  H              0      -2.644  -1.607   3.480                      H
HETATM   44  H              0       1.791  -2.020   2.573                      H
HETATM   45  H              0       3.244  -1.333   3.316                      H
HETATM   46  H              0       3.293  -2.990   2.677                      H
HETATM   47  H              0       6.209   1.498  -0.086                      H
HETATM   48  H              0       7.072   0.075   0.521                      H
HETATM   49  H              0       6.836   0.303  -1.236                      H
HETATM   50  H              0       1.345  -0.813  -2.574                      H
HETATM   51  H              0       2.865  -1.233  -3.403                      H
HETATM   52  H              0       1.909  -2.484  -2.570                      H
HETATM   53  C              0       1.212   1.704  -0.764                      C
HETATM   54  F              0       1.384   2.034  -2.017                      F
HETATM   55  F              0       2.240   2.164  -0.101                      F
HETATM   56  C              0      -0.486   2.803  -0.190                      C
HETATM   57  C              0      -1.593   1.944   0.230                      C
HETATM   58  F              0      -0.683   3.368  -1.388                      F
HETATM   59 Cl              0       0.161   3.930   0.973                     Cl
HETATM   60  F              0      -2.669   1.937  -0.550                      F
HETATM   61  F              0      -1.994   2.017   1.502                      F
END
```

sc5e.pdb

```
TITLE      sc5e.pdb
HETATM    1 Ru              0      -0.171   0.481   0.078                     Ru
HETATM    2  C              0       0.243  -3.646  -0.332                      C
HETATM    3  C              0       1.731  -3.319  -0.404                      C
HETATM    4  H              0      -0.126  -4.196  -1.217                      H
HETATM    5  H              0       2.165  -3.507  -1.406                      H
HETATM    6  C              0       0.545  -1.328  -0.086                      C
HETATM    7  N              0       1.778  -1.882  -0.111                      N
HETATM    8  N              0      -0.367  -2.322  -0.260                      N
HETATM    9 Cl              0      -0.613   0.738  -2.251                     Cl
HETATM   10 Cl              0      -0.371   0.468   2.457                     Cl
HETATM   11  C              0      -2.638   2.331   0.521                      C
HETATM   12  C              0       3.035  -1.212  -0.088                      C
HETATM   13  C              0       3.759  -1.185   1.119                      C
HETATM   14  C              0       3.542  -0.630  -1.262                      C
HETATM   15  C              0       5.013  -0.571   1.130                      C
HETATM   16  C              0       4.796  -0.009  -1.203                      C
HETATM   17  C              0       5.543   0.027  -0.021                      C
HETATM   18  H              0       5.583  -0.541   2.068                      H
HETATM   19  H              0       5.200   0.454  -2.113                      H
HETATM   20  C              0      -1.777  -2.111  -0.156                      C
HETATM   21  C              0      -2.385  -2.095   1.119                      C
HETATM   22  C              0      -2.552  -1.998  -1.331                      C
HETATM   23  C              0      -3.755  -1.823   1.199                      C
HETATM   24  C              0      -3.922  -1.751  -1.200                      C
HETATM   25  C              0      -4.533  -1.629   0.052                      C
HETATM   26  H              0      -4.228  -1.783   2.188                      H
HETATM   27  H              0      -4.527  -1.643  -2.110                      H
HETATM   28  C              0       3.143  -1.718   2.375                      C
HETATM   29  H              0       3.848  -1.675   3.221                      H
HETATM   30  H              0       2.247  -1.126   2.651                      H
HETATM   31  H              0       2.803  -2.766   2.276                      H
HETATM   32  C              0       2.749  -0.626  -2.532                      C
HETATM   33  H              0       2.214  -1.578  -2.704                      H
HETATM   34  H              0       1.963   0.154  -2.522                      H
HETATM   35  H              0       3.394  -0.435  -3.405                      H
HETATM   36  C              0       6.871   0.720   0.027                      C
HETATM   37  H              0       6.777   1.738   0.450                      H
```

```
HETATM   38  H              0       7.594   0.182   0.665                           H
HETATM   39  H              0       7.316   0.831  -0.976                           H
HETATM   40  C              0      -1.637  -2.486   2.358                           C
HETATM   41  H              0      -1.967  -1.901   3.230                           H
HETATM   42  H              0      -1.826  -3.555   2.586                           H
HETATM   43  H              0      -0.549  -2.344   2.274                           H
HETATM   44  C              0      -1.960  -2.214  -2.690                           C
HETATM   45  H              0      -2.183  -3.239  -3.047                           H
HETATM   46  H              0      -2.382  -1.512  -3.427                           H
HETATM   47  H              0      -0.869  -2.075  -2.703                           H
HETATM   48  C              0      -5.986  -1.286   0.164                           C
HETATM   49  H              0      -6.128  -0.191   0.240                           H
HETATM   50  H              0      -6.560  -1.621  -0.717                           H
HETATM   51  H              0      -6.448  -1.726   1.064                           H
HETATM   52  H              0       2.340  -3.881   0.326                           H
HETATM   53  H              0      -0.024  -4.239   0.565                           H
HETATM   54  C              0      -1.614   3.173   0.271                           C
HETATM   55  C              0       1.379   1.412   0.177                           C
HETATM   56  F              0      -2.970   2.023   1.758                           F
HETATM   57  F              0      -0.889   3.704   1.223                           F
HETATM   58  F              0      -1.341   3.647  -0.913                           F
HETATM   59  F              0       2.349   1.285   1.054                           F
HETATM   60 Cl              0       1.733   2.839  -0.788                          Cl
HETATM   61 Cl              0      -3.681   1.741  -0.691                          Cl
END
```

sc5f.pdb

```
TITLE       sc5f.pdb
HETATM    1 Ru              0      -0.029   0.636  -0.046                          Ru
HETATM    2 Cl              0      -0.815   0.196  -2.310                          Cl
HETATM    3 Cl              0       0.770   0.700   2.227                          Cl
HETATM    4  C              0      -0.010  -1.446   0.111                           C
HETATM    5  N              0      -1.099  -2.220   0.224                           N
HETATM    6  C              0      -0.753  -3.638   0.382                           C
HETATM    7  C              0       0.770  -3.639   0.242                           C
HETATM    8  N              0       1.090  -2.209   0.141                           N
HETATM    9  C              0      -2.445  -1.743   0.132                           C
HETATM   10  C              0      -3.137  -1.875  -1.085                           C
HETATM   11  C              0      -4.418  -1.321  -1.180                           C
HETATM   12  C              0      -5.018  -0.664  -0.101                           C
HETATM   13  C              0      -4.328  -0.605   1.115                           C
HETATM   14  C              0      -3.047  -1.150   1.258                           C
HETATM   15  C              0       2.431  -1.716   0.058                           C
HETATM   16  C              0       3.202  -1.621   1.234                           C
HETATM   17  C              0       4.496  -1.106   1.137                           C
HETATM   18  C              0       5.044  -0.717  -0.092                           C
HETATM   19  C              0       4.280  -0.887  -1.249                           C
HETATM   20  C              0       2.973  -1.385  -1.201                           C
HETATM   21  C              0      -2.556  -2.614  -2.251                           C
HETATM   22  C              0      -6.364  -0.022  -0.247                           C
HETATM   23  C              0      -2.354  -1.137   2.587                           C
HETATM   24  C              0       2.676  -2.089   2.556                           C
HETATM   25  C              0       6.408  -0.103  -0.159                           C
HETATM   26  C              0       2.194  -1.560  -2.467                           C
HETATM   27  H              0      -1.261  -4.241  -0.391                           H
HETATM   28  H              0      -1.099  -4.002   1.368                           H
HETATM   29  H              0       1.125  -4.167  -0.663                           H
HETATM   30  H              0       1.287  -4.083   1.110                           H
HETATM   31  H              0      -4.956  -1.395  -2.134                           H
HETATM   32  H              0      -4.797  -0.125   1.984                           H
HETATM   33  H              0       5.093  -1.001   2.052                           H
```

```
HETATM   34  H             0       4.706  -0.619  -2.225                    H
HETATM   35  H             0      -2.888  -2.172  -3.205                    H
HETATM   36  H             0      -1.455  -2.595  -2.257                    H
HETATM   37  H             0      -2.884  -3.673  -2.255                    H
HETATM   38  H             0      -6.941  -0.052   0.693                    H
HETATM   39  H             0      -6.967  -0.501  -1.036                    H
HETATM   40  H             0      -6.267   1.045  -0.524                    H
HETATM   41  H             0      -1.401  -0.576   2.576                    H
HETATM   42  H             0      -2.106  -2.162   2.923                    H
HETATM   43  H             0      -2.994  -0.681   3.360                    H
HETATM   44  H             0       1.582  -1.976   2.635                    H
HETATM   45  H             0       3.120  -1.515   3.385                    H
HETATM   46  H             0       2.926  -3.155   2.732                    H
HETATM   47  H             0       6.355   0.992  -0.002                    H
HETATM   48  H             0       6.885  -0.258  -1.142                    H
HETATM   49  H             0       7.083  -0.500   0.618                    H
HETATM   50  H             0       1.556  -0.684  -2.692                    H
HETATM   51  H             0       2.870  -1.705  -3.326                    H
HETATM   52  H             0       1.502  -2.419  -2.424                    H
HETATM   53  C             0      -1.530   1.741   0.211                    C
HETATM   54  F             0      -2.407   2.082  -0.697                    F
HETATM   55 Cl             0      -2.122   2.268   1.761                   Cl
HETATM   56  C             0       0.185   2.888  -0.296                    C
HETATM   57  C             0       1.255   2.047  -0.817                    C
HETATM   58  F             0      -0.460   3.605  -1.212                    F
HETATM   59  F             0       0.396   3.609   0.793                    F
HETATM   60  F             0       1.427   2.109  -2.143                    F
HETATM   61 Cl             0       2.809   2.058  -0.010                   Cl
END
```

sc5NB.pdb

```
TITLE       sc5NB.pdb
HETATM    1 Ru             0      -0.006   0.518  -0.037                   Ru
HETATM    2  C             0      -0.825  -3.644   0.489                    C
HETATM    3  C             0       0.680  -3.686   0.208                    C
HETATM    4  H             0      -1.415  -4.268  -0.204                    H
HETATM    5  H             0       0.932  -4.194  -0.743                    H
HETATM    6  C             0      -0.054  -1.492   0.127                    C
HETATM    7  N             0       1.037  -2.263   0.116                    N
HETATM    8  N             0      -1.158  -2.226   0.296                    N
HETATM    9 Cl             0      -0.824   0.092  -2.284                   Cl
HETATM   10 Cl             0       0.801   0.610   2.227                   Cl
HETATM   11  C             0      -1.300   1.992   0.131                    C
HETATM   12  C             0       2.377  -1.765   0.018                    C
HETATM   13  C             0       3.160  -1.685   1.189                    C
HETATM   14  C             0       2.895  -1.382  -1.235                    C
HETATM   15  C             0       4.436  -1.128   1.095                    C
HETATM   16  C             0       4.181  -0.830  -1.278                    C
HETATM   17  C             0       4.952  -0.672  -0.125                    C
HETATM   18  H             0       5.039  -1.032   2.007                    H
HETATM   19  H             0       4.585  -0.512  -2.248                    H
HETATM   20  C             0      -2.490  -1.705   0.195                    C
HETATM   21  C             0      -3.063  -1.040   1.296                    C
HETATM   22  C             0      -3.189  -1.860  -1.016                    C
HETATM   23  C             0      -4.317  -0.441   1.125                    C
HETATM   24  C             0      -4.444  -1.257  -1.136                    C
HETATM   25  C             0      -5.008  -0.521  -0.088                    C
HETATM   26  H             0      -4.764   0.097   1.970                    H
HETATM   27  H             0      -4.985  -1.349  -2.086                    H
HETATM   28  C             0       2.661  -2.206   2.502                    C
HETATM   29  H             0       3.131  -1.672   3.344                    H
```

```
HETATM   30  H              0       1.571  -2.085   2.614                         H
HETATM   31  H              0       2.904  -3.280   2.627                         H
HETATM   32  C              0       2.133  -1.560  -2.512                         C
HETATM   33  H              0       1.308  -2.285  -2.424                         H
HETATM   34  H              0       1.666  -0.618  -2.850                         H
HETATM   35  H              0       2.807  -1.900  -3.318                         H
HETATM   36  C              0       6.285   0.007  -0.185                         C
HETATM   37  H              0       6.171   1.100  -0.049                         H
HETATM   38  H              0       6.969  -0.342   0.607                         H
HETATM   39  H              0       6.781  -0.137  -1.160                         H
HETATM   40  C              0      -2.381  -1.011   2.629                         C
HETATM   41  H              0      -3.029  -0.548   3.391                         H
HETATM   42  H              0      -2.131  -2.031   2.979                         H
HETATM   43  H              0      -1.431  -0.444   2.622                         H
HETATM   44  C              0      -2.638  -2.671  -2.149                         C
HETATM   45  H              0      -2.990  -3.721  -2.098                         H
HETATM   46  H              0      -2.969  -2.269  -3.120                         H
HETATM   47  H              0      -1.536  -2.678  -2.168                         H
HETATM   48  C              0      -6.320   0.180  -0.269                         C
HETATM   49  H              0      -6.172   1.187  -0.704                         H
HETATM   50  H              0      -6.990  -0.365  -0.955                         H
HETATM   51  H              0      -6.849   0.322   0.689                         H
HETATM   52  H              0       1.259  -4.171   1.012                         H
HETATM   53  H              0      -1.081  -3.950   1.520                         H
HETATM   54  C              0       0.035   2.793  -0.317                         C
HETATM   55  C              0       1.273   1.843  -0.733                         C
HETATM   56  F              0      -2.269   2.165  -0.763                         F
HETATM   57  F              0      -0.296   3.500  -1.410                         F
HETATM   58  F              0       0.410   3.641   0.649                         F
HETATM   59  F              0       1.511   1.942  -2.045                         F
HETATM   60 Cl              0       2.780   2.176   0.109                        Cl
HETATM   61 Cl              0      -1.941   2.473   1.702                        Cl
END
```

sc5g.pdb

```
TITLE       sc5g.pdb
HETATM    1 Ru              0      -0.029   0.636  -0.046                        Ru
HETATM    2 Cl              0      -0.815   0.196  -2.310                        Cl
HETATM    3 Cl              0       0.770   0.700   2.227                        Cl
HETATM    4  C              0      -0.010  -1.446   0.111                         C
HETATM    5  N              0      -1.099  -2.220   0.224                         N
HETATM    6  C              0      -0.753  -3.638   0.382                         C
HETATM    7  C              0       0.770  -3.639   0.242                         C
HETATM    8  N              0       1.090  -2.209   0.141                         N
HETATM    9  C              0      -2.445  -1.743   0.132                         C
HETATM   10  C              0      -3.137  -1.875  -1.085                         C
HETATM   11  C              0      -4.418  -1.321  -1.180                         C
HETATM   12  C              0      -5.018  -0.664  -0.101                         C
HETATM   13  C              0      -4.328  -0.605   1.115                         C
HETATM   14  C              0      -3.047  -1.150   1.258                         C
HETATM   15  C              0       2.431  -1.716   0.058                         C
HETATM   16  C              0       3.202  -1.621   1.234                         C
HETATM   17  C              0       4.496  -1.106   1.137                         C
HETATM   18  C              0       5.044  -0.717  -0.092                         C
HETATM   19  C              0       4.280  -0.887  -1.249                         C
HETATM   20  C              0       2.973  -1.385  -1.201                         C
HETATM   21  C              0      -2.556  -2.614  -2.251                         C
HETATM   22  C              0      -6.364  -0.022  -0.247                         C
HETATM   23  C              0      -2.354  -1.137   2.587                         C
HETATM   24  C              0       2.676  -2.089   2.556                         C
HETATM   25  C              0       6.408  -0.103  -0.159                         C
```

```
HETATM   26  C              0       2.194  -1.560  -2.467                       C
HETATM   27  H              0      -1.261  -4.241  -0.391                       H
HETATM   28  H              0      -1.099  -4.002   1.368                       H
HETATM   29  H              0       1.125  -4.167  -0.663                       H
HETATM   30  H              0       1.287  -4.083   1.110                       H
HETATM   31  H              0      -4.956  -1.395  -2.134                       H
HETATM   32  H              0      -4.797  -0.125   1.984                       H
HETATM   33  H              0       5.093  -1.001   2.052                       H
HETATM   34  H              0       4.706  -0.619  -2.225                       H
HETATM   35  H              0      -2.888  -2.172  -3.205                       H
HETATM   36  H              0      -1.455  -2.595  -2.257                       H
HETATM   37  H              0      -2.884  -3.673  -2.255                       H
HETATM   38  H              0      -6.941  -0.052   0.693                       H
HETATM   39  H              0      -6.967  -0.501  -1.036                       H
HETATM   40  H              0      -6.267   1.045  -0.524                       H
HETATM   41  H              0      -1.401  -0.576   2.576                       H
HETATM   42  H              0      -2.106  -2.162   2.923                       H
HETATM   43  H              0      -2.994  -0.681   3.360                       H
HETATM   44  H              0       1.582  -1.976   2.635                       H
HETATM   45  H              0       3.120  -1.515   3.385                       H
HETATM   46  H              0       2.926  -3.155   2.732                       H
HETATM   47  H              0       6.355   0.992  -0.002                       H
HETATM   48  H              0       6.885  -0.258  -1.142                       H
HETATM   49  H              0       7.083  -0.500   0.618                       H
HETATM   50  H              0       1.556  -0.684  -2.692                       H
HETATM   51  H              0       2.870  -1.705  -3.326                       H
HETATM   52  H              0       1.502  -2.419  -2.424                       H
HETATM   53  C              0      -1.530   1.741   0.211                       C
HETATM   54  F              0      -2.407   2.082  -0.697                       F
HETATM   55 Cl              0      -2.122   2.268   1.761                      Cl
HETATM   56  C              0       0.185   2.888  -0.296                       C
HETATM   57  C              0       1.255   2.047  -0.817                       C
HETATM   58  F              0      -0.460   3.605  -1.212                       F
HETATM   59  F              0       0.396   3.609   0.793                       F
HETATM   60  F              0       1.427   2.109  -2.143                       F
HETATM   61 Cl              0       2.809   2.058  -0.010                      Cl
END
```

sc5h.pdb

```
TITLE       sc5h.pdb
HETATM    1 Ru              0      -0.171   0.481   0.078                      Ru
HETATM    2  C              0       0.243  -3.646  -0.332                       C
HETATM    3  C              0       1.731  -3.319  -0.404                       C
HETATM    4  H              0      -0.126  -4.196  -1.217                       H
HETATM    5  H              0       2.165  -3.507  -1.406                       H
HETATM    6  C              0       0.545  -1.328  -0.086                       C
HETATM    7  N              0       1.778  -1.882  -0.111                       N
HETATM    8  N              0      -0.367  -2.322  -0.260                       N
HETATM    9 Cl              0      -0.613   0.738  -2.251                      Cl
HETATM   10 Cl              0      -0.371   0.468   2.457                      Cl
HETATM   11  C              0      -2.638   2.331   0.521                       C
HETATM   12  C              0       3.035  -1.212  -0.088                       C
HETATM   13  C              0       3.759  -1.185   1.119                       C
HETATM   14  C              0       3.542  -0.630  -1.262                       C
HETATM   15  C              0       5.013  -0.571   1.130                       C
HETATM   16  C              0       4.796  -0.009  -1.203                       C
HETATM   17  C              0       5.543   0.027  -0.021                       C
HETATM   18  H              0       5.583  -0.541   2.068                       H
HETATM   19  H              0       5.200   0.454  -2.113                       H
HETATM   20  C              0      -1.777  -2.111  -0.156                       C
HETATM   21  C              0      -2.385  -2.095   1.119                       C
```

```
HETATM   22  C             0      -2.552  -1.998  -1.331                      C
HETATM   23  C             0      -3.755  -1.823   1.199                      C
HETATM   24  C             0      -3.922  -1.751  -1.200                      C
HETATM   25  C             0      -4.533  -1.629   0.052                      C
HETATM   26  H             0      -4.228  -1.783   2.188                      H
HETATM   27  H             0      -4.527  -1.643  -2.110                      H
HETATM   28  C             0       3.143  -1.718   2.375                      C
HETATM   29  H             0       3.848  -1.675   3.221                      H
HETATM   30  H             0       2.247  -1.126   2.651                      H
HETATM   31  H             0       2.803  -2.766   2.276                      H
HETATM   32  C             0       2.749  -0.626  -2.532                      C
HETATM   33  H             0       2.214  -1.578  -2.704                      H
HETATM   34  H             0       1.963   0.154  -2.522                      H
HETATM   35  H             0       3.394  -0.435  -3.405                      H
HETATM   36  C             0       6.871   0.720   0.027                      C
HETATM   37  H             0       6.777   1.738   0.450                      H
HETATM   38  H             0       7.594   0.182   0.665                      H
HETATM   39  H             0       7.316   0.831  -0.976                      H
HETATM   40  C             0      -1.637  -2.486   2.358                      C
HETATM   41  H             0      -1.967  -1.901   3.230                      H
HETATM   42  H             0      -1.826  -3.555   2.586                      H
HETATM   43  H             0      -0.549  -2.344   2.274                      H
HETATM   44  C             0      -1.960  -2.214  -2.690                      C
HETATM   45  H             0      -2.183  -3.239  -3.047                      H
HETATM   46  H             0      -2.382  -1.512  -3.427                      H
HETATM   47  H             0      -0.869  -2.075  -2.703                      H
HETATM   48  C             0      -5.986  -1.286   0.164                      C
HETATM   49  H             0      -6.128  -0.191   0.240                      H
HETATM   50  H             0      -6.560  -1.621  -0.717                      H
HETATM   51  H             0      -6.448  -1.726   1.064                      H
HETATM   52  H             0       2.340  -3.881   0.326                      H
HETATM   53  H             0      -0.024  -4.239   0.565                      H
HETATM   54  C             0      -1.614   3.173   0.271                      C
HETATM   55  C             0       1.379   1.412   0.177                      C
HETATM   56  F             0      -2.970   2.023   1.758                      F
HETATM   57  F             0      -0.889   3.704   1.223                      F
HETATM   58  F             0      -1.341   3.647  -0.913                      F
HETATM   59  F             0       2.349   1.285   1.054                      F
HETATM   60 Cl             0       1.733   2.839  -0.788                     Cl
HETATM   61 Cl             0      -3.681   1.741  -0.691                     Cl
END
```

st5e.pdb

```
TITLE      st5e.pdb
HETATM    1 Ru             0      -0.152   0.454   0.002                     Ru
HETATM    2  C             0       0.450  -3.656   0.156                      C
HETATM    3  C             0       1.928  -3.281   0.064                      C
HETATM    4  H             0       0.124  -4.329  -0.658                      H
HETATM    5  H             0       2.399  -3.611  -0.882                      H
HETATM    6  C             0       0.660  -1.315   0.066                      C
HETATM    7  N             0       1.915  -1.815   0.122                      N
HETATM    8  N             0      -0.212  -2.361   0.051                      N
HETATM    9 Cl             0      -0.625   0.563  -2.327                     Cl
HETATM   10 Cl             0      -0.340   0.609   2.378                     Cl
HETATM   11  C             0      -3.004   2.061   0.130                      C
HETATM   12  C             0       3.142  -1.092   0.063                      C
HETATM   13  C             0       3.854  -0.876   1.256                      C
HETATM   14  C             0       3.629  -0.640  -1.175                      C
HETATM   15  C             0       5.079  -0.209   1.190                      C
HETATM   16  C             0       4.853   0.042  -1.195                      C
HETATM   17  C             0       5.590   0.261  -0.027                      C
```

```
HETATM   18  H            0       5.638  -0.029   2.117                        H
HETATM   19  H            0       5.242   0.402  -2.157                        H
HETATM   20  C            0      -1.623  -2.180  -0.076                        C
HETATM   21  C            0      -2.424  -2.023   1.076                        C
HETATM   22  C            0      -2.204  -2.200  -1.365                        C
HETATM   23  C            0      -3.785  -1.752   0.904                        C
HETATM   24  C            0      -3.568  -1.927  -1.485                        C
HETATM   25  C            0      -4.366  -1.667  -0.365                        C
HETATM   26  H            0      -4.408  -1.599   1.795                        H
HETATM   27  H            0      -4.016  -1.904  -2.486                        H
HETATM   28  C            0       3.250  -1.261   2.571                        C
HETATM   29  H            0       3.922  -1.017   3.410                        H
HETATM   30  H            0       2.296  -0.721   2.732                        H
HETATM   31  H            0       3.014  -2.339   2.638                        H
HETATM   32  C            0       2.847  -0.848  -2.435                        C
HETATM   33  H            0       2.456  -1.879  -2.521                        H
HETATM   34  H            0       1.961  -0.184  -2.488                        H
HETATM   35  H            0       3.464  -0.647  -3.325                        H
HETATM   36  C            0       6.882   1.019  -0.069                        C
HETATM   37  H            0       6.721   2.098   0.118                        H
HETATM   38  H            0       7.591   0.669   0.701                        H
HETATM   39  H            0       7.377   0.938  -1.052                        H
HETATM   40  C            0      -1.887  -2.239   2.457                        C
HETATM   41  H            0      -2.309  -1.512   3.169                        H
HETATM   42  H            0      -2.167  -3.250   2.816                        H
HETATM   43  H            0      -0.793  -2.145   2.516                        H
HETATM   44  C            0      -1.404  -2.572  -2.575                        C
HETATM   45  H            0      -1.429  -3.668  -2.736                        H
HETATM   46  H            0      -1.811  -2.099  -3.483                        H
HETATM   47  H            0      -0.348  -2.266  -2.498                        H
HETATM   48  C            0      -5.803  -1.278  -0.516                        C
HETATM   49  H            0      -5.912  -0.177  -0.478                        H
HETATM   50  H            0      -6.226  -1.611  -1.478                        H
HETATM   51  H            0      -6.431  -1.684   0.297                        H
HETATM   52  H            0       2.534  -3.692   0.890                        H
HETATM   53  H            0       0.189  -4.144   1.116                        H
HETATM   54  C            0      -2.057   2.993  -0.089                        C
HETATM   55  C            0       1.326   1.499   0.033                        C
HETATM   56  F            0      -1.449   3.642   0.870                        F
HETATM   57  F            0      -1.742   3.417  -1.283                        F
HETATM   58  F            0       2.323   1.478   0.888                        F
HETATM   59 Cl            0       1.520   2.925  -0.971                       Cl
HETATM   60  F            0      -3.614   1.482  -0.891                        F
HETATM   61 Cl            0      -3.623   1.687   1.672                       Cl
END
```

st5f.pdb

```
TITLE      st5f.pdb
HETATM    1 Ru            0       0.060  -0.585  -0.012                       Ru
HETATM    2 Cl            0       0.810  -0.444  -2.303                       Cl
HETATM    3 Cl            0      -0.661  -0.423   2.307                       Cl
HETATM    4  C            0      -0.067   1.493   0.067                        C
HETATM    5  N            0       0.999   2.297   0.155                        N
HETATM    6  C            0       0.618   3.711   0.269                        C
HETATM    7  C            0      -0.910   3.660   0.185                        C
HETATM    8  N            0      -1.191   2.220   0.101                        N
HETATM    9  C            0       2.343   1.821   0.021                        C
HETATM   10  C            0       2.960   1.860  -1.244                        C
HETATM   11  C            0       4.229   1.290  -1.378                        C
HETATM   12  C            0       4.888   0.701  -0.294                        C
HETATM   13  C            0       4.280   0.741   0.965                        C
```

```
HETATM   14  C              0        3.014   1.307   1.151                     C
HETATM   15  C              0       -2.508   1.666   0.057                     C
HETATM   16  C              0       -3.270   1.579   1.237                     C
HETATM   17  C              0       -4.522   0.958   1.170                     C
HETATM   18  C              0       -5.026   0.445  -0.029                     C
HETATM   19  C              0       -4.272   0.604  -1.197                     C
HETATM   20  C              0       -3.018   1.224  -1.179                     C
HETATM   21  C              0        2.301   2.510  -2.422                     C
HETATM   22  C              0        6.207   0.016  -0.483                     C
HETATM   23  C              0        2.409   1.393   2.519                     C
HETATM   24  C              0       -2.793   2.163   2.532                     C
HETATM   25  C              0       -6.339  -0.276  -0.060                     C
HETATM   26  C              0       -2.252   1.431  -2.449                     C
HETATM   27  H              0        1.080   4.294  -0.548                     H
HETATM   28  H              0        0.988   4.126   1.225                     H
HETATM   29  H              0       -1.314   4.174  -0.707                     H
HETATM   30  H              0       -1.408   4.093   1.071                     H
HETATM   31  H              0        4.703   1.285  -2.368                     H
HETATM   32  H              0        4.798   0.315   1.833                     H
HETATM   33  H              0       -5.113   0.864   2.090                     H
HETATM   34  H              0       -4.667   0.240  -2.154                     H
HETATM   35  H              0        2.579   2.001  -3.359                     H
HETATM   36  H              0        1.201   2.485  -2.360                     H
HETATM   37  H              0        2.613   3.569  -2.520                     H
HETATM   38  H              0        6.061  -1.053  -0.730                     H
HETATM   39  H              0        6.788   0.455  -1.311                     H
HETATM   40  H              0        6.826   0.048   0.429                     H
HETATM   41  H              0        3.109   1.013   3.281                     H
HETATM   42  H              0        1.471   0.812   2.612                     H
HETATM   43  H              0        2.161   2.437   2.787                     H
HETATM   44  H              0       -1.695   2.212   2.591                     H
HETATM   45  H              0       -3.132   1.558   3.389                     H
HETATM   46  H              0       -3.195   3.186   2.678                     H
HETATM   47  H              0       -6.202  -1.358   0.125                     H
HETATM   48  H              0       -7.032   0.092   0.716                     H
HETATM   49  H              0       -6.839  -0.182  -1.039                     H
HETATM   50  H              0       -1.326   0.825  -2.499                     H
HETATM   51  H              0       -2.864   1.164  -3.326                     H
HETATM   52  H              0       -1.938   2.485  -2.570                     H
HETATM   53  C              0       -1.403  -1.719  -0.357                     C
HETATM   54 Cl              0       -2.007  -2.131  -1.934                    Cl
HETATM   55  F              0       -2.263  -2.142   0.534                     F
HETATM   56  C              0        0.340  -2.850   0.055                     C
HETATM   57  C              0        1.484  -2.022   0.418                     C
HETATM   58  F              0        0.460  -3.557  -1.059                     F
HETATM   59  F              0       -0.220  -3.577   1.013                     F
HETATM   60  F              0        2.490  -2.041  -0.466                     F
HETATM   61 Cl              0        2.133  -2.144   2.041                    Cl
END
```

st5NB.pdb

```
TITLE       st5NB.pdb
HETATM    1 Ru              0        0.014  -0.500   0.006                    Ru
HETATM    2  C              0        0.794   3.693   0.318                     C
HETATM    3  C              0       -0.726   3.712   0.126                     C
HETATM    4  H              0        1.336   4.263  -0.457                     H
HETATM    5  H              0       -1.041   4.212  -0.809                     H
HETATM    6  C              0        0.024   1.521   0.093                     C
HETATM    7  N              0       -1.072   2.284   0.067                     N
HETATM    8  N              0        1.127   2.265   0.211                     N
HETATM    9 Cl              0        0.809  -0.373  -2.271                    Cl
```

```
HETATM   10 Cl          0      -0.731  -0.341   2.304                    Cl
HETATM   11  C          0       1.275  -1.972   0.380                     C
HETATM   12  C          0      -2.404   1.757   0.023                     C
HETATM   13  C          0      -3.165   1.693   1.207                     C
HETATM   14  C          0      -2.922   1.305  -1.208                     C
HETATM   15  C          0      -4.413   1.068   1.154                     C
HETATM   16  C          0      -4.175   0.683  -1.209                     C
HETATM   17  C          0      -4.919   0.530  -0.035                     C
HETATM   18  H          0      -5.000   0.985   2.078                     H
HETATM   19  H          0      -4.574   0.305  -2.159                     H
HETATM   20  C          0       2.451   1.735   0.068                     C
HETATM   21  C          0       3.094   1.160   1.183                     C
HETATM   22  C          0       3.068   1.781  -1.195                     C
HETATM   23  C          0       4.335   0.545   0.982                     C
HETATM   24  C          0       4.313   1.165  -1.345                     C
HETATM   25  C          0       4.945   0.520  -0.277                     C
HETATM   26  H          0       4.837   0.078   1.839                     H
HETATM   27  H          0       4.790   1.170  -2.333                     H
HETATM   28  C          0      -2.689   2.308   2.487                     C
HETATM   29  H          0      -3.043   1.733   3.358                     H
HETATM   30  H          0      -1.591   2.346   2.556                     H
HETATM   31  H          0      -3.080   3.339   2.599                     H
HETATM   32  C          0      -2.176   1.508  -2.491                     C
HETATM   33  H          0      -1.842   2.556  -2.609                     H
HETATM   34  H          0      -1.268   0.879  -2.567                     H
HETATM   35  H          0      -2.813   1.262  -3.356                     H
HETATM   36  C          0      -6.217  -0.216  -0.045                     C
HETATM   37  H          0      -6.051  -1.292   0.151                     H
HETATM   38  H          0      -6.910   0.145   0.734                     H
HETATM   39  H          0      -6.727  -0.147  -1.021                     H
HETATM   40  C          0       2.490   1.232   2.552                     C
HETATM   41  H          0       3.195   0.857   3.311                     H
HETATM   42  H          0       2.226   2.271   2.826                     H
HETATM   43  H          0       1.560   0.641   2.645                     H
HETATM   44  C          0       2.438   2.488  -2.357                     C
HETATM   45  H          0       2.795   3.535  -2.431                     H
HETATM   46  H          0       2.693   1.991  -3.307                     H
HETATM   47  H          0       1.337   2.507  -2.298                     H
HETATM   48  C          0       6.243  -0.201  -0.480                     C
HETATM   49  H          0       6.067  -1.267  -0.720                     H
HETATM   50  H          0       6.824   0.222  -1.316                     H
HETATM   51  H          0       6.874  -0.181   0.424                     H
HETATM   52  H          0      -1.264   4.194   0.961                     H
HETATM   53  H          0       1.113   4.079   1.304                     H
HETATM   54  C          0      -0.070  -2.788   0.002                     C
HETATM   55  C          0      -1.348  -1.869  -0.358                     C
HETATM   56  F          0       2.248  -2.238  -0.493                     F
HETATM   57  F          0       0.233  -3.586  -1.033                     F
HETATM   58  F          0      -0.438  -3.548   1.043                     F
HETATM   59  F          0      -2.335  -2.090   0.509                     F
HETATM   60 Cl          0      -1.998  -2.112  -1.977                    Cl
HETATM   61 Cl          0       1.904  -2.320   1.985                    Cl
END
```

st5g.pdb

```
TITLE       st5g.pdb
HETATM    1 Ru          0       0.060  -0.585  -0.012                    Ru
HETATM    2 Cl          0       0.810  -0.444  -2.303                    Cl
HETATM    3 Cl          0      -0.661  -0.423   2.307                    Cl
HETATM    4  C          0      -0.067   1.493   0.067                     C
HETATM    5  N          0       0.999   2.297   0.155                     N
```

```
HETATM    6  C              0       0.618   3.711   0.269                          C
HETATM    7  C              0      -0.910   3.660   0.185                          C
HETATM    8  N              0      -1.191   2.220   0.101                          N
HETATM    9  C              0       2.343   1.821   0.021                          C
HETATM   10  C              0       2.960   1.860  -1.244                          C
HETATM   11  C              0       4.229   1.290  -1.378                          C
HETATM   12  C              0       4.888   0.701  -0.294                          C
HETATM   13  C              0       4.280   0.741   0.965                          C
HETATM   14  C              0       3.014   1.307   1.151                          C
HETATM   15  C              0      -2.508   1.666   0.057                          C
HETATM   16  C              0      -3.270   1.579   1.237                          C
HETATM   17  C              0      -4.522   0.958   1.170                          C
HETATM   18  C              0      -5.026   0.445  -0.029                          C
HETATM   19  C              0      -4.272   0.604  -1.197                          C
HETATM   20  C              0      -3.018   1.224  -1.179                          C
HETATM   21  C              0       2.301   2.510  -2.422                          C
HETATM   22  C              0       6.207   0.016  -0.483                          C
HETATM   23  C              0       2.409   1.393   2.519                          C
HETATM   24  C              0      -2.793   2.163   2.532                          C
HETATM   25  C              0      -6.339  -0.276  -0.060                          C
HETATM   26  C              0      -2.252   1.431  -2.449                          C
HETATM   27  H              0       1.080   4.294  -0.548                          H
HETATM   28  H              0       0.988   4.126   1.225                          H
HETATM   29  H              0      -1.314   4.174  -0.707                          H
HETATM   30  H              0      -1.408   4.093   1.071                          H
HETATM   31  H              0       4.703   1.285  -2.368                          H
HETATM   32  H              0       4.798   0.315   1.833                          H
HETATM   33  H              0      -5.113   0.864   2.090                          H
HETATM   34  H              0      -4.667   0.240  -2.154                          H
HETATM   35  H              0       2.579   2.001  -3.359                          H
HETATM   36  H              0       1.201   2.485  -2.360                          H
HETATM   37  H              0       2.613   3.569  -2.520                          H
HETATM   38  H              0       6.061  -1.053  -0.730                          H
HETATM   39  H              0       6.788   0.455  -1.311                          H
HETATM   40  H              0       6.826   0.048   0.429                          H
HETATM   41  H              0       3.109   1.013   3.281                          H
HETATM   42  H              0       1.471   0.812   2.612                          H
HETATM   43  H              0       2.161   2.437   2.787                          H
HETATM   44  H              0      -1.695   2.212   2.591                          H
HETATM   45  H              0      -3.132   1.558   3.389                          H
HETATM   46  H              0      -3.195   3.186   2.678                          H
HETATM   47  H              0      -6.202  -1.358   0.125                          H
HETATM   48  H              0      -7.032   0.092   0.716                          H
HETATM   49  H              0      -6.839  -0.182  -1.039                          H
HETATM   50  H              0      -1.326   0.825  -2.499                          H
HETATM   51  H              0      -2.864   1.164  -3.326                          H
HETATM   52  H              0      -1.938   2.485  -2.570                          H
HETATM   53  C              0      -1.403  -1.719  -0.357                          C
HETATM   54 Cl              0      -2.007  -2.131  -1.934                         Cl
HETATM   55  F              0      -2.263  -2.142   0.534                          F
HETATM   56  C              0       0.340  -2.850   0.055                          C
HETATM   57  C              0       1.484  -2.022   0.418                          C
HETATM   58  F              0       0.460  -3.557  -1.059                          F
HETATM   59  F              0      -0.220  -3.577   1.013                          F
HETATM   60  F              0       2.490  -2.041  -0.466                          F
HETATM   61 Cl              0       2.133  -2.144   2.041                         Cl
END
```

st5h.pdb

```
TITLE      st5h.pdb
REMARK   1 File created by GaussView 5.0.8
```

```
HETATM    1 Ru           0       -0.152   0.454   0.002                 Ru
HETATM    2  C           0        0.450  -3.656   0.156                  C
HETATM    3  C           0        1.928  -3.281   0.064                  C
HETATM    4  H           0        0.124  -4.329  -0.658                  H
HETATM    5  H           0        2.399  -3.611  -0.882                  H
HETATM    6  C           0        0.660  -1.315   0.066                  C
HETATM    7  N           0        1.915  -1.815   0.122                  N
HETATM    8  N           0       -0.212  -2.361   0.051                  N
HETATM    9 Cl           0       -0.625   0.563  -2.327                 Cl
HETATM   10 Cl           0       -0.340   0.609   2.378                 Cl
HETATM   11  C           0       -3.004   2.061   0.130                  C
HETATM   12  C           0        3.142  -1.092   0.063                  C
HETATM   13  C           0        3.854  -0.876   1.256                  C
HETATM   14  C           0        3.629  -0.640  -1.175                  C
HETATM   15  C           0        5.079  -0.209   1.190                  C
HETATM   16  C           0        4.853   0.042  -1.195                  C
HETATM   17  C           0        5.590   0.261  -0.027                  C
HETATM   18  H           0        5.638  -0.029   2.117                  H
HETATM   19  H           0        5.242   0.402  -2.157                  H
HETATM   20  C           0       -1.623  -2.180  -0.076                  C
HETATM   21  C           0       -2.424  -2.023   1.076                  C
HETATM   22  C           0       -2.204  -2.200  -1.365                  C
HETATM   23  C           0       -3.785  -1.752   0.904                  C
HETATM   24  C           0       -3.568  -1.927  -1.485                  C
HETATM   25  C           0       -4.366  -1.667  -0.365                  C
HETATM   26  H           0       -4.408  -1.599   1.795                  H
HETATM   27  H           0       -4.016  -1.904  -2.486                  H
HETATM   28  C           0        3.250  -1.261   2.571                  C
HETATM   29  H           0        3.922  -1.017   3.410                  H
HETATM   30  H           0        2.296  -0.721   2.732                  H
HETATM   31  H           0        3.014  -2.339   2.638                  H
HETATM   32  C           0        2.847  -0.848  -2.435                  C
HETATM   33  H           0        2.456  -1.879  -2.521                  H
HETATM   34  H           0        1.961  -0.184  -2.488                  H
HETATM   35  H           0        3.464  -0.647  -3.325                  H
HETATM   36  C           0        6.882   1.019  -0.069                  C
HETATM   37  H           0        6.721   2.098   0.118                  H
HETATM   38  H           0        7.591   0.669   0.701                  H
HETATM   39  H           0        7.377   0.938  -1.052                  H
HETATM   40  C           0       -1.887  -2.239   2.457                  C
HETATM   41  H           0       -2.309  -1.512   3.169                  H
HETATM   42  H           0       -2.167  -3.250   2.816                  H
HETATM   43  H           0       -0.793  -2.145   2.516                  H
HETATM   44  C           0       -1.404  -2.572  -2.575                  C
HETATM   45  H           0       -1.429  -3.668  -2.736                  H
HETATM   46  H           0       -1.811  -2.099  -3.483                  H
HETATM   47  H           0       -0.348  -2.266  -2.498                  H
HETATM   48  C           0       -5.803  -1.278  -0.516                  C
HETATM   49  H           0       -5.912  -0.177  -0.478                  H
HETATM   50  H           0       -6.226  -1.611  -1.478                  H
HETATM   51  H           0       -6.431  -1.684   0.297                  H
HETATM   52  H           0        2.534  -3.692   0.890                  H
HETATM   53  H           0        0.189  -4.144   1.116                  H
HETATM   54  C           0       -2.057   2.993  -0.089                  C
HETATM   55  C           0        1.326   1.499   0.033                  C
HETATM   56  F           0       -1.449   3.642   0.870                  F
HETATM   57  F           0       -1.742   3.417  -1.283                  F
HETATM   58  F           0        2.323   1.478   0.888                  F
HETATM   59 Cl           0        1.520   2.925  -0.971                 Cl
HETATM   60  F           0       -3.614   1.482  -0.891                  F
HETATM   61 Cl           0       -3.623   1.687   1.672                 Cl
END
```

ac5e.pdb

```
TITLE       ac5e.pdb
HETATM    1 Ru             0       -0.187   0.408  -0.114                     Ru
HETATM    2  C             0        0.434  -3.628   0.719                      C
HETATM    3  C             0        1.895  -3.198   0.824                      C
HETATM    4  H             0        0.254  -4.343  -0.110                      H
HETATM    5  H             0        2.575  -3.800   0.195                      H
HETATM    6  C             0        0.614  -1.337   0.217                      C
HETATM    7  N             0        1.874  -1.804   0.370                      N
HETATM    8  N             0       -0.246  -2.366   0.455                      N
HETATM    9 Cl             0       -0.600   0.166  -2.443                     Cl
HETATM   10 Cl             0       -0.554   0.918   2.177                     Cl
HETATM   11  C             0       -3.065   1.912  -0.405                      C
HETATM   12  C             0        3.100  -1.095   0.219                      C
HETATM   13  C             0        3.619  -0.334   1.280                      C
HETATM   14  C             0        3.792  -1.223  -1.000                      C
HETATM   15  C             0        4.853   0.304   1.094                      C
HETATM   16  C             0        5.025  -0.584  -1.139                      C
HETATM   17  C             0        5.568   0.186  -0.101                      C
HETATM   18  H             0        5.267   0.904   1.914                      H
HETATM   19  H             0        5.568  -0.674  -2.089                      H
HETATM   20  C             0       -1.660  -2.240   0.303                      C
HETATM   21  C             0       -2.457  -1.976   1.438                      C
HETATM   22  C             0       -2.244  -2.426  -0.970                      C
HETATM   23  C             0       -3.826  -1.762   1.251                      C
HETATM   24  C             0       -3.616  -2.201  -1.107                      C
HETATM   25  C             0       -4.414  -1.836  -0.017                      C
HETATM   26  H             0       -4.447  -1.520   2.123                      H
HETATM   27  H             0       -4.072  -2.306  -2.100                      H
HETATM   28  C             0        2.862  -0.163   2.560                      C
HETATM   29  H             0        3.535   0.111   3.390                      H
HETATM   30  H             0        2.095   0.631   2.478                      H
HETATM   31  H             0        2.306  -1.072   2.853                      H
HETATM   32  C             0        3.163  -1.953  -2.146                      C
HETATM   33  H             0        2.900  -2.999  -1.899                      H
HETATM   34  H             0        2.220  -1.460  -2.455                      H
HETATM   35  H             0        3.830  -1.978  -3.023                      H
HETATM   36  C             0        6.875   0.897  -0.287                      C
HETATM   37  H             0        6.740   1.848  -0.835                      H
HETATM   38  H             0        7.349   1.148   0.677                      H
HETATM   39  H             0        7.591   0.296  -0.874                      H
HETATM   40  C             0       -1.889  -2.011   2.823                      C
HETATM   41  H             0       -2.380  -1.275   3.479                      H
HETATM   42  H             0       -2.050  -3.011   3.275                      H
HETATM   43  H             0       -0.809  -1.797   2.848                      H
HETATM   44  C             0       -1.449  -2.935  -2.132                      C
HETATM   45  H             0       -1.526  -4.039  -2.194                      H
HETATM   46  H             0       -1.823  -2.526  -3.084                      H
HETATM   47  H             0       -0.380  -2.675  -2.071                      H
HETATM   48  C             0       -5.858  -1.494  -0.209                      C
HETATM   49  H             0       -5.977  -0.411  -0.407                      H
HETATM   50  H             0       -6.301  -2.024  -1.069                      H
HETATM   51  H             0       -6.463  -1.720   0.686                      H
HETATM   52  H             0        2.282  -3.245   1.861                      H
HETATM   53  H             0        0.050  -4.090   1.646                      H
HETATM   54  C             0       -2.221   2.956  -0.497                      C
HETATM   55  C             0        1.301   1.383  -0.431                      C
HETATM   56  F             0       -3.631   1.545   0.715                      F
HETATM   57  F             0       -1.721   3.322  -1.666                      F
```

```
HETATM   58  F             0       2.250   1.154  -1.311                         F
HETATM   59  F             0      -3.478   1.220  -1.435                         F
HETATM   60 Cl             0       1.564   2.982   0.241                        Cl
HETATM   61 Cl             0      -1.863   3.973   0.821                        Cl
END
```

ac5f.pdb

```
TITLE       ac5f.pdb
HETATM    1 Ru             0      -0.062   0.543  -0.063                        Ru
HETATM    2 Cl             0      -0.905   0.150  -2.319                        Cl
HETATM    3 Cl             0       0.676   0.603   2.215                        Cl
HETATM    4  C             0      -0.078  -1.521   0.074                         C
HETATM    5  N             0      -1.185  -2.264   0.193                         N
HETATM    6  C             0      -0.875  -3.687   0.370                         C
HETATM    7  C             0       0.646  -3.729   0.199                         C
HETATM    8  N             0       1.006  -2.308   0.092                         N
HETATM    9  C             0      -2.506  -1.717   0.211                         C
HETATM   10  C             0      -3.293  -1.799  -0.958                         C
HETATM   11  C             0      -4.539  -1.173  -0.962                         C
HETATM   12  C             0      -5.024  -0.487   0.161                         C
HETATM   13  C             0      -4.252  -0.481   1.323                         C
HETATM   14  C             0      -2.995  -1.099   1.378                         C
HETATM   15  C             0       2.355  -1.836   0.016                         C
HETATM   16  C             0       3.117  -1.735   1.196                         C
HETATM   17  C             0       4.409  -1.206   1.108                         C
HETATM   18  C             0       4.956  -0.803  -0.114                         C
HETATM   19  C             0       4.200  -0.978  -1.278                         C
HETATM   20  C             0       2.902  -1.497  -1.239                         C
HETATM   21  C             0      -2.832  -2.563  -2.161                         C
HETATM   22  C             0      -6.334   0.237   0.102                         C
HETATM   23  C             0      -2.251  -1.111   2.679                         C
HETATM   24  C             0       2.586  -2.202   2.517                         C
HETATM   25  C             0       6.308  -0.161  -0.176                         C
HETATM   26  C             0       2.130  -1.689  -2.507                         C
HETATM   27  H             0      -1.415  -4.292  -0.379                         H
HETATM   28  H             0      -1.208  -4.019   1.372                         H
HETATM   29  H             0       0.965  -4.268  -0.713                         H
HETATM   30  H             0       1.168  -4.189   1.057                         H
HETATM   31  H             0      -5.144  -1.206  -1.877                         H
HETATM   32  H             0      -4.630   0.020   2.224                         H
HETATM   33  H             0       4.997  -1.095   2.028                         H
HETATM   34  H             0       4.628  -0.701  -2.250                         H
HETATM   35  H             0      -3.244  -2.130  -3.087                         H
HETATM   36  H             0      -1.735  -2.556  -2.265                         H
HETATM   37  H             0      -3.169  -3.619  -2.118                         H
HETATM   38  H             0      -6.212   1.236  -0.359                         H
HETATM   39  H             0      -6.764   0.396   1.105                         H
HETATM   40  H             0      -7.077  -0.302  -0.511                         H
HETATM   41  H             0      -1.936  -0.096   2.978                         H
HETATM   42  H             0      -1.333  -1.720   2.653                         H
HETATM   43  H             0      -2.899  -1.500   3.485                         H
HETATM   44  H             0       1.493  -2.077   2.595                         H
HETATM   45  H             0       3.035  -1.635   3.348                         H
HETATM   46  H             0       2.825  -3.271   2.690                         H
HETATM   47  H             0       6.219   0.943  -0.177                         H
HETATM   48  H             0       6.937  -0.429   0.690                         H
HETATM   49  H             0       6.856  -0.432  -1.095                         H
HETATM   50  H             0       1.361  -0.908  -2.662                         H
HETATM   51  H             0       2.801  -1.672  -3.381                         H
HETATM   52  H             0       1.581  -2.648  -2.522                         H
HETATM   53  C             0       1.306   1.627  -0.784                         C
```

```
HETATM   54  F             0       1.454   1.958  -2.044                      F
HETATM   55 Cl             0       2.769   2.014   0.065                     Cl
HETATM   56  C             0      -0.312   2.817  -0.209                      C
HETATM   57  C             0      -1.486   1.991   0.128                      C
HETATM   58  F             0      -0.433   3.415  -1.400                      F
HETATM   59 Cl             0       0.290   3.906   1.010                     Cl
HETATM   60  F             0      -2.513   2.075  -0.709                      F
HETATM   61  F             0      -1.959   2.070   1.376                      F
END
```

ac5PB.pdb

```
TITLE      ac5PB.pdb
HETATM    1 Ru             0      -0.076   0.463  -0.035                     Ru
HETATM    2  C             0      -0.927  -3.705   0.357                      C
HETATM    3  C             0       0.591  -3.759   0.147                      C
HETATM    4  H             0      -1.490  -4.308  -0.376                      H
HETATM    5  H             0       0.882  -4.276  -0.787                      H
HETATM    6  C             0      -0.116  -1.556   0.064                      C
HETATM    7  N             0       0.966  -2.340   0.067                      N
HETATM    8  N             0      -1.232  -2.281   0.182                      N
HETATM    9 Cl             0      -0.895   0.093  -2.298                     Cl
HETATM   10 Cl             0       0.643   0.552   2.245                     Cl
HETATM   11  C             0      -1.376   1.908   0.145                      C
HETATM   12  C             0       2.312  -1.855  -0.010                      C
HETATM   13  C             0       3.079  -1.774   1.169                      C
HETATM   14  C             0       2.848  -1.488  -1.261                      C
HETATM   15  C             0       4.365  -1.235   1.085                      C
HETATM   16  C             0       4.143  -0.960  -1.295                      C
HETATM   17  C             0       4.903  -0.803  -0.133                      C
HETATM   18  H             0       4.958  -1.137   2.003                      H
HETATM   19  H             0       4.564  -0.658  -2.263                      H
HETATM   20  C             0      -2.540  -1.700   0.215                      C
HETATM   21  C             0      -2.990  -1.055   1.382                      C
HETATM   22  C             0      -3.340  -1.755  -0.947                      C
HETATM   23  C             0      -4.211  -0.367   1.329                      C
HETATM   24  C             0      -4.554  -1.069  -0.946                      C
HETATM   25  C             0      -4.991  -0.343   0.172                      C
HETATM   26  H             0      -4.556   0.160   2.228                      H
HETATM   27  H             0      -5.168  -1.081  -1.855                      H
HETATM   28  C             0       2.556  -2.271   2.482                      C
HETATM   29  H             0       3.016  -1.727   3.323                      H
HETATM   30  H             0       1.465  -2.142   2.574                      H
HETATM   31  H             0       2.790  -3.345   2.627                      H
HETATM   32  C             0       2.076  -1.646  -2.534                      C
HETATM   33  H             0       1.356  -2.482  -2.494                      H
HETATM   34  H             0       1.471  -0.750  -2.766                      H
HETATM   35  H             0       2.757  -1.818  -3.384                      H
HETATM   36  C             0       6.248  -0.147  -0.187                      C
HETATM   37  H             0       6.145   0.955  -0.171                      H
HETATM   38  H             0       6.882  -0.422   0.672                      H
HETATM   39  H             0       6.795  -0.396  -1.113                      H
HETATM   40  C             0      -2.255  -1.122   2.687                      C
HETATM   41  H             0      -2.915  -1.540   3.470                      H
HETATM   42  H             0      -1.343  -1.738   2.646                      H
HETATM   43  H             0      -1.935  -0.124   3.033                      H
HETATM   44  C             0      -2.923  -2.550  -2.147                      C
HETATM   45  H             0      -3.262  -3.602  -2.068                      H
HETATM   46  H             0      -3.365  -2.137  -3.068                      H
HETATM   47  H             0      -1.830  -2.549  -2.288                      H
HETATM   48  C             0      -6.259   0.450   0.112                      C
HETATM   49  H             0      -6.102   1.405  -0.426                      H
```

```
HETATM   50  H              0       -7.057  -0.086  -0.431                      H
HETATM   51  H              0       -6.637   0.704   1.116                      H
HETATM   52  H              0        1.128  -4.245   0.980                      H
HETATM   53  H              0       -1.235  -4.030   1.369                      H
HETATM   54  C              0       -0.022   2.734  -0.318                      C
HETATM   55  C              0        1.221   1.788  -0.697                      C
HETATM   56  F              0       -2.360   2.153  -0.704                      F
HETATM   57  F              0       -1.828   2.221   1.356                      F
HETATM   58  F              0       -0.375   3.353  -1.461                      F
HETATM   59  F              0        1.481   1.904  -2.003                      F
HETATM   60 Cl              0        2.743   2.014   0.155                     Cl
HETATM   61 Cl              0        0.368   3.950   0.916                     Cl
END
```

ac5g.pdb

```
TITLE      ac5g.pdb
HETATM    1 Ru              0       -0.071   0.533  -0.030                     Ru
HETATM    2 Cl              0        0.690   0.599   2.255                     Cl
HETATM    3 Cl              0       -0.920   0.176  -2.278                     Cl
HETATM    4  C              0       -0.148  -1.513   0.136                      C
HETATM    5  N              0        0.914  -2.325   0.139                      N
HETATM    6  C              0        0.521  -3.734   0.269                      C
HETATM    7  C              0       -0.997  -3.652   0.458                      C
HETATM    8  N              0       -1.275  -2.219   0.296                      N
HETATM    9  C              0        2.269  -1.881   0.015                      C
HETATM   10  C              0        3.073  -1.804   1.170                      C
HETATM   11  C              0        4.379  -1.326   1.036                      C
HETATM   12  C              0        4.901  -0.950  -0.207                      C
HETATM   13  C              0        4.100  -1.095  -1.342                      C
HETATM   14  C              0        2.784  -1.564  -1.259                      C
HETATM   15  C              0       -2.590  -1.656   0.235                      C
HETATM   16  C              0       -3.320  -1.751  -0.966                      C
HETATM   17  C              0       -4.560  -1.112  -1.040                      C
HETATM   18  C              0       -5.087  -0.401   0.045                      C
HETATM   19  C              0       -4.368  -0.380   1.244                      C
HETATM   20  C              0       -3.126  -1.013   1.368                      C
HETATM   21  C              0        2.566  -2.246   2.508                      C
HETATM   22  C              0        6.275  -0.365  -0.317                      C
HETATM   23  C              0        1.971  -1.717  -2.507                      C
HETATM   24  C              0       -2.813  -2.539  -2.135                      C
HETATM   25  C              0       -6.388   0.330  -0.083                      C
HETATM   26  C              0       -2.410  -1.013   2.684                      C
HETATM   27  H              0        1.042  -4.194   1.127                      H
HETATM   28  H              0        0.818  -4.291  -0.640                      H
HETATM   29  H              0       -1.328  -3.988   1.458                      H
HETATM   30  H              0       -1.561  -4.233  -0.292                      H
HETATM   31  H              0        5.003  -1.236   1.934                      H
HETATM   32  H              0        4.505  -0.835  -2.329                      H
HETATM   33  H              0       -5.122  -1.158  -1.981                      H
HETATM   34  H              0       -4.788   0.133   2.119                      H
HETATM   35  H              0        3.060  -1.689   3.322                      H
HETATM   36  H              0        1.482  -2.084   2.620                      H
HETATM   37  H              0        2.775  -3.321   2.682                      H
HETATM   38  H              0        6.232   0.741  -0.303                      H
HETATM   39  H              0        6.771  -0.647  -1.262                      H
HETATM   40  H              0        6.927  -0.670   0.519                      H
HETATM   41  H              0        2.617  -1.962  -3.367                      H
HETATM   42  H              0        1.421  -0.792  -2.759                      H
HETATM   43  H              0        1.199  -2.502  -2.420                      H
HETATM   44  H              0       -1.713  -2.558  -2.184                      H
```

```
HETATM   45  H             0       -3.166  -2.106  -3.085                          H
HETATM   46  H             0       -3.178  -3.586  -2.101                          H
HETATM   47  H             0       -6.237   1.334  -0.525                          H
HETATM   48  H             0       -7.097  -0.199  -0.742                          H
HETATM   49  H             0       -6.875   0.482   0.895                          H
HETATM   50  H             0       -1.587  -0.275   2.724                          H
HETATM   51  H             0       -3.105  -0.777   3.507                          H
HETATM   52  H             0       -1.946  -1.991   2.906                          H
HETATM   53  C             0       -1.400   1.872   0.225                          C
HETATM   54  F             0       -1.776   2.278   1.420                          F
HETATM   55  F             0       -2.340   2.223  -0.617                          F
HETATM   56  C             0        0.173   2.771  -0.340                          C
HETATM   57  C             0        1.298   1.833  -0.723                          C
HETATM   58 Cl             0        0.500   3.936   0.936                         Cl
HETATM   59  F             0       -0.309   3.391  -1.429                          F
HETATM   60 Cl             0        2.834   1.911   0.123                         Cl
HETATM   61  F             0        1.550   1.845  -2.040                          F
END
```

ac5h.pdb

```
TITLE      ac5h.pdb
HETATM    1 Ru             0        0.158   0.396  -0.076                         Ru
HETATM    2  C             0       -0.503  -3.677   0.416                          C
HETATM    3  C             0       -1.973  -3.260   0.496                          C
HETATM    4  H             0       -0.158  -4.219   1.315                          H
HETATM    5  H             0       -2.412  -3.431   1.498                          H
HETATM    6  C             0       -0.673  -1.350   0.137                          C
HETATM    7  N             0       -1.937  -1.823   0.207                          N
HETATM    8  N             0        0.182  -2.396   0.293                          N
HETATM    9 Cl             0        0.550   0.729   2.266                         Cl
HETATM   10 Cl             0        0.256   0.327  -2.470                         Cl
HETATM   11  C             0        2.661   2.090  -0.549                          C
HETATM   12  C             0       -3.138  -1.058   0.146                          C
HETATM   13  C             0       -3.814  -0.958  -1.084                          C
HETATM   14  C             0       -3.637  -0.446   1.310                          C
HETATM   15  C             0       -5.011  -0.239  -1.129                          C
HETATM   16  C             0       -4.829   0.281   1.217                          C
HETATM   17  C             0       -5.527   0.393   0.009                          C
HETATM   18  H             0       -5.542  -0.152  -2.085                          H
HETATM   19  H             0       -5.224   0.768   2.118                          H
HETATM   20  C             0        1.596  -2.232   0.165                          C
HETATM   21  C             0        2.186  -2.259  -1.119                          C
HETATM   22  C             0        2.389  -2.080   1.325                          C
HETATM   23  C             0        3.555  -1.993  -1.226                          C
HETATM   24  C             0        3.755  -1.838   1.167                          C
HETATM   25  C             0        4.348  -1.758  -0.099                          C
HETATM   26  H             0        4.013  -1.979  -2.224                          H
HETATM   27  H             0        4.373  -1.697   2.064                          H
HETATM   28  C             0       -3.207  -1.532  -2.325                          C
HETATM   29  H             0       -3.897  -1.464  -3.182                          H
HETATM   30  H             0       -2.282  -0.984  -2.593                          H
HETATM   31  H             0       -2.918  -2.593  -2.210                          H
HETATM   32  C             0       -2.894  -0.543   2.607                          C
HETATM   33  H             0       -2.628  -1.585   2.862                          H
HETATM   34  H             0       -1.938   0.015   2.578                          H
HETATM   35  H             0       -3.491  -0.138   3.440                          H
HETATM   36  C             0       -6.786   1.203  -0.073                          C
HETATM   37  H             0       -6.568   2.256  -0.332                          H
HETATM   38  H             0       -7.472   0.823  -0.850                          H
HETATM   39  H             0       -7.330   1.219   0.886                          H
HETATM   40  C             0        1.415  -2.671  -2.336                          C
```

```
HETATM   41  H             0        1.738  -2.109  -3.226                      H
HETATM   42  H             0        1.586  -3.747  -2.541                      H
HETATM   43  H             0        0.330  -2.512  -2.238                      H
HETATM   44  C             0        1.814  -2.254   2.697                      C
HETATM   45  H             0        2.011  -3.280   3.067                      H
HETATM   46  H             0        2.270  -1.553   3.414                      H
HETATM   47  H             0        0.728  -2.083   2.726                      H
HETATM   48  C             0        5.800  -1.419  -0.241                      C
HETATM   49  H             0        5.949  -0.322  -0.266                      H
HETATM   50  H             0        6.399  -1.798   0.605                      H
HETATM   51  H             0        6.230  -1.818  -1.174                      H
HETATM   52  H             0       -2.615  -3.783  -0.235                      H
HETATM   53  H             0       -0.286  -4.316  -0.463                      H
HETATM   54  C             0        1.663   3.010  -0.527                      C
HETATM   55  C             0       -1.361   1.408  -0.115                      C
HETATM   56  F             0        3.045   1.551  -1.688                      F
HETATM   57  F             0        1.068   3.364  -1.650                      F
HETATM   58  F             0       -2.252   1.489  -1.076                      F
HETATM   59  F             0       -1.721   2.300   0.785                      F
HETATM   60 Cl             0        1.235   3.930   0.838                     Cl
HETATM   61 Cl             0        3.621   1.681   0.796                     Cl
END
```

at5e.pdb

```
TITLE       at5e.pdb
HETATM    1 Ru             0       -0.255   0.409   0.015                     Ru
HETATM    2  C             0        0.196  -3.726   0.216                      C
HETATM    3  C             0        1.681  -3.408   0.055                      C
HETATM    4  H             0       -0.193  -4.388  -0.580                      H
HETATM    5  H             0        2.098  -3.768  -0.906                      H
HETATM    6  C             0        0.488  -1.396   0.090                      C
HETATM    7  N             0        1.725  -1.941   0.094                      N
HETATM    8  N             0       -0.420  -2.407   0.133                      N
HETATM    9 Cl             0       -0.716   0.482  -2.324                     Cl
HETATM   10 Cl             0       -0.448   0.561   2.394                     Cl
HETATM   11  C             0       -2.744   2.168   0.124                      C
HETATM   12  C             0        2.975  -1.262   0.022                      C
HETATM   13  C             0        3.700  -1.057   1.210                      C
HETATM   14  C             0        3.478  -0.853  -1.225                      C
HETATM   15  C             0        4.949  -0.438   1.130                      C
HETATM   16  C             0        4.725  -0.216  -1.259                      C
HETATM   17  C             0        5.473  -0.006  -0.096                      C
HETATM   18  H             0        5.519  -0.269   2.053                      H
HETATM   19  H             0        5.123   0.115  -2.226                      H
HETATM   20  C             0       -1.827  -2.175   0.038                      C
HETATM   21  C             0       -2.595  -1.989   1.209                      C
HETATM   22  C             0       -2.435  -2.169  -1.238                      C
HETATM   23  C             0       -3.946  -1.652   1.068                      C
HETATM   24  C             0       -3.789  -1.837  -1.326                      C
HETATM   25  C             0       -4.549  -1.542  -0.189                      C
HETATM   26  H             0       -4.542  -1.477   1.973                      H
HETATM   27  H             0       -4.260  -1.800  -2.317                      H
HETATM   28  C             0        3.092  -1.412   2.532                      C
HETATM   29  H             0        3.785  -1.204   3.363                      H
HETATM   30  H             0        2.167  -0.829   2.708                      H
HETATM   31  H             0        2.805  -2.478   2.600                      H
HETATM   32  C             0        2.691  -1.068  -2.480                      C
HETATM   33  H             0        2.334  -2.110  -2.574                      H
HETATM   34  H             0        1.786  -0.430  -2.519                      H
HETATM   35  H             0        3.294  -0.839  -3.374                      H
HETATM   36  C             0        6.794   0.699  -0.152                      C
```

```
HETATM   37  H              0         6.679   1.781   0.047                         H
HETATM   38  H              0         7.501   0.313   0.602                         H
HETATM   39  H              0         7.269   0.607  -1.144                         H
HETATM   40  C              0        -2.033  -2.250   2.572                         C
HETATM   41  H              0        -2.430  -1.541   3.315                         H
HETATM   42  H              0        -2.316  -3.269   2.905                         H
HETATM   43  H              0        -0.936  -2.170   2.607                         H
HETATM   44  C              0        -1.689  -2.590  -2.466                         C
HETATM   45  H              0        -1.845  -3.671  -2.657                         H
HETATM   46  H              0        -2.043  -2.045  -3.355                         H
HETATM   47  H              0        -0.605  -2.414  -2.392                         H
HETATM   48  C              0        -5.976  -1.104  -0.315                         C
HETATM   49  H              0        -6.042  -0.003  -0.408                         H
HETATM   50  H              0        -6.462  -1.526  -1.211                         H
HETATM   51  H              0        -6.575  -1.383   0.568                         H
HETATM   52  H              0         2.307  -3.832   0.860                         H
HETATM   53  H              0        -0.040  -4.200   1.189                         H
HETATM   54  C              0        -1.757   3.064  -0.088                         C
HETATM   55  C              0         1.279   1.374   0.002                         C
HETATM   56  F              0        -3.200   1.841   1.302                         F
HETATM   57  F              0        -1.484   3.456  -1.317                         F
HETATM   58  F              0        -3.429   1.637  -0.855                         F
HETATM   59 Cl              0        -0.969   3.916   1.160                        Cl
HETATM   60  F              0         2.239   1.369   0.902                         F
HETATM   61 Cl              0         1.631   2.666  -1.130                        Cl
END
```

at5f.pdb

```
TITLE      at5f.pdb
HETATM    1 Ru              0         0.131   0.511  -0.009                        Ru
HETATM    2 Cl              0         0.955   0.365   2.265                        Cl
HETATM    3 Cl              0        -0.603   0.383  -2.309                        Cl
HETATM    4  C              0        -0.015  -1.555  -0.058                         C
HETATM    5  N              0         1.061  -2.346  -0.149                         N
HETATM    6  C              0         0.697  -3.758  -0.308                         C
HETATM    7  C              0        -0.828  -3.731  -0.162                         C
HETATM    8  N              0        -1.129  -2.295  -0.089                         N
HETATM    9  C              0         2.392  -1.823  -0.116                         C
HETATM   10  C              0         3.104  -1.863   1.101                         C
HETATM   11  C              0         4.343  -1.228   1.165                         C
HETATM   12  C              0         4.887  -0.563   0.056                         C
HETATM   13  C              0         4.192  -0.598  -1.153                         C
HETATM   14  C              0         2.947  -1.236  -1.271                         C
HETATM   15  C              0        -2.450  -1.754  -0.023                         C
HETATM   16  C              0        -3.228  -1.672  -1.192                         C
HETATM   17  C              0        -4.488  -1.069  -1.106                         C
HETATM   18  C              0        -4.983  -0.567   0.102                         C
HETATM   19  C              0        -4.206  -0.711   1.258                         C
HETATM   20  C              0        -2.944  -1.311   1.220                         C
HETATM   21  C              0         2.552  -2.575   2.297                         C
HETATM   22  C              0         6.172   0.195   0.183                         C
HETATM   23  C              0         2.297  -1.319  -2.619                         C
HETATM   24  C              0        -2.755  -2.235  -2.497                         C
HETATM   25  C              0        -6.308   0.130   0.158                         C
HETATM   26  C              0        -2.147  -1.497   2.475                         C
HETATM   27  H              0         1.197  -4.373   0.462                         H
HETATM   28  H              0         1.033  -4.123  -1.297                         H
HETATM   29  H              0        -1.184  -4.237   0.754                         H
HETATM   30  H              0        -1.354  -4.187  -1.019                         H
HETATM   31  H              0         4.887  -1.224   2.118                         H
HETATM   32  H              0         4.622  -0.118  -2.041                         H
```

```
HETATM   33  H             0       -5.094  -0.980  -2.017                     H
HETATM   34  H             0       -4.589  -0.348   2.220                     H
HETATM   35  H             0        2.999  -2.190   3.228                     H
HETATM   36  H             0        1.460  -2.443   2.387                     H
HETATM   37  H             0        2.765  -3.662   2.258                     H
HETATM   38  H             0        5.992   1.201   0.609                     H
HETATM   39  H             0        6.665   0.343  -0.793                     H
HETATM   40  H             0        6.885  -0.308   0.858                     H
HETATM   41  H             0        2.031  -0.322  -3.009                     H
HETATM   42  H             0        1.364  -1.904  -2.618                     H
HETATM   43  H             0        2.992  -1.778  -3.346                     H
HETATM   44  H             0       -1.657  -2.264  -2.568                     H
HETATM   45  H             0       -3.114  -1.627  -3.344                     H
HETATM   46  H             0       -3.141  -3.263  -2.652                     H
HETATM   47  H             0       -6.182   1.229   0.124                     H
HETATM   48  H             0       -6.854  -0.094   1.091                     H
HETATM   49  H             0       -6.956  -0.143  -0.692                     H
HETATM   50  H             0       -1.213  -0.902   2.487                     H
HETATM   51  H             0       -2.733  -1.203   3.362                     H
HETATM   52  H             0       -1.843  -2.552   2.612                     H
HETATM   53  C             0       -1.330   1.620   0.428                     C
HETATM   54 Cl             0       -1.855   2.092   2.013                    Cl
HETATM   55  F             0       -2.247   1.989  -0.429                     F
HETATM   56  C             0        0.425   2.794  -0.054                     C
HETATM   57  C             0        1.553   1.947  -0.422                     C
HETATM   58  F             0        0.576   3.410   1.118                     F
HETATM   59 Cl             0       -0.248   3.832  -1.276                    Cl
HETATM   60  F             0        2.619   2.008   0.372                     F
HETATM   61  F             0        1.969   1.963  -1.689                     F
END
```

at5PB.pdb

```
TITLE      at5PB.pdb
HETATM    1 Ru             0        0.029   0.454  -0.012                    Ru
HETATM    2  C             0        1.048  -3.700  -0.147                     C
HETATM    3  C             0       -0.475  -3.801   0.007                     C
HETATM    4  H             0        1.601  -4.214   0.659                     H
HETATM    5  H             0       -0.784  -4.292   0.949                     H
HETATM    6  C             0        0.149  -1.570  -0.022                     C
HETATM    7  N             0       -0.901  -2.394   0.013                     N
HETATM    8  N             0        1.294  -2.255  -0.083                     N
HETATM    9 Cl             0        0.857   0.319   2.260                    Cl
HETATM   10 Cl             0       -0.707   0.337  -2.301                    Cl
HETATM   11  C             0        1.246   1.930  -0.384                     C
HETATM   12  C             0       -2.258  -1.934   0.008                     C
HETATM   13  C             0       -2.976  -1.912  -1.203                     C
HETATM   14  C             0       -2.835  -1.490   1.215                     C
HETATM   15  C             0       -4.251  -1.341  -1.202                     C
HETATM   16  C             0       -4.114  -0.926   1.165                     C
HETATM   17  C             0       -4.822  -0.817  -0.037                     C
HETATM   18  H             0       -4.805  -1.288  -2.148                     H
HETATM   19  H             0       -4.562  -0.554   2.095                     H
HETATM   20  C             0        2.586  -1.640  -0.107                     C
HETATM   21  C             0        3.064  -1.068  -1.302                     C
HETATM   22  C             0        3.349  -1.605   1.079                     C
HETATM   23  C             0        4.282  -0.376  -1.263                     C
HETATM   24  C             0        4.558  -0.911   1.067                     C
HETATM   25  C             0        5.028  -0.269  -0.088                     C
HETATM   26  H             0        4.657   0.082  -2.187                     H
HETATM   27  H             0        5.145  -0.856   1.992                     H
HETATM   28  C             0       -2.417  -2.509  -2.458                     C
```

```
HETATM   29  H              0       -2.834  -2.016  -3.351                     H
HETATM   30  H              0       -1.321  -2.405  -2.520                     H
HETATM   31  H              0       -2.668  -3.586  -2.535                     H
HETATM   32  C              0       -2.119  -1.633   2.522                     C
HETATM   33  H              0       -1.741  -2.661   2.673                     H
HETATM   34  H              0       -1.242  -0.963   2.608                     H
HETATM   35  H              0       -2.791  -1.398   3.364                     H
HETATM   36  C              0       -6.151  -0.129  -0.080                     C
HETATM   37  H              0       -6.025   0.953  -0.279                     H
HETATM   38  H              0       -6.798  -0.525  -0.881                     H
HETATM   39  H              0       -6.693  -0.213   0.877                     H
HETATM   40  C              0        2.338  -1.197  -2.606                     C
HETATM   41  H              0        3.049  -1.424  -3.419                     H
HETATM   42  H              0        1.566  -1.984  -2.595                     H
HETATM   43  H              0        1.811  -0.267  -2.884                     H
HETATM   44  C              0        2.902  -2.316   2.320                     C
HETATM   45  H              0        3.281  -3.357   2.345                     H
HETATM   46  H              0        3.284  -1.812   3.223                     H
HETATM   47  H              0        1.805  -2.347   2.412                     H
HETATM   48  C              0        6.297   0.526  -0.051                     C
HETATM   49  H              0        6.115   1.542   0.346                     H
HETATM   50  H              0        7.056   0.065   0.605                     H
HETATM   51  H              0        6.738   0.648  -1.055                     H
HETATM   52  H              0       -0.962  -4.337  -0.826                     H
HETATM   53  H              0        1.414  -4.100  -1.112                     H
HETATM   54  C              0       -0.127   2.732   0.070                     C
HETATM   55  C              0       -1.367   1.783   0.417                     C
HETATM   56  F              0        2.251   2.262   0.414                     F
HETATM   57  F              0        1.642   2.190  -1.626                     F
HETATM   58  F              0        0.226   3.427   1.167                     F
HETATM   59  F              0       -2.394   1.917  -0.421                     F
HETATM   60 Cl              0       -1.998   2.002   2.048                    Cl
HETATM   61 Cl              0       -0.592   3.857  -1.223                    Cl
END
```

at5g.pdb

```
TITLE      at5g.pdb
HETATM    1 Ru              0        0.017   0.510  -0.021                    Ru
HETATM    2 Cl              0       -0.733   0.381  -2.308                    Cl
HETATM    3 Cl              0        0.837   0.370   2.258                    Cl
HETATM    4  C              0        0.159  -1.548  -0.013                     C
HETATM    5  N              0       -0.882  -2.386   0.032                     N
HETATM    6  C              0       -0.454  -3.790   0.040                     C
HETATM    7  C              0        1.072  -3.684  -0.063                     C
HETATM    8  N              0        1.306  -2.235  -0.056                     N
HETATM    9  C              0       -2.243  -1.940   0.006                     C
HETATM   10  C              0       -2.947  -1.938  -1.214                     C
HETATM   11  C              0       -4.235  -1.398  -1.231                     C
HETATM   12  C              0       -4.833  -0.885  -0.074                     C
HETATM   13  C              0       -4.135  -0.966   1.135                     C
HETATM   14  C              0       -2.843  -1.499   1.202                     C
HETATM   15  C              0        2.602  -1.629  -0.078                     C
HETATM   16  C              0        3.357  -1.564   1.109                     C
HETATM   17  C              0        4.578  -0.886   1.080                     C
HETATM   18  C              0        5.065  -0.295  -0.092                     C
HETATM   19  C              0        4.325  -0.435  -1.270                     C
HETATM   20  C              0        3.098  -1.108  -1.290                     C
HETATM   21  C              0       -2.356  -2.521  -2.461                     C
HETATM   22  C              0       -6.183  -0.240  -0.135                     C
HETATM   23  C              0       -2.136  -1.621   2.518                     C
HETATM   24  C              0        2.901  -2.231   2.371                     C
```

```
HETATM   25  C              0       6.347   0.480  -0.080                         C
HETATM   26  C              0       2.359  -1.278  -2.582                         C
HETATM   27  H              0      -0.910  -4.329  -0.809                         H
HETATM   28  H              0      -0.794  -4.284   0.969                         H
HETATM   29  H              0       1.477  -4.127  -0.992                         H
HETATM   30  H              0       1.598  -4.156   0.787                         H
HETATM   31  H              0      -4.780  -1.363  -2.183                         H
HETATM   32  H              0      -4.602  -0.599   2.058                         H
HETATM   33  H              0       5.160  -0.808   2.007                         H
HETATM   34  H              0       4.715  -0.021  -2.208                         H
HETATM   35  H              0      -2.774  -2.040  -3.360                         H
HETATM   36  H              0      -1.263  -2.388  -2.509                         H
HETATM   37  H              0      -2.577  -3.605  -2.541                         H
HETATM   38  H              0      -6.093   0.840  -0.360                         H
HETATM   39  H              0      -6.816  -0.677  -0.926                         H
HETATM   40  H              0      -6.724  -0.320   0.823                         H
HETATM   41  H              0      -2.812  -1.367   3.350                         H
HETATM   42  H              0      -1.255  -0.957   2.596                         H
HETATM   43  H              0      -1.768  -2.650   2.690                         H
HETATM   44  H              0       1.803  -2.290   2.442                         H
HETATM   45  H              0       3.247  -1.677   3.259                         H
HETATM   46  H              0       3.310  -3.258   2.449                         H
HETATM   47  H              0       6.166   1.541   0.176                         H
HETATM   48  H              0       6.844   0.471  -1.065                         H
HETATM   49  H              0       7.059   0.092   0.668                         H
HETATM   50  H              0       1.573  -0.512  -2.727                         H
HETATM   51  H              0       3.049  -1.207  -3.438                         H
HETATM   52  H              0       1.840  -2.251  -2.645                         H
HETATM   53  C              0       1.342   1.809  -0.419                         C
HETATM   54  F              0       1.701   2.126  -1.642                         F
HETATM   55  F              0       2.278   2.246   0.386                         F
HETATM   56  C              0      -0.270   2.763   0.099                         C
HETATM   57  C              0      -1.419   1.864   0.440                         C
HETATM   58 Cl              0      -0.583   3.846  -1.249                        Cl
HETATM   59  F              0       0.219   3.432   1.150                         F
HETATM   60  F              0      -2.449   1.902  -0.411                         F
HETATM   61 Cl              0      -2.052   1.975   2.076                        Cl
END
```

at5h.pdb

```
TITLE      at5h.pdb
HETATM    1 Ru              0      -0.173   0.349   0.033                        Ru
HETATM    2  C              0       0.681  -3.713   0.118                         C
HETATM    3  C              0       2.137  -3.242   0.072                         C
HETATM    4  H              0       0.416  -4.376  -0.726                         H
HETATM    5  H              0       2.661  -3.550  -0.853                         H
HETATM    6  C              0       0.745  -1.364   0.054                         C
HETATM    7  N              0       2.028  -1.779   0.115                         N
HETATM    8  N              0      -0.059  -2.460   0.041                         N
HETATM    9 Cl              0      -0.688   0.595  -2.285                        Cl
HETATM   10 Cl              0      -0.222   0.398   2.430                        Cl
HETATM   11  C              0      -2.867   2.097   0.210                         C
HETATM   12  C              0       3.194  -0.964   0.026                         C
HETATM   13  C              0       3.916  -0.672   1.196                         C
HETATM   14  C              0       3.616  -0.498  -1.233                         C
HETATM   15  C              0       5.083   0.088   1.085                         C
HETATM   16  C              0       4.782   0.273  -1.298                         C
HETATM   17  C              0       5.527   0.572  -0.152                         C
HETATM   18  H              0       5.651   0.326   1.995                         H
HETATM   19  H              0       5.119   0.643  -2.275                         H
HETATM   20  C              0      -1.474  -2.331  -0.103                         C
```

```
HETATM   21  C              0       -2.297  -2.233   1.039                      C
HETATM   22  C              0       -2.027  -2.296  -1.404                      C
HETATM   23  C              0       -3.659  -1.975   0.850                      C
HETATM   24  C              0       -3.390  -2.027  -1.541                      C
HETATM   25  C              0       -4.212  -1.828  -0.425                      C
HETATM   26  H              0       -4.302  -1.866   1.734                      H
HETATM   27  H              0       -3.816  -1.952  -2.549                      H
HETATM   28  C              0        3.388  -1.091   2.532                      C
HETATM   29  H              0        4.069  -0.794   3.346                      H
HETATM   30  H              0        2.402  -0.624   2.725                      H
HETATM   31  H              0        3.235  -2.184   2.610                      H
HETATM   32  C              0        2.822  -0.798  -2.468                      C
HETATM   33  H              0        2.543  -1.866  -2.537                      H
HETATM   34  H              0        1.870  -0.230  -2.497                      H
HETATM   35  H              0        3.387  -0.539  -3.378                      H
HETATM   36  C              0        6.759   1.422  -0.240                      C
HETATM   37  H              0        6.523   2.490  -0.071                      H
HETATM   38  H              0        7.509   1.142   0.519                      H
HETATM   39  H              0        7.235   1.356  -1.233                      H
HETATM   40  C              0       -1.776  -2.475   2.422                      C
HETATM   41  H              0       -2.212  -1.765   3.143                      H
HETATM   42  H              0       -2.049  -3.496   2.758                      H
HETATM   43  H              0       -0.684  -2.368   2.495                      H
HETATM   44  C              0       -1.190  -2.590  -2.610                      C
HETATM   45  H              0       -1.147  -3.682  -2.799                      H
HETATM   46  H              0       -1.609  -2.116  -3.511                      H
HETATM   47  H              0       -0.155  -2.227  -2.507                      H
HETATM   48  C              0       -5.645  -1.427  -0.593                      C
HETATM   49  H              0       -5.732  -0.325  -0.652                      H
HETATM   50  H              0       -6.084  -1.829  -1.522                      H
HETATM   51  H              0       -6.271  -1.751   0.256                      H
HETATM   52  H              0        2.737  -3.607   0.924                      H
HETATM   53  H              0        0.432  -4.251   1.053                      H
HETATM   54  C              0       -1.906   3.049   0.251                      C
HETATM   55  C              0        1.294   1.435  -0.022                      C
HETATM   56  F              0       -3.435   1.745  -0.925                      F
HETATM   57  F              0       -1.394   3.451   1.396                      F
HETATM   58  F              0        2.181   1.638   0.926                      F
HETATM   59  F              0        1.610   2.270  -0.992                      F
HETATM   60 Cl              0       -1.392   3.917  -1.120                     Cl
HETATM   61 Cl              0       -3.553   1.406   1.609                     Cl
END
```
